# Supplementary material for: Construction of poly-N-heterocyclic scaffolds via the controlled reactivity of Cu-allenylidene intermediates
Source: Commun Chem. 2021 Nov 18;4:158. doi: 10.1038/s42004-021-00596-x (PMC9814594; doi:10.1038/s42004-021-00596-x)
Supplement: Supplementary file 1 — Description of Additional Supplementary Files [file 42004_2021_596_MOESM1_ESM.pdf]

## Description of Additional Supplementary Files

**File Name:** Supplementary Data 1

**Description**: cif file of CCDC2026703

**File Name:** Supplementary Data 2

**Description**: cif file of CCDC2026704

**File Name:** Supplementary Data 3

**Description**: cif file of CCDC2026705

**File Name:** Supplementary Data 4

**Description**: cif file of CCDC2026702

**File Name:** Supplementary Data 5

**Description**: Electronic Calculations PDF file
