# Supplementary material for: Construction of poly-N-heterocyclic scaffolds via the controlled reactivity of Cu-allenylidene intermediates
Source: Commun Chem. 2021 Nov 18;4:158. doi: 10.1038/s42004-021-00596-x (PMC9814594; doi:10.1038/s42004-021-00596-x)
Supplement: Supplementary file 2 — Supplementary Information [file 42004_2021_596_MOESM2_ESM.pdf]

Supplemental Information

Construction of poly-*N*-heterocyclic scaffolds via the controlled reactivity of Cu-allenylidene intermediates

Malla Reddy Gannarapu,[1] Takanori Imai,[1] Kentaro Iwaki,[1] Seiji Tsuzuki[2] and Norio Shibata* [1,3]

[1] *Department of Nanopharmaceutical Sciences & Department of Life Science and Applied Chemistry, Nagoya Institute of Technology, Gokiso, Showa-ku, Nagoya 466-8555, Japan.*

[2] *Research Center for Computational Design of Advanced Functional Materials, National Institute of Advanced Industrial Science and Technology, Tsukuba, Ibaraki 305-8568, Japan.*

[3] *Institute of Advanced Fluorine-Containing Materials, Zhejiang Normal University, 688 Yingbin Avenue, 321004 Jinhua, China.*

[*] *Correspondence and requests for materials should be addressed to N.S. (email: nozshiba@nitech.ac.jp*

*E-mail: nozshiba@nitech.ac.jp

CONTENTS

Supplementary Methods

1. General information S3
2. Supplementary details for condition optimization S4
3. General experimental procedures for the synthesis of starting materials S8
4. General Procedure and Spectral Data of Products S13
5. Synthetic utility I S24
6. Synthetic utility II S30
7. General Procedure for Cu-catalyzed decarboxylative annulation reaction of Me-benzoxazinanone S34

8. **Applications of spiro carbazole/indole skeleton** **S38**
9. **General Procedure for**
Cu-catalyzed decarboxylative annulation reaction of 4-ethynyl benzoxazinanone **S39**
10. **X-Ray crystallographic analysis of products** **S41**
11. **Computational methods** **S42**
12. **HPLC Data and NMR Data** **S44**
13. **Supplementary References** **S179**

Supplementary Methods

1. General information

All reactions were performed in oven-dried glassware under a positive pressure of nitrogen or argon. Solvents were transferred via syringe and were introduced into the reaction vessels through a rubber septum. All solvents were dried by standard method. All of the reactions were monitored by thin-layer chromatography (TLC) carried out on 0.25 mm Merck silica gel (60-F254). The TLC plates were visualized with UV light. All of the reaction products were purified by column chromatography and was carried out on a column packed with silica gel 60N spherical neutral size 50-63 mm. The ^{1}H NMR (300 MHz and 500 MHz) and ^{19}F NMR (282 MHz) spectra (with hexafluorobenzene (δ ppm -162.2) as an internal standard) as for solution in $CDCl_3$ and DMSO were recorded on a Varian Mercury 300 and BRUKER 500 Ultra Shield TR. ^{13}C NMR (126 MHz) spectra for solution in $CDCl_3$ was recorded on a BRUKER 500 Ultra Shield TR. The chemical shifts (δ) are expressed in ppm downfield from internal TMS (δ = 0.00) and coupling constants (J) are reported in hertz (Hz). The hexafluorobenzene (C_6F_6) [δ = -162.2 ($CDCl_3$)] was used as internal standard for ^{19}F NMR. The following abbreviations were used to explain the multiplicities: s = singlet, d = doublet, t = triplet, q = quartet, m = multiplet, br = broad. Mass spectra were recorded on a SHIMADZU GCMS-QP5050A (EI-MS) and SHIMADZU LCMS-2020 (ESI-MS). High resolution mass spectrometry (HRMS) was carried out on an electron impact ionization mass spectrometer with a micro-TOF analyzer. Infrared spectra were recorded on a JASCO FT/IR-4100 spectrometer. Melting points were recorded on a BUCHI M-565. Optical rotations were measured on a SEPA-300 instrument (HORIBA Ltd, Kyoto, Japan). HPLC analyses were performed on a JASCOLC-2000 Plus series using 4.6 x 250 mm CHIRALPAK series. Commercially available chemicals were obtained from Aldrich Chemical Co., Alfa Aesar, TCI and used as received unless otherwise noted. Solvents acetonitrile, ethyl acetate, ethanol, Dioxane, DMF, DCM, THF and Toluene were dried and distilled before use.

2. Supplementary details for condition optimization

Supplementary Table 1. Ligand effects for copper-catalyzed annulation. [a]

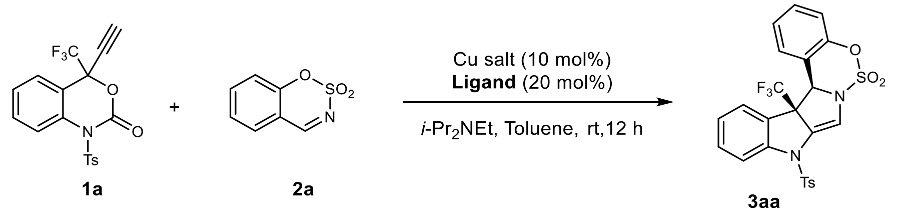

Entry	Ligand	dr[b]	Yield (%)[b]	*ee* (%)[c]
1	**L1**	>95:5	47	15
**2**	**L2**	**>99:1**	**70**	**96**
3	**L3**	-	28	-
4	**L4**	>95:5	32	75
5	**L5**	-	Trace	-
6	**L6**	-	14	-
7	***R*,*R*-DBFOX/Ph**	-	Trace	-
8	**(*R*)-BINAP**	-	NR	-

[a] Reactions were carried out with **1a** (0.1 mmol), **2a** (0.1 mmol), CuOTf.1/2C_6H_6 (10 mol%), ligand (20 mol%), *i*-Pr_2NEt (DIPEA, 2.4 equiv) in Toluene at room temperature.
[b] Determined by ^{19}F NMR analysis of the reaction mixture.
[c] The *ee* was determined by chiral HPLC analysis.

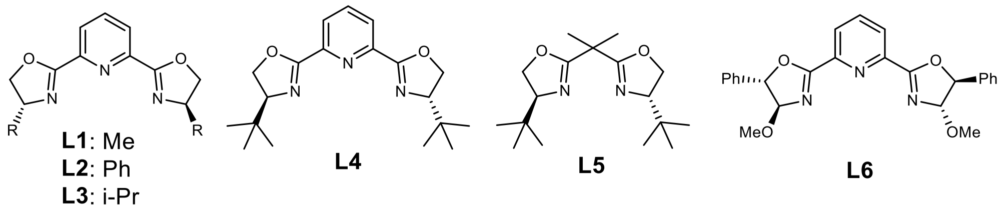

Supplementary Table 2. Solvent effects for copper-catalyzed annulation. [a]

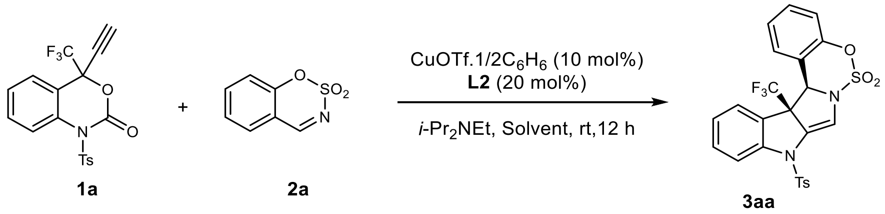

Entry	Solvent	dr[b]	Yield (%)[b]	*ee* (%)[c]
1	**Toluene**	>99:1	**70**	**96**
2	THF	>99:1	65	96
3	ACN	>99:1	36	25
4	DCM	>99:1	42	25
5	Xylene	>99:1	68	96
6	EA	>99:1	58	96

[a] Reactions were carried out with **1a** (0.1 mmol), **2a** (0.1 mmol), $CuOTf.1/2C_6H_6$ (10 mol%), **L2** (20 mol%), *i*-Pr_2NEt (DIPEA, 2.4 equiv) in solvent at room temperature.
[b] Determined by ^{19}F NMR analysis of the reaction mixture.
[c] The *ee* was determined by chiral HPLC analysis.

Supplementary Table 3. Screen of Cu salts for copper-catalyzed annulation. [a]

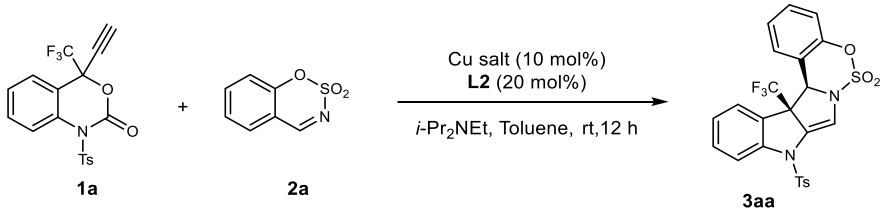

Entry	Copper salt	dr[b]	Yield (%)[b]	*ee* (%)[c]
1	$CuOTf.1/2C_6H_6$	>99:1	70	96
2	$Cu(OTf)_2$	>99:1	71	99
3	CuOTf-Toluene	>99:1	69	99
4	CuI	-	21	-
**5[d]**	**$Cu(OTf)_2$**	**>99:1**	**68**	**98**

[a] Reactions were carried out with **1a** (0.1 mmol), **2a** (0.1 mmol), Cu salt (10 mol%), **L2** (20 mol%), *i*-Pr_2NEt (DIPEA, 2.4 equiv) in Toluene at room temperature.
[b] Determined by ^{19}F NMR analysis of the reaction mixture.
[c] The *ee* was determined by chiral HPLC analysis.
[d] Using Cu salt (5 mol%), **L2** (10 mol%).

Supplementary Table 4. Substrate ratio effects for copper-catalyzed annulation.[a]

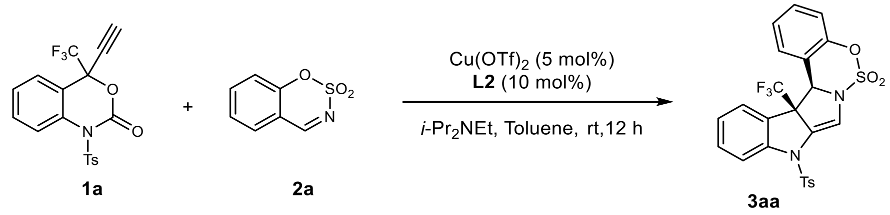

Entry	**2a** (eq mol)	dr[b]	Yield (%)[b]	*ee* (%)[c]
1	1.0	>99:1	68	98
2	**1.1**	**>99:1**	**71**	**98**
3	1.2	>99:1	65	98
4	1.5	>99:1	67	98

[a] Reactions were carried out with **1a** (0.1 mmol), **2a**, Cu(OTf)$_2$ (5 mol%), **L2** (10 mol%), *i*-Pr$_2$NEt (DIPEA, 2.4 equiv) in Toluene at room temperature.
[b] Determined by ^{19}F NMR analysis of the reaction mixture.
[c] The *ee* was determined by chiral HPLC analysis.

Supplementary Table 5. Base effect for copper-catalyzed annulation.[a]

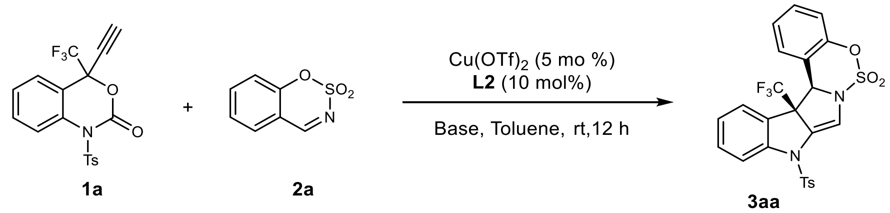

Entry	Base	Ratio	dr[b]	Yield (%)[c]	*ee* (%)[d]
1	DIPEA	2.4	>99:1	71	98
2	DIPEA	1.2	>99:1	77	98
3	DIPEA	**0.5**	**>99:1**	**88**	**98**
4	DIPEA	0.3	>99:1	79	97
5	DIPEA	0.15	>99:1	40	95
6[d]	-	-	-	NR	-
7	TEA	0.5	>99:1	63	98
8	*N*-Ethylmorpholine	0.5	>99:1	70	98
9	K_2CO_3	0.5	-	24	-

[a] Reactions were carried out with **1a** (0.1 mmol), **2a** (0.11 mmol), Cu(OTf)$_2$ (5 mol%), **L2** (10 mol%), Base in Toluene at room temperature.
[b] Determined by ^{19}F NMR analysis of the reaction mixture.
[c] The *ee* was determined by chiral HPLC analysis.
[d] No base was used

Supplementary Table 6. Temperature effects for copper-catalyzed annulation. [a]

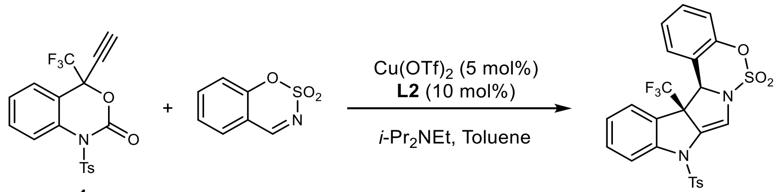

1a **2a** **3aa**

Entry	Temp (°C)	Time (h)	dr[b]	Yield (%)[c]	*ee* (%)[d]
1	40	12	>99:1	78	95
2	R.T.	12	>99:1	88	98
3	0	24	>99:1	73	>99
**4**	**10**	**16**	**>99:1**	**94**	**>99**

[a] Reactions were carried out with **1a** (0.1 mmol), **2a** (0.11 mmol), $Cu(OTf)_2$ (5 mol%), **L2** (10 mol%), *i*-Pr_2NEt (DIPEA, 0.5 equiv.) in Toluene.
[b] Determined by ^{19}F NMR analysis of the reaction mixture.
[c] The *ee* was determined by chiral HPLC analysis.

Supplementary Table 7. The screening of ligand for the transformation of 4a to 5a

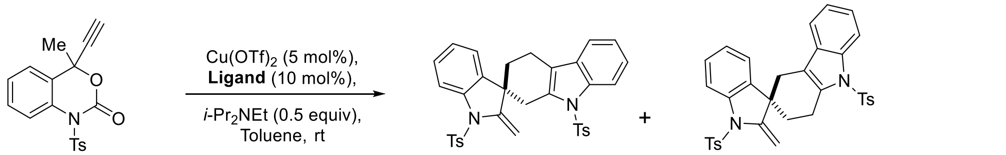

4a **5a (2C)** **5a (3C)**

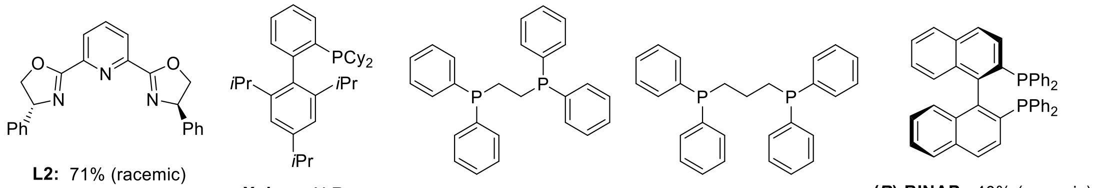

L2: 71% (racemic)
2C:3C = 85:15

Xphos : N.R.

dppe : 56%
2C:3C = 88:12

dppp : N.R.

(*R*)-BINAP : 40% (racemic)
2C:3C = 85 : 15

3. General experimental procedures for the synthesis of starting materials.

CF_3-Benzoxazinanone (**1**),[1] Me-benzoxazinanone (**4**)[1] and benzoxazinanone[2] were prepared according to literature.

3.1 Synthesis of substituted ethynyl benzoxazinanones (1a-1f) (Method A)

Benzoxazinanones (**1a-1e**) were prepared according to literature[1]. Overall reaction scheme for the synthesis of 6-methyl-4-trifluoromethyl ethynyl benzoxazinanones (**1f**) is showing below.

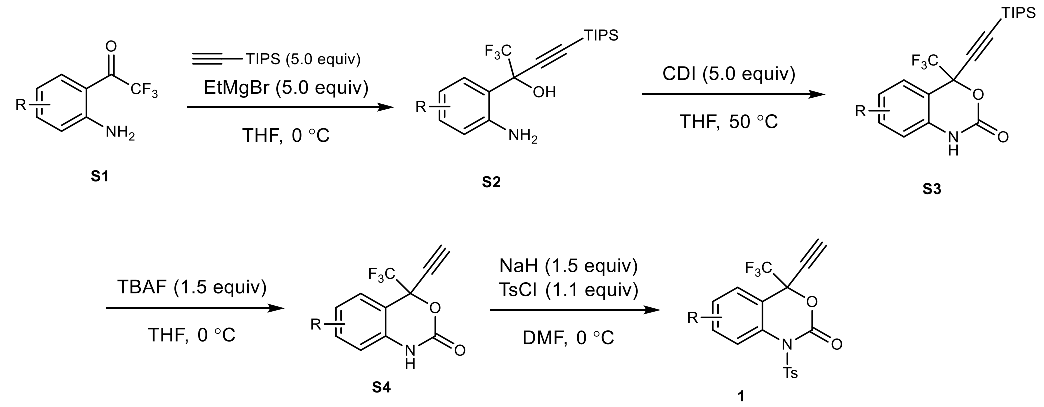

Synthesis of 6-Methyl-4-(trifluoromethyl)-4-((triisopropylsilyl)ethynyl)-1,4-dihydro-2*H*-benzo[*d*][1,3]oxazin-2-one (S3f):

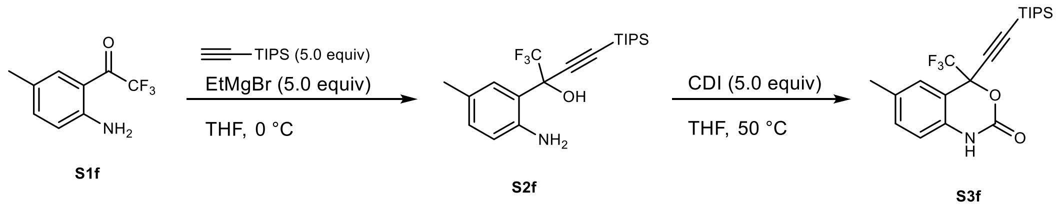

Under N_2 atmosphere, in a flame dried 20 mL round bottom flask, triisopropylethynylsilane (5.50 mmol, 5.0 equiv) and ethylmagnesium bromide (1.0 M in THF, 5.50 mol, 5.0 equiv) was stirred for 1 h at 0 °C. To this solution a THF (5 mL) solution of **S1f** (1.1 mmol, 1.0 equiv) was added dropwise and the mixture was allowed to stir for 2 h at 0 °C. After that, the reaction mixture was quenched by NH_4Cl aqueous solution followed by extraction with ethyl acetate (3 × 10 mL). Combined organic layers were finally washed with brine solution, dried over anhydrous Na_2SO_4 and then solvent was removed under reduced pressure. In a flame dried 30 mL round bottom flask, the crude product **S2f**, and dry THF (11 mL) were added. To this suspension carbonyldiimidazole (CDI) (5.5 mmol, 5.0 equiv) was added and the mixture was heated to 50 °C for 12 h. After that, the reaction mixture was added NH_3 aqueous solution followed by extraction with DCM (3 × 10 mL). Combined organic layers were finally washed with brine solution, dried over anhydrous Na_2SO_4 and then solvent was removed under reduced pressure. The crude product was purified by

flash column chromatography (using 8:2 hexane/ethyl acetate) to obtain the pure product **S3f** as a white solid (359 mg, yield: 79% in two steps), m.p. = 153.7 – 154.7 °C. **^{1}H NMR** (500 MHz, $CDCl_3$) δ 8.95 (s, 1H), 7.37 (s, 1H), 7.19 (m, 1H), 6.78 (d, *J* = 10.0 Hz, 1H), 2.34 (s, 3H), 1.11 – 1.15 (m, 21H). **^{13}C NMR** (126 MHz, $CDCl_3$) δ 149.2, 133.7, 132.3, 132.2, 128.4, 122.3 (q, *J* = 287.6 Hz), 114.7, 113.0, 97.1, 93.9, 79.3 (q, *J* = 34.5 Hz), 20.8, 18.4, 10.9. **^{19}F NMR** (282 MHz, $CDCl_3$) δ −81.5 (s, 3F). **IR (KBr)**: 3158, 2948, 1727, 1617, 1515, 1346, 1251, 1189, 1170, 1045, 883, 819 cm^{-1}. **HRMS (ESI)** calculated for $C_{21}H_{28}F_3NO_2NaSi$ $[M+Na]^+$: 434.1739, found: 434.1726.

Synthesis of 4-ethynyl-6-methyl-4-(trifluoromethyl)-1,4-dihydro-2*H*-benzo[*d*][1,3]oxazin-2-one (S4f):

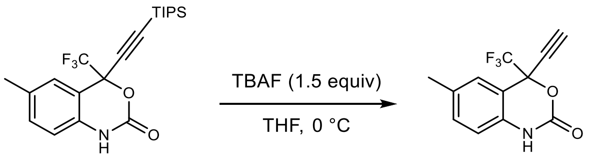

S3f **S4f**

In 25 mL round bottom flask, compound **S3f** (0.83 mmol, 1.0 equiv) and THF (5 mL) was stirred at 0 °C, and TBAF solution (1.0 M in THF, 1.25 mmol, 1.5 equiv) was added dropwise. The reaction completed in 10 min by TLC followed by extraction with ethyl acetate (3 × 10 mL). Combined organic layers were finally washed with brine solution, dried over anhydrous Na_2SO_4 and then solvent was removed under reduced pressure. The crude product was washed by pentane to obtain the pure product **S4f**. Compound **S4f** was obtained as a white solid (228.9 mg, Yield: 97%), m.p. = 156.8 – 157.8 °C. **^{1}H NMR** (500 MHz, $CDCl_3$) δ 8.73 (s, 1H), 7.33 (s, 1H), 7.20 (d, *J* = 7.9 Hz, 1H), 6.94 (d, *J* = 7.9 Hz, 1H), 2.94 (s, 1H), 2.36 (s, 3H). **^{13}C NMR** (126 MHz, $CDCl_3$) δ 147.7, 133.7, 132.5, 132.4, 127.8, 122.2 (q, *J* = 287.0 Hz), 115.0, 112.2, 78.3, 75.2, 20.3. **^{19}F NMR** (282 MHz, $CDCl_3$) δ −81.4 (s, 3F). **IR (KBr)**: 3280, 2967, 2136, 2095, 1739, 1617, 1517, 1344, 1251, 1170, 1085, 827 cm^{-1}. **HRMS (ESI)** calculated for $C_{12}H_8F_3NO_2Na$ $[M+Na]^+$: 278.0405, found: 278.0403.

Synthesis of 4-ethynyl-6-methyl-1-tosyl-4-(trifluoromethyl)-1,4-dihydro-2*H*-benzo[*d*][1,3] oxazin-2-one (1f):

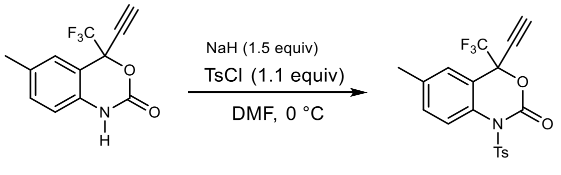

In a flame dried 100 mL round bottom flask, compound **S4f** (2 mmol, 1.0 equiv) was suspended in dry DMF (6 mL) and allowed to cool to 0 °C. To this solution NaH (60% dispersion in mineral oil, 3 mmol, 1.5 equiv) was added and the mixture was allowed to stir for 30 min under N_2 atmosphere. After 30 min, the solution of *p*-toluenesulfonyl chloride (2.2 mmol, 1.1 equiv) in dry DMF (3 mL) was added dropwise to the reaction mixture and stirred the reaction mixture at 0 °C until completion of the reaction. After that, the reaction mixture was poured into crushed ice followed by extraction with ethyl acetate (3 ×30 mL). Combined organic layers were finally washed with brine solution, dried over anhydrous Na_2SO_4 and then solvent was removed under reduced pressure. The crude product was purified by flash column chromatography (using 9:1 hexane/ethyl acetate) to obtain the pure product **1f**. Compound **1f** was obtained as a white solid (99 mg, Yield: 30%), m.p. = 158.1 – 159.1 °C. **^{1}H NMR** (500 MHz, $CDCl_3$) δ 8.06 (d, *J* = 8.5 Hz, 2H), 7.64 (d, *J* = 8.5 Hz, 1H), 7.42 (s, 1H), 7.38 (d, *J* = 7.9 Hz, 2H), 7.34 – 7.36 (m, 1H), 2.93 (s, 1H), 2.47 (s, 3H), 2.42 (s, 3H). **^{13}C NMR** (126 MHz, $CDCl_3$) δ 146.2, 145.4, 136.6, 134.8, 131.8, 131.2, 129.6, 129.5, 127.2, 121.5 (q, *J* = 287.0 Hz), 120.9, 118.9, 88.2, 77.8 (q, *J* = 35.4 Hz), 74.1, 21.8, 20.9. **^{19}F NMR** (282 MHz, $CDCl_3$) δ −78.5 (s, 3F). **IR (KBr)**: 3274, 2929, 2136, 1758, 1594, 1502, 1216, 1081, 968, 935, 817, 665 cm^{-1}. **HRMS (ESI)** calculated for $C_{19}H_{14}F_3NO_4NaS$ $[M+Na]^+$: 432.0493, found: 432.0492.

3.2 Synthesis of substituted benzoxathiazine (2a-2e, 2g-2j) (Method B)

Benzoxathiazine (**2a-2e, 2g-2j**) were prepared according to literature[3, 4]. Overall reaction scheme for the synthesis of substituted benzoxathiazine (**2a-2e, 2g-2j**) is showing below.

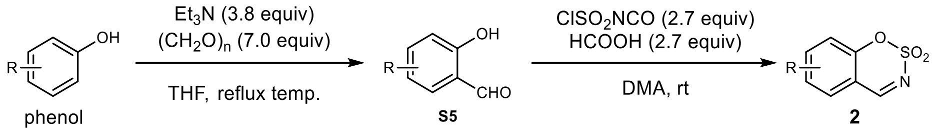

Anhydrous magnesium chloride (7.5 mmol, 1.5 equiv) was dried further by heating under vacuum. To this THF (20 mL), substituted-phenol (5.0 mmol, 1.0 equiv), triethylamine (12.5 mmol, 2.5

equiv) and paraformaldehyde (22.5 mmol, 4.5 equiv) were added at room temperature. The mixture was heated to reflux for 18 h after which the reaction was quenched via the slow addition of HCl (2M, 12 mL). The reaction was extracted with ethyl acetate, dried over anhydrous Na_2SO_4 and then solvent was removed under reduced pressure. The crude product was purified by flash column chromatography (using 8:2 hexane/ethyl acetate) to obtain the pure product **S5**.

Anhydrous formic acid (11.6 mmol, 2.7 equiv) was added dropwise to neat chlorosulfonyl isocyanate (11.6 mmol, 2.7 equiv) at 0 °C with rapid stirring. The resulting viscous suspension was stirred at 0 °C for 1 h and afforded the $ClSO_2NH_2$. To a solution of **S5** (4.3 mmol, 1.0 equiv) in DMA (29 mL) at 0 °C was carefully added freshly prepared $ClSO_2NH_2$ in small portions and the resulting solution was stirred at room temperature for 24 h. The reaction was quenched carefully with ice-cold water and the mixture was transferred to a separating funnel containing CH_2Cl_2. The aqueous layer was separated and extracted with CH_2Cl_2, and the combined organic layers were washed with saturated $NaHCO_3$ aqueous solution, dried over $MgSO_4$, filtered through a short pad of silica using CH_2Cl_2 as eluent and concentrated in vacuum. The residue was purified via flash column chromatography on silica gel (using 8:2 hexane/ethyl acetate) to obtain the pure product **2**.

3.3 Synthesis of 5-nitrobenzoxathiazine (2f) (Method C)

5-nitrobenzoxathiazine (**2f**) were prepared according to literature[4, 5]. Overall reaction scheme for the synthesis of **2f** is showing below.

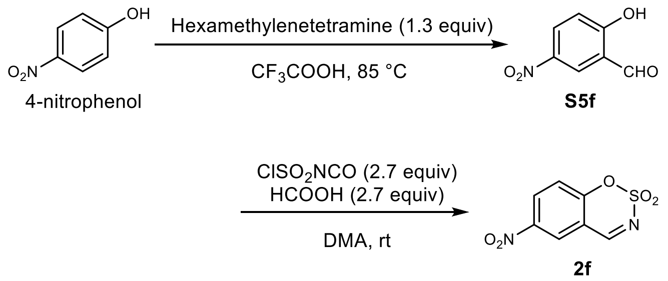

A mixture of 4-nitrophenol (5.0 mmol, 1.0 equiv), hexamethylenetetramine (6.5 mmol, 1.3 equiv) in trifluoroacetic acid (5.8 mL) was heated at 85 °C for 15 h. The reaction mixture was cooled to room temperature and concentrated under reduced pressure. The mixture was diluted with H_2O and then neutralized with Na_2CO_3 to pH 5. The precipitate formed was collected by filtration, washed with water and dried to afford the pure product **S5f** as a yellow solid.

Anhydrous formic acid (11.6 mmol, 2.7 equiv) was added dropwise to neat chlorosulfonyl isocyanate (11.6 mmol, 2.7 equiv) at 0 °C with rapid stirring. The resulting viscous suspension was stirred at 0 °C for 1 h and afforded the $ClSO_2NH_2$. To a solution of **S5f** (4.3 mmol, 1.0 equiv) in DMA (29 mL) at 0 °C was carefully added freshly prepared $ClSO_2NH_2$ in small portions and the resulting solution was stirred at room temperature for 24 h. The reaction was quenched carefully with ice-cold water and the mixture was transferred to a separating funnel containing CH_2Cl_2. The aqueous layer was separated and extracted with CH_2Cl_2, and the combined organic layers were washed with saturated $NaHCO_3$ aqueous solution, dried over $MgSO_4$, filtered through a short pad of silica using CH_2Cl_2 as eluent and concentrated in vacuum. The residue was purified via flash column chromatography on silica gel (using 8:2 hexane/ethyl acetate) to obtain the pure product **2f** as a white solid.

4. General Procedure and Spectral Data of Products

4.1 General Procedure for the asymmetric annulation reaction (Method D)

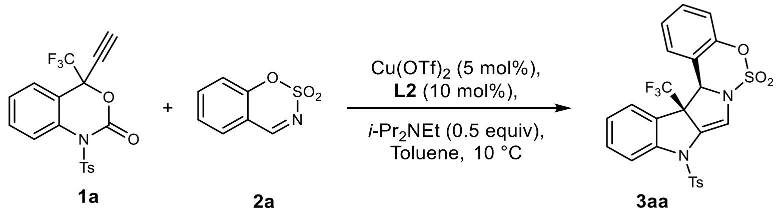

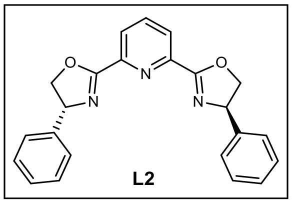

In an argon filled glove box, a flame-dried 10 mL Schlenk tube was charged with copper(II) trifluoromethanesulfonate (1.81 mg, 0.005 mmol, 5 mol%), 2,6-bis[(4*R*)-phenyl-2-oxazolin-2-yl]-pyridine **L2** (3.69 mg, 0.01 mmol, 10 mol%) and anhydrous Toluene (1 mL). The resulting solution was stirred for 1 h at 80 °C. In an argon filled glove box, ethynyl benzoxazinanones **1** (0.1 mmol), benzoxathiazine **2** (0.11 mmol) and DIPEA (8.7 μL, 0.05 mmol, 0.5 equiv) were added. The resulting solution was stirred at 10 °C until complete conversion of ethynyl benzoxazinanones (monitored by TLC). The reaction was quenched by saturated NH_4Cl aqueous solution (2 mL). The resulting solution was extracted with ethyl acetate (5 mL × 3). The combined organic layers were dried over Na_2SO_4, filtered and concentrated under *vacuum*. The diastereomeric ratio and crude yield were determined by ^{19}F NMR analysis of the crude reaction mixture. The residue was purified by flash silica gel chromatography (Toluene) to afford the title compound **3**. The characterization data of **3** are summarized below.

4.2 Characterization data of product

(13b*S*,13c*R*)-9-Tosyl-13b-(trifluoromethyl)-13b,13c-dihydro-9*H*-benzo[5',6'][1,2,3] oxathiazino[3',4':1,5]pyrrolo[3,4-*b*]indole 6,6-dioxide (3aa):

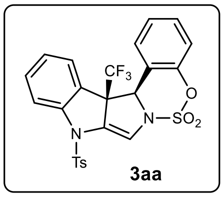

Following the general method **D**, compound **3aa** was obtained as a white solid (90.8 mg, Yield: 85%), m.p. = 113.4 – 114.4 °C. The enantiomeric excess (99% *ee*) was determined by chiral HPLC using CHIRALPAK® IC (n-hexane/isopropanol = 85.0/15.0, flow rate 1.0 mL/min, λ = 254 nm) t (major) = 15.458 min, t (minor) = 30.608 min). $[\alpha]^{25}_{D}$ = −113.1 (c = 0.2, $CHCl_3$, 99% *ee*). **^{1}H NMR** (500 MHz, $CDCl_3$) δ 7.78 – 7.80 (m, 3H), 7.44 – 7.50 (m, 4H), 7.36 – 7.39 (m, 1H), 7.18 – 7.28 (m, 4H), 6.96 (s, 1H), 5.80 (s, 1H), 2.38 (s, 3H). **^{13}C NMR** (126 MHz, $CDCl_3$) δ 150.7, 147.1, 145.4, 134.0, 131.5, 131.1, 129.9, 128.4, 127.6, 127.3, 126.7, 126.4, 125.9, 124.7, 123.3 (q, *J* = 287.0 Hz), 123.7, 122.2, 120.3, 118.6, 118.4, 64.7 (q, *J* = 26.9 Hz), 21.7. **^{19}F NMR** (282 MHz, $CDCl_3$) δ −75.0 (s, 3F). **IR (KBr)**: 3135, 3012, 1594, 1496, 1405, 1376, 1172, 842, 757, 665, 572 cm^{-1}. **HRMS (ESI)** calculated for $C_{24}H_{17}F_3N_2O_5NaS_2$ $[M+Na]^+$: 557.0429, found: 557.0429.

(13b*S*,13c*R*)-2-Methyl-9-tosyl-13b-(trifluoromethyl)-13b,13c-dihydro-9*H*-benzo[5',6'][1,2,3] oxathiazino[3',4':1,5]pyrrolo[3,4-*b*]indole 6,6-dioxide (3ab):

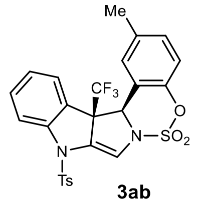

3ab

Following the general method **D**, compound **3ab** was obtained as a white solid (91.8 mg, Yield: 84%), m.p. = 112.7 – 113.7 °C. The enantiomeric excess (99% *ee*) was determined by chiral HPLC using CHIRALPAK® IC (n-hexane/isopropanol = 85.0/15.0, flow rate 1.0 mL/min, λ = 254 nm) t (major) = 18.150 min, t (minor) = 41.242 min). $[\alpha]^{25}_{D}$ = −169.9 (c = 0.4, $CHCl_3$, 99% *ee*). **^{1}H NMR** (500 MHz, $CDCl_3$) 7.78 – 7.80 (m, 3H), 7.46 – 7.50 (m, 1H), 7.42 (d, *J* = 7.3 Hz, 1H), 7.24 – 7.28 (m, 4H), 7.19 – 7.22 (m, 1H), 7.11 (d, *J* = 8.2 Hz, 1H), 6.95 (s, 1H), 5.73 (s, 1H), 2.43 (s, 3H), 2.37 (s, 3H). **^{13}C NMR** (126 MHz, $CDCl_3$) δ 148.6, 147.2, 145.4, 136.4, 134.0, 131.7, 131.5, 129.9, 128.6, 127.6, 127.3, 125.8, 124.7, 123.8, 123.3 (q, *J* = 287.0 Hz), 120.0, 118.7, 118.0, 114.8, 69.7, 64.6 (q, *J* = 26.6 Hz), 21.7, 21.0. **^{19}F NMR** (282 MHz, $CDCl_3$) δ −75.0 (s, 3F). **IR (KBr)**: 3124, 3052, 2940, 1600, 1460, 1376, 1170, 1091, 950, 835, 761, 661 cm^{-1}. **HRMS (ESI)** calculated for $C_{25}H_{19}F_3N_2O_5NaS_2$ $[M+Na]^+$: 571.0585, found: 571.0587.

(13b*S*,13c*R*)-2-Methoxy-9-tosyl-13b-(trifluoromethyl)-13b,13c-dihydro-9*H*-benzo[5',6'][1,2,3]oxathiazino[3',4':1,5]pyrrolo[3,4-*b*]indole 6,6-dioxide (3ac):

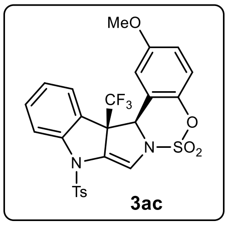

Following the general method **D**, compound **3aa** was obtained as a yellow solid (95.7 mg, Yield: 85%), m.p. = 113.2 – 114.1 °C. The enantiomeric excess (99% *ee*) was determined by chiral HPLC using CHIRALPAK® IA (n-hexane/isopropanol = 95.0/5.0, flow rate 1.0 mL/min, λ = 254 nm) t (major) = 48.742 min, t (minor) = 62.825 min). $[\alpha]^{25}_{D}$ = −159.4 (c = 0.6, $CHCl_3$, 99% *ee*). **^{1}H NMR** (500 MHz, $CDCl_3$) δ 7.77 – 7.79 (m, 3H), 7.41 – 7.47 (m, 2H), 7.27 (d, *J* = 7.6 Hz, 2H), 7.16 – 7.20 (m, 2H), 6.94 – 6.98 (m, 3H), 5.69 (s, 1H), 3.86 (s, 3H), 2.38 (s, 3H). **^{13}C NMR** (126 MHz, $CDCl_3$) δ 157.4, 147.2, 145.5, 144.3, 134.0, 131.5, 129.9, 127.5, 127.3, 125.8, 124.7, 123.7, 123.3 (q, *J* = 287.3 Hz), 121.2, 119.3, 118.7, 115.3, 114.8, 114.2, 69.7, 64.6 (q, *J* = 26.9 Hz), 55.9, 21.7. **^{19}F NMR** (282 MHz, $CDCl_3$) δ −74.9 (s, 3F). **IR (KBr)**: 3135, 3012, 2840, 1596, 1496, 1405, 1376, 1230, 1172, 1091, 943, 842, 757 cm^{-1}. **HRMS (ESI)** calculated for $C_{25}H_{19}F_3N_2O_6NaS_2$ $[M+Na]^+$: 587.0534, found: 587.0511.

(13b*S*,13c*R*)-2-Fluoro-9-tosyl-13b-(trifluoromethyl)-13b,13c-dihydro-9*H*-benzo[5',6'][1,2,3]oxathiazino[3',4':1,5]pyrrolo[3,4-*b*]indole 6,6-dioxide (3ad):

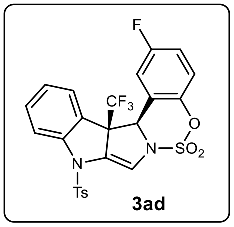

Following the general method **D**, compound **3ad** was obtained as a yellow solid (78.0 mg, Yield: 71%), m.p. = 85.6 – 86.5 °C. The enantiomeric excess (95% *ee*) was determined by chiral HPLC using CHIRALPAK® IC (n-hexane/isopropanol = 85.0/15.0, flow rate 1.0 mL/min, λ = 254 nm) t (major) = 11.733 min, t (minor) = 23.408 min). $[\alpha]^{25}_{D}$ = −104.7 (c = 0.6, $CHCl_3$, 95% *ee*). **^{1}H NMR** δ 7.77 – 7.80 (m, 3H), 7.47 – 7.50 (m, 1H), 7.41 (d, *J* = 7.6 Hz, 1H), 7.27 (d, *J* = 8.2 Hz, 2H), 7.16 – 7.24 (m, 4H), 6.95 (s, 1H), 5.72 (s, 1H), 2.38 (s, 3H). **^{13}C NMR** (126 MHz, $CDCl_3$) δ 159.8 (d,

J = 247.9 Hz), 147.1, 146.7, 145.6, 133.9, 131.7, 129.9, 127.6, 127.3, 125.8, 124.8, 123.3, 123.3 (q, *J* = 287.9 Hz), 122.1 (d, *J* = 8.2 Hz), 120.2 (d, *J* = 7.3 Hz), 118.5, 118.2 (d, *J* = 23.6 Hz), 115.2 (d, *J* = 25.4 Hz), 114.8, 69.3, 64.8 (q, *J* = 26.9 Hz), 21.7. **^{19}F NMR** (282 MHz, $CDCl_3$) δ −74.9 (s, 3F), −114.3 – −114.4 (m, 1F). **IR (KBr)**: 3139, 3075, 2921, 1599, 1492, 1405, 1170, 944, 840 761, 663, 572 cm^{-1}. **HRMS (ESI)** calculated for $C_{24}H_{16}F_4N_2O_5NaS_2$ [M+Na]$^+$: 575.0334, found: 575.0330.

(13b*S*,13c*R*)-2-Bromo-9-tosyl-13b-(trifluoromethyl)-13b,13c-dihydro-9*H*-benzo[5',6'][1,2,3] oxathiazino[3',4':1,5]pyrrolo[3,4-*b*]indole 6,6-dioxide (3ae):

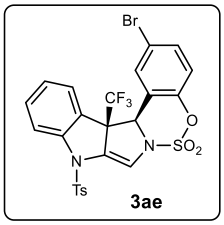

Following the general method **D**, compound **3ae** was obtained as a yellow solid (90.2 mg, Yield: 73%), m.p. = 87.5 – 88.5 °C. The enantiomeric excess (95% *ee*) was determined by chiral HPLC using CHIRALPAK® IA (n-hexane/isopropanol = 95.0/5.0, flow rate 1.0 mL/min, λ = 254 nm) t (major) = 27.125 min, t (minor) = 34.067 min). $[\alpha]^{25}_{D}$ = −170.5 (c = 0.2, $CHCl_3$, 95% *ee*). **^{1}H NMR** (500 MHz, $CDCl_3$) δ 7.78 – 7.80 (m, 3H), 7.58 – 7.61 (m, 2H), 7.48 – 7.51 (m, 1H), 7.38 (d, *J* = 7.6 Hz, 1H), 7.28 (d, *J* = 7.9 Hz, 2H), 7.22 – 7.25 (m, 1H), 7.13 (d, *J* = 9.2 Hz, 1H), 6.95 (s, 1H), 5.74 (s, 1H), 2.39 (s, 3H). **^{13}C NMR** (126 MHz, $CDCl_3$) δ 149.8, 147.1, 145.5, 134.2, 133.9, 131.7, 131.2, 129.9, 127.7, 127.3, 125.7, 124.9, 123.2, 123.2 (q, *J* = 282.9 Hz), 122.1, 120.6, 119.2, 118.4, 114.8, 69.1, 64.8 (q, *J* = 26.9 Hz), 21.7. **^{19}F NMR** (282 MHz, $CDCl_3$) δ −74.9 (s, 3F). **IR (KBr)**: 3127, 2921, 1600, 1477, 1405, 1170, 1116, 831, 782, 663, 576 cm^{-1}. **HRMS (ESI)** calculated for $C_{24}H_{16}BrF_3N_2O_5NaS_2$ [M+Na]$^+$: 634.9534, found: 634.9532.

(13b*S*,13c*R*)-2-Nitro-9-tosyl-13b-(trifluoromethyl)-13b,13c-dihydro-9*H*-benzo[5',6'][1,2,3] oxathiazino[3',4':1,5]pyrrolo[3,4-*b*]indole 6,6-dioxide (3af):

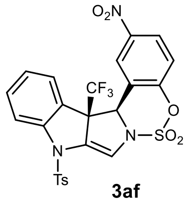

Following the general method **D**, compound **3af** was obtained as a white solid (30.2 mg, Yield: 52%), m.p. = 113.2 – 114.1 °C. The enantiomeric excess (92% *ee*) was determined by chiral HPLC using CHIRALPAK® IC (n-hexane/isopropanol = 85.0/15.0, flow rate 1.0 mL/min, λ = 254 nm) t (major) = 20.125 min, t (minor) = 30.692 min). $[\alpha]^{25}_{D}$ = −66.4 (c = 0.1, $CHCl_3$, 92% *ee*). **^{1}H NMR** (500 MHz, $CDCl_3$) δ 8.42 (d, *J* = 2.4 Hz, 1H), 8.38 (dd, *J* = 8.9, 2.7 Hz, 1H), 7.79 – 7.81 (m, 3H), 7.51 – 7.54 (m, 1H), 7.44 (d, *J* = 7.6 Hz, 1H), 7.42 (d, *J* = 8.9 Hz, 1H), 7.28 – 7.30 (m, 2H), 7.25 – 7.27 (m, 1H), 6.99 (s, 1H), 5.89 (s, 1H), 2.39 (s, 3H). **^{13}C NMR** (126 MHz, $CDCl_3$) δ 154.9, 147.0, 145.7, 145.2, 133.8, 132.0, 130.0, 127.9, 127.3, 126.5, 125.8, 125.1, 124.3, 123.2 (q, *J* = 287.6 Hz), 122.7, 121.6, 120.0, 117.8, 114.9, 69.2, 65.0 (q, *J* = 26.6 Hz), 21.7. **^{19}F NMR** (282 MHz, $CDCl_3$) δ −74.7 (s, 3F). **IR (KBr)**: 3127, 3052, 1537, 1409, 1376, 1172, 944, 779, 671 cm^{-1}. **HRMS (EI)** calculated for $C_{24}H_{16}F_3N_3O_7S_2$ $[M]^+$: 579.0382, found: 579.0403.

(13b*S*,13c*R*)-3-Methyl-9-tosyl-13b-(trifluoromethyl)-13b,13c-dihydro-9*H*-benzo[5',6'][1,2,3] oxathiazino[3',4':1,5]pyrrolo[3,4-*b*]indole 6,6-dioxide (3ag):

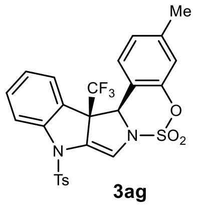

Following the general method **D**, compound **3ag** was obtained as a white solid (79.5 mg, Yield: 72%), m.p. = 132.4 – 133.4 °C. The enantiomeric excess (99% *ee*) was determined by chiral HPLC using CHIRALPAK® IC (n-hexane/isopropanol = 85.0/15.0, flow rate 1.0 mL/min, λ = 254 nm) t (major) = 15.975 min, t (minor) = 37.100 min). $[\alpha]^{25}_{D}$ = −124.4 (c = 0.8, $CHCl_3$, 99% *ee*). **^{1}H NMR** (500 MHz, $CDCl_3$) δ 7.78 – 7.80 (m, 3H), 7.42 – 7.49 (m, 2H), 7.35 (d, *J* = 7.9 Hz, 1H), 7.27 (d, *J* = 7.9 Hz, 2H), 7.16 – 7.21 (m, 2H), 7.05 (s, 1H), 6.95 (s, 1H), 5.75 (s, 1H), 2.41 (s, 3H), 2.38 (s, 3H). **^{13}C NMR** (126 MHz, $CDCl_3$) δ 150.5, 147.1, 145.4, 142.0, 134.0, 131.5, 129.9, 128.0, 127.6, 127.3, 125.9, 124.7, 123.8, 123.3 (q, *J* = 287.0 Hz), 120.7, 119.9, 118.7, 115.2, 114.8, 69.6, 64.6

(q, J = 26.9 Hz), 21.7, 21.3. **^{19}F NMR** (282 MHz, $CDCl_3$) δ −75.0 (s, 3F). **IR (KBr)**: 3435, 3127, 1600, 1457, 1402, 1369, 1303, 1241, 1170, 1108, 952, 798 cm^{-1}. **HRMS (ESI)** calculated for $C_{25}H_{19}F_3N_2O_5NaS_2$ $[M+Na]^+$: 571.0585, found: 571.0587.

(13b*S*,13c*R*)-3-Methoxy-9-tosyl-13b-(trifluoromethyl)-13b,13c-dihydro-9*H*-benzo[5',6'][1,2,3]oxathiazino[3',4':1,5]pyrrolo[3,4-*b*]indole 6,6-dioxide (3ah):

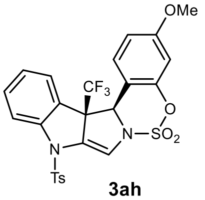

3ah

Following the general method **D**, compound **3ah** was obtained as a yellow solid (93.3 mg, Yield: 83%), m.p. = 102.5 – 103.4 °C. The enantiomeric excess (97% *ee*) was determined by chiral HPLC using CHIRALPAK® IC (n-hexane/isopropanol = 85.0/15.0, flow rate 1.0 mL/min, λ = 254 nm) t (major) = 18.317 min, t (minor) = 37.942 min). $[\alpha]^{25}_{D}$ = −111.1 (c = 0.6, $CHCl_3$, 97% *ee*). **^{1}H NMR** (500 MHz, $CDCl_3$) δ 7.78 – 7.79 (m, 3H), 7.45 – 7.48 (m, 1H), 7.40 (d, J = 7.3 Hz, 1H), 7.34 (d, J = 8.5 Hz, 1H), 7.27 (d, J = 7.9 Hz, 2H), 7.17 – 7.20 (m, 1H), 6.94 (s, 1H), 6.90 (d, J = 10.4 Hz, 1H), 6.74 (s, 1H), 5.72 (s, 1H), 3.85 (s, 3H), 2.38 (s, 3H). **^{13}C NMR** (126 MHz, $CDCl_3$) δ 161.4, 151.6, 147.1, 145.4, 134.0, 131.4, 129.9, 129.0, 127.8, 127.3, 125.8, 124.7, 123.8, 123.4 (q, J = 287.0 Hz), 118.6, 114.8, 113.3, 109.7, 105.2, 69.5, 64.4 (q, J = 26.6 Hz), 55.7, 21.7. **^{19}F NMR** (282 MHz, $CDCl_3$) δ −75.1 (s, 3F). **IR (KBr)**: 3135, 2937, 1627, 1510, 1459, 1402, 1170, 1089, 952, 796, 754, 659 cm^{-1}. **HRMS (ESI)** calculated for $C_{25}H_{19}F_3N_2O_6S_2$ $[M]^+$: 564.0637, found: 564.0615.

(13b*S*,13c*R*)-3-Chloro-9-tosyl-13b-(trifluoromethyl)-13b,13c-dihydro-9*H*-benzo[5',6'][1,2,3] oxathiazino[3',4':1,5]pyrrolo[3,4-*b*]indole 6,6-dioxide (3ai):

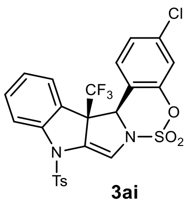

3ai

Following the general method **D**, compound **3ai** was obtained as a yellow solid (94.3 mg, Yield: 91%), m.p. = 104.7 – 105.6 °C. The enantiomeric excess (98% *ee*) was determined by chiral HPLC using CHIRALPAK® IC (n-hexane/isopropanol = 85.0/15.0, flow rate 1.0 mL/min, λ = 254 nm) t (major) = 12.000 min, t (minor) = 22.567 min). $[\alpha]^{25}_{D}$ = −115.4 (c = 0.5, $CHCl_3$, 98% *ee*). **^{1}H NMR** (500 MHz, $CDCl_3$) δ 7.78 – 7.79 (m, 3H), 7.47 – 7.50 (m, 1H), 7.33 – 7.42 (m, 3H), 7.26 – 7.28 (m, 3H), 7.18 – 7.21 (m, 1H), 6.95 (s, 1H), 5.76 (s, 1H), 2.39 (s, 3H). **^{13}C NMR** (126 MHz, $CDCl_3$) δ 151.0, 147.1, 145.6, 136.7, 133.9, 131.7, 129.9, 129.2, 127.7, 127.3, 126.8, 125.7, 124.7, 123.3, 123.3 (q, *J* = 287.3 Hz), 120.9, 118.4, 117.1, 114.9, 69.3, 64.7 (q, *J* = 26.9 Hz), 21.7. **^{19}F NMR** (282 MHz, $CDCl_3$) δ −74.9 (s, 3F). **IR (KBr)**: 3135, 3064, 2929, 1600, 1459, 1405, 1170, 923, 782, 657, 572 cm^{-1}. **HRMS (ESI)** calculated for $C_{24}H_{16}ClF_3N_2O_5NaS_2$ $[M+Na]^+$: 591.0039, found: 591.0026.

(13b*S*,13c*R*)-9-Tosyl-13b-(trifluoromethyl)-13b,13c-dihydro-9*H*-naphtho[2'',1'':5',6'][1,2,3] oxathiazino[3',4':1,5]pyrrolo[3,4-*b*]indole 6,6-dioxide (3aj):

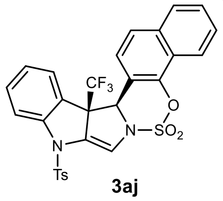

3aj

Following the general method **D**, compound **3aj** was obtained as a yellow solid (101.6 mg, Yield: 87%), m.p. = 115.2 – 116.0 °C. The enantiomeric excess (99% *ee*) was determined by chiral HPLC using CHIRALPAK® IC (n-hexane/isopropanol = 85.0/15.0, flow rate 1.0 mL/min, λ = 254 nm) t (major) = 18.683 min, t (minor) = 28.933 min). $[\alpha]^{25}_{D}$ = −99.7 (c = 0.2, $CHCl_3$, 99% *ee*). **^{1}H NMR** (500 MHz, $CDCl_3$) δ 8.19 – 8.21 (m, 1H), 7.88 – 7.90 (m, 1H), 7.79 – 7.84 (m, 4H), 7.63 – 7.65 (m, 2H), 7.44 – 7.53 (m, 3H), 7.20 – 7.27 (m, 3H), 7.02 (s, 1H), 5.90 (s, 1H), 2.36 (s, 3H). **^{13}C NMR** (126 MHz, $CDCl_3$) δ 147.2, 146.6, 145.5, 134.3, 133.9, 131.6, 129.9, 129.9, 128.3, 128.0, 127.9, 127.8, 127.3, 126.3, 126.0, 125.2, 124.8, 123.6, 123.4, 123.4 (q, *J* = 287.0 Hz), 121.5, 114.8, 113.7, 70.1, 65.0 (q, *J* = 26.9 Hz), 21.6. **^{19}F NMR** (282 MHz, $CDCl_3$) δ −75.2 (s, 3F). **IR (KBr)**: 3135, 3060, 1598, 1459, 1403, 1243, 1170, 1089, 883, 796 cm^{-1}. **HRMS (ESI)** calculated for $C_{28}H_{19}F_3N_2O_5NaS_2$ $[M+Na]^+$: 607.0585, found: 607.0586.

(13b*S*,13c*R*)-9-Tosyl-11,13b-bis(trifluoromethyl)-13b,13c-dihydro-9*H*-benzo[5',6'][1,2,3] oxathiazino[3',4':1,5]pyrrolo[3,4-*b*]indole 6,6-dioxide (3ba):

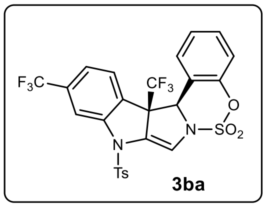

Following the general method **D**, compound **3ba** was obtained as a white solid (30.8 mg, Yield: 81%), m.p. = 128.2 – 129.1 °C. The enantiomeric excess (92% *ee*) was determined by chiral HPLC using CHIRALPAK® IG (n-hexane/isopropanol = 90.0/10.0, flow rate 1.0 mL/min, λ = 254 nm) t (major) = 21.417 min, t (minor) = 25.692 min). $[\alpha]^{25}_{D}$ = −120.7 (c = 0.1, $CHCl_3$, 92% *ee*). **^{1}H NMR** (500 MHz, $CDCl_3$) δ 8.03 (s, 1H), 7.79 (d, *J* = 8.2 Hz, 2H), 7.45 – 7.56 (m, 4H), 7.40 – 7.41 (m, 1H), 7.25 – 7.31 (m, 3H), 7.02 (s, 1H), 5.82 (s, 1H), 2.40 (s, 3H). **^{13}C NMR** (126 MHz, $CDCl_3$) δ 150.7, 147.7, 146.0, 133.9 (q, *J* = 33.0 Hz), 133.7, 131.4, 130.1, 128.2, 127.2, 126.9, 126.6, 126.3, 123.2 (q, *J* = 273.1 Hz), 123.0 (q, *J* = 287.3 Hz), 121.6 (q, *J* = 3.6 Hz), 120.5, 119.7, 118.0, 111.7 (q, *J* = 3.6 Hz), 69.4, 64.5 (q, *J* = 27.2 Hz), 21.7. **^{19}F NMR** (282 MHz, $CDCl_3$) δ −63.3 (s, 3F), −75.0 (s, 3F). **IR (KBr)**: 3139, 2948, 1599, 1402, 1377, 1323, 1178, 1137, 962, 902, 796 cm^{-1}. **HRMS (ESI)** calculated for $C_{25}H_{16}F_6N_2O_5NaS_2$ $[M+Na]^+$: 625.0303, found: 625.0308.

Methyl (13b*S*,13c*R*)-9-tosyl-13b-(trifluoromethyl)-13b,13c-dihydro-9*H*-benzo[5',6'][1,2,3] oxathiazino[3',4':1,5]pyrrolo[3,4-*b*]indole-11-carboxylate 6,6-dioxide (3ca):

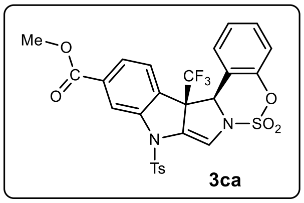

Following the general method **D**, compound **3ca** was obtained as a white solid (42.4 mg, Yield: 72%), m.p. = 134.0 – 134.7 °C. The enantiomeric excess (96% *ee*) was determined by chiral HPLC using CHIRALPAK® IC (n-hexane/isopropanol = 95.0/5.0, flow rate 1.0 mL/min, λ = 254 nm) t (major) = 32.133 min, t (minor) = 39.2 min). $[\alpha]^{25}_{D}$ = −120.7 (c = 0.1, $CHCl_3$, 96% *ee*). $[\alpha]^{25}_{D}$ = −88.6 (c = 0.3, $CHCl_3$). **^{1}H NMR** (500 MHz, $CDCl_3$) δ 8.39 (d, *J* = 1.2 Hz, 1H), 7.89 (dd, *J* = 7.9, 1.5 Hz, 1H), 7.81 (d, *J* = 8.5 Hz, 2H), 7.47 – 7.51 (m, 3H), 7.38 – 7.41 (m, 1H), 7.24 – 7.29 (m, 3H), 6.99 (s, 1H), 5.82 (s, 1H), 3.99 (s, 3H), 2.39 (s, 3H). **^{13}C NMR** (126 MHz, $CDCl_3$) δ 165.7,

150.7, 147.4, 145.7, 133.8, 133.5, 131.3, 130.0, 128.3, 128.0, 127.3, 127.2, 126.6, 126.1, 125.9, 122.8 (q, *J* = 264.6 Hz), 120.4, 119.2, 118.1, 115.3, 69.4, 64.6 (q, *J* = 26.9 Hz,), 52.8, 21.7. **^{19}F NMR** (282 MHz, $CDCl_3$) δ −74.9 (s, 3F). **IR (KBr)**: 3127, 2956, 1720, 1400, 1295, 1170, 906, 734, 663, 578 cm^{-1}. **HRMS (ESI)** calculated for $C_{26}H_{19}F_3N_2O_7NaS_2$ $[M+Na]^+$: 615.0483, found: 615.0483.

(13b*S*,13c*R*)-12-Fluoro-9-tosyl-13b-(trifluoromethyl)-13b,13c-dihydro-9*H*-benzo[5',6'][1,2,3]oxathiazino[3',4':1,5]pyrrolo[3,4-*b*]indole 6,6-dioxide (3da):

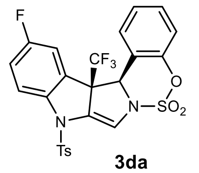

3da

Following the general method **D**, compound **3da** was obtained as a yellow solid (50.5 mg, Yield: 91%), m.p. = 104.8 – 105.8 °C. The enantiomeric excess (99% *ee*) was determined by chiral HPLC using CHIRALPAK® IC (n-hexane/isopropanol = 85.0/15.0, flow rate 1.0 mL/min, λ = 254 nm) t (major) = 12.333 min, t (minor) = 24.967 min). $[\alpha]^{25}_D$ = −131.4 (c = 0.6, $CHCl_3$, 99% *ee*). **^{1}H NMR** (500 MHz, $CDCl_3$) δ 7.76 – 7.78 (m, 3H), 7.47 – 7.50 (m, 1H), 7.39 – 7.43 (m, 2H), 7.13 – 7.29 (m, 5H), 6.97 (s, 1H), 5.78 (s, 1H), 2.39 (s, 3H). **^{13}C NMR** (126 MHz, $CDCl_3$) δ 159.5 (d, *J* = 245.2 Hz), 150.7, 145.6, 143.3, 133.7, 131.3, 129.9, 128.2, 127.5, 127.3, 126.6, 125.0 (d, *J* = 8.2 Hz), 123.1 (q, *J* = 287.3 Hz), 120.4, 119.3, 118.4 (d, *J* = 23.6 Hz), 118.1, 116.0 (d, *J* = 8.2 Hz), 113.5 (d, *J* = 24.5 Hz), 69.5, 64.5 (q, *J* = 27.2 Hz), 21.7. **^{19}F NMR** (282 MHz, $CDCl_3$) δ −74.9 (s, 3F), −116.7 – −116.8 (m, 1F). **IR (KBr)**: 3141, 2960, 1599, 1471, 1403, 1363, 1170, 1087, 952, 908, 815 cm^{-1}. **HRMS (ESI)** calculated for $C_{24}H_{16}F_4N_2O_5NaS_2$ $[M+Na]^+$: 575.0334, found: 575.0342.

(13b*S*,13cR)-12-Fluoro-2-methyl-9-tosyl-13b-(trifluoromethyl)-13b,13c-dihydro-9*H*-benzo[5',6'][1,2,3]oxathiazino[3',4':1,5]pyrrolo[3,4-*b*]indole 6,6-dioxide (3db):

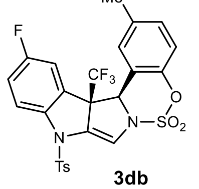

3db

Following the general method **D**, compound **3db** was obtained as a yellow solid (47.0 mg, Yield: 83%), m.p. = 107.2 – 108.1 °C. The enantiomeric excess (98% *ee*) was determined by chiral HPLC using CHIRALPAK® IG (n-hexane/isopropanol = 95.0/5.0, flow rate 1.0 mL/min, λ = 254 nm) t (major) = 69.183 min, t (minor) = 76.500 min). $[\alpha]^{25}_{D}$ = −174.3 (c = 0.6, $CHCl_3$, 98% *ee*). **^{1}H NMR** (500 MHz, $CDCl_3$) δ 7.75 – 7.78 (m, 3H), 7.26 – 7.29 (m, 3H), 7.18 – 7.21 (m, 2H), 7.09 – 7.13 (m, 2H), 6.96 (s, 1H), 5.72 (s, 1H), 2.44 (s, 3H), 2.39 (s, 3H). **^{13}C NMR** (126 MHz, $CDCl_3$) δ 159.4 (d, *J* = 245.2 Hz), 148.6, 145.6, 143.3, 136.7, 133.7, 131.9, 129.9, 128.4, 127.4, 127.3, 125.2 (d, *J* = 8.2 Hz), 123.1 (q, *J* = 287.3 Hz), 120.2, 119.5, 118.3 (d, *J* = 23.6 Hz), 117.7, 116.0 (d, *J* = 8.2 Hz), 113.5 (d, *J* = 25.4 Hz), 69.5, 64.5 (q, *J* = 26.9 Hz), 21.7, 21.1. **^{19}F NMR** (282 MHz, $CDCl_3$) δ −74.9 (s, 3F), −116.7 – −116.8 (s, 1F). **IR (KBr)**: 3135, 2929, 1598, 1471, 1403, 1278, 1186, 1116, 1089, 950, 840, 759 cm^{-1}. **HRMS (ESI)** calculated for $C_{25}H_{18}F_4N_2O_5NaS_2$ $[M+Na]^+$: 589.0491, found: 589.0504.

(13b*S*,13c*R*)-12-Chloro-9-tosyl-13b-(trifluoromethyl)-13b,13c-dihydro-9*H*-benzo[5',6'][1,2,3]oxathiazino[3',4':1,5]pyrrolo[3,4-*b*]indole 6,6-dioxide (3ea):

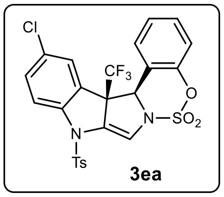

Following the general method **D**, compound **3ea** was obtained as a yellow solid (48.1 mg, Yield: 84%), m.p. = 103.9 – 104.9 °C. The enantiomeric excess (99% *ee*) was determined by chiral HPLC using CHIRALPAK® IC (n-hexane/isopropanol = 85.0/15.0, flow rate 1.0 mL/min, λ = 254 nm) t (major) = 11.867 min, t (minor) = 28.683 min). $[\alpha]^{25}_{D}$ = −123.7 (c = 0.4, $CHCl_3$, 99% *ee*). **^{1}H NMR** (500 MHz, $CDCl_3$) δ 7.73 – 7.78 (m, 3H), 7.41 – 7.50 (m, 4H), 7.36 (d, *J* = 2.1 Hz, 1H), 7.24 – 7.29 (m, 3H), 6.98 (s, 1H), 5.79 (s, 1H), 2.39 (s, 3H). **^{13}C NMR** (126 MHz, $CDCl_3$) δ 150.7, 145.8, 145.7, 133.7, 131.6, 131.3, 130.0, 128.3, 127.3, 127.1, 126.7, 126.1, 125.2, 123.0 (q, *J* = 287.3 Hz), 120.4, 119.4, 118.1, 115.8, 69.5, 64.5 (q, *J* = 26.9 Hz), 21.7. **^{19}F NMR** (282 MHz, $CDCl_3$) δ −75.0 (s, 3F). **IR (KBr)**: 3127, 2929, 1598, 1457, 1403, 1278, 1170, 904, 786, 663, 617, 588 cm^{-1}. **HRMS (ESI)** calculated for $C_{24}H_{16}ClF_3N_2O_5NaS_2$ $[M+Na]^+$: 591.0039, found: 591.0042.

(13b*S*,13c*R*)-12-Chloro-2-methoxy-9-tosyl-13b-(trifluoromethyl)-13b,13c-dihydro-9*H*-benzo[5',6'][1,2,3]oxathiazino[3',4':1,5]pyrrolo[3,4-*b*]indole 6,6-dioxide (3ec):

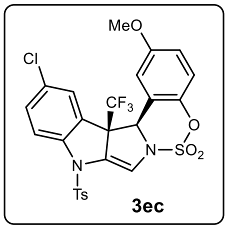

Following the general method **D**, compound **3ec** was obtained as a yellow solid (51.7 mg, Yield: 86%), m.p. = 103.8 – 104.8 °C. The enantiomeric excess (99% *ee*) was determined by chiral HPLC using CHIRALPAK® IC (n-hexane/isopropanol = 90.0/10.0, flow rate 1.0 mL/min, λ = 254 nm) t (major) = 20.258 min, t (minor) = 60.733 min). $[\alpha]^{25}_{D}$ = −170.9 (c = 0.5, $CHCl_3$, 99% *ee*). **^{1}H NMR** (500 MHz, $CDCl_3$) δ 7.72 – 7.77 (m, 3H), 7.44 – 7.46 (m, 1H), 7.34 (d, *J* = 1.8 Hz, 1H), 7.26 – 7.29 (m, 2H), 7.17 – 7.19 (m, 1H), 7.02 – 7.00 (m, 1H), 7.00 – 6.97 (m, 1H), 6.90 (s, 1H), 5.70 (s, 1H), 3.87 (s, 3H), 2.39 (s, 3H). **^{13}C NMR** (126 MHz, $CDCl_3$) δ 157.5, 145.8, 145.7, 144.2, 133.7, 131.6, 130.0, 127.3, 127.1, 126.0, 125.3, 123.0 (q, *J* = 287.3 Hz), 121.3, 119.5, 119.0, 115.8, 115.4, 114.0, 69.5, 64.5 (q, *J* = 26.9 Hz), 55.9, 21.7. **^{19}F NMR** (282 MHz, $CDCl_3$) δ −74.9 (s, 3F). **IR (KBr)**: 3131, 2968, 1595, 1496, 1461, 1278, 1170, 840. 728, 663, 590 cm^{-1}. **HRMS (ESI)** calculated for $C_{25}H_{18}ClF_3N_2O_6NaS_2$ $[M+Na]^+$: 621.0145, found: 621.0142.

(13b*S*,13c*R*)-12-Methyl-9-tosyl-13b-(trifluoromethyl)-13b,13c-dihydro-9*H*-benzo[5',6'] [1,2,3]oxathiazino[3',4':1,5]pyrrolo[3,4-*b*]indole 6,6-dioxide (3fa):

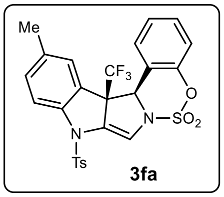

Following the general method **D**, compound **3fa** was obtained as a yellow solid (42.3 mg, Yield: 77%), m.p. = 116.7 – 117.4 °C. The enantiomeric excess (98% *ee*) was determined by chiral HPLC using CHIRALPAK® IC (n-hexane/isopropanol = 85.0/15.0, flow rate 1.0 mL/min, λ = 254 nm) t (major) = 15.333 min, t (minor) = 43.892 min). $[\alpha]^{25}_{D}$ = −133.4 (c = 0.4, $CHCl_3$, 98% *ee*). **^{1}H NMR** (500 MHz, $CDCl_3$) δ 7.78 (d, *J* = 8.2 Hz, 2H), 7.68 (d, *J* = 8.2 Hz, 1H), 7.48 – 7.50 (m, 2H), 7.40 – 7.42 (m, 1H), 7.24 – 7.29 (m, 4H), 7.20 (s, 1H), 6.94 (s, 1H), 5.76 (s, 1H), 2.39 (s, 3H), 2.38 (s, 3H). **^{13}C NMR** (126 MHz, $CDCl_3$) δ 150.7, 145.3, 144.8, 134.7, 133.9, 132.1, 131.1, 129.8, 128.4,

127.9, 127.3, 126.4, 126.3, 123.7, 123.3 (q, J = 287.3 Hz), 120.3, 118.5, 118.4, 114.6, 69.7, 64.7 (q, J = 26.6 Hz), 21.7, 21.1. **^{19}F NMR** (282 MHz, $CDCl_3$) δ −74.9 (s, 3F). **IR (KBr)**: 3131, 2929, 1598, 1477, 1402, 1170, 906, 759, 665, 566 cm^{-1}. **HRMS (ESI)** calculated for $C_{25}H_{19}F_3N_2O_5NaS_2$ $[M+Na]^+$: 571.0585, found: 571.0572.

5. Synthetic utility I:

5.1. Synthesis of (13b*S*,13c*R*)-2-Phenyl-9-tosyl-13b-(trifluoromethyl)-13b,13c-dihydro-9*H*-benzo[5',6'] [1,2,3]oxathiazino[3',4':1,5]pyrrolo[3,4-*b*]indole 6,6-dioxide (6):

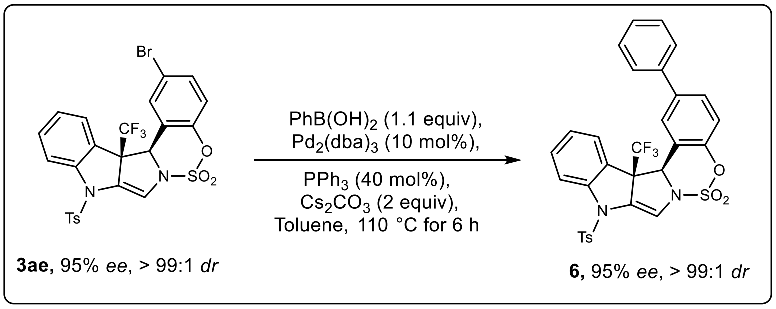

In a flame dried Schlenk tube 1 mL Toluene was taken. The solvent was degassed by using standard "freeze-pump-thaw" method. The process was repeated for three times and finally the Schlenk tube was filled with argon gas. After that, $Pd_2(dba)_3$ (10 mol%, 9.2 mg) and triphenylphosphine (40 mol%, 10.5 mg) were taken in the tube inside the glove box and allowed to stir for 5 min. To the above mixture, bromo-indole **3ae** (0.1 mmol, 1.0 equiv, 61 mg), phenylboronic acid (0.11 mmol, 1.1 equiv, 13.4 mg) and Cs_2CO_3 (0.2 mmol, 2.0 equiv, 65 mg) were added and removed from the glove box. The mixture was allowed to stir at 110 °C for 6 h under Ar. After completion, the reaction was quenched by saturated NH_4Cl aqueous solution (5 mL) at room temperature and extracted with CH_2Cl_2 (3×10 mL). The combined organic layers were washed with water and brine, then dried over Na_2SO_4, filtrated, and concentrated under vacuum. The residue was purified by silica gel column chromatography (PE/EtOAc = 9/1) to afford the desired product **6** (48 mg, yield: 79%) as white solid. m.p. = 128.0 – 128.9 °C. The enantiomeric excess (95% *ee*) was determined by chiral HPLC using CHIRALPAK® IC (n-hexane/isopropanol = 85.0/15.0, flow rate 1.0 mL/min, λ = 254 nm) t (major) = 19.425 min, t (minor) = 41.225 min). $[\alpha]^{25}_D$ = −198.1 (c = 0.6, $CHCl_3$, 95% *ee*). **^{1}H NMR** (500 MHz, $CDCl_3$) δ 7.79 – 7.81 (m, 3H), 7.62 – 7.66 (m, 2H), 7.40 – 7.56 (m, 7H), 7.26 – 7.31 (m, 3H), 7.19 – 7.19 (m, 1H), 6.98 (s, 1H), 5.84 (s, 1H), 2.38 (s, 3H). **^{13}C NMR** (126 MHz, $CDCl_3$) δ 150.0, 147.1,

145.5, 140.0, 139.2, 134.0, 131.2, 130.0, 129.9, 129.2, 128.2, 127.7, 127.3, 127.1, 127.0, 125.8, 124.8, 123.6, 123.4 (q, *J* = 287.3 Hz), 120.6, 118.7, 118.6, 114.8, 69.9, 64.8 (q, *J* = 26.6 Hz), 21.7. **^{19}F NMR** (282 MHz, $CDCl_3$) δ −74.8 (s, 3F). **IR (KBr)**: 3139, 3072, 1600, 1481, 1396, 1172, 1091, 950, 842, 665, 572 cm^{-1}. **HRMS (ESI)** calculated for $C_{30}H_{21}F_3N_2O_5NaS_2$ $[M+Na]^+$: 633.0742, found: 633.0733.

5.2. General Procedure for the detosylative-methoxylation reaction (Method E):

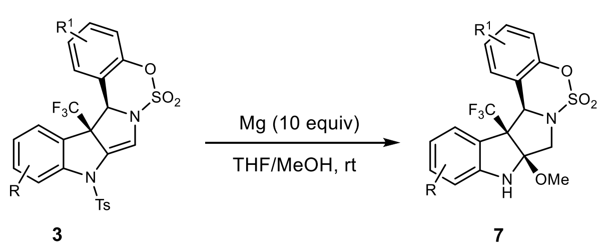

Under argon atmosphere, a flame-dried Schlenk tube was charged with compound **3** (0.1 mmol, 1 equiv), Mg powder (1.0 mmol, 10 equiv), THF (0.3 mL) and MeOH (0.7 mL). the resulting solution was left for 6 h at room temperature under sonication. The mixture was filtered through a celite pad and concentrated under reduced pressure and purified by flash column chromatography (using Toluene) to obtain the pure product **7**.

(13b*S*,13c*R*)-8a-Methoxy-13b-(trifluoromethyl)-8a,9,13b,13c-tetrahydro-8*H*-benzo[5',6'][1,2,3]oxathiazino[3',4':1,5]pyrrolo[3,4-*b*]indole 6,6-dioxide (7aa):

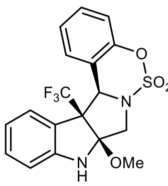

Following the general method **E**, compound **7aa** was obtained as a white solid (32.2 mg, Yield: 78%), m.p. = 116.5 – 117.4 °C. The enantiomeric excess (99% *ee*) was determined by chiral HPLC using CHIRALPAK® OD-3 (n-hexane/isopropanol = 95.0/5.0, flow rate 1.0 mL/min, λ = 254 nm) t (minor) = 18.717 min, t (major) = 29.975 min). $[\alpha]^{25}_D$ = +78.0 (c = 0.5, $CHCl_3$, 99% *ee*). **^{1}H NMR** (500 MHz, $CDCl_3$) δ 7.39 – 7.42 (m, 1H), 7.35 (d, *J* = 7.6 Hz, 1H), 7.28 – 7.31 (m, 1H), 7.20 – 7.23 (m, 1H), 7.11 – 7.15 (m, 2H), 6.91 – 6.94 (m, 1H), 6.83 (d, *J* = 7.9 Hz, 1H), 5.01 (s, 1H), 4.96 (s, 1H), 4.19 (d, *J* = 10.1 Hz, 1H), 4.01 (d, *J* = 10.1 Hz, 1H), 3.19 (s, 3H). **^{13}C NMR** (126 MHz, $CDCl_3$) δ 150.7, 147.0, 130.8, 128.4, 125.6, 131.1, 129.8, 128.4, 125.6, 124.5 (q, *J* = 281.5 Hz),

123.6, 120.1, 109.7, 103.6, 71.4, 65.1 (q, *J* = 25.7 Hz), 57.1, 51.8. **^{19}F NMR** (282 MHz, $CDCl_3$) δ −63.8 (s, 3F). **IR (KBr)**: 3411, 3365, 2956, 1612, 1469, 1382, 1174, 964, 881, 752, 539 cm^{-1}. **HRMS (ESI)** calculated for $C_{18}H_{14}F_3N_2O_4S$ $[M-H]^-$: 411.0626, found: 411.0628.

(8a*S*,13b*S*,13c*R*)-8a-methoxy-2-methyl-13b-(trifluoromethyl)-8a,9,13b,13c-tetrahydro-8*H*-benzo[5',6'][1,2,3]oxathiazino[3',4':1,5]pyrrolo[3,4-b]indole 6,6-dioxide (7ab):

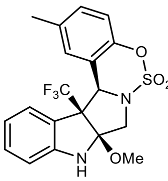

7ab

Following the general method **E**, compound **7ab** was obtained as a white solid (32 mg, Yield: 75%), m.p. = 125.1 – 126.3 °C. The enantiomeric excess (99% *ee)* was determined by chiral HPLC using CHIRALPAK® OD-3 (n-hexane/isopropanol = 95.0/5.0, flow rate 1.0 mL/min, λ = 254 nm) t (minor) = 16.933 min, t (major) = 38.558 min). $[\alpha]^{25}_{D}$ = +74.9 (c = 0.2, $CHCl_3$, 99% *ee*). **^{1}H NMR** (500 MHz, $CDCl_3$) δ 7.29 – 7.35 (m, 2H), 7.19 (dd, *J* = 8.4, 1.7 Hz, 1H), 7.03 (d, *J* = 8.5 Hz, 1H), 6.93 – 6.96 (m, 1H), 6.90 (s, 1H), 6.85 (d, *J* = 7.9 Hz, 1H), 4.96 (s, 1H), 4.89 (s, 1H), 4.18 (d, *J* = 10.1 Hz, 1H), 4.01 (d, *J* = 10.1 Hz, 1H), 3.19 (s, 3H), 2.33 (s, 3H). **^{13}C NMR** (126 MHz, $CDCl_3$) δ 148.6, 147.0, 135.4, 131.0, 130.7, 128.7, 124.5 (q, *J* = 281.5 Hz), 123.8, 123.6, 120.1, 119.5, 118.4, 109.7, 103.6, 71.5, 65.1 (q, *J* = 25.7 Hz), 57.1, 51.8, 20.9. **^{19}F NMR** (282 MHz, $CDCl_3$) δ −63.74 (s, 3F). **IR (KBr)**: 3355, 2960, 1612, 1496, 1267, 1176, 1141, 1116, 839, 821 cm^{-1}. **HRMS (ESI)** calculated for $C_{19}H_{16}F_3N_2O_4S$ $[M-H]^-$: 425.0783, found: 425.0779.

(8a*S*,13b*S*,13c*R*)-2,8a-dimethoxy-13b-(trifluoromethyl)-8a,9,13b,13c-tetrahydro-8*H*-benzo [5',6'][1,2,3]oxathiazino[3',4':1,5]pyrrolo[3,4-b]indole 6,6-dioxide (7ac):

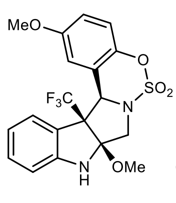

7ac

Following the general method **E**, compound **7ac** was obtained as a white solid (36.3 mg, Yield: 82%), m.p. = 128.6 – 129.4 °C. The enantiomeric excess (99% *ee*) was determined by chiral HPLC

using CHIRALPAK® IA (n-hexane/isopropanol = 95.0/5.0, flow rate 1.0 mL/min, λ = 254 nm) t (minor) = 18.767 min, t (major) = 35.225 min). $[\alpha]^{25}_{D}$ = +86.6 (c = 0.2, $CHCl_3$, 99% *ee*). **^{1}H NMR** (500 MHz, $CDCl_3$) δ 7.37 (d, *J* = 7.6 Hz, 1H), 7.28 – 7.32 (m, 1H), 7.08 (d, *J* = 8.9 Hz, 1H), 6.91 – 6.94 (m, 2H), 6.84 (d, *J* = 7.6 Hz, 1H), 6.60 (d, *J* = 2.7 Hz, 1H), 4.94 (s, 2H), 4.18 (d, *J* = 10.1 Hz, 1H), 4.01 (d, *J* = 10.1 Hz, 1H), 3.77 (s, 3H), 3.19 (s, 3H). **^{13}C NMR** (126 MHz, $CDCl_3$) δ 156.8, 147.0, 144.5, 130.8, 124.4 (q, *J* = 281.8 Hz), 123.6, 123.5, 120.7, 120.1, 119.6, 115.7, 113.2, 109.7, 103.6, 71.5, 65.1 (q, *J* = 25.7 Hz), 57.2, 55.7, 51.8. **^{19}F NMR** (282 MHz, $CDCl_3$) δ −63.70 (s, 3F). **IR (KBr)**: 3442, 2956, 1589, 1494, 1455, 1261, 1211, 1170, 860, 757 cm^{-1}. **HRMS (ESI)** calculated for $C_{19}H_{16}F_3N_2O_5S$ $[M-H]^-$: 441.0732, found: 441.0722.

(8a*S*,13b*S*,13c*R*)-2-fluoro-8a-methoxy-13b-(trifluoromethyl)-8a,9,13b,13c-tetrahydro-8*H*-benzo[5',6'][1,2,3]oxathiazino[3',4':1,5]pyrrolo[3,4-b]indole 6,6-dioxide (7ad):

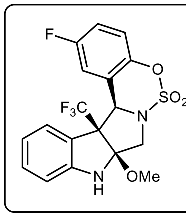

Following the general method **E**, compound **7ad** was obtained as a white solid (27.1 mg, Yield: 63%), m.p. = 134.1 – 135.1 °C. The enantiomeric excess (85% *ee*) was determined by chiral HPLC using CHIRALPAK® OD-3 (n-hexane/isopropanol = 95.0/5.0, flow rate 1.0 mL/min, λ = 230 nm) t (minor) = 18.117 min, t (major) = 34.392 min). $[\alpha]^{25}_{D}$ = +82.2 (c = 0.2, $CHCl_3$, 85% *ee*). **^{1}H NMR** (500 MHz, $CDCl_3$) δ 7.37 (d, *J* = 7.6 Hz, 1H), 7.30 – 7.34 (m, 1H), 7.11 – 7.14 (m, 2H), 6.94 – 6.97 (m, 1H), 6.83 – 6.86 (m, 2H), 4.96 (s, 1H), 4.90 (s, 1H), 4.17 (d, *J* = 10.1 Hz, 1H), 4.02 (d, *J* = 10.1 Hz, 1H), 3.20 (s, 3H). **^{13}C NMR** (126 MHz, $CDCl_3$) δ 159.4 (d, *J* = 246.1 Hz), 146.9, 146.8 (d, *J* = 1.8 Hz), 131.0, 124.4 (q, *J* = 281.5 Hz), 123.5, 123.3, 121.5 (d, *J* = 8.2 Hz), 120.5 (d, *J* = 8.2 Hz), 120.4, 117.5 (d, *J* = 23.6 Hz), 115.0 (d, *J* = 25.4 Hz), 109.7, 103.5, 71.1, 65.1 (q, *J* = 25.7 Hz), 57.2, 51.8. **^{19}F NMR** (282 MHz, $CDCl_3$) δ −63.69 (s, 3F), −115.69 – −115.74 (m, 1F). **IR (KBr)**: 3403, 3054, 2956, 2848, 1612, 1488, 1172, 1105, 829, 750 cm^{-1}. **HRMS (ESI)** calculated for $C_{18}H_{13}F_4N_2O_4S$ $[M-H]^-$: 429.0532, found: 429.0528.

(8a*S*,13b*S*,13c*R*)-8a-methoxy-3-methyl-13b-(trifluoromethyl)-8a,9,13b,13c-tetrahydro-8*H*-benzo[5',6'][1,2,3]oxathiazino[3',4':1,5]pyrrolo[3,4-b]indole 6,6-dioxide (7ag):

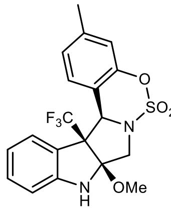

7ag

Following the general method **E**, compound **7ag** was obtained as a white solid (28.6 mg, Yield: 67%), m.p. = 124.4 – 125.2 °C. The enantiomeric excess (99% *ee*) was determined by chiral HPLC using CHIRALPAK® OD-3 (n-hexane/isopropanol = 95.0/5.0, flow rate 1.0 mL/min, λ = 230 nm) t (minor) = 18.892 min, t (major) = 27.700 min). $[\alpha]^{25}_{D}$ = +93.1 (c = 0.1, $CHCl_3$, 99% *ee*). **^{1}H NMR** (500 MHz, $CDCl_3$) δ 7.34 (d, *J* = 7.6 Hz, 1H), 7.28 – 7.32 (m, 1H), 6.98 – 7.01 (m, 2H), 6.91 – 6.95 (m, 2H), 6.84 (d, *J* = 7.9 Hz, 1H), 4.98 (s, 1H), 4.87 (s, 1H), 4.18 (d, *J* = 10.1 Hz, 1H), 4.01 (d, *J* = 10.1 Hz, 1H), 3.20 (s, 3H), 2.38 (s, 3H). **^{13}C NMR** (126 MHz, $CDCl_3$) δ 150.5, 147.0, 141.2, 130.7, 128.1, 126.5, 124.5 (q, *J* = 281.8 Hz), 123.8, 123.7, 120.2, 120.1, 115.6, 109.6, 103.6, 71.4, 65.1 (q, *J* = 25.4 Hz), 57.2, 51.8, 21.2. **^{19}F NMR** (282 MHz, $CDCl_3$) δ −63.71 (s, 3F). **IR (KBr)**: 3384, 3045, 2954, 2850, 1612, 1508, 1257, 1189, 1170, 844 cm^{-1}. **HRMS (ESI)** calculated for $C_{19}H_{16}F_3N_2O_4S$ $[M-H]^-$: 425.0783, found: 425.0789.

(8a*S*,13b*S*,13c*R*)-3,8a-dimethoxy-13b-(trifluoromethyl)-8a,9,13b,13c-tetrahydro-8*H*-benzo [5',6'][1,2,3]oxathiazino[3',4':1,5]pyrrolo[3,4-b]indole 6,6-dioxide (7ah):

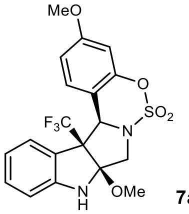

7ah

Following the general method **E**, compound **7ah** was obtained as a white solid (35.8 mg, Yield: 81%), m.p. = 113.9 – 114.6 °C. The enantiomeric excess (99% *ee*) was determined by chiral HPLC using CHIRALPAK® OD-3 (n-hexane/isopropanol = 95.0/5.0, flow rate 1.0 mL/min, λ = 230 nm) t (minor) = 24.617 min, t (major) = 38.833 min). $[\alpha]^{25}_{D}$ = +105.9 (c = 0.2, $CHCl_3$, 99% *ee*). **^{1}H NMR** (500 MHz, $CDCl_3$) δ 7.33 (d, *J* = 7.3 Hz, 1H), 7.28 – 7.31 (m, 1H), 7.00 (d, *J* = 8.5 Hz, 1H), 6.90 – 6.93 (m, 1H), 6.83 (d, *J* = 7.9 Hz, 1H), 6.77 (dd, *J* = 8.7, 2.6 Hz, 1H), 6.65 (d, *J* = 2.4 Hz, 1H), 4.96 (s, 1H), 4.89 (s, 1H), 4.18 (d, *J* = 10.1 Hz, 1H), 4.00 (d, *J* = 10.1 Hz, 1H), 3.82 (s, 3H), 3.19 (s, 3H). **^{13}C NMR** (126 MHz, $CDCl_3$) δ 161.0, 151.5, 146.9, 130.7, 129.1, 124.5 (q, *J* = 281.8

Hz), 123.8, 123.6, 120.1, 112.6, 110.3, 109.6, 104.6, 103.5, 71.3, 64.9 (q, *J* = 25.7 Hz), 57.2, 55.6, 51.7. **^{19}F NMR** (282 MHz, $CDCl_3$) δ −63.84 (s, 3F). **IR (KBr)**: 3334, 2958, 1616, 1583, 1510, 1284, 1168, 1141, 819, 750 cm^{-1}. **HRMS (ESI)** calculated for $C_{19}H_{16}F_3N_2O_5S$ $[M-H]^-$: 441.0732, found: 441.0728.

(8a*S*,13b*S*,13c*R*)-12-fluoro-8a-methoxy-2-methyl-13b-(trifluoromethyl)-8a,9,13b,13c-tetrahydro-8*H*-benzo[5',6'][1,2,3]oxathiazino[3',4':1,5]pyrrolo[3,4-b]indole 6,6-dioxide (7db):

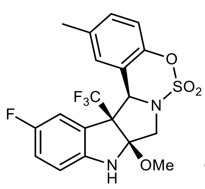

7db

Following the general method **E**, compound **7db** was obtained as a white solid (26.7 mg, Yield: 60%), m.p. = 125.1 – 126.3 °C. The enantiomeric excess (99% *ee*) was determined by chiral HPLC using CHIRALPAK® IA (n-hexane/isopropanol = 90.0/10.0, flow rate 1.0 mL/min, λ = 254 nm) t (minor) = 7.767 min, t (major) = 13.900 min). $[\alpha]^{25}_{D}$ = +68.1 (c = 0.3, $CHCl_3$, 99% *ee*). **^{1}H NMR** (500 MHz, $CDCl_3$) δ 7.20 – 7.22 (m, 1H), 7.08 (dd, *J* = 7.9, 1.8 Hz, 1H), 7.00 – 7.05 (m, 2H), 6.88 (s, 1H), 6.76 (dd, *J* = 8.5, 4.0 Hz, 1H), 4.97 (s, 1H), 4.88 (s, 1H), 4.17 (d, *J* = 10.1 Hz, 1H), 3.99 (d, *J* = 10.1 Hz, 1H), 3.20 (s, 3H), 2.34 (s, 3H). **^{13}C NMR** (126 MHz, $CDCl_3$) δ 157.0 (d, *J* = 237.9 Hz), 148.6, 143.2, 135.7, 131.3, 128.6, 124.9 (d, *J* = 8.2 Hz), 124.2 (q, *J* = 281.8 Hz), 119.7, 118.1, 117.2 (d, *J* = 23.6 Hz), 111.5 (d, *J* = 25.4 Hz), 110.3 (d, *J* = 8.2 Hz), 104.3, 71.4, 65.3 (q, *J* = 26.0 Hz), 57.1, 51.8, 21.0. **^{19}F NMR** (282 MHz, $CDCl_3$) δ −63.81 (s, 3F), −123.46 – −123.53 (m, 1F). **IR (KBr)**: 3384, 3363, 3033, 2952, 1608, 1488, 1268, 1180, 838, 821 cm^{-1}. **HRMS (ESI)** calculated for $C_{19}H_{15}F_4N_2O_4S$ $[M-H]^-$: 443.0689, found: 443.0686.

(8a*S*,13b*S*,13c*R*)-12-chloro-8a-methoxy-13b-(trifluoromethyl)-8a,9,13b,13c-tetrahydro-8*H*-benzo[5',6'][1,2,3]oxathiazino[3',4':1,5]pyrrolo[3,4-b]indole 6,6-dioxide (7ea):

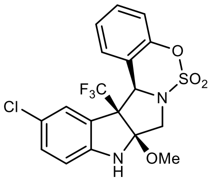

7ea

Following the general method **E**, compound **7ea** was obtained as a white solid (33.5 mg, Yield: 61%), m.p. = 110.2 – 111.2 °C. The enantiomeric excess (99% *ee*) was determined by chiral HPLC using CHIRALPAK® OD-3 (n-hexane/isopropanol = 97.0/3.0, flow rate 0.8 mL/min, λ = 254 nm) t (minor) = 42.958 min, t (major) = 87.358 min). $[\alpha]^{25}_{D}$ = +111.1 (c = 0.2, $CHCl_3$, 99% *ee*). **^{1}H NMR** (500 MHz, $CDCl_3$) δ 7.41 – 7.44 (m, 1H), 7.32 (s, 1H), 7.29 (dd, *J* = 8.4, 2.0 Hz, 1H), 7.25 – 7.26 (m, 1H), 7.15 (dd, *J* = 8.2, 0.9 Hz, 1H), 7.11 (d, *J* = 7.6 Hz, 1H), 6.79 (d, *J* = 8.2 Hz, 1H), 5.02 (s, 1H), 4.92 (s, 1H), 4.19 (d, *J* = 10.1 Hz, 1H), 4.02 (d, *J* = 10.1 Hz, 1H), 3.21 (s, 3H). **^{13}C NMR** (126 MHz, $CDCl_3$) δ 150.7, 145.6, 130.8, 130.6, 128.3, 125.8, 125.3, 124.9, 124.1 (q, *J* = 281.8 Hz), 124.0, 120.0, 118.5, 110.6, 104.0, 71.4, 65.1 (q, *J* = 26.0 Hz), 57.1, 51.9. **^{19}F NMR** (282 MHz, $CDCl_3$) δ −63.70 (s, 3F). **IR (KBr)**: 3419, 2996, 2842, 1612, 1577, 1261, 1178, 1147, 1118, 815, 754 cm^{-1}. **HRMS (ESI)** calculated for $C_{18}H_{13}F_3N_2O_4SCl$ $[M{-}H]^-$: 445.0237, found: 445.0229.

6. Synthetic utility II:

6.1. Synthesis of (8a*R*,13b*S*,13c*R*)-8a-allyl-13b-(trifluoromethyl)-8a,9,13b,13c-tetrahydro-8*H*-benzo[5',6'][1,2,3]oxathiazino[3',4':1,5]pyrrolo[3,4-b]indole (8):

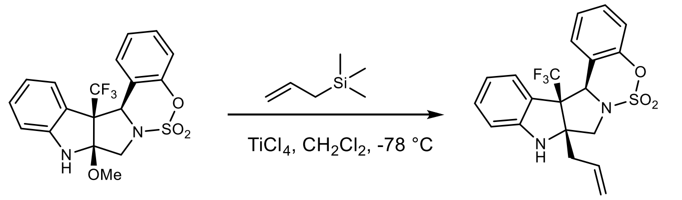

7aa, 99% *ee*, > 99:1 *dr* **8**, 99% *ee*, > 99:1 *dr*

Under argon atmosphere, a flame-dried Schlenk tube was charged with 2-methoxy indoline **7aa** (0.1 mmol) and DCM (1.0 mL) than cooled to –78 °C. To this solution, $TiCl_4$ (0.1 mmol, 0.1 mL of 1.0 M solution in DCM), and then trimethylallylsilane (0.4 mmol, 63.6 µL) were added slowly. The resulted solution was allowed to stir at room temperature until substrate **7aa** had been consumed as indicated by TLC. the reaction mixture was quenched by adding cool water and extracted with CH_2Cl_2 (3 × 10 mL). The combined organic layers were washed with water and brine, then dried over Na_2SO_4, filtrated, and concentrated under vacuum. The residue was purified by silica gel column chromatography (Hex/EtOAc = 19/1) to afford the desired product **8** (33 mg, yield: 79%) as white solid, m.p. = 53.2 – 54.0 °C. The enantiomeric excess (99% *ee*) was determined by chiral HPLC using CHIRALPAK® IA (n-hexane/isopropanol = 95.0/5.0, flow rate

1.0 mL/min, λ = 254 nm) t (minor) = 14.575 min, t (major) = 46.192 min). $[\alpha]^{25}_D$ = +123.5 (c = 0.4, $CHCl_3$, 99% *ee*). **^{1}H NMR** (500 MHz, $CDCl_3$) δ 7.38 – 7.41 (m, 1H), 7.33 (d, *J* = 7.6 Hz, 1H), 7.25 – 7.29 (m, 1H), 7.20 – 7.23 (m, 1H), 7.11 – 7.15 (m, 2H), 6.90 – 6.93 (m, 1H), 6.79 (d, *J* = 7.9 Hz, 1H), 5.73 – 5.81 (m, 1H), 5.35 (dd, *J* = 10.1, 0.6 Hz, 1H), 5.20 – 5.25 (m, 2H), 4.58 (s, 1H), 4.08 (d, *J* = 10.4 Hz, 1H), 3.90 (d, J = 10.4 Hz, 1H), 2.44 – 2.55 (m, 2H). **^{13}C NMR** (126 MHz, $CDCl_3$) δ 150.7, 147.8, 131.1, 130.6, 130.3, 128.6, 125.6, 125.1 (q, *J* = 282.7 Hz), 124.2, 123.8, 122.3, 120.0, 119.8, 119.1, 111.3, 70.3, 67.7 (q, *J* = 25.4 Hz), 58.5, 38.6, 38.5. **^{19}F NMR** (282 MHz, $CDCl_3$) δ −61.74 (s, 3F). **IR (KBr)**: 3370, 3079, 2923, 1612, 1484, 1469, 1166, 1105, 838, 752 cm^{-1}. **HRMS (ESI)** calculated for $C_{20}H_{16}F_3N_2O_3S$ $[M-H]^-$: 421.0834, found: 421.0834.

6.2. Synthesis of (8a*R*,13b*S*,13c*R*)-13b-(trifluoromethyl)-13b,13c-dihydro-8*H*-benzo[5',6'][1,2,3]oxathiazino [3',4':1,5]pyrrolo[3,4-b]indole-8a(9*H*)-carbonitrile 6,6-dioxide (9):

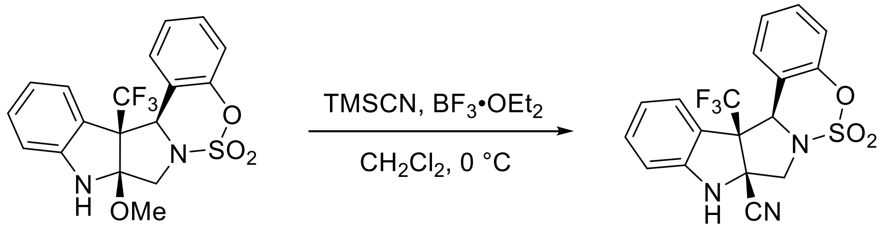

7aa, 99% *ee*, > 99:1 *dr* **9**, 99% *ee*, > 99:1 *dr*

Under argon atmosphere, a flame-dried Schlenk tube was charged with 2-methoxy indoline **7aa** (0.1 mmol) and DCM (1.0 mL) than cooled to 0 °C. To this solution, BF_3•OEt_2 (0.2 mmol, 24.7 μL), and then trimethylsilly cyanide (0.4 mmol, 50 μL) were added slowly. The resulted solution was allowed to stir at room temperature until substrate **7aa** had been consumed as indicated by TLC. the reaction mixture was quenched by adding cool water and extracted with CH_2Cl_2 (3×10 mL). The combined organic layers were washed with water and brine, then dried over Na_2SO_4, filtrated, and concentrated under vacuum. The residue was purified by silica gel column chromatography (Hex/EtOAc = 4/1) to afford the desired product **9** (36 mg, yield: 88%) as white solid, m.p. = 125.1 – 126.3 °C. The enantiomeric excess (99% *ee*) was determined by chiral HPLC using CHIRALPAK® OD-3 (n-hexane/isopropanol = 95.0/5.0, flow rate 1.0 mL/min, λ = 254 nm) t (minor) = 26.525 min, t (major) = 37.450 min). $[\alpha]^{25}_D$ = +112.2 (c = 0.2, $CHCl_3$, 99% *ee*). **^{1}H NMR** (500 MHz, $CDCl_3$) δ 7.44 – 7.48 (m, 1H), 7.37 – 7.40 (m, 2H), 7.26 – 7.30 (m, 1H), 7.19 (dd, *J* = 8.2, 0.9 Hz, 1H), 7.16 (d, *J* = 7.6 Hz, 1H), 7.05 – 7.08 (m, 1H), 6.92 (d, *J* = 7.6 Hz, 1H),

5.22 (s, 1H), 4.85 (s, 1H), 4.45 (d, *J* = 11.0 Hz, 1H), 4.22 (d, *J* = 11.0 Hz, 1H). **^{13}C NMR** (126 MHz, $CDCl_3$) δ 150.6, 146.6, 131.6, 131.1, 128.4, 126.1, 124.1, 123.7 (q, *J* = 280.3 Hz), 122.1, 122.0, 120.2, 117.3, 115.8, 111.9, 69.8 (q, *J* = 26.3 Hz), 69.5, 69.2, 58.1. **^{19}F NMR** (282 MHz, $CDCl_3$) δ −63.57 (s, 3F). **IR (KBr)**: 3378, 3068, 2923, 2852, 1612, 1486, 1469, 1176, 1103, 813 cm^{-1}. **HRMS (ESI)** calculated for $C_{18}H_{11}F_3N_3O_3S$ [M−H]$^-$: 406.0473, found: 406.0447.

6.3. Synthesis of (8a*R*,13b*R*,13c*R*)-13b-(trifluoromethyl)-8a,9,13b,13c-tetrahydro-8*H*-benzo [5',6'][1,2,3] oxathiazino[3',4':1,5]pyrrolo[3,4-b]indole 6,6-dioxide (10):

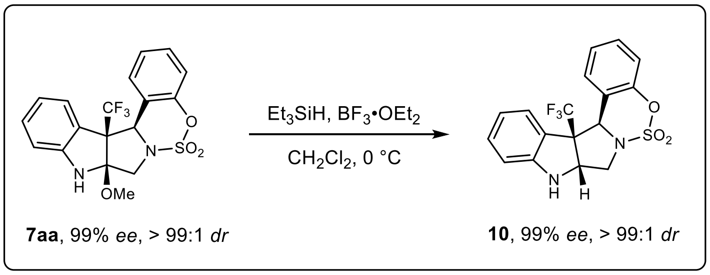

Under argon atmosphere, a flame-dried Schlenk tube was charged with 2-methoxy indoline **7aa** (0.1 mmol) and DCM (1.0 mL) than cooled to 0 °C. To this solution, BF_3•OEt_2 (0.2 mmol, 24.7 μL), and then triethylsilane (0.4 mmol, 64 μL) were added slowly. The resulted solution was allowed to stir at room temperature until substrate **7aa** had been consumed as indicated by TLC. the reaction mixture was quenched by adding cool water and extracted with CH_2Cl_2 (3 × 10 mL). The combined organic layers were washed with water and brine, then dried over Na_2SO_4, filtrated, and concentrated under vacuum. The residue was purified by silica gel column chromatography (Hex/EtOAc = 6/1) to afford the desired product **10** (34.8 mg, yield: 91%) as white solid, m.p. = 125.1 – 126.3 °C. The enantiomeric excess (99% *ee*) was determined by chiral HPLC using CHIRALPAK® OD-3 (n-hexane/isopropanol = 95.0/5.0, flow rate 1.0 mL/min, λ = 254 nm) t (minor) = 25.183 min, t (major) = 29.858 min). $[\alpha]^{25}_D$ = +113.2 (c = 0.2, $CHCl_3$, 99% *ee*). **^{1}H NMR** (500 MHz, $CDCl_3$) δ 7.39 – 7.42 (m, 2H), 7.34 (d, *J* = 7.6 Hz, 1H), 7.25 – 7.30 (m, 2H), 7.13 (dd, *J* = 8.2, 1.2 Hz, 1H), 6.91 – 6.94 (m, 1H), 6.80 (d, *J* = 7.9 Hz, 1H), 5.23 (s, 1H), 4.87 (t, *J* = 4.6 Hz, 1H), 4.36 (d, *J* = 4.0 Hz, 1H), 4.10 (dd, *J* = 10.7, 5.2 Hz, 1H), 3.89 (d, *J* = 10.7 Hz, 1H). **^{13}C NMR** (126 MHz, $CDCl_3$) δ 150.7, 149.7, 130.8, 130.2, 128.4, 125.6, 125.3 (q, *J* = 280.6 Hz), 124.3, 124.2, 120.2, 119.8, 118.4, 110.9, 69.4, 67.0 (q, *J* = 26.3 Hz), 66.4, 55.7. **^{19}F NMR** (282 MHz,

CDCl3) δ −62.45 (s, 1F). **IR (KBr)**: 3388, 3056, 2919, 2854, 1608, 1465, 1176, 1101, 821, 748 cm^{-1}. **HRMS (ESI)** calculated for $C_{17}H_{12}F_3N_2O_3S$ $[M-H]^-$: 381.0521, found: 381.0522.

6.4. Synthesis of (8a*R*,13b*S*,13c*R*)-8a-(1*H*-indol-3-yl)-13b-(trifluoromethyl)-8a,9,13b,13c-tetrahydro-8*H*-benzo[5',6'][1,2,3]oxathiazino[3',4':1,5]pyrrolo[3,4-b]indole 6,6-dioxide (11)[6]:

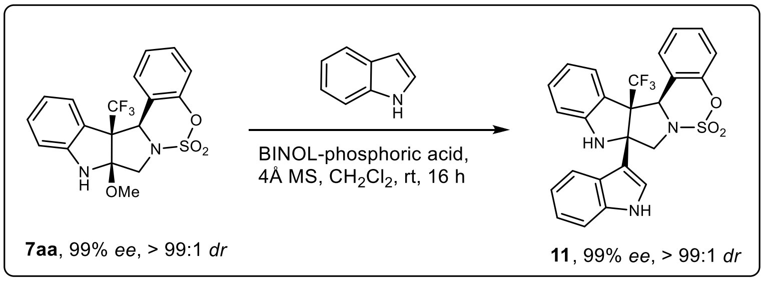

Under argon atmosphere, a flame-dried Schlenk tube was charged with 2-methoxy indoline **7aa** (0.1 mmol), chiral phosphoric acid (0.005 mmol), 4Å MS (5 mg), and DCM (1.0 mL). The solution was stirred at room temperature for 10 min then indole (0.2 mmol) was added in one portion and continued stirring for 16 h. After the reaction was completed (monitored by TLC), the reaction mixture was quenched by adding cool water and extracted with CH_2Cl_2 (3 × 10 mL). The combined organic layers were washed with water and brine, then dried over Na_2SO_4, filtrated, and concentrated under vacuum. The residue was purified by silica gel column chromatography (MeOH/DCM = 1/1) to afford the desired product **11** (48 mg, yield: 82%) as white solid, m.p. = 129.9 – 130.5 °C. The enantiomeric excess (99% *ee*) was determined by chiral HPLC using CHIRALPAK® IA (n-hexane/isopropanol = 90.0/10.0, flow rate 1.0 mL/min, λ = 254 nm) t (minor) = 29.183 min, t (major) = 63.867 min). $[\alpha]^{25}_D$ = +132.1 (c = 0.4, $CHCl_3$, 99% *ee*). **^{1}H NMR** (500 MHz, $CDCl_3$) δ 8.25 (s, 1H), 7.37 – 7.40 (m, 3H), 7.30 (d, *J* = 7.9 Hz, 1H), 7.20 – 7.26 (m, 3H), 7.10 – 7.16 (m, 2H), 6.96 – 6.99 (m, 1H), 6.87 – 6.91 (m, 2H), 6.82 (d, *J* = 8.2 Hz, 1H), 5.39 (s, 1H), 4.95 (d, *J* = 10.4 Hz, 1H), 4.60 (s, 1H), 4.20 (d, *J* = 10.4 Hz, 1H). **^{13}C NMR** (126 MHz, $CDCl_3$) δ 150.7, 149.4, 136.8, 130.8, 130.2, 128.9, 125.6, 125.1, 124.8, 124.6 (q, *J* = 282.4 Hz), 124.1, 123.7, 122.5, 121.2, 120.4, 119.9, 119.7, 119.2, 113.5, 111.4, 110.4, 77.9, 72.1, 69.0 (q, *J* = 24.8 Hz), 59.6. **^{19}F NMR** (282 MHz, $CDCl_3$) δ −62.45 (s, 3F). **IR (KBr)**: 3405, 3060, 2960, 1608, 1482, 1465, 1268, 1178, 1103, 835 cm^{-1}. **HRMS (ESI)** calculated for $C_{25}H_{17}F_3N_3O_3S$ $[M-H]^-$: 496.0943, found: 496.0941.

7. General Procedure for Cu-catalyzed decarboxylative annulation reaction of Me-benzoxazinanone (Method F):

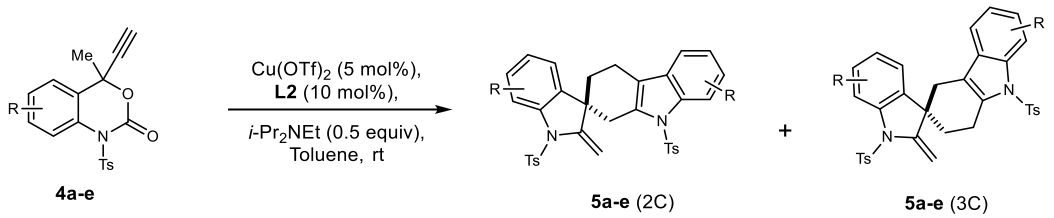

In an argon filled glove box, a flame-dried 10 mL Schlenk tube was charged with copper(II) trifluoromethanesulfonate (1.81 mg, 0.005 mmol, 5 mol%), 2,6-bis[(4*R*)-phenyl-2-oxazolin-2-yl]-pyridine **L2** (3.69 mg, 0.01 mmol, 10 mol%) and anhydrous Toluene (1 mL). The resulting solution was stirred for 1 h at 80 °C. In an argon filled glove box, Me-benzoxazinanone **4** (0.1 mmol) and DIPEA (8.7 μL, 0.05 mmol, 0.5 equiv) were added. The resulting solution was stirred at room temperature until complete conversion of Me-benzoxazinanone **4** (monitored by TLC). The reaction was quenched by saturated NH_4Cl aqueous solution (2 mL). The resulting solution was extracted with ethyl acetate (5 mL × 3). The combined organic layers were dried over Na_2SO_4, filtered and concentrated in *vacuo*. The diastereomeric ratio and crude yield were determined by ^{19}F NMR analysis of the crude reaction mixture. The residue was purified by flash silica gel chromatography (Hexane/EtOAc) to afford the title compound **5**. The characterization data of **5** are summarized below.

2'-Methylene-1',9-ditosyl-1,3,4,9-tetrahydrospiro[carbazole-2,3'-indoline] [5a (2C)]

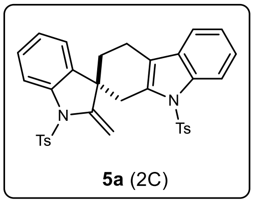

Following the general method **F**, compound **5a** (2C) was obtained as a light yellow solid (21.9 mg, Yield: 71%), m.p. = 156.2 – 157.2 °C. **^{1}H NMR** (500 MHz, $CDCl_3$) δ 8.19 (d, *J* = 8.2 Hz, 1H), 7.93 (d, *J* = 8.2 Hz, 1H), 7.61 (d, *J* = 8.2 Hz, 2H), 7.43 (d, *J* = 8.5 Hz, 2H), 7.26 – 7.36 (m, 4H), 7.22 (d, *J* = 8.2 Hz, 2H), 7.06 (d, *J* = 8.2 Hz, 2H), 6.88 – 6.91 (m, 1H), 6.70 (d, *J* = 7.3 Hz, 1H), 5.67 (d, *J* = 2.1 Hz, 1H), 4.53 (d, *J* = 2.1 Hz, 1H), 3.10 (dd, *J* = 26.6, 18.3 Hz, 2H), 2.44 – 2.53 (m, 2H), 2.36 (s, 3H), 2.31 (s, 3H), 1.30 – 1.34 (m, 1H), 1.10 – 1.13 (m, 1H). **^{13}C NMR** (126 MHz, $CDCl_3$) δ 152.7, 144.8, 144.7, 140.4, 136.6, 136.6, 135.6, 134.1, 133.2, 129.7, 129.4, 129.3, 128.3,

127.2, 126.2, 124.8, 124.5, 123.5, 123.0, 118.2, 118.1, 116.7, 114.7, 97.4, 47.4, 36.7, 33.9, 21.6, 21.5, 17.6. **IR (KBr)**: 3063, 2959, 2921, 1598, 1492, 1474, 1454, 1263, 866, 750 cm^{-1}. **HRMS (ESI)** calculated for $C_{34}H_{30}N_2O_4NaS_2$ $[M+Na]^+$: 617.1545, found: 617.1531.

5',6-Dichloro-2'-methylene-1',9-ditosyl-1,3,4,9-tetrahydrospiro[carbazole-2,3'-indoline] [5b (2C)]:

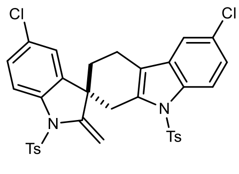

5b (2C)

Following the general method **F**, compound **5b** (2C) was obtained as a light yellow solid (22.9 mg, Yield: 67%), m.p. = 163.1 – 163.8 °C. **^{1}H NMR** (500 MHz, $CDCl_3$) δ 8.13 (d, *J* = 8.9 Hz, 1H), 7.87 (d, *J* = 8.9 Hz, 1H), 7.60 (d, *J* = 8.2 Hz, 2H), 7.38 (d, *J* = 8.5 Hz, 2H), 7.31 – 7.32 (m, 1H), 7.24 – 7.29 (m, 4H), 7.09 (d, *J* = 13.1 Hz, 2H), 6.55 (d, *J* = 2.1 Hz, 1H), 5.69 (d, *J* = 2.4 Hz, 1H), 4.53 (d, *J* = 2.1 Hz, 1H), 3.06 (s, 2H), 2.39 – 2.53 (m, 2H), 2.39 (s, 3H), 2.33 (s, 3H), 1.23 – 1.28 (m, 1H), 1.08 – 1.13 (m, 1H). **^{13}C NMR** (126 MHz, $CDCl_3$) δ 152.2, 145.3, 145.2, 139.1, 138.3, 135.2, 135.0, 134.2, 133.8, 130.4, 130.2, 129.9, 129.5, 129.5, 128.6, 127.2, 126.0, 124.7, 123.1, 118.2, 117.8, 117.4, 115.8, 97.9, 47.4, 36.5, 33.6, 21.7, 21.6, 17.4. **IR (KBr)**: 3060, 2925, 2845, 1650, 1596, 1457, 1291, 909, 805, 704 cm^{-1}. **HRMS (ESI)** calculated for $C_{34}H_{28}N_2O_4NaS_2Cl_2$ $[M+Na]^+$: 685.0765, found: 685.0748.

5,5'-Dibromo-2'-methylene-1',9-ditosyl-1,3,4,9-tetrahydrospiro[carbazole-2,3'-indoline] [5c (2C)]:

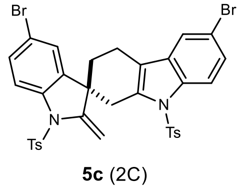

5c (2C)

Following the general method **F**, compound **5c** (2C) was obtained as a light yellow solid (23.2 mg, Yield: 60%), m.p. = 169.1 – 170.0 °C. **^{1}H NMR** (500 MHz, $CDCl_3$) δ 8.07 (d, *J* = 8.9 Hz, 1H), 7.82 (d, *J* = 8.5 Hz, 1H), 7.59 (d, *J* = 7.9 Hz, 2H), 7.48 (s, 1H), 7.39 – 7.43 (m, 4H), 7.24 – 7.26

(m, 2H), 7.11 (d, J = 7.9 Hz, 2H), 6.73 (s, 1H), 5.67 (d, J = 1.8 Hz, 1H), 4.50 (d, J = 1.8 Hz, 1H), 3.08 (s, 2H), 2.43 – 2.54 (m, 2H), 2.39 (s, 3H), 2.34 (s, 3H), 1.24 – 1.29 (m, 1H), 1.03 – 1.08 (m, 1H). **^{13}C NMR** (126 MHz, $CDCl_3$) δ 152.0, 145.3, 145.2, 139.6, 138.7, 135.3, 135.2, 134.0, 133.8, 131.5, 130.9, 130.0, 129.5, 127.4, 127.2, 126.0, 125.9, 121.2, 118.2, 117.8, 117.3, 117.1, 116.1, 97.9, 47.6, 36.4, 33.6, 21.7, 17.5. **IR (KBr)**: 3066, 2923, 2845, 1650, 1492, 1456, 1273, 906, 876, 759 cm^{-1}. **HRMS (ESI)** calculated for $C_{34}H_{28}N_2O_4NaS_2Br_2$ [M+Na]$^+$: 772.9755, found: 772.9729.

5',6-Dibromo-2'-methylene-1',9-ditosyl-1,2,4,9-tetrahydrospiro[carbazole-3,3'-indoline] [5c (3C)]:

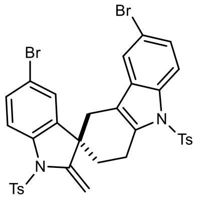

5c (3C)

Following the general method **F**, compound **5c** (3C) was obtained as a yellow solid, m.p. = 169.1 – 170.0 °C. **^{1}H NMR** (500 MHz, $CDCl_3$) δ 8.05 (d, J = 8.9 Hz, 1H), 7.80 (d, J = 8.9 Hz, 1H), 7.64 (d, J = 7.9 Hz, 2H), 7.56 (d, J = 7.9 Hz, 2H), 7.39 – 7.42 (m, 2H), 7.26 – 7.28 (m, 5H), 6.81 (s, 1H), 5.60 (d, J = 2.4 Hz, 1H), 4.45 (d, J = 2.1 Hz, 1H), 2.97 – 3.03 (m, 1H), 2.84 – 2.90 (m, 1H), 2.54 (s, 2H), 2.47 (s, 3H), 2.38 (s, 3H), 1.34 – 1.40 (m, 1H), 1.17 – 1.22 (m, 1H). **^{13}C NMR** (126 MHz, $CDCl_3$) δ 152.2, 145.4, 145.1, 139.7, 139.1, 135.5, 135.3, 134.9, 133.9, 131.6, 131.1, 130.3, 129.5, 127.4, 127.3, 126.2, 125.8, 120.9, 118.4, 117.9, 117.0, 115.9, 97.8, 46.0, 34.7, 32.9, 21.8, 21.7, 20.9. **IR (KBr)**: 3062, 2924, 2857, 1595, 1363, 1456, 1232, 813, 773, 756 cm^{-1}. **HRMS (ESI)** calculated for $C_{34}H_{28}N_2O_4NaS_2Br_2$ [M+Na]$^+$: 772.9755, found: 772.9755.

6',7-Difluoro-2'-methylene-1',9-ditosyl-1,3,4,9-tetrahydrospiro[carbazole-2,3'-indoline] [5d (2C)]:

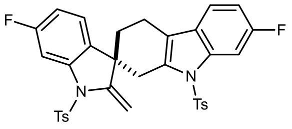

5d (2C)

Following the general method **F**, compound **5d** (2C) was obtained as a light yellow solid (25.5 mg, Yield: 78%), m.p. = 157.3 – 158.1 °C. **^{1}H NMR** (500 MHz, $CDCl_3$) δ 7.93 (dd, *J* = 10.2, 2.3 Hz, 1H), 7.69 (d, *J* = 10.4 Hz, 1H), 7.65 (s, 2H), 7.41 (d, *J* = 8.5 Hz, 2H), 7.25 – 7.28 (m, 3H), 7.09 (d, *J* = 8.5 Hz, 2H), 7.00 – 7.04 (m, 1H), 6.54 (dd, *J* = 7.0, 1.2 Hz, 2H), 5.69 (d, *J* = 2.4 Hz, 1H), 4.54 (d, *J* = 2.4 Hz, 1H), 3.03 (s, 2H), 2.42 – 2.57 (m, 2H), 2.38 (s, 3H), 2.34 (s, 3H), 1.25 – 1.33 (m, 1H), 1.16 – 1.21 (m, 1H). **^{13}C NMR** (126 MHz, $CDCl_3$) δ 163.7, 161.8 (d, *J* = 14.5 Hz), 159.9, 152.8, 145.2 (d, *J* = 4.5 Hz), 141.6 (d, *J* = 11.8 Hz), 136.8 (d, *J* = 12.7 Hz), 135.4, 133.9, 133.1 (d, *J* = 3.6 Hz), 131.9 (d, *J* = 2.7 Hz), 129.9, 129.5, 127.2, 126.2, 125.5, 123.7 (d, *J* = 10.0 Hz), 118.9 (d, *J* = 10.0 Hz), 117.6, 111.7 (d, *J* = 23.6 Hz), 111.3 (d, *J* = 21.8 Hz), 104.7 (d, *J* = 28.2 Hz), 102.4 (d, *J* = 29.1 Hz), 97.3, 46.8, 36.8, 33.9, 21.7, 21.6, 17.6. **^{19}F NMR** (282 MHz, $CDCl_3$) δ −112.56 – −112.64 (m, 1F), −116.88 – −116.97 (m, 1F). **IR (KBr)**: 3062, 2925, 2849, 1650, 1599, 1432, 1295, 931, 811, 754 cm^{-1}. **HRMS (ESI)** calculated for $C_{34}H_{28}N_2O_4F_2NaS_2$ $[M+Na]^+$: 653.1356, found: 653.1366.

6',7-Dimethyl-2'-methylene-1',9-ditosyl-1,3,4,9-tetrahydrospiro[carbazole-2,3'-indoline] [5e (2C)]:

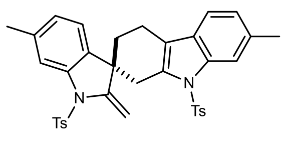

5e (2C)

Following the general method **F**, compound **5e** (2C) was obtained as a white solid (25.5 mg, Yield: 79%), m.p. = 155.7 – 156.4 °C. **^{1}H NMR** (500 MHz, $CDCl_3$) δ 8.00 (s, 1H), 7.76 (s, 1H), 7.61 (d, *J* = 8.5 Hz, 2H), 7.43 (d, *J* = 8.2 Hz, 2H), 7.21 – 7.23 (m, 3H), 7.06 – 7.09 (m, 3H), 6.69 (d, *J* = 7.6 Hz, 1H), 6.56 (d, *J* = 7.9 Hz, 1H), 5.62 (d, *J* = 2.1 Hz, 1H), 4.49 (d, *J* = 2.1 Hz, 1H), 2.99 – 3.08 (m, 2H), 2.50 (s, 3H), 2.44 – 2.48 (m, 2H), 2.40 (s, 3H), 2.36 (s, 3H), 2.32 (s, 3H), 1.24 – 1.31 (m, 1H), 1.04 – 1.09 (m, 1H). **^{13}C NMR** (126 MHz, $CDCl_3$) δ 153.0, 144.7, 144.6, 140.5, 138.4, 137.0, 135.8, 134.5, 134.2, 133.8, 132.5, 129.7, 129.4, 127.4, 127.1, 126.2, 125.5, 124.8, 122.7, 118.0, 117.8, 117.1, 114.9, 97.3, 47.1, 36.7, 33.9, 22.0, 21.8, 21.6, 21.6, 17.7. **IR (KBr)**: 3061, 2920, 2849, 1651, 1597, 1455, 1295, 868, 811, 778 cm^{-1}. **HRMS (ESI)** calculated for $C_{36}H_{34}N_2O_4NaS_2$ $[M+Na]^+$: 645.1845, found: 645.1858.

8. Applications of spiro carbazole/indole skeleton

Synthesis of (S)-2'-methyl-1,3,4,9-tetrahydrospiro[carbazole-2,3'-indole] (12):

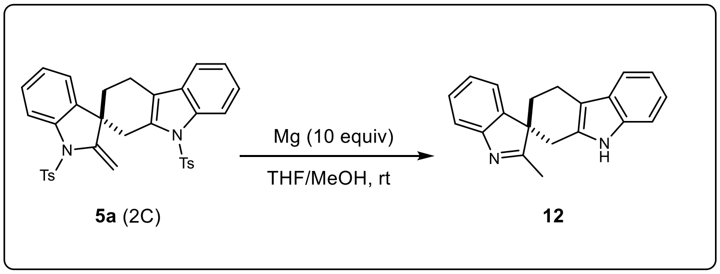

Under argon atmosphere, a flame-dried Schlenk tube was charged with compound **5a** (0.1 mmol, 1 equiv), Mg powder (1.0 mmol, 10 equiv), THF (0.3 mL) and MeOH (0.7 mL). the resulting solution was left for 24 h at room temperature under sonication. The mixture was filtered through a celite pad and concentrated under reduced pressure and purified by flash column chromatography (using Toluene) to obtain the pure product **12** as a pale yellow solid (9.2 mg, Yield: 32%), m.p. = 169.3 – 170.1 °C. **^{1}H NMR** (500 MHz, $CDCl_3$) δ 7.82 (s, 1H), 7.57 – 7.60 (m, 2H), 7.35 – 7.37 (m, 1H), 7.29 – 7.32 (m, 1H), 7.16 – 7.23 (m, 2H), 6.99 – 7.00 (m, 2H), 3.30 (d, *J* = 15.9 Hz, 1H), 3.07 – 3.12 (m, 1H), 2.99 – 3.03 (m, 1H), 2.48 (d, *J* = 15.9 Hz, 1H), 2.38 (s, 3H), 2.25 – 2.31 (m, 1H), 1.60 – 1.62 (m, 1H). **^{13}C NMR** (126 MHz, $CDCl_3$) δ 186.1, 154.0, 142.8, 136.2, 131.0, 128.0, 127.1, 125.1, 123.2, 121.8, 120.0, 119.5, 118.2, 110.7, 109.6, 56.7, 28.8, 28.8, 18.6, 16.2. **IR (KBr)**: 3415, 3060, 2927, 2846, 1590, 1452, 1379, 1258, 871, 758 cm^{-1}. **HRMS (ESI)** calculated for $C_{20}H_{19}N_2$ $[M+H]^+$: 287.1548, found: 287.1548.

Synthesis of (S)-2'-methylene-5',6-diphenyl-1',9-ditosyl-1,3,4,9-tetrahydrospiro[carbazole-2,3'-indoline] (13):

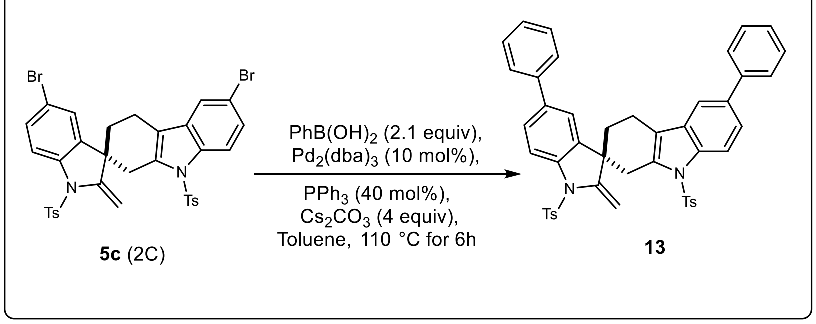

In a flame dried Schlenk tube 1 mL Toluene was taken. The solvent was degassed by using standard "freeze-pump-thaw" method. The process was repeated for three times and finally the

Schlenk tube was filled with argon gas. After that, $Pd_2(dba)_3$ (10 mol%, 2.7 mg) and triphenylphosphine (40 mol%, 3.1 mg) were taken in the tube inside the glove box and allowed to stir for 5 min. To the above mixture, **5c** (2C) (0.03 mmol, 1.0 equiv, 22.6 mg), phenylboronic acid (0.063 mmol, 2.1 equiv, 7.7 mg) and Cs_2CO_3 (0.12 mmol, 4.0 equiv, 39 mg) were added and removed from the glove box. The mixture was allowed to stir at 110 °C for 6 h under Ar. After completion, the reaction was quenched by saturated NH_4Cl aqueous solution (2 mL) at room temperature and extracted with CH_2Cl_2 (3×5 mL). The combined organic layers were washed with water and brine, then dried over Na_2SO_4, filtrated, and concentrated under vacuum. The residue was purified by silica gel column chromatography (Hexane/EtOAc = 19/1) to afford the desired product **13** (12.5 mg, yield: 56 %) as pale yellow solid. m.p. = 192.1 – 192.7 °C. **^{1}H NMR** (500 MHz, $CDCl_3$) δ 8.24 (d, *J* = 7.9 Hz, 1H), 8.00 (d, *J* = 5.5 Hz, 1H), 7.63 – 7.66 (m, 4H), 7.55 – 7.58 (m, 3H), 7.44 – 7.50 (m, 4H), 7.28 – 7.37 (m, 6H), 7.24 – 7.25 (m, 2H), 7.05 (d, *J* = 2.1 Hz, 1H), 7.00 (d, *J* = 7.9 Hz, 2H), 5.65 (d, *J* = 2.1 Hz, 1H), 4.51 (d, *J* = 2.1 Hz, 1H), 3.23 (dd, *J* = 43.3, 18.6 Hz, 2H), 2.54 – 2.56 (m, 2H), 2.38 (s, 3H), 2.19 (s, 3H), 1.39 – 1.44 (m, 1H), 1.00 – 1.05 (m, 1H). **^{13}C NMR** (126 MHz, $CDCl_3$) δ 152.5, 144.9, 141.1, 140.1, 139.8, 137.9, 137.5, 136.9, 136.0, 135.6, 134.2, 133.7, 129.8, 129.5, 128.8, 127.3, 127.3, 127.2, 127.1, 126.6, 126.2, 123.9, 121.3, 118.3, 116.9, 116.7, 114.8, 97.7, 47.5, 36.5, 33.8, 21.7, 21.5, 17.6. **IR (KBr)**: 3059, 2955, 2854, 1596, 1459, 1365, 1259, 898, 810, 746 cm^{-1}. **HRMS (ESI)** calculated for $C_{46}H_{38}N_2O_4NaS_2$ $[M+Na]^+$: 769.2171, found: 769.2181.

9. General Procedure for Cu-catalyzed decarboxylative annulation reaction of 4-ethynyl benzoxazinanone (Method G):

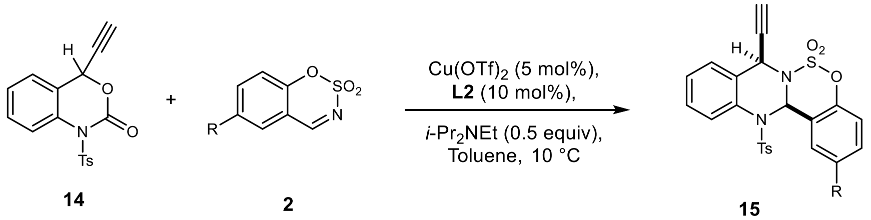

In an argon filled glove box, a flame-dried 10 mL Schlenk tube was charged with copper(II) trifluoromethanesulfonate (1.81 mg, 0.005 mmol, 5 mol%), 2,6-bis[(4*R*)-phenyl-2-oxazolin-2-yl]-pyridine **L2** (3.69 mg, 0.01 mmol, 10 mol%) and anhydrous Toluene (1 mL). The resulting solution was stirred for 1 h at 80 °C. In an argon filled glove box, 4-ethynyl benzoxazinanone **14** (0.1 mmol), sulfamate-derived cyclic imine **2** (0.11 mmol) and DIPEA (8.7 µL, 0.05 mmol, 0.5 equiv) were

added. The resulting solution was stirred at 10 °C until complete conversion of ethynyl benzoxazinanones (monitored by TLC). The reaction was quenched by saturated NH_4Cl aqueous solution (2 mL). The resulting solution was extracted with ethyl acetate (5 mL × 3). The combined organic layers were dried over Na_2SO_4, filtered and concentrated in *vacuo*. The diastereomeric ratio and crude yield were determined by ^{1}H NMR analysis of the crude reaction mixture. The residue was purified by flash silica gel chromatography to afford the title compound **15**. The characterization data of **3** are summarized below.

8-Ethynyl-13-tosyl-13,13a-dihydro-8*H*-benzo[5,6][1,2,3]oxathiazino[4,3-b]quinazoline 6,6 dioxide (15a):

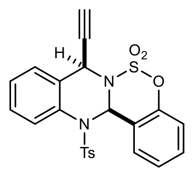

15a

Following the general method **G**, compound **15a** was obtained as a white solid (25.4 mg, Yield: 52%), m.p. = 172.1 – 173.1 °C. **^{1}H NMR** (500 MHz, $CDCl_3$) δ 8.05 (d, *J* = 9.2 Hz, 1H), 7.39 – 7.44 (m, 4H), 7.31 – 7.34 (m, 1H), 7.25 – 7.28 (m, 2H), 7.18 – 7.19 (m, 3H), 7.10 (t, *J* = 7.8 Hz, 1H), 6.92 (d, *J* = 7.3 Hz, 1H), 4.86 (d, *J* = 2.4 Hz, 1H), 2.39 (s, 3H), 2.12 (d, *J* = 2.7 Hz, 1H). **^{13}C NMR** (126 MHz, $CDCl_3$) δ 152.6, 145.1, 133.3, 131.1, 130.2, 129.5, 128.9, 128.3, 128.1, 127.6, 127.4, 126.2, 125.1, 124.9, 117.5, 117.1, 79.4, 74.8, 66.8, 45.6, 21.7. **IR (KBr)**: 3290, 3077, 2959, 1595, 1455, 1250, 924, 863, 772, 704 cm^{-1}. **HRMS (ESI)** calculated for $C_{23}H_{18}N_2O_5NaS_2$ $[M+Na]^+$: 489.0555, found: 489.0550.

2-Bromo-8-ethynyl-13-tosyl-13,13a-dihydro-8*H*-benzo[5,6][1,2,3]oxathiazino[4,3-*b*] quinazoline 6,6-dioxide (15e):

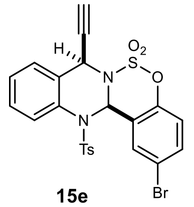

15e

Following the general method **G**, compound **15e** was obtained as a white solid (36.3 mg, Yield: 64%), m.p. = 177.7 – 178.4 °C. **^{1}H NMR** (500 MHz, $CDCl_3$) δ 8.06 – 8.07 (m, 1H), 7.55 – 7.55 (m, 1H), 7.42 – 7.48 (m, 2H), 7.37 – 7.40 (m, 3H), 7.29 – 7.32 (m, 1H), 7.18 – 7.21 (m, 3H), 6.81 (d, *J* = 8.5 Hz, 1H), 4.86 (d, *J* = 2.4 Hz, 1H), 2.39 (s, 3H), 2.16 (d, *J* = 2.4 Hz, 1H). **^{13}C NMR** (126 MHz, $CDCl_3$) δ 151.5, 145.3, 134.2, 133.1, 129.6, 129.2, 129.0, 128.3, 128.0, 127.9, 127.4, 124.6, 119.4, 119.0, 118.8, 117.8, 79.2, 75.3, 66.4, 45.6, 21.7. **IR (KBr)**: 3301, 3063, 2966, 1597, 1492, 1453, 1249, 905, 855, 704 cm^{-1}. **HRMS (ESI)** calculated for $C_{23}H_{17}N_2O_5NaS_2Br$ $[M+Na]^+$: 566.9660, found: 566.9653.

10. X-Ray crystallographic analysis of products

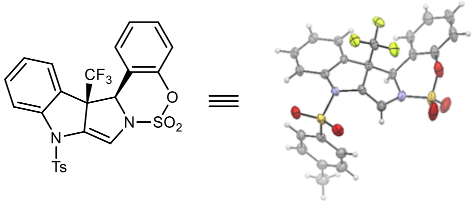

3aa CCDC: 2026703

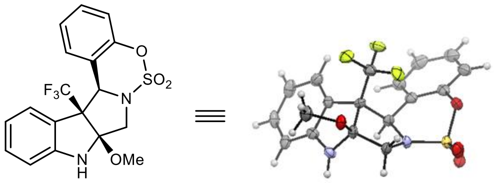

7aa CCDC: 2026704

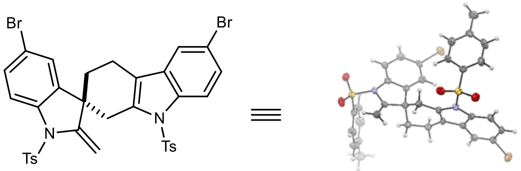

5c (2C) CCDC: 2026705

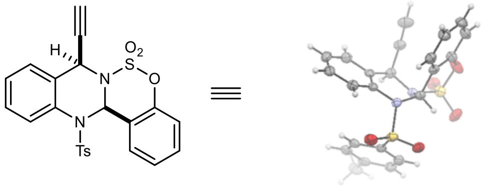

15a CCDC:2026702

11. Computational methods

Two-types of potential conformations of zwitterionic Cu-allenylidene intermediates **I** (X=CF_3), **I'** (X= Me), and **I"** (X=H) were used as initial structures for geometry optimizations. Ligand **L1** was used instead of **L2** for computation to simplify their calculations. The Gaussian 16 program[7] was used for the DFT calculations. The geometry optimizations and energy calculations were carried out at the B3LYP/6-311G** level[8] with Grimme's dispersion correction methods of the D3.[9] Atomic charge distributions were calculated from the B3LYP/6-311G** level wave functions by electrostatic potential fitting using the Merz-Singh-Kollman scheme.[10,11]

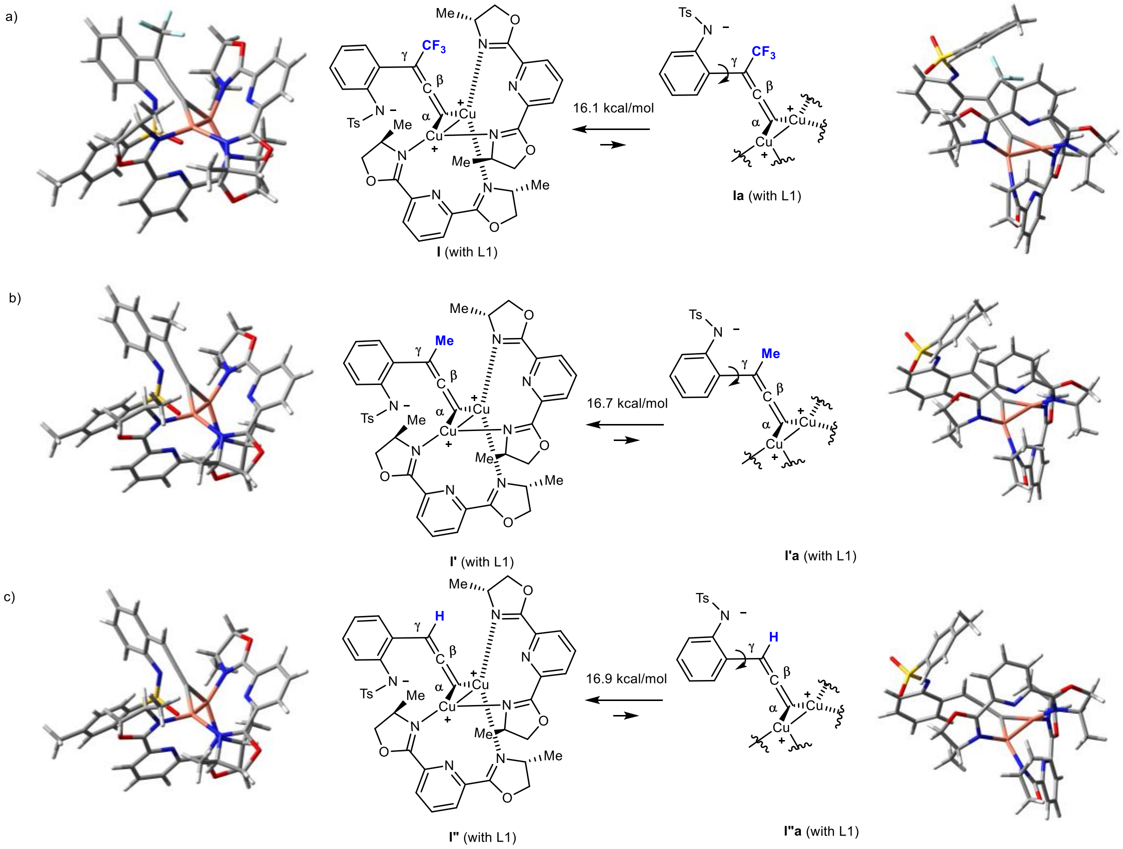

Supplementary Figure 1. a) The optimized geometries of **I** and **Ia** (with **L1**) with their relative energy. b) The optimized geometries of **I'** and **I'a** (with **L1**) with their relative energy. c) The optimized geometries of **I"** and **I"a** (with **L1**) with their relative energy.

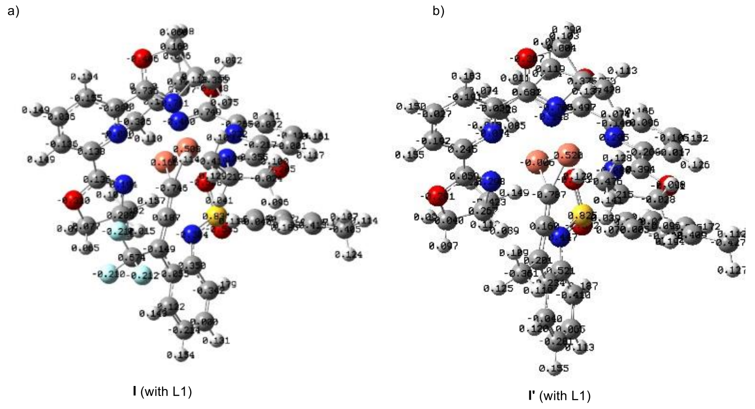

Supplementary Figure 2. Atomic charge distributions of a) **I** (with **L1**), **I'** (with **L1**) and **I''** (with **L1**).

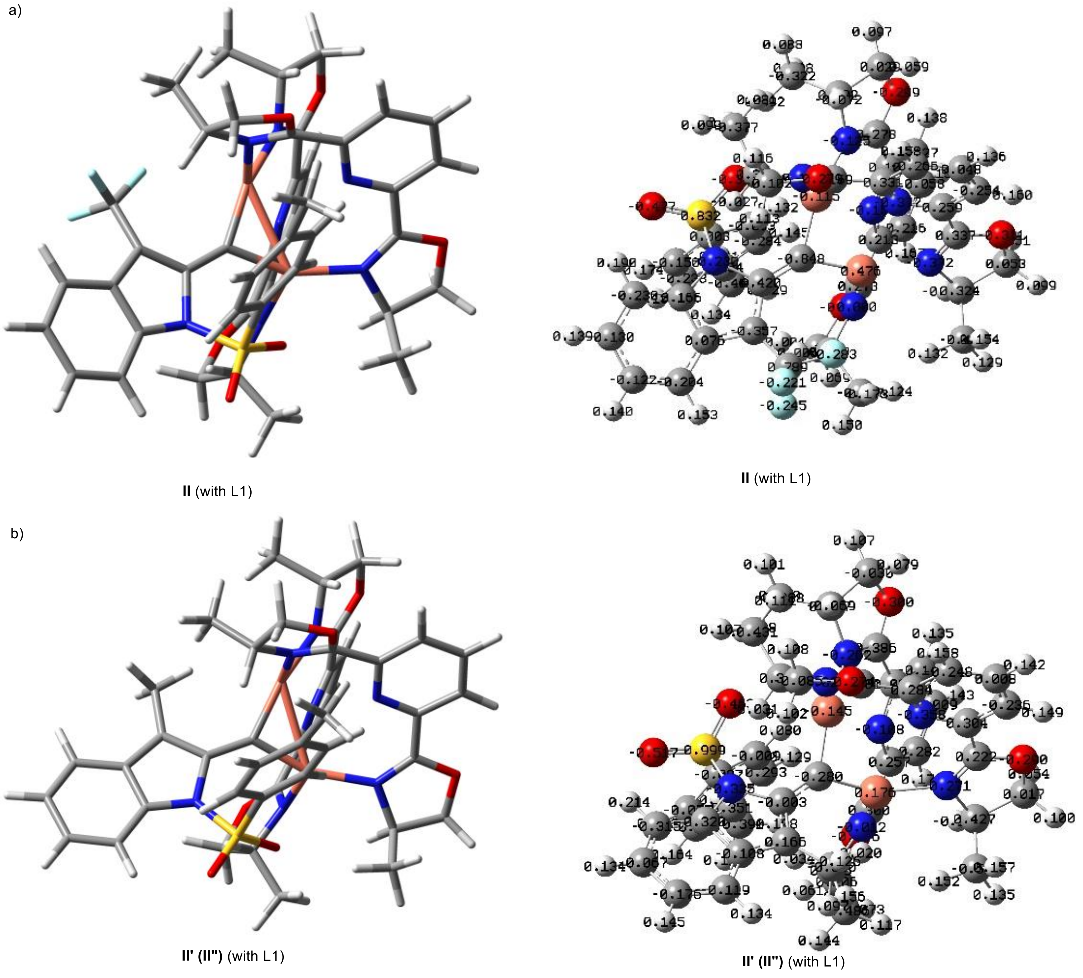

Supplementary Figure 3. a) The optimized geometry of **II** (with **L1**) with its atomic charge distributions. b) The optimized geometry of **II'** (**II''**) (with **L1**) with its atomic charge distributions.

12. HPLC Data and NMR Data:

(13b*S*,13c*R*)-9-Tosyl-13b-(trifluoromethyl)-13b,13c-dihydro-9*H*-benzo[5',6'][1,2,3] oxathiazino[3',4':1,5]pyrrolo[3,4-*b*]indole 6,6-dioxide (3aa):

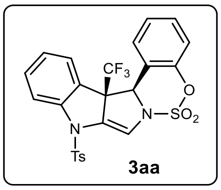

HPLC using CHIRALPAK® IC (n-hexane/isopropanol = 85.0/15.0, flow rate 1.0 mL/min, λ = 254 nm)

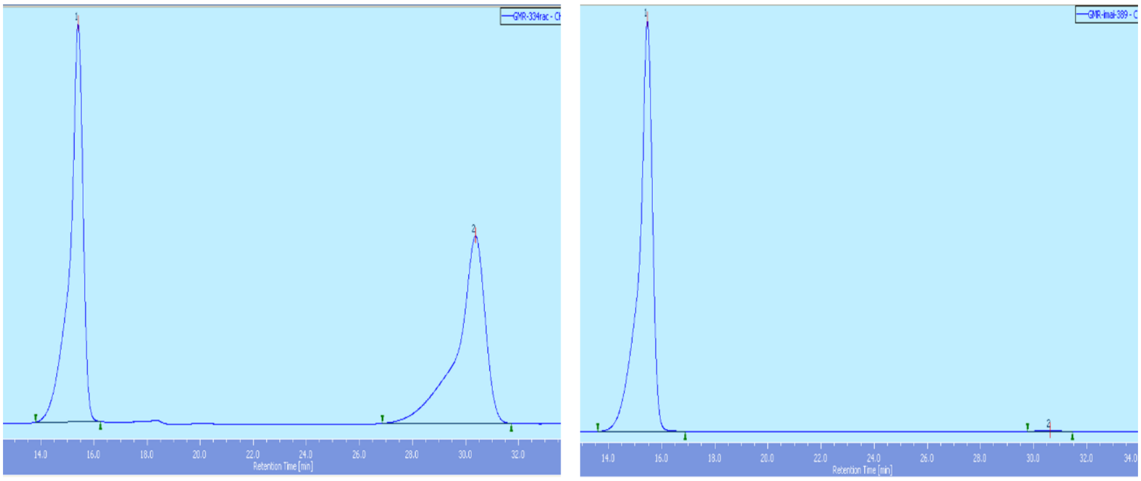

No.	tR (min)	Area (%)	Height (%)
1	15.400	50.212	68.007
2	30.358	49.788	31.993

No.	tR (min)	Area (%)	Height (%)
1	15.458	99.645	99.728
2	30.608	0.355	0.272

Supplementary Figure 4. Determination of enantiomeric excess of **3aa**.

(13b*S*,13c*R*)-2-Methyl-9-tosyl-13b-(trifluoromethyl)-13b,13c-dihydro-9*H*-benzo [5',6'][1,2,3]oxathiazino[3',4':1,5]pyrrolo[3,4-*b*]indole 6,6-dioxide (3ab):

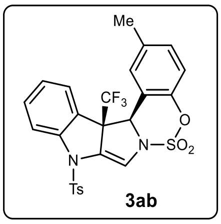

HPLC using CHIRALPAK® IC (n-hexane/isopropanol = 85.0/15.0, flow rate 1.0 mL/min, λ = 254 nm).

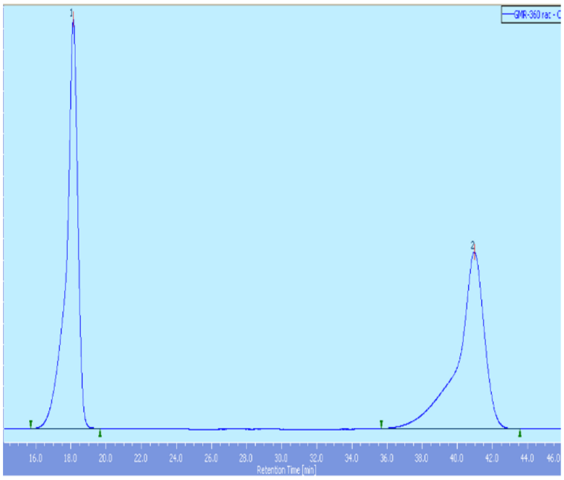

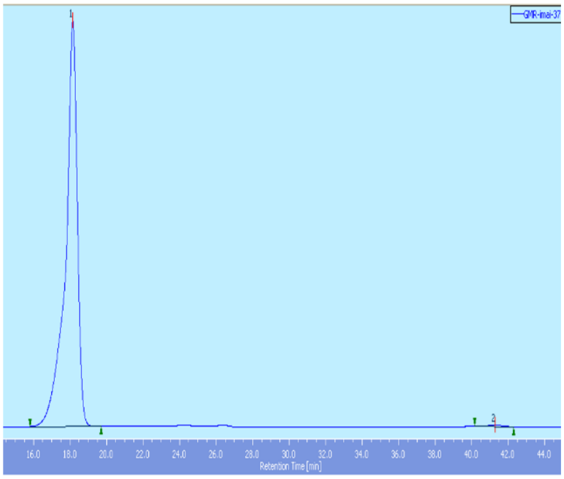

No.	tR (min)	Area (%)	Height (%)
1	18.125	50.019	69.818
2	40.950	49.981	30.182

No.	tR (min)	Area (%)	Height (%)
1	18.150	99.506	99.651
2	41.242	0.494	0.349

Supplementary Figure 5. Determination of enantiomeric excess of **3ab**.

(13b*S*,13c*R*)-2-Methoxy-9-tosyl-13b-(trifluoromethyl)-13b,13c-dihydro-9*H*-benzo [5',6'][1,2,3]oxathiazino[3',4':1,5]pyrrolo[3,4-*b*]indole 6,6-dioxide (3ac):

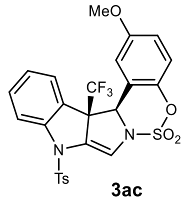

3ac

HPLC using CHIRALPAK® IA (n-hexane/isopropanol = 95.0/5.0, flow rate 1.0 mL/min, λ = 254 nm).

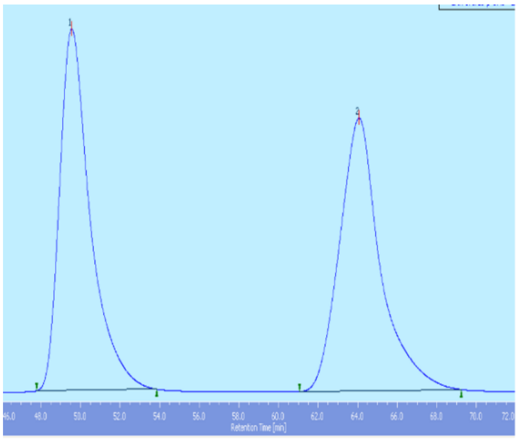

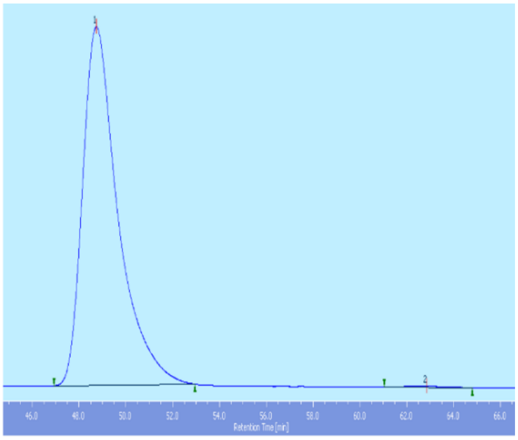

No.	tR (min)	Area (%)	Height (%)
1	49.550	49.949	56.990
2	64.050	50.051	43.010

No.	tR (min)	Area (%)	Height (%)
1	48.742	99.566	99.616
2	62.825	0.434	0.384

Supplementary Figure 6. Determination of enantiomeric excess of **3ac**.

(13b*S*,13c*R*)-2-Fluoro-9-tosyl-13b-(trifluoromethyl)-13b,13c-dihydro-9*H*-benzo [5',6'][1,2,3]oxathiazino[3',4':1,5]pyrrolo[3,4-*b*]indole 6,6-dioxide (3ad):

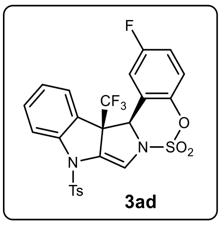

HPLC using CHIRALPAK® IC (n-hexane/isopropanol = 85.0/15.0, flow rate 1.0 mL/min, λ = 254 nm).

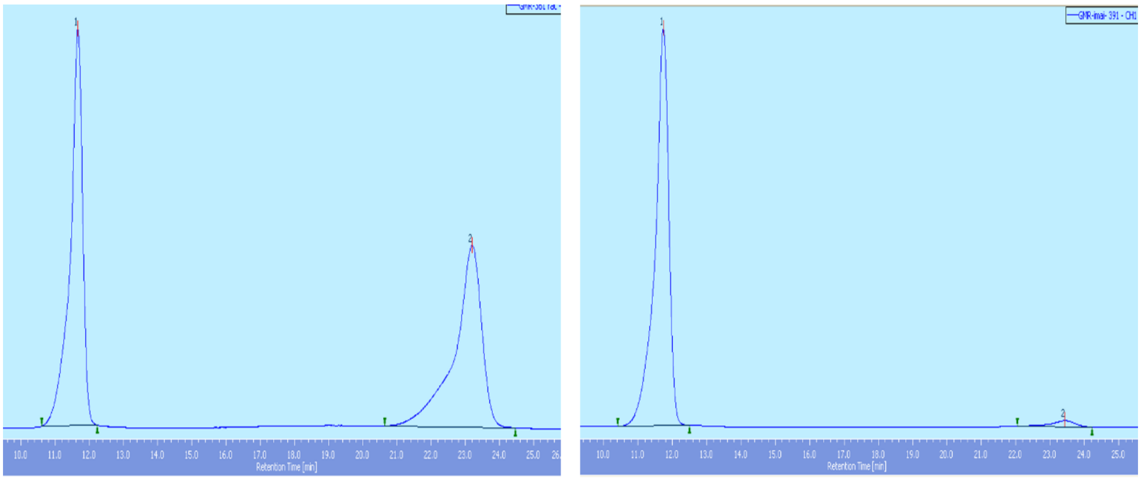

No.	tR (min)	Area (%)	Height (%)
1	11.667	50.024	68.551
2	23.183	49.976	31.449

No.	tR (min)	Area (%)	Height (%)
1	11.733	97.402	98.489
2	23.408	2.598	1.511

Supplementary Figure 7. Determination of enantiomeric excess of **3ad**.

(13b*S*,13c*R*)-2-Bromo-9-tosyl-13b-(trifluoromethyl)-13b,13c-dihydro-9*H*-benzo [5',6'][1,2,3]oxathiazino[3',4':1,5]pyrrolo[3,4-*b*]indole 6,6-dioxide (3ae):

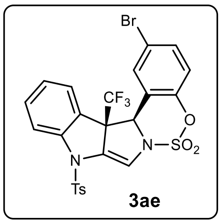

HPLC using CHIRALPAK® IA (n-hexane/isopropanol = 95.0/5.0, flow rate 1.0 mL/min, λ = 254 nm).

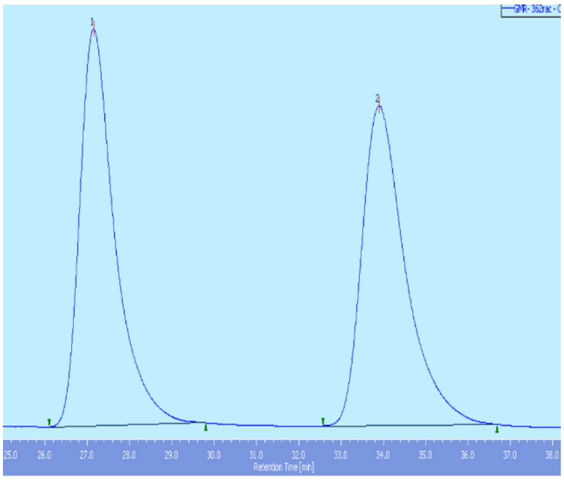

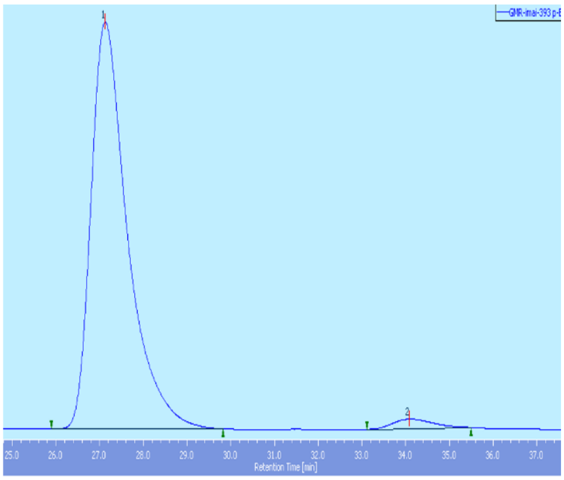

No.	tR (min)	Area (%)	Height (%)
1	27.158	50.013	55.386
2	33.883	49.987	44.614

No.	tR (min)	Area (%)	Height (%)
1	27.125	97.522	97.711
2	34.067	2.478	2.289

Supplementary Figure 8. Determination of enantiomeric excess of **3ae**.

(13b*S*,13c*R*)-2-Nitro-9-tosyl-13b-(trifluoromethyl)-13b,13c-dihydro-9*H*-benzo [5',6'][1,2,3]oxathiazino[3',4':1,5]pyrrolo[3,4-*b*]indole 6,6-dioxide (3af):

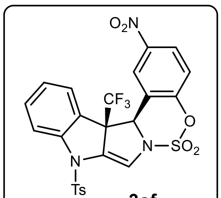

3af

HPLC using CHIRALPAK® IC (n-hexane/isopropanol = 85.0/15.0, flow rate 1.0 mL/min, λ = 254 nm).

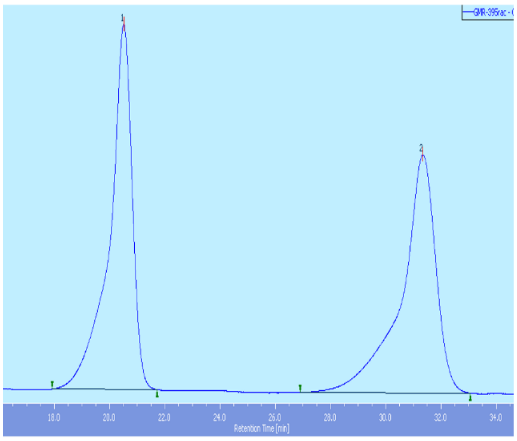

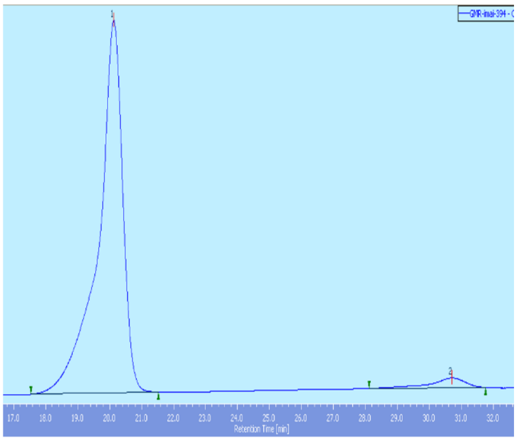

No.	tR (min)	Area (%)	Height (%)
1	20.508	50.059	60.533
2	31.325	49.941	39.467

No.	tR (min)	Area (%)	Height (%)
1	20.125	96.310	97.410
2	30.692	3.690	2.590

Supplementary Figure 9. Determination of enantiomeric excess of **3af**.

(13b*S*,13c*R*)-3-Methyl-9-tosyl-13b-(trifluoromethyl)-13b,13c-dihydro-9*H*-benzo [5',6'][1,2,3]oxathiazino[3',4':1,5]pyrrolo[3,4-*b*]indole 6,6-dioxide (3ag):

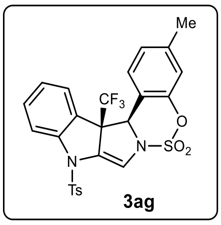

HPLC using CHIRALPAK® IC (n-hexane/isopropanol = 85.0/15.0, flow rate 1.0 mL/min, λ = 254 nm).

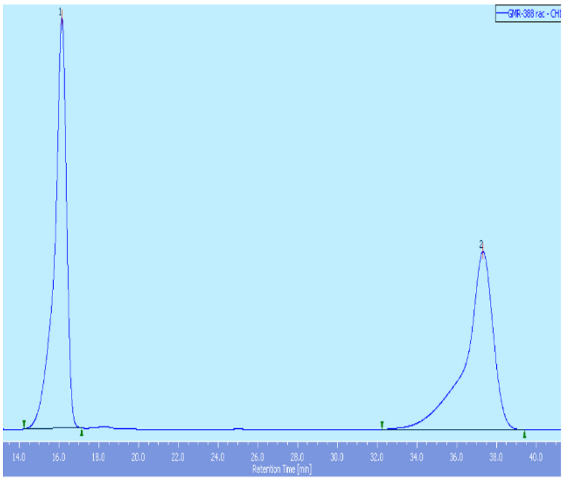

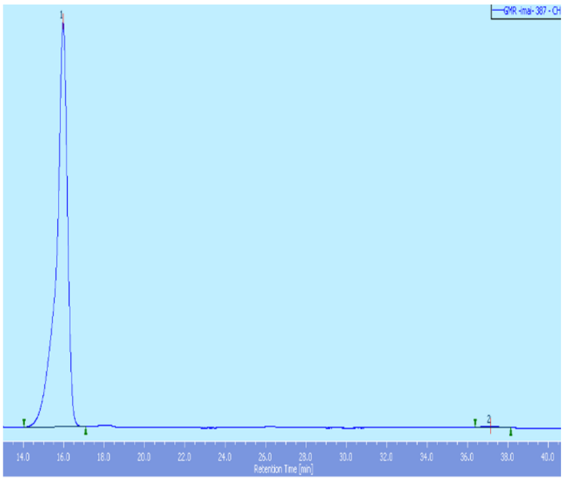

No.	tR (min)	Area (%)	Height (%)
1	16.142	50.103	69.760
2	37.300	49.897	30.240

No.	tR (min)	Area (%)	Height (%)
1	15.975	99.638	99.759
2	37.100	0.362	0.241

Supplementary Figure 10. Determination of enantiomeric excess of **3ag**.

(13b*S*,13c*R*)-3-Methoxy-9-tosyl-13b-(trifluoromethyl)-13b,13c-dihydro-9*H*-benzo [5',6'][1,2,3]oxathiazino[3',4':1,5]pyrrolo[3,4-*b*]indole 6,6-dioxide (3ah):

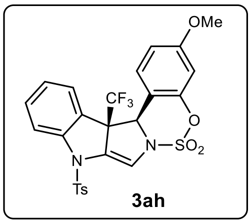

HPLC using CHIRALPAK® IC (n-hexane/isopropanol = 85.0/15.0, flow rate 1.0 mL/min, λ = 254 nm).

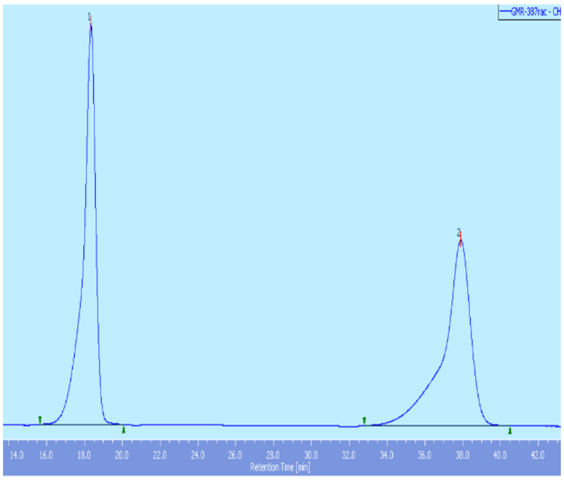

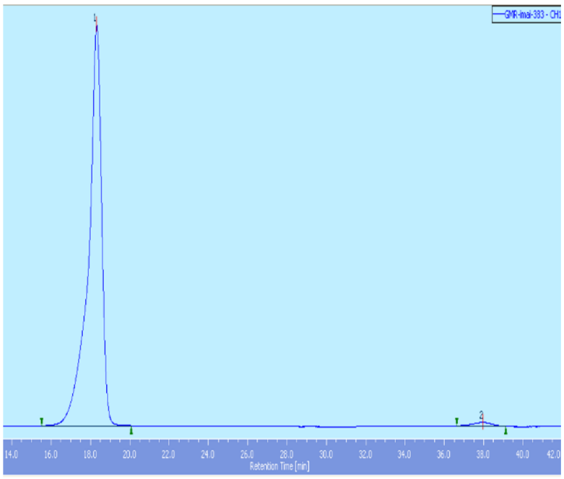

No.	tR (min)	Area (%)	Height (%)
1	18.333	50.134	68.353
2	37.875	49.866	31.647

No.	tR (min)	Area (%)	Height (%)
1	18.317	98.698	99.087
2	37.942	1.302	0.913

Supplementary Figure 11. Determination of enantiomeric excess of **3ah**.

(13b*S*,13c*R*)-3-Chloro-9-tosyl-13b-(trifluoromethyl)-13b,13c-dihydro-9*H*-benzo [5',6'][1,2,3]oxathiazino[3',4':1,5]pyrrolo[3,4-*b*]indole 6,6-dioxide (3ai):

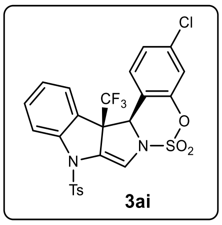

HPLC using CHIRALPAK® IC (n-hexane/isopropanol = 85.0/15.0, flow rate 1.0 mL/min, λ = 254 nm).

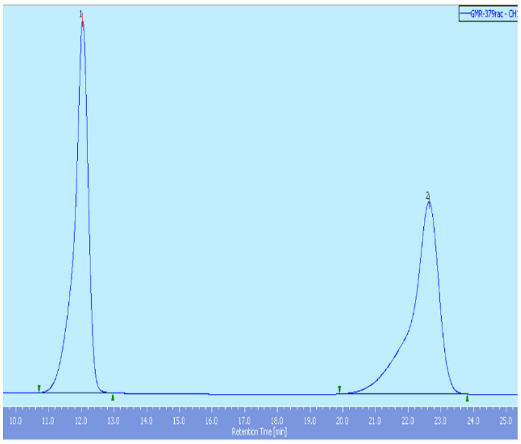

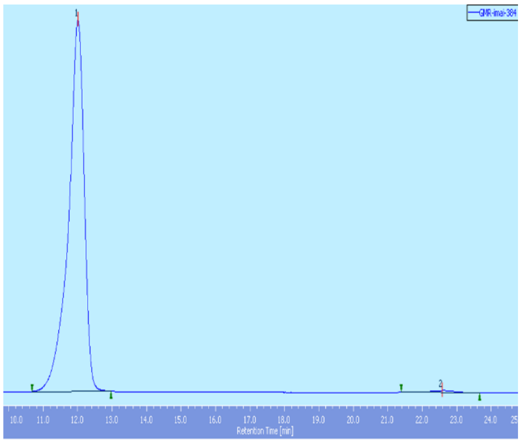

No.	tR (min)	Area (%)	Height (%)
1	12.042	50.102	65.933
2	22.625	49.898	34.067

No.	tR (min)	Area (%)	Height (%)
1	12.000	99.146	99.457
2	22.567	0.854	0.543

Supplementary Figure 12. Determination of enantiomeric excess of **3ai**.

(13b*S*,13c*R*)-9-Tosyl-13b-(trifluoromethyl)-13b,13c-dihydro-9*H*-naphtho[2'',1'':5',6'][1,2,3]oxathiazino[3',4':1,5]pyrrolo[3,4-*b*]indole 6,6-dioxide (3aj):

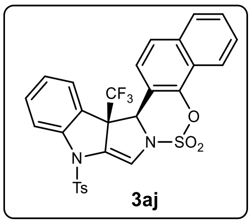

HPLC using CHIRALPAK® IC (n-hexane/isopropanol = 85.0/15.0, flow rate 1.0 mL/min, λ = 254 nm).

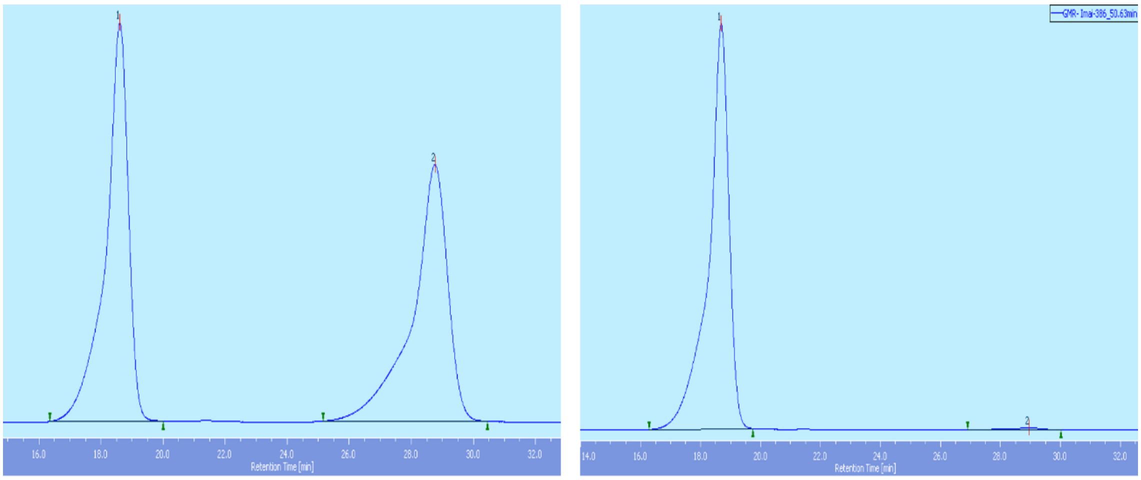

No.	tR (min)	Area (%)	Height (%)
1	18.600	50.084	60.843
2	28.750	49.916	39.157

No.	tR (min)	Area (%)	Height (%)
1	18.683	99.338	99.550
2	28.933	0.662	0.450

Supplementary Figure 13. Determination of enantiomeric excess of **3aj**.

(13b*S*,13c*R*)-9-Tosyl-11,13b-bis(trifluoromethyl)-13b,13c-dihydro-9*H*-benzo[5',6'][1,2,3]oxathiazino[3',4':1,5]pyrrolo[3,4-*b*]indole 6,6-dioxide (3ba):

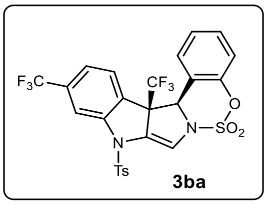

HPLC using CHIRALPAK® IG (n-hexane/isopropanol = 90.0/10.0, flow rate 1.0 mL/min, λ = 254 nm).

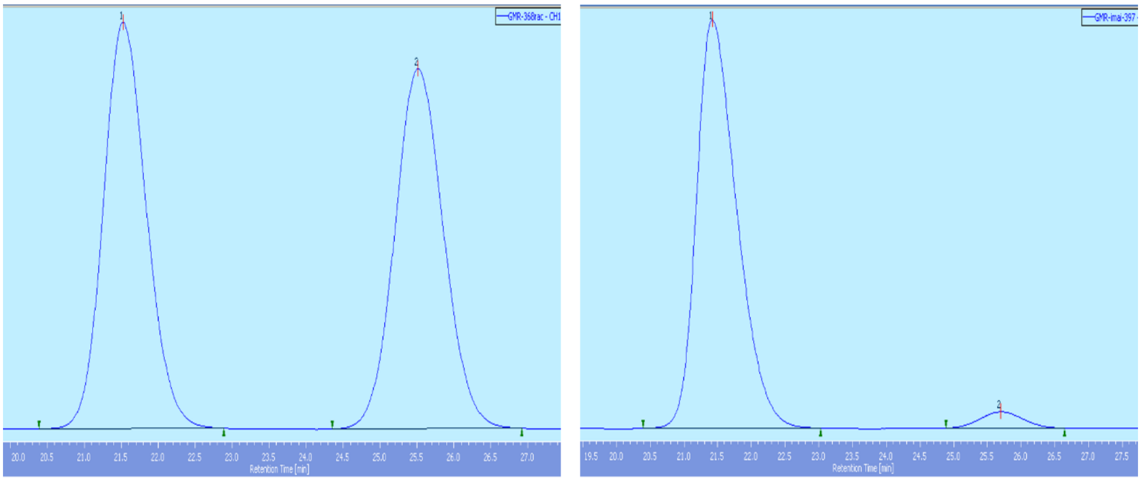

No.	tR (min)	Area (%)	Height (%)
1	21.525	49.913	53.006
2	25.508	50.087	46.994

No.	tR (min)	Area (%)	Height (%)
1	21.417	95.910	96.152
2	25.692	4.090	3.848

Supplementary Figure 14. Determination of enantiomeric excess of **3ba**.

Methyl (13b*S*,13c*R*)-9-tosyl-13b-(trifluoromethyl)-13b,13c-dihydro-9*H*-benzo[5',6'][1,2,3] oxathiazino[3',4':1,5]pyrrolo[3,4-*b*]indole-11-carboxylate 6,6-dioxide (3ca):

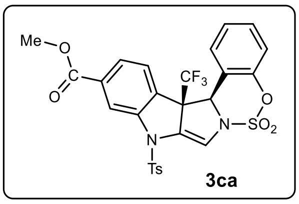

HPLC using CHIRALPAK® IC (n-hexane/isopropanol = 95.0/5.0, flow rate 1.0 mL/min, λ = 254 nm).

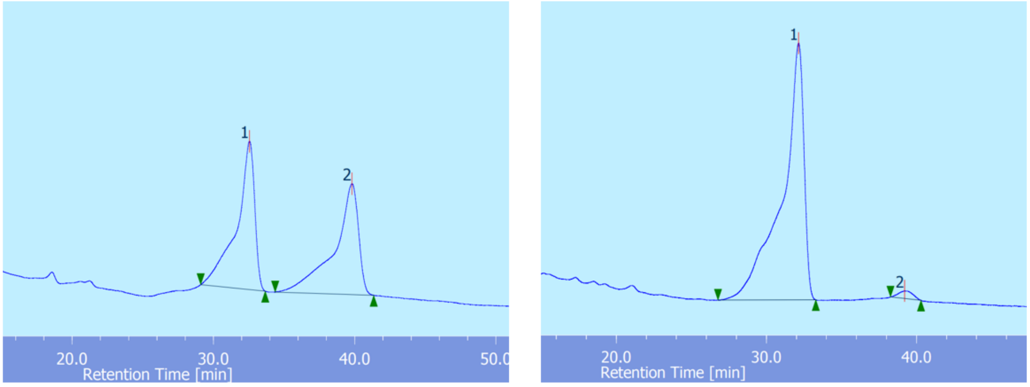

No.	tR(min)	Area (%)	Height (%)
1	32.542	50.01	57.253
2	39.792	49.99	42.747

No.	tR(min)	Area (%)	Height (%)
1	32.133	98.039	97.064
2	39.2	1.961	2.936

Supplementary Figure 15. Determination of enantiomeric excess of **3ca**.

(13b*S*,13c*R*)-12-Fluoro-9-tosyl-13b-(trifluoromethyl)-13b,13c-dihydro-9*H*-benzo [5',6'][1,2,3]oxathiazino[3',4':1,5]pyrrolo[3,4-*b*]indole 6,6-dioxide (3da):

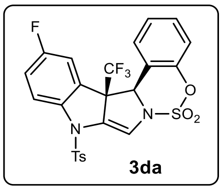

HPLC using CHIRALPAK® IC (n-hexane/isopropanol = 85.0/15.0, flow rate 1.0 mL/min, λ = 254 nm).

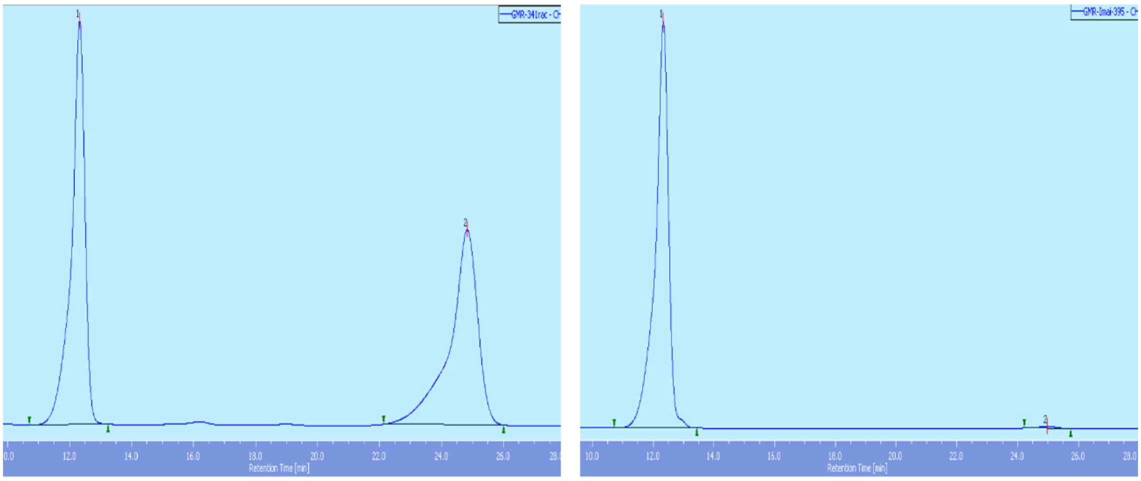

No.	tR (min)	Area (%)	Height (%)
1	12.325	50.010	67.418
2	24.817	49.990	32.582

No.	tR (min)	Area (%)	Height (%)
1	12.333	99.369	99.542
2	24.967	0.631	0.458

Supplementary Figure 16. Determination of enantiomeric excess of **3da**.

(13b*S*,13cR)-12-Fluoro-2-methyl-9-tosyl-13b-(trifluoromethyl)-13b,13c-dihydro-9*H*-benzo[5',6'][1,2,3]oxathiazino[3',4':1,5]pyrrolo[3,4-*b*]indole 6,6-dioxide (3db):

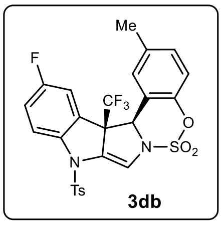

HPLC using CHIRALPAK® IG (n-hexane/isopropanol = 95.0/5.0, flow rate 1.0 mL/min, λ = 254 nm).

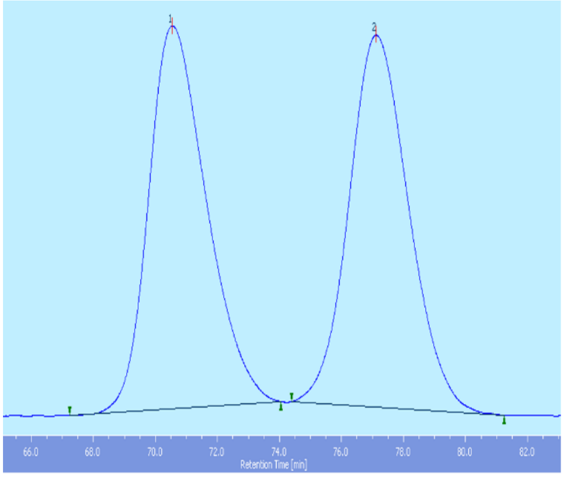

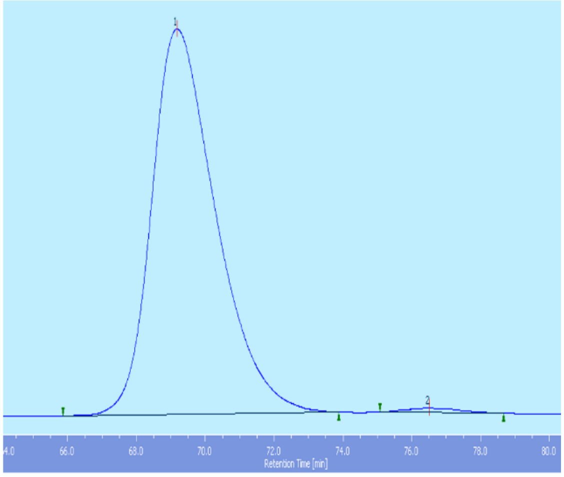

No.	tR (min)	Area (%)	Height (%)
1	70.542	49.907	50.728
2	77.092	50.093	49.272

No.	tR (min)	Area (%)	Height (%)
1	69.183	99.163	98.893
2	76.500	0.837	1.107

Supplementary Figure 17. Determination of enantiomeric excess of **3db**.

(13b*S*,13c*R*)-12-Chloro-9-tosyl-13b-(trifluoromethyl)-13b,13c-dihydro-9*H*-benzo [5',6'][1,2,3]oxathiazino[3',4':1,5]pyrrolo[3,4-*b*]indole 6,6-dioxide (3ea):

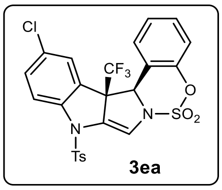

HPLC using CHIRALPAK® IC (n-hexane/isopropanol = 85.0/15.0, flow rate 1.0 mL/min, λ = 254 nm).

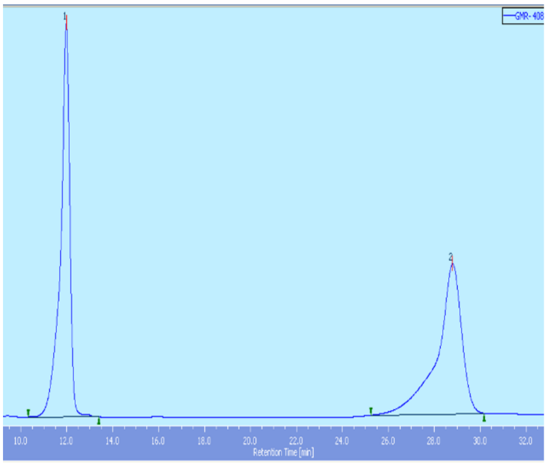

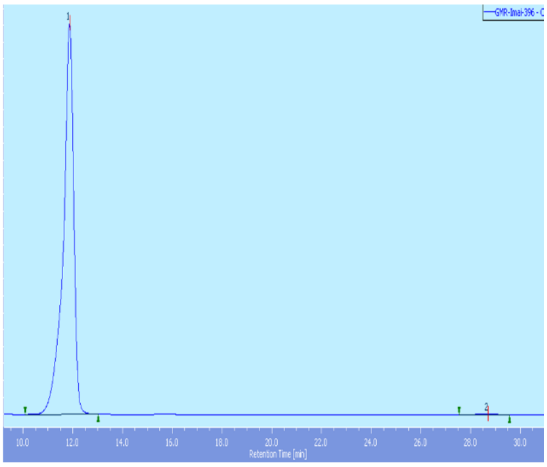

No.	tR (min)	Area (%)	Height (%)
1	11.975	50.015	72.373
2	28.775	49.985	27.627

No.	tR (min)	Area (%)	Height (%)
1	11.867	99.364	99.638
2	28.683	0.636	0.362

Supplementary Figure 18. Determination of enantiomeric excess of **3ea**.

(13b*S*,13c*R*)-12-Chloro-2-methoxy-9-tosyl-13b-(trifluoromethyl)-13b,13c-dihydro-9*H*-benzo[5',6'][1,2,3]oxathiazino[3',4':1,5]pyrrolo[3,4-*b*]indole 6,6-dioxide (3ec):

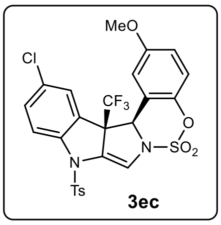

HPLC using CHIRALPAK® IC (n-hexane/isopropanol = 90.0/10.0, flow rate 1.0 mL/min, λ = 254 nm).

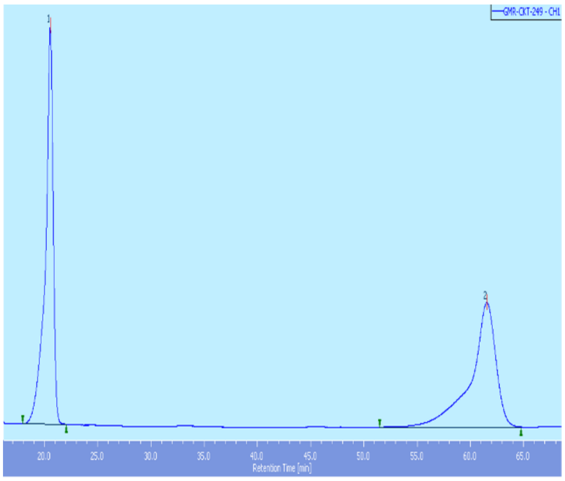

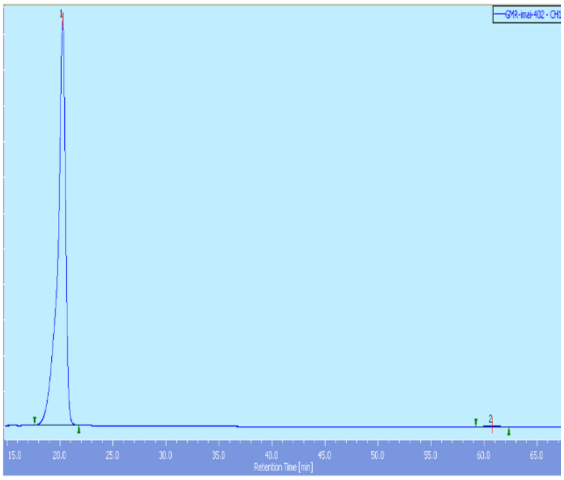

No.	tR (min)	Area (%)	Height (%)
1	20.550	49.950	76.144
2	61.492	50.050	23.856

No.	tR (min)	Area (%)	Height (%)
1	20.258	99.518	99.739
2	60.733	0.482	0.261

Supplementary Figure 19. Determination of enantiomeric excess of **3ec**.

(13b*S*,13c*R*)-12-Methyl-9-tosyl-13b-(trifluoromethyl)-13b,13c-dihydro-9*H*-benzo [5',6'][1,2,3]oxathiazino[3',4':1,5]pyrrolo[3,4-*b*]indole 6,6-dioxide (3fa):

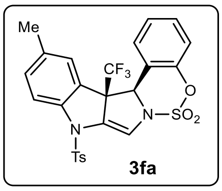

HPLC using CHIRALPAK® IC (n-hexane/isopropanol = 85.0/15.0, flow rate 1.0 mL/min, λ = 254 nm).

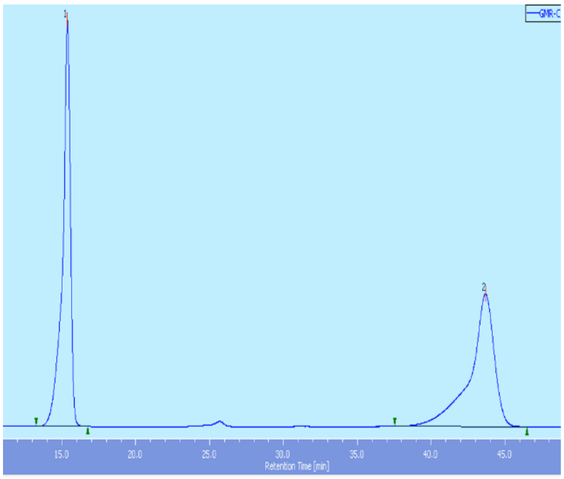

No.	tR (min)	Area (%)	Height (%)
1	15.367	50.033	75.347
2	43.650	49.967	24.653

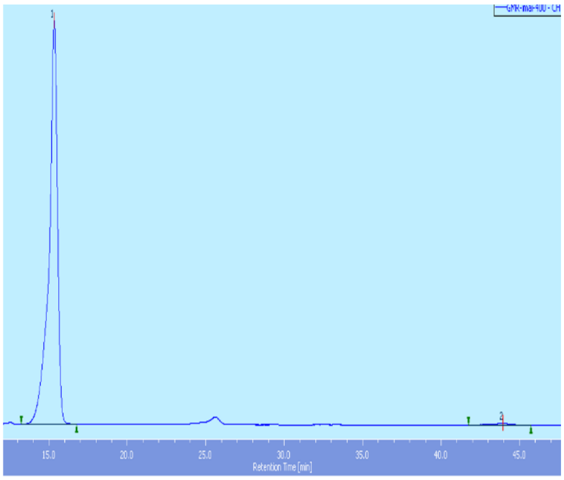

No.	tR (min)	Area (%)	Height (%)
1	15.333	99.200	99.619
2	43.892	0.800	0.381

Supplementary Figure 20. Determination of enantiomeric excess of **3fa**.

(13b*S*,13c*R*)-2-Phenyl-9-tosyl-13b-(trifluoromethyl)-13b,13c-dihydro-9*H*-benzo [5',6'][1,2,3]oxathiazino[3',4':1,5]pyrrolo[3,4-*b*]indole 6,6-dioxide (6):

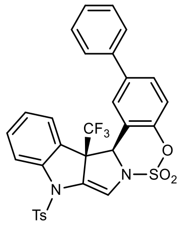

6

HPLC using CHIRALPAK® IC (n-hexane/isopropanol = 85.0/15.0, flow rate 1.0 mL/min, λ = 254 nm).

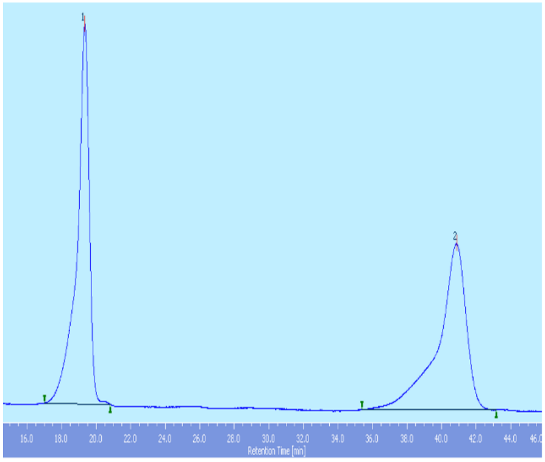

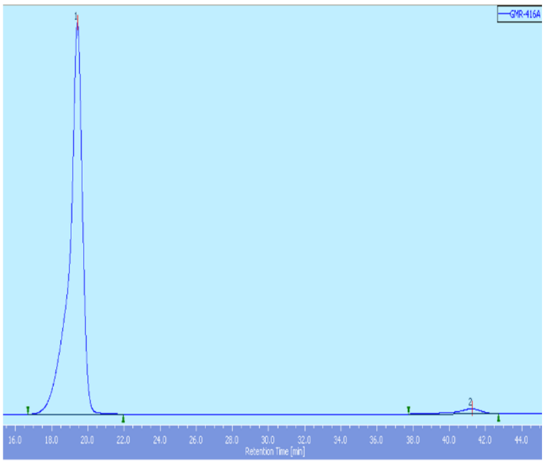

No.	tR (min)	Area (%)	Height (%)
1	19.342	50.009	69.543
2	40.850	49.991	30.457

No.	tR (min)	Area (%)	Height (%)
1	19.425	97.498	98.708
2	41.225	2.502	1.292

Supplementary Figure 21. Determination of enantiomeric excess of **6**.

(13b*S*,13c*R*)-8a-Methoxy-13b-(trifluoromethyl)-8a,9,13b,13c-tetrahydro-8*H*-benzo [5',6'][1,2,3]oxathiazino[3',4':1,5]pyrrolo[3,4-*b*]indole 6,6-dioxide (7aa):

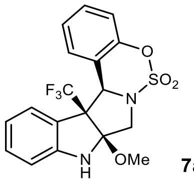

7aa

HPLC using CHIRALPAK® OD-3 (n-hexane/isopropanol = 95.0/5.0, flow rate 1.0 mL/min, λ = 254 nm).

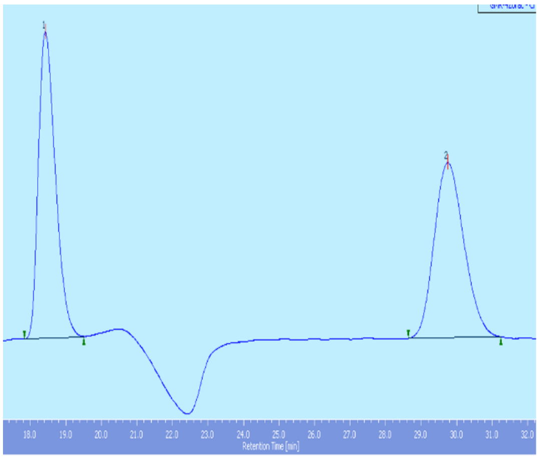

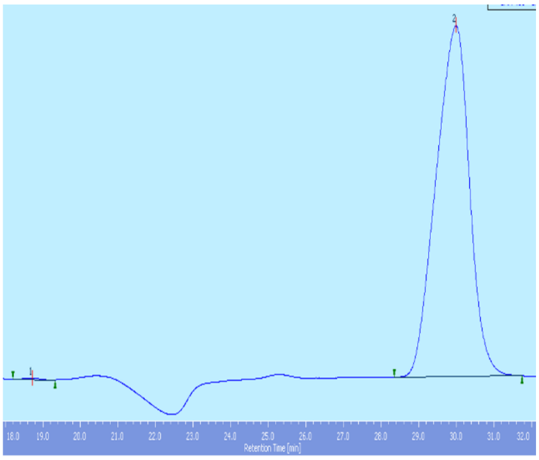

No.	tR (min)	Area (%)	Height (%)
1	18.425	49.911	63.617
2	29.733	50.089	36.383

No.	tR (min)	Area (%)	Height (%)
1	18.717	0.169	0.385
2	29.975	99.831	99.615

Supplementary Figure 22. Determination of enantiomeric excess of **7aa**.

(8a*S*,13b*S*,13c*R*)-8a-methoxy-2-methyl-13b-(trifluoromethyl)-8a,9,13b,13c-tetra hydro-8*H*-benzo[5',6'][1,2,3]oxathiazino[3',4':1,5]pyrrolo[3,4-b]indole-6,6-dioxide (7ab):

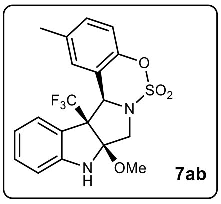

HPLC using CHIRALPAK® OD-3 (n-hexane/isopropanol = 95.0/5.0, flow rate 1.0 mL/min, λ = 254 nm).

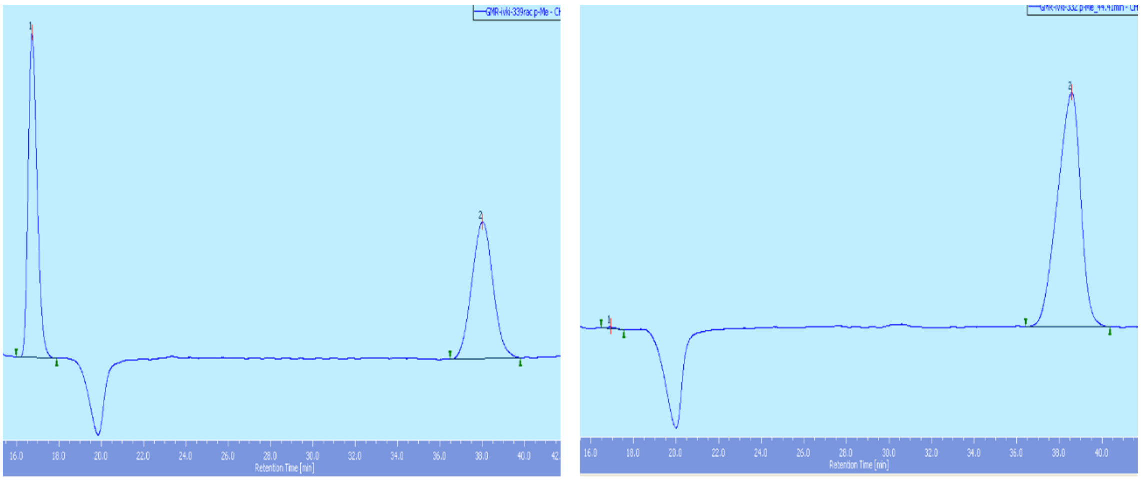

No.	tR (min)	Area (%)	Height (%)
1	16.725	49.908	70.345
2	37.992	50.092	29.655

No.	tR (min)	Area (%)	Height (%)
1	16.933	0.292	0.758
2	38.558	99.708	99.242

Supplementary Figure 23. Determination of enantiomeric excess of **7ab**.

(8a*S*,13b*S*,13c*R*)-2,8a-dimethoxy-13b-(trifluoromethyl)-8a,9,13b,13c-tetrahydro-8*H*-benzo[5',6'][1,2,3]oxathiazino[3',4':1,5]pyrrolo[3,4-b]indole 6,6-dioxide (7ac):

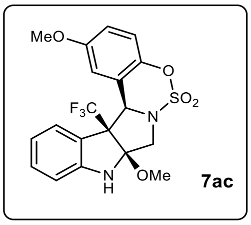

HPLC using CHIRALPAK® IA (n-hexane/isopropanol = 95.0/5.0, flow rate 1.0 mL/min, λ = 254 nm).

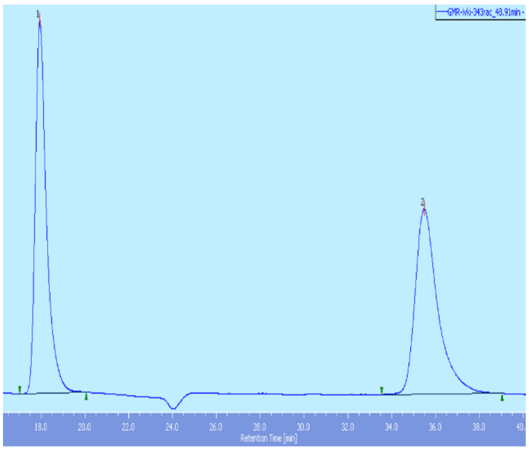

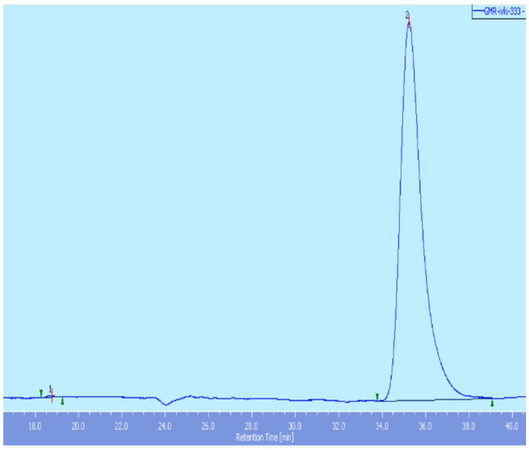

No.	tR (min)	Area (%)	Height (%)
1	17.942	50.007	66.814
2	35.458	49.993	33.186

No.	tR (min)	Area (%)	Height (%)
1	18.767	0.183	0.475
2	35.225	99.817	99.525

Supplementary Figure 24. Determination of enantiomeric excess of **7ac**.

(8a*S*,13b*S*,13c*R*)-2-fluoro-8a-methoxy-13b-(trifluoromethyl)-8a,9,13b,13c-tetrahydro-8*H*-benzo[5',6'][1,2,3]oxathiazino[3',4':1,5]pyrrolo[3,4-b]indole 6,6-dioxide (7ad):

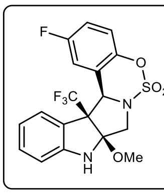

7ad

HPLC using CHIRALPAK® OD-3 (n-hexane/isopropanol = 95.0/5.0, flow rate 1.0 mL/min, λ = 230 nm).

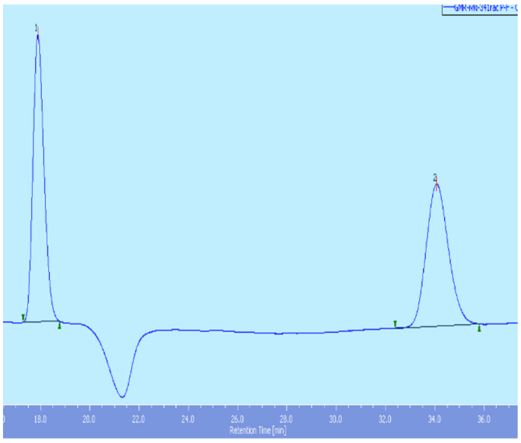

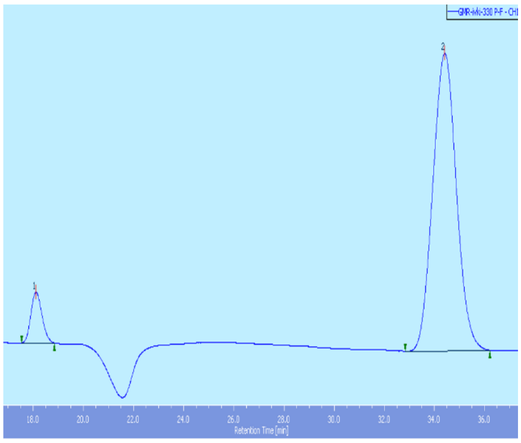

No.	tR (min)	Area (%)	Height (%)
1	17.892	50.049	66.937
2	34.058	49.951	33.063

No.	tR (min)	Area (%)	Height (%)
1	18.117	7.468	14.603
2	34.392	92.532	85.397

Supplementary Figure 25. Determination of enantiomeric excess of **7ad**.

(8a*S*,13b*S*,13c*R*)-8a-methoxy-3-methyl-13b-(trifluoromethyl)-8a,9,13b,13c-tetra hydro-8*H*-benzo[5',6'][1,2,3]oxathiazino[3',4':1,5]pyrrolo[3,4-b]indole 6,6-dioxide (7ag):

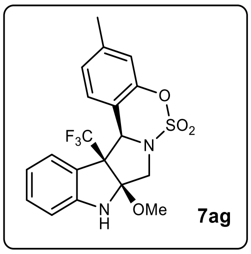

HPLC using CHIRALPAK® OD-3 (n-hexane/isopropanol = 95.0/5.0, flow rate 1.0 mL/min, λ = 230 nm).

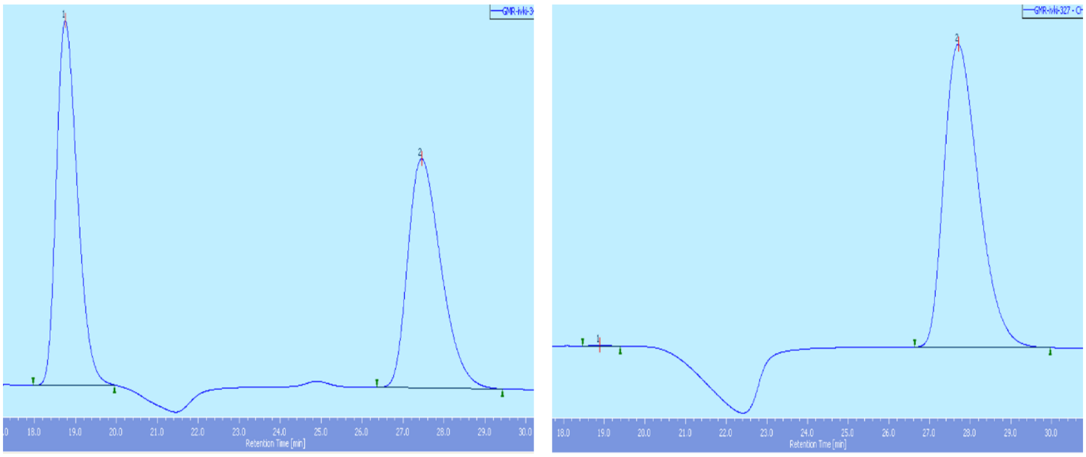

No.	tR (min)	Area (%)	Height (%)
1	18.750	50.052	61.388
2	27.442	49.948	38.612

No.	tR (min)	Area (%)	Height (%)
1	18.892	0.059	0.117
2	27.700	99.941	99.883

Supplementary Figure 26. Determination of enantiomeric excess of **7ag**.

(8a*S*,13b*S*,13c*R*)-3,8a-dimethoxy-13b-(trifluoromethyl)-8a,9,13b,13c-tetrahydro-8*H*-benzo[5',6'][1,2,3]oxathiazino[3',4':1,5]pyrrolo[3,4-b]indole 6,6-dioxide (7ah):

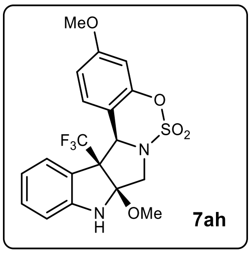

HPLC using CHIRALPAK® OD-3 (n-hexane/isopropanol = 95.0/5.0, flow rate 1.0 mL/min, λ = 230 nm).

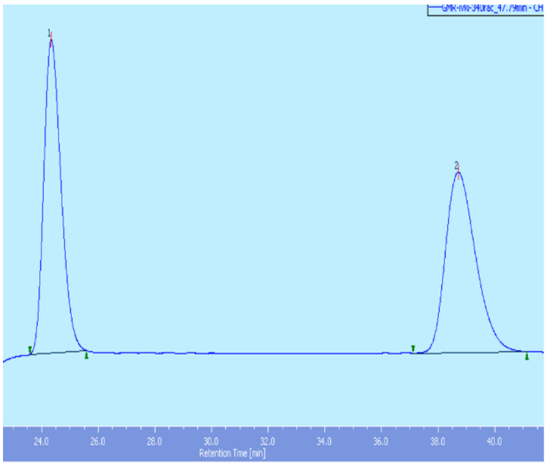

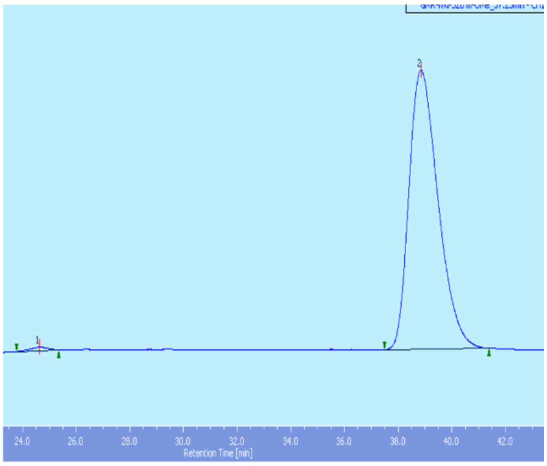

No.	tR (min)	Area (%)	Height (%)
1	24.333	49.886	63.477
2	38.700	50.114	36.523

No.	tR (min)	Area (%)	Height (%)
1	24.617	0.707	1.163
2	38.833	99.293	98.837

Supplementary Figure 27. Determination of enantiomeric excess of **7ah**.

(8a*S*,13b*S*,13c*R*)-12-fluoro-8a-methoxy-2-methyl-13b-(trifluoromethyl)-8a,9,13b,13c-tetrahydro-8*H*-benzo[5',6'][1,2,3]oxathiazino[3',4':1,5]pyrrolo[3,4-b]indole 6,6-dioxide (7db):

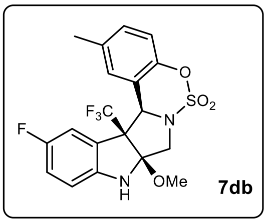

HPLC using CHIRALPAK® IA (n-hexane/isopropanol = 90.0/10.0, flow rate 1.0 mL/min, λ = 254 nm).

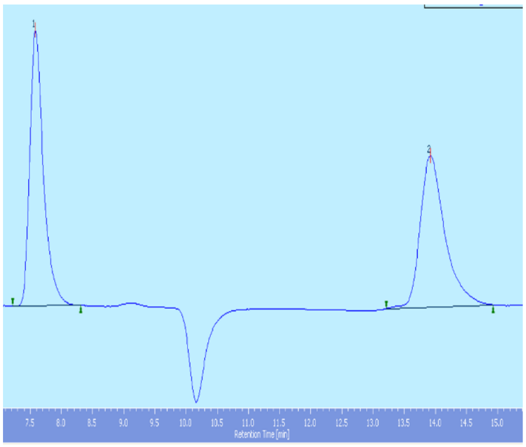

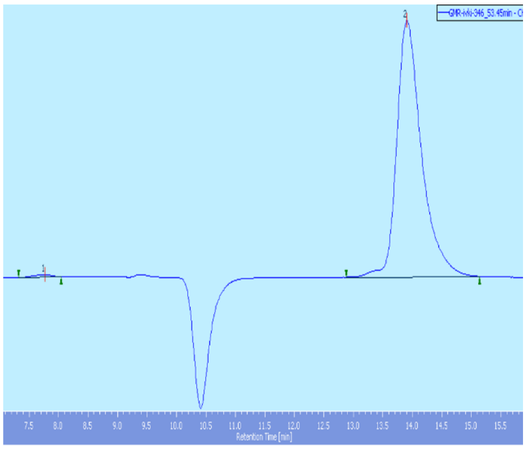

No.	tR (min)	Area (%)	Height (%)
1	7.583	50.042	64.579
2	13.917	49.958	35.421

No.	tR (min)	Area (%)	Height (%)
1	7.767	0.659	0.980
2	13.900	99.341	99.020

Supplementary Figure 28. Determination of enantiomeric excess of **7db**.

(8aS,13bS,13cR)-12-chloro-8a-methoxy-13b-(trifluoromethyl)-8a,9,13b,13c-tetrahydro-8H-benzo[5',6'][1,2,3]oxathiazino[3',4':1,5]pyrrolo[3,4-b]indole 6,6-dioxide (7ea):

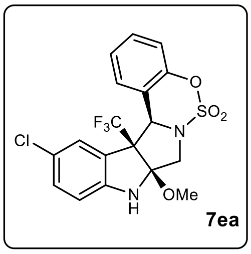

HPLC using CHIRALPAK® OD-3 (n-hexane/isopropanol = 97.0/3.0, flow rate 0.8 mL/min, λ = 254 nm).

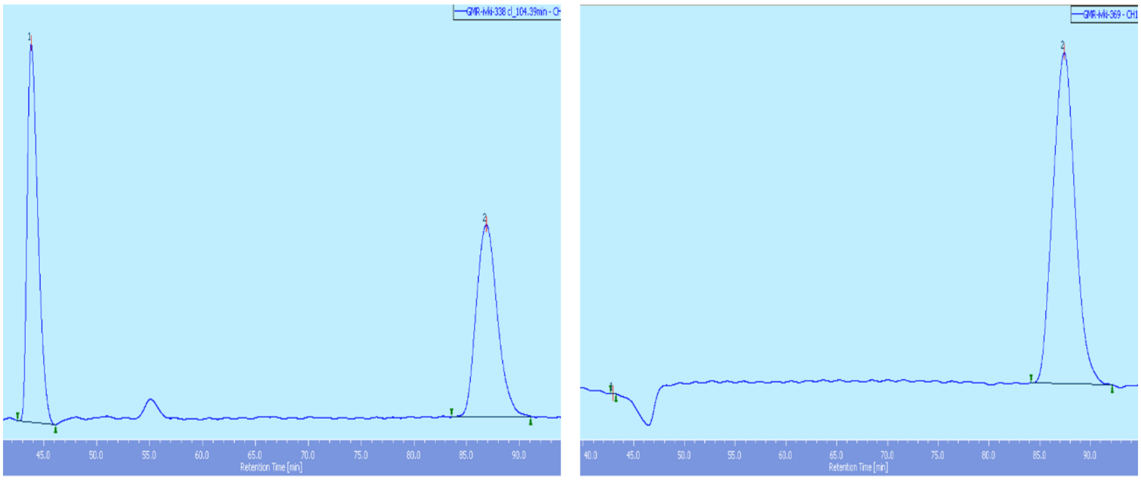

No.	tR (min)	Area (%)	Height (%)
1	43.800	49.921	66.310
2	86.800	50.079	33.690

No.	tR (min)	Area (%)	Height (%)
1	42.958	0.010	0.083
2	87.358	99.990	99.917

Supplementary Figure 29. Determination of enantiomeric excess of **7ea**.

(8a*R*,13b*S*,13c*R*)-8a-allyl-13b-(trifluoromethyl)-8a,9,13b,13c-tetrahydro-8*H*-benzo[5',6'] [1,2,3]oxathiazino[3',4':1,5]pyrrolo[3,4-b]indole (8):

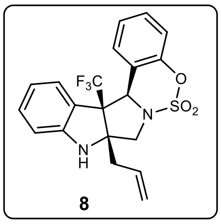

HPLC using CHIRALPAK® IA (n-hexane/isopropanol = 95.0/5.0, flow rate 1.0 mL/min, λ = 254 nm).

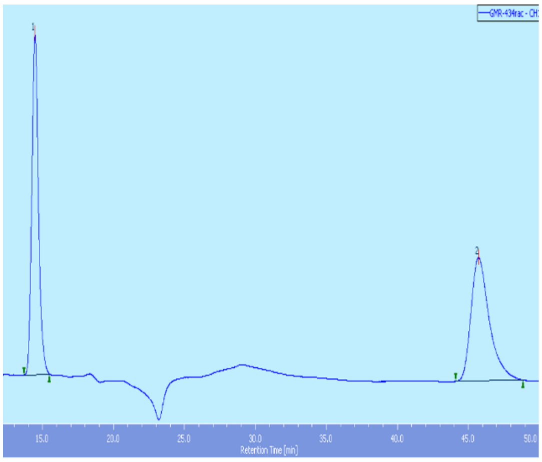

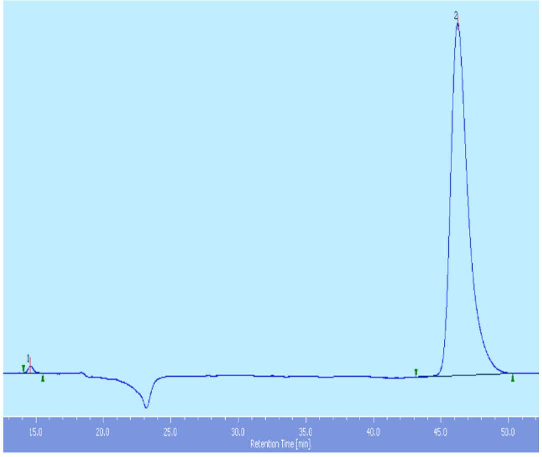

No.	tR (min)	Area (%)	Height (%)
1	14.467	50.030	73.463
2	45.692	49.970	26.537

No.	tR (min)	Area (%)	Height (%)
1	14.575	0.635	2.000
2	46.192	99.365	98.000

Supplementary Figure 30. Determination of enantiomeric excess of **8**.

(8a*R*,13b*S*,13c*R*)-13b-(trifluoromethyl)-13b,13c-dihydro-8*H*-benzo[5',6'][1,2,3] oxathiazino [3',4':1,5]pyrrolo[3,4-b]indole-8a(9*H*)-carbonitrile 6,6-dioxide (9):

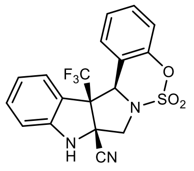

9

HPLC using CHIRALPAK® OD-3 (n-hexane/isopropanol = 95.0/5.0, flow rate 1.0 mL/min, λ = 254 nm).

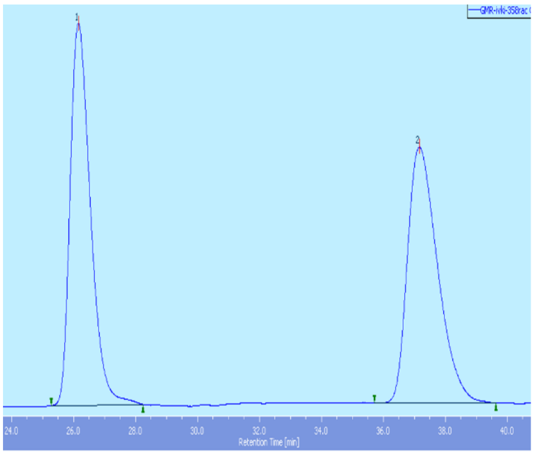

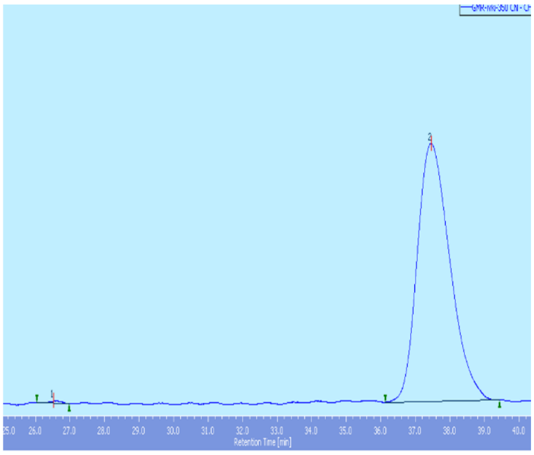

No.	tR (min)	Area (%)	Height (%)
1	26.150	50.041	59.865
2	37.150	49.959	40.135

No.	tR (min)	Area (%)	Height (%)
1	26.525	0.366	0.882
2	37.450	99.664	99.118

Supplementary Figure 31. Determination of enantiomeric excess of **9**.

(8a*R*,13b*R*,13c*R*)-13b-(trifluoromethyl)-8a,9,13b,13c-tetrahydro-8*H*-benzo[5',6'][1,2,3]oxathiazino[3',4':1,5]pyrrolo[3,4-b]indole 6,6-dioxide (10):

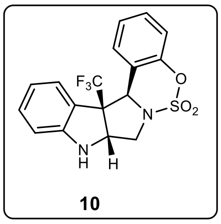

HPLC using CHIRALPAK® OD-3 (n-hexane/isopropanol = 95.0/5.0, flow rate 1.0 mL/min, λ = 254 nm).

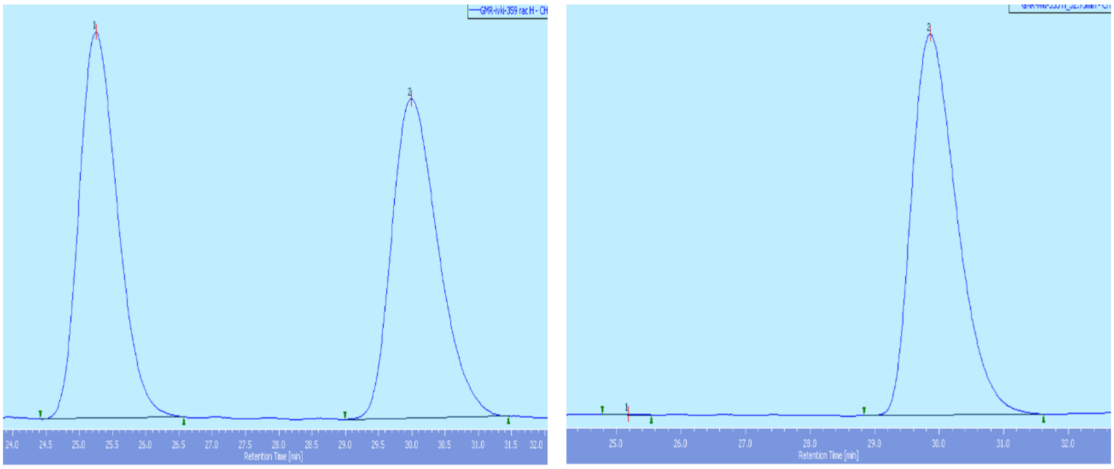

No.	tR (min)	Area (%)	Height (%)
1	25.250	49.994	54.705
2	29.992	50.006	45.295

No.	tR (min)	Area (%)	Height (%)
1	25.183	0.108	0.237
2	29.858	99.892	99.763

Supplementary Figure 32. Determination of enantiomeric excess of **10**.

(8a*R*,13b*S*,13c*R*)-8a-(1*H*-indol-3-yl)-13b-(trifluoromethyl)-8a,9,13b,13c-tetrahydro-8*H*-benzo[5',6'][1,2,3]oxathiazino[3',4':1,5]pyrrolo[3,4-b]indole 6,6-dioxide (11):

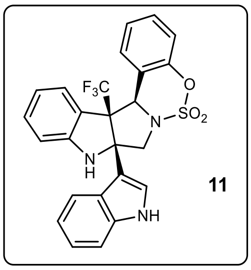

HPLC using CHIRALPAK® IA (n-hexane/isopropanol = 90.0/10.0, flow rate 1.0 mL/min, λ = 254 nm).

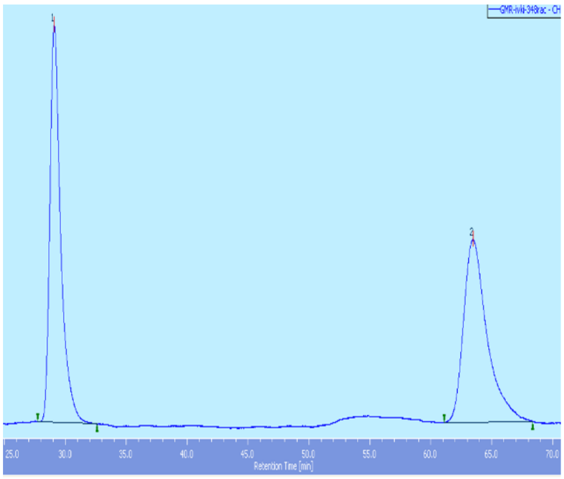

No.	tR (min)	Area (%)	Height (%)
1	29.092	50.057	68.518
2	63.442	49.943	31.482

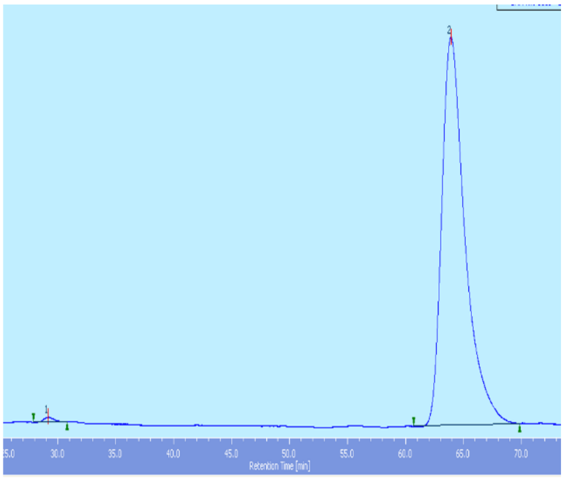

No.	tR (min)	Area (%)	Height (%)
1	29.183	0.527	1.239
2	63.867	99.473	98.761

Supplementary Figure 33. Determination of enantiomeric excess of **11**.

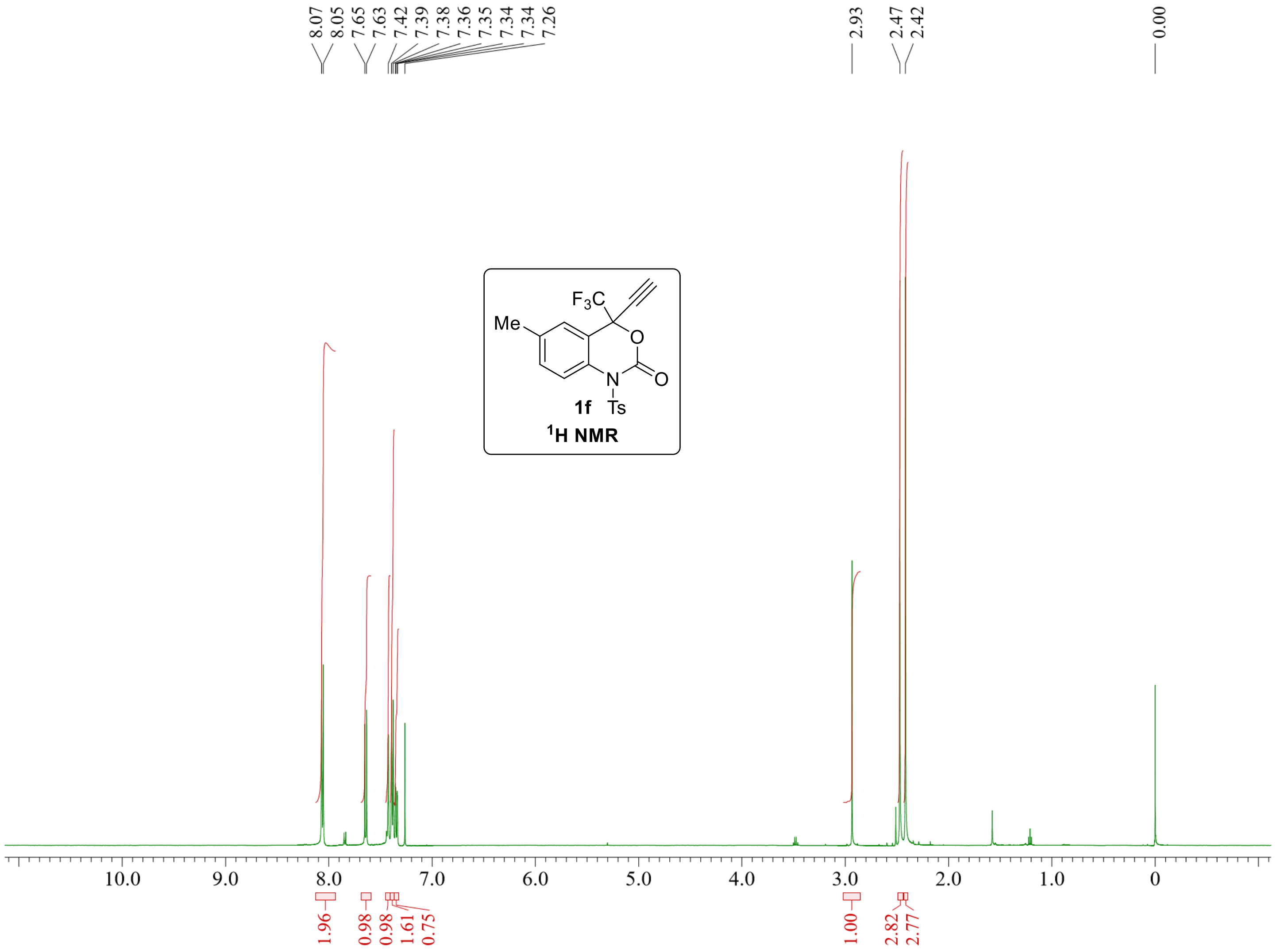

Supplementary Figure 34. ^{1}H NMR of **1f**.

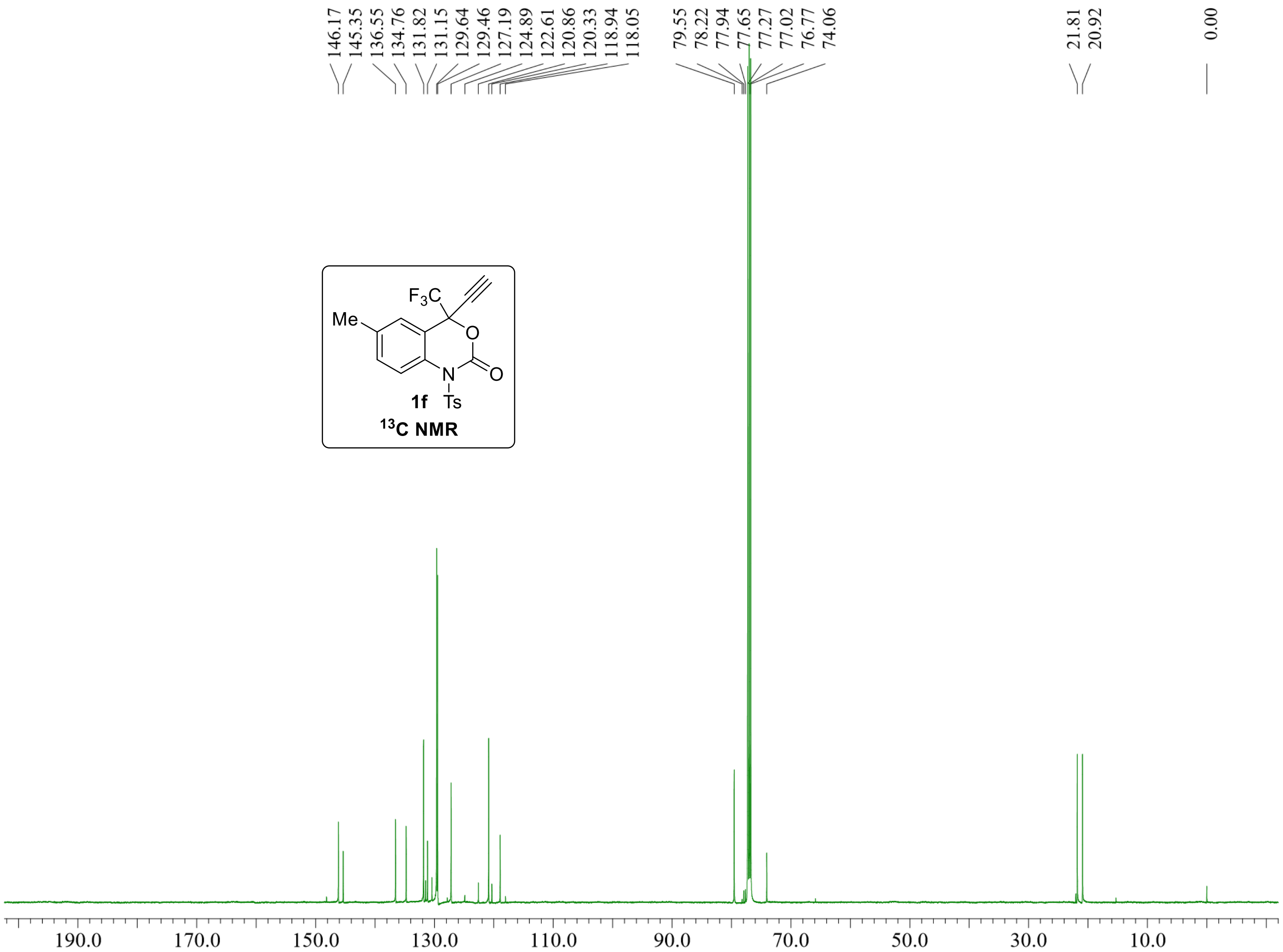

Supplementary Figure 35. ^{13}C NMR of **1f**.

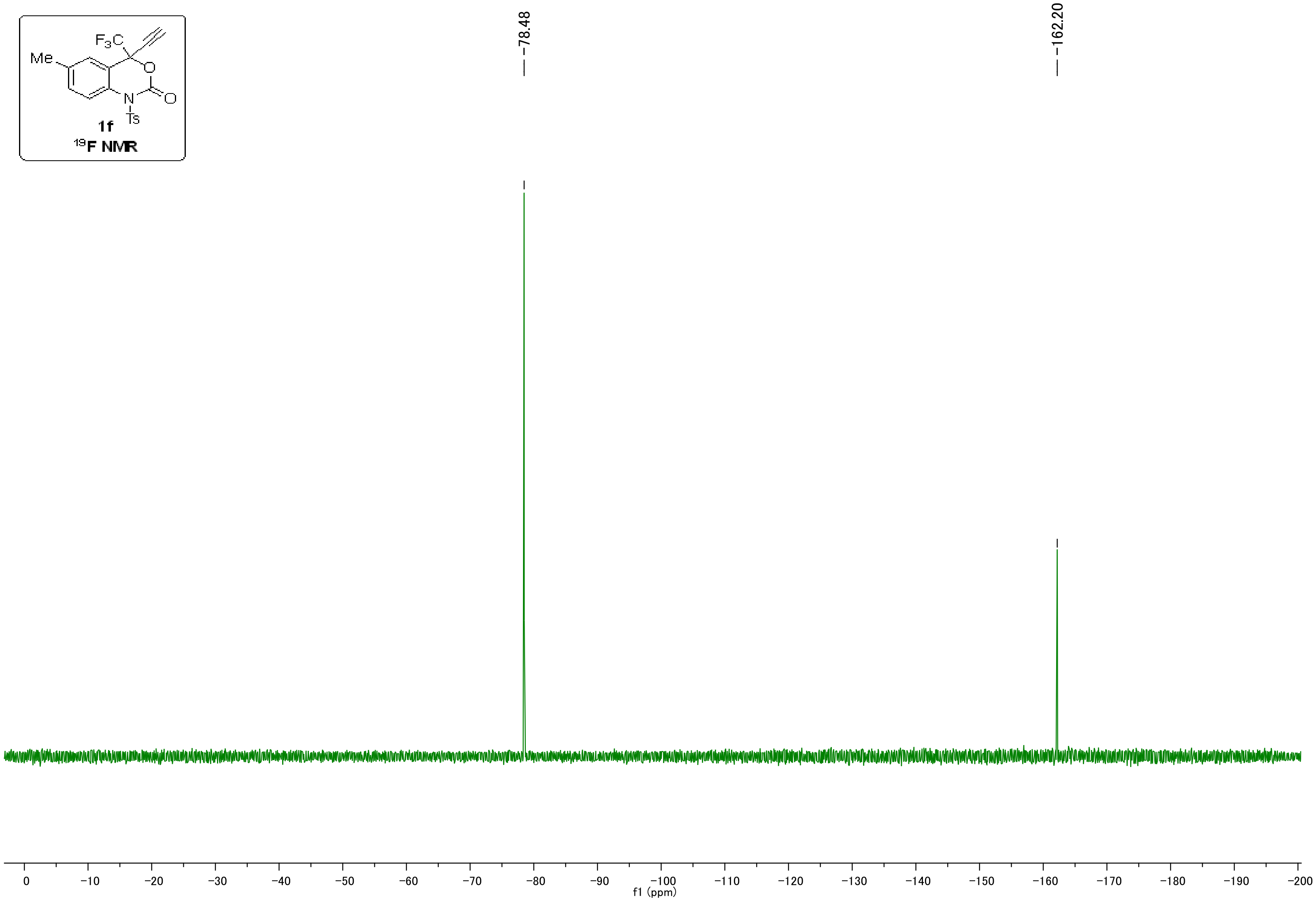

Supplementary Figure 36. ^{19}F NMR of **1f**.

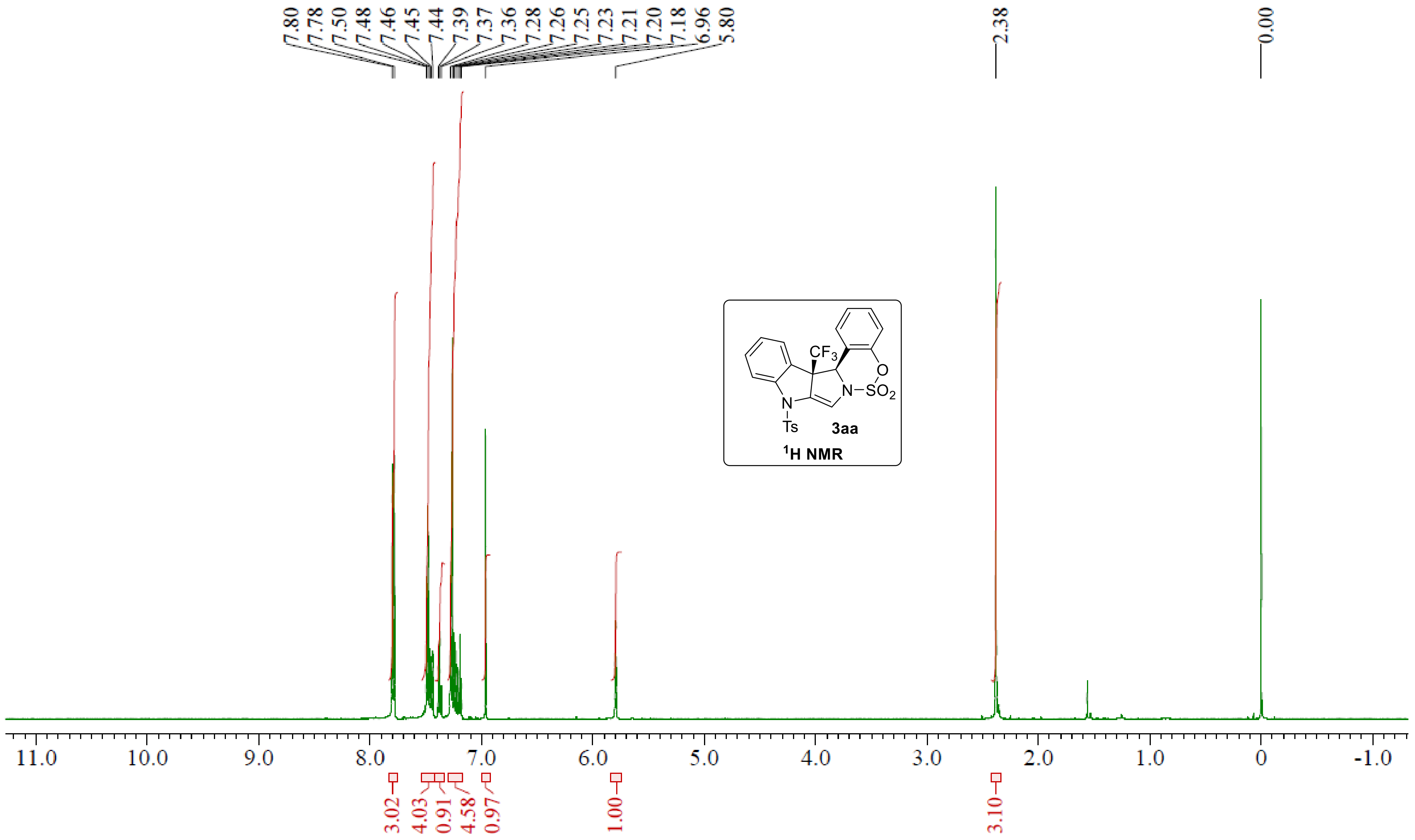

Supplementary Figure 37. ^{1}H NMR of **3aa**.

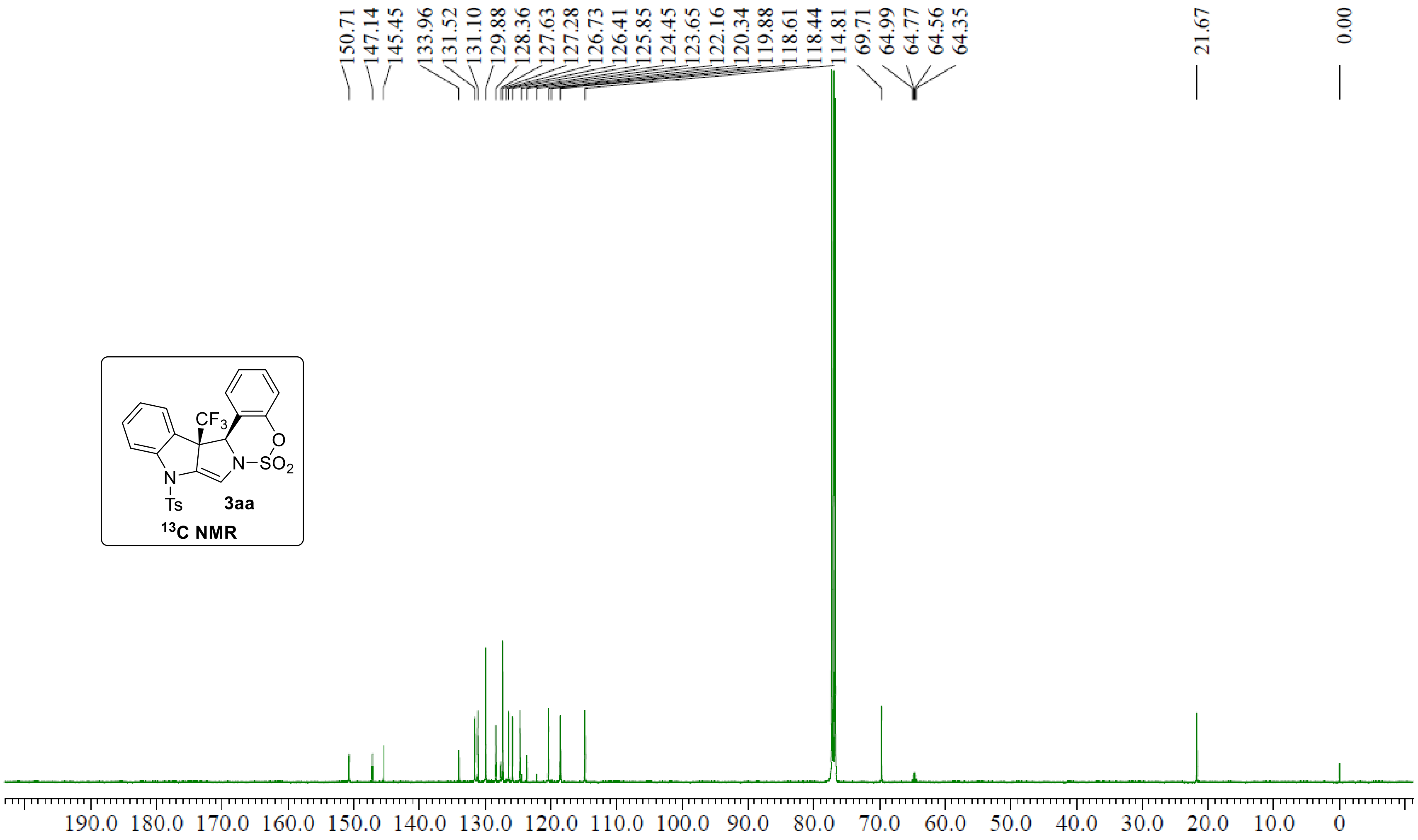

Supplementary Figure 38. ^{13}C NMR of **3aa**.

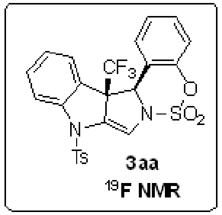

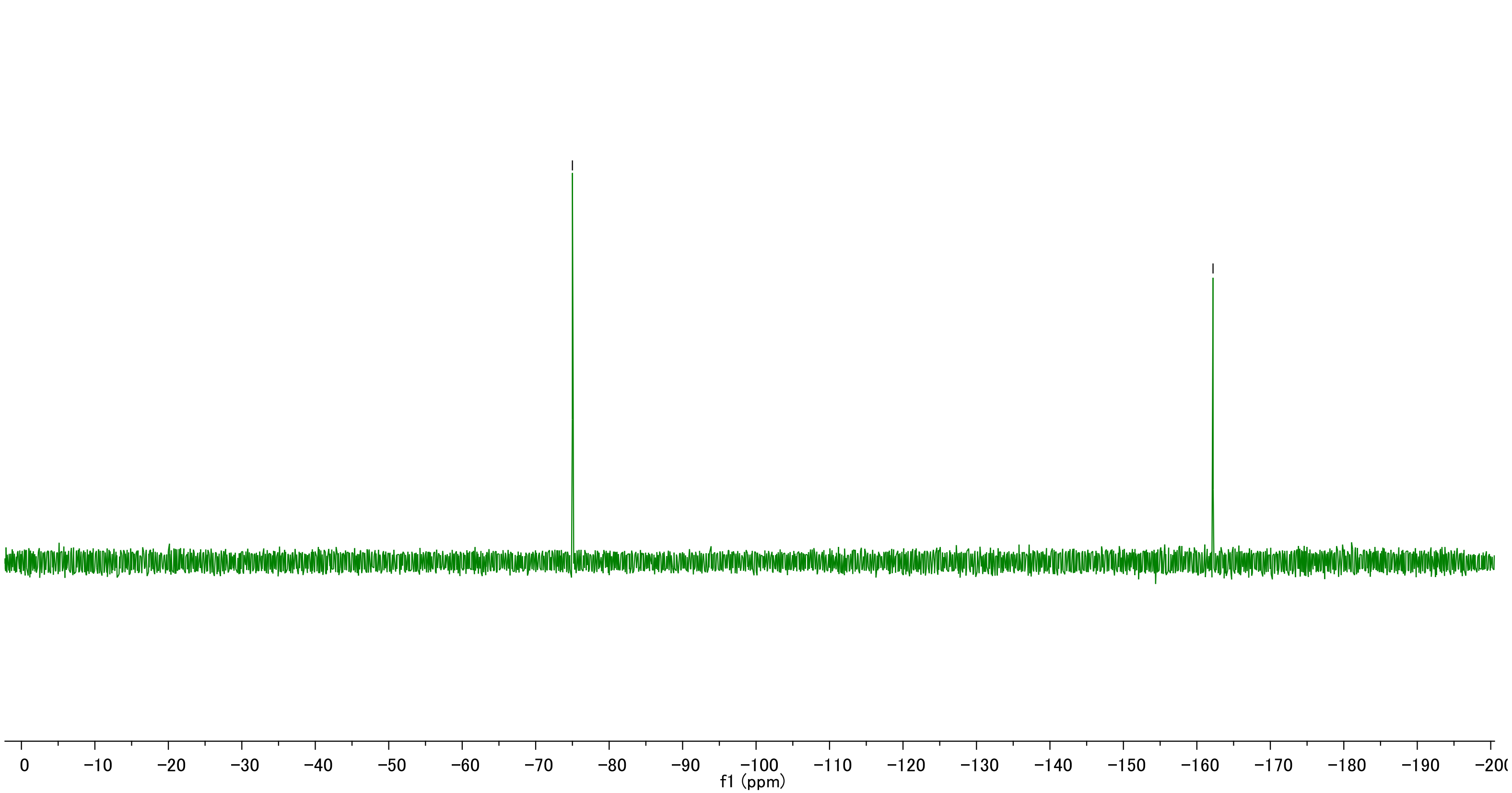

Supplementary Figure 39. ^{19}F NMR of **3aa**.

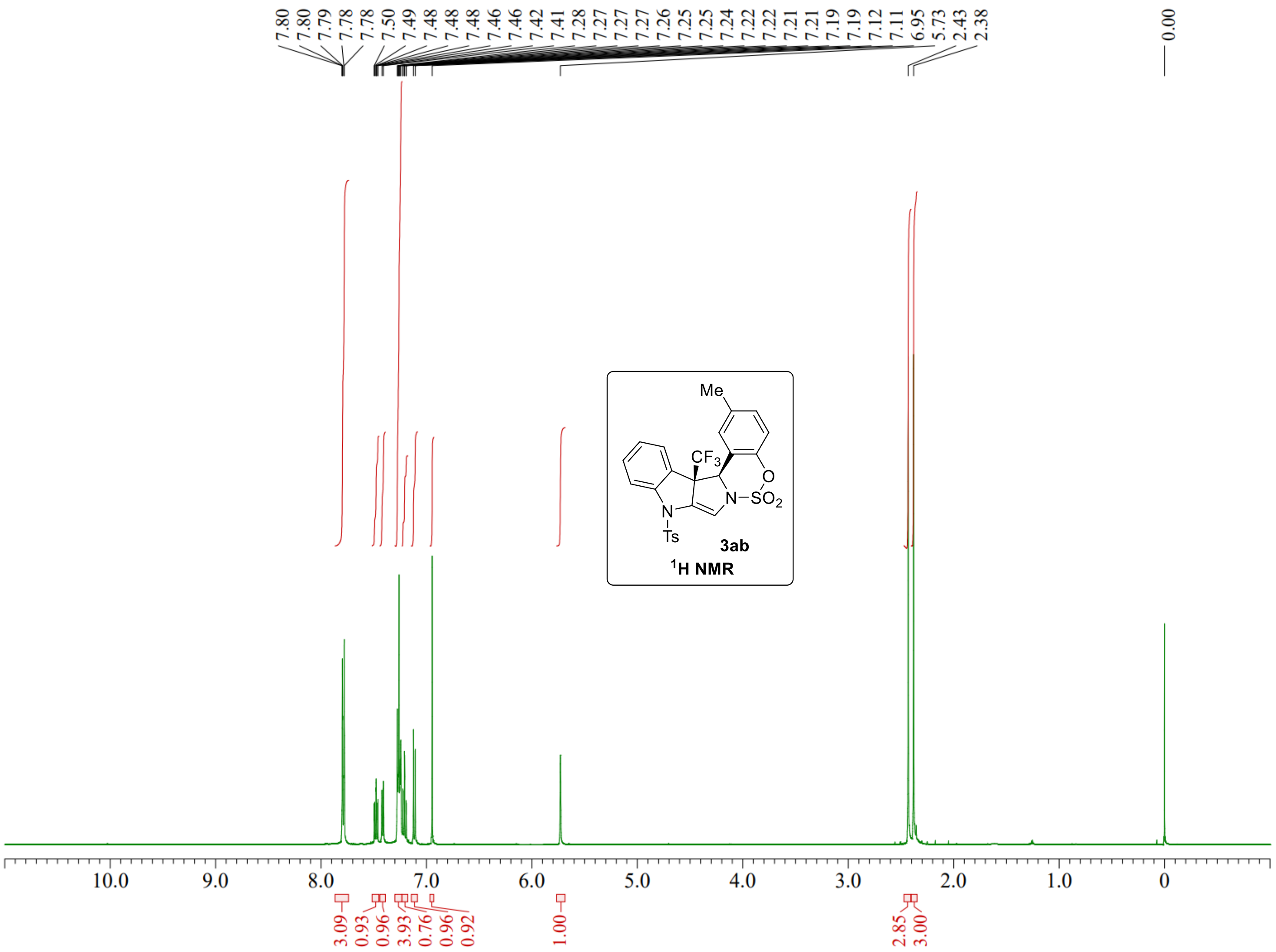

Supplementary Figure 40. ^{1}H NMR of **3ab**.

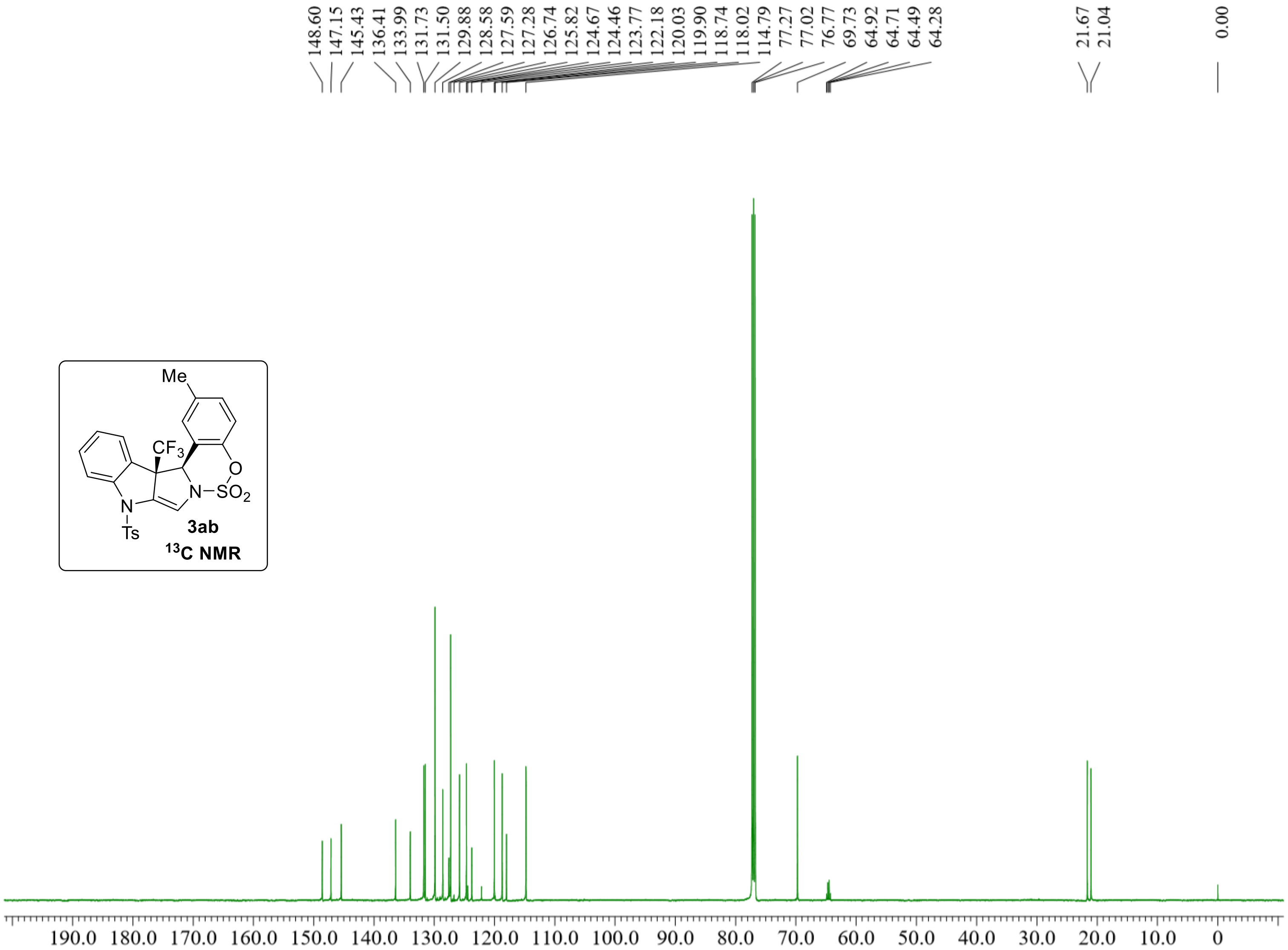

Supplementary Figure 41. ^{13}C NMR of **3ab**.

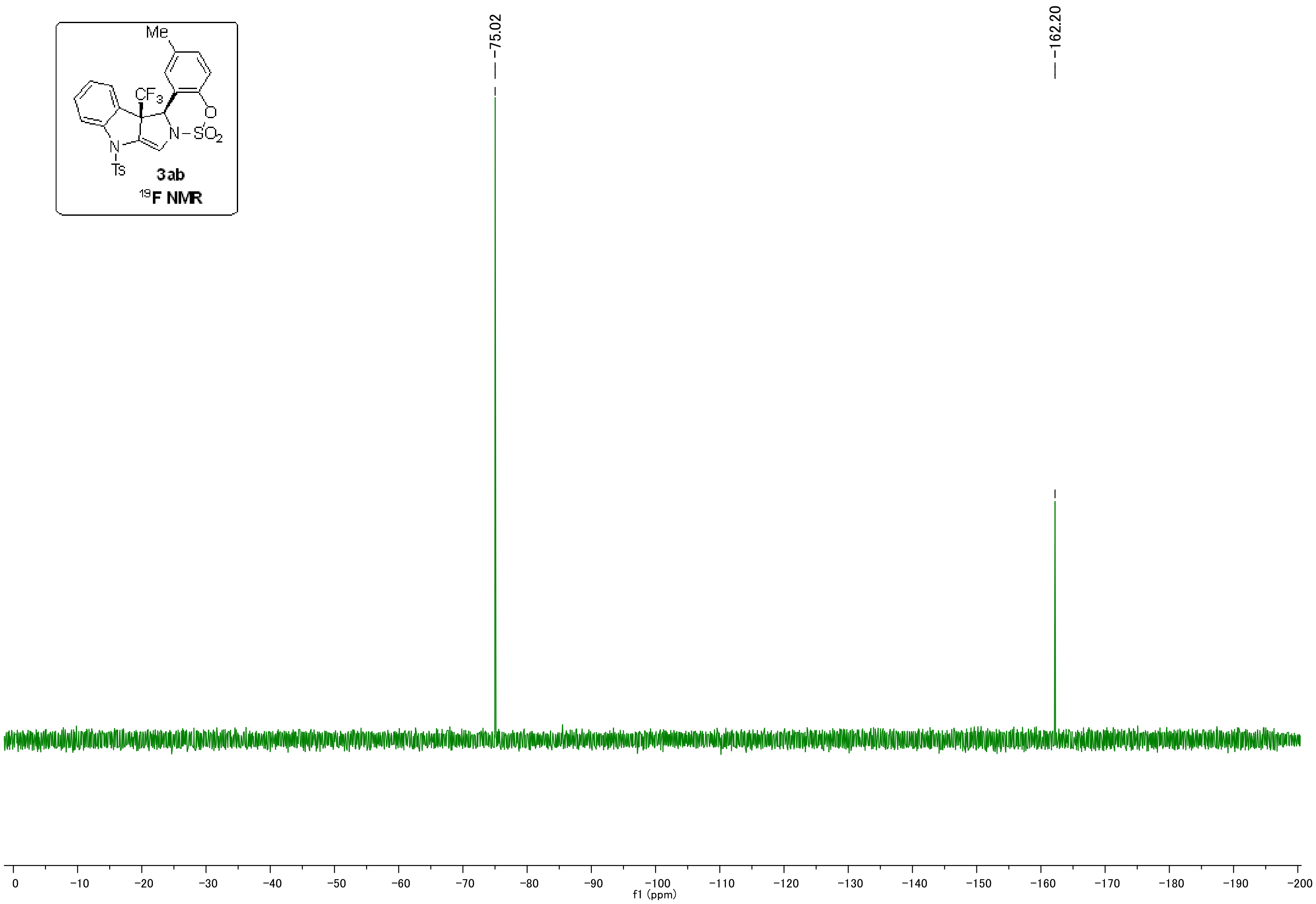

Supplementary Figure 42. ^{19}F NMR of **3ab**.

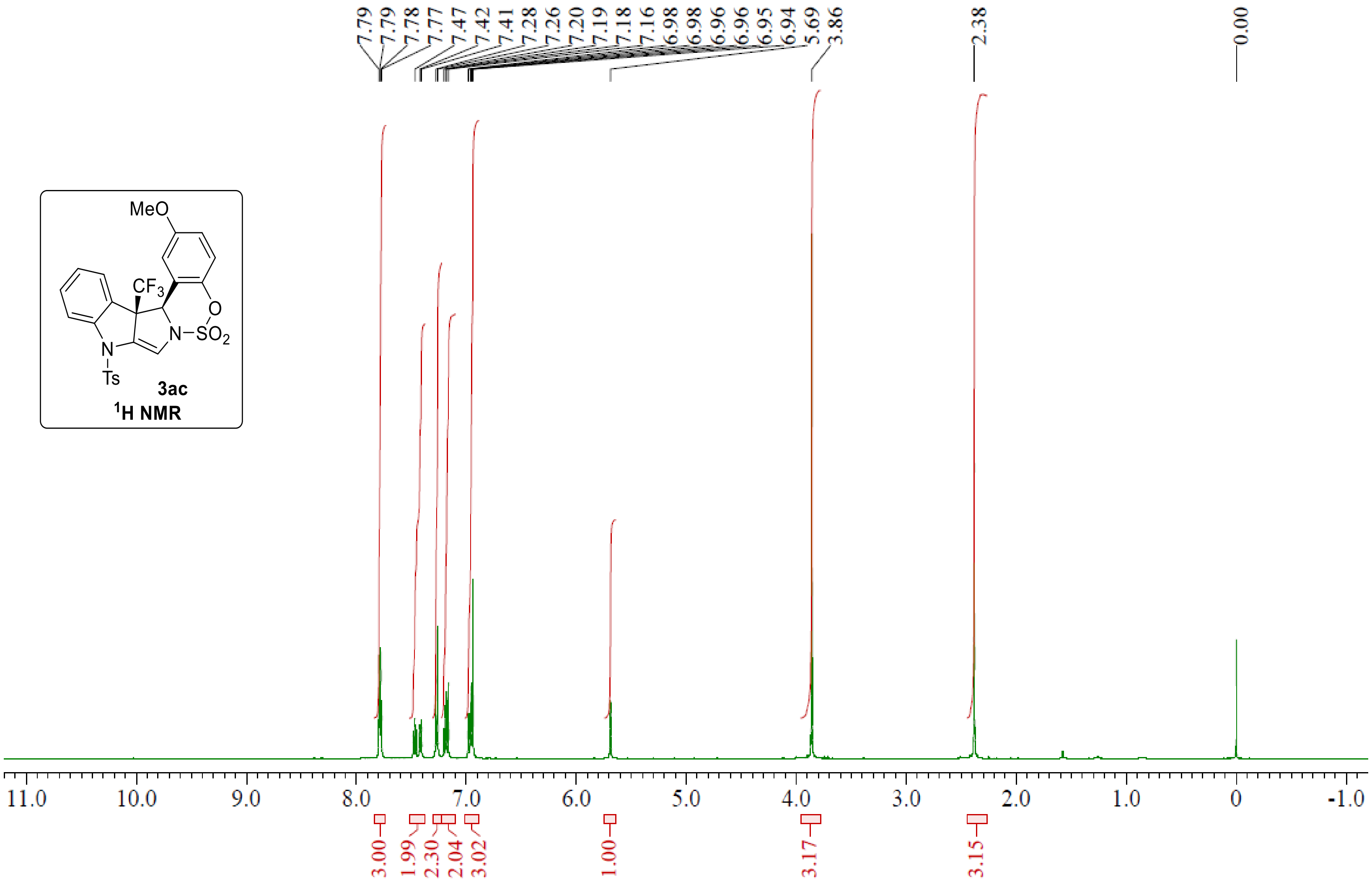

Supplementary Figure 43. ^{1}H NMR of **3ac**.

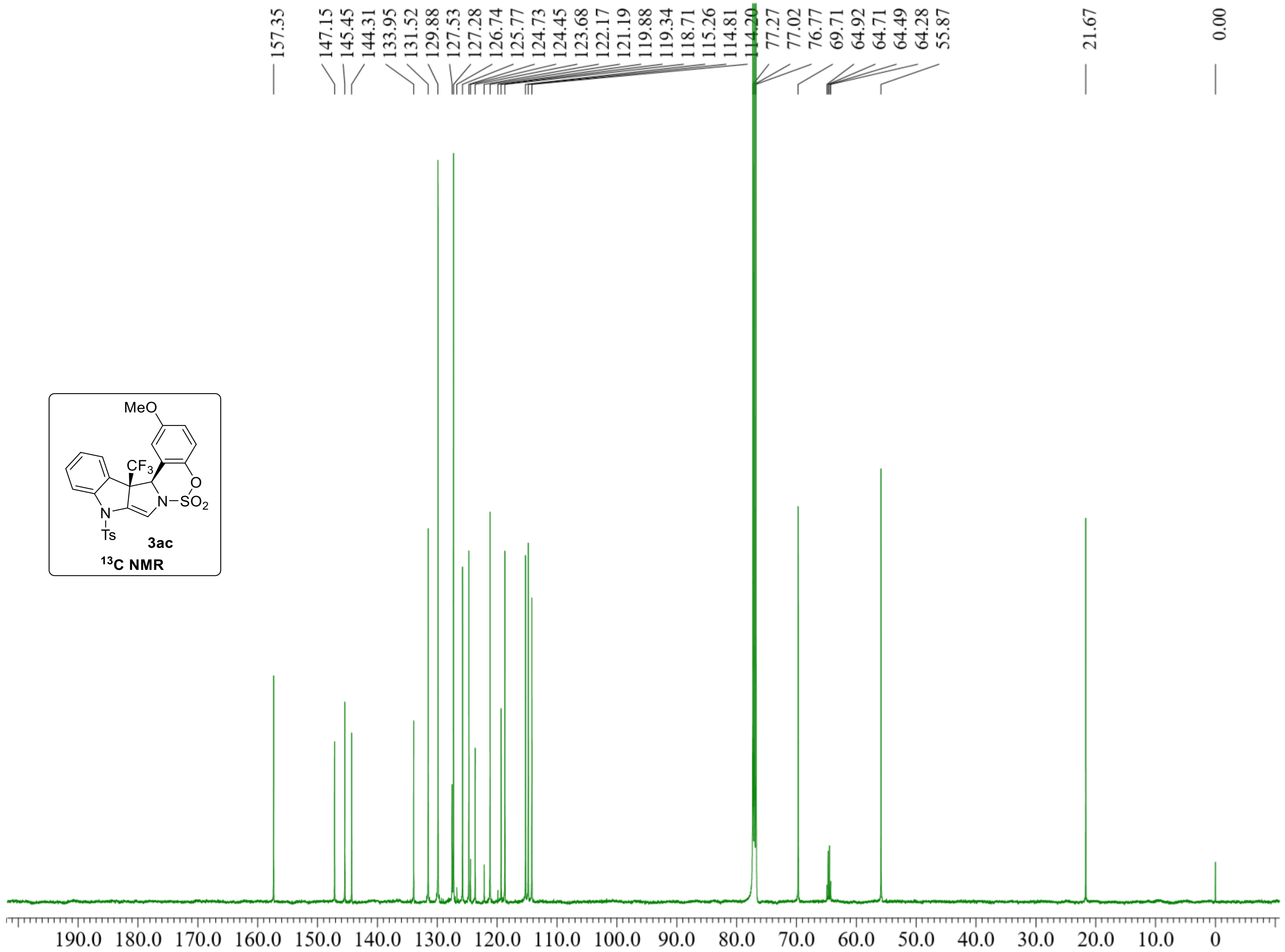

Supplementary Figure 44. ^{13}C NMR of **3ac**.

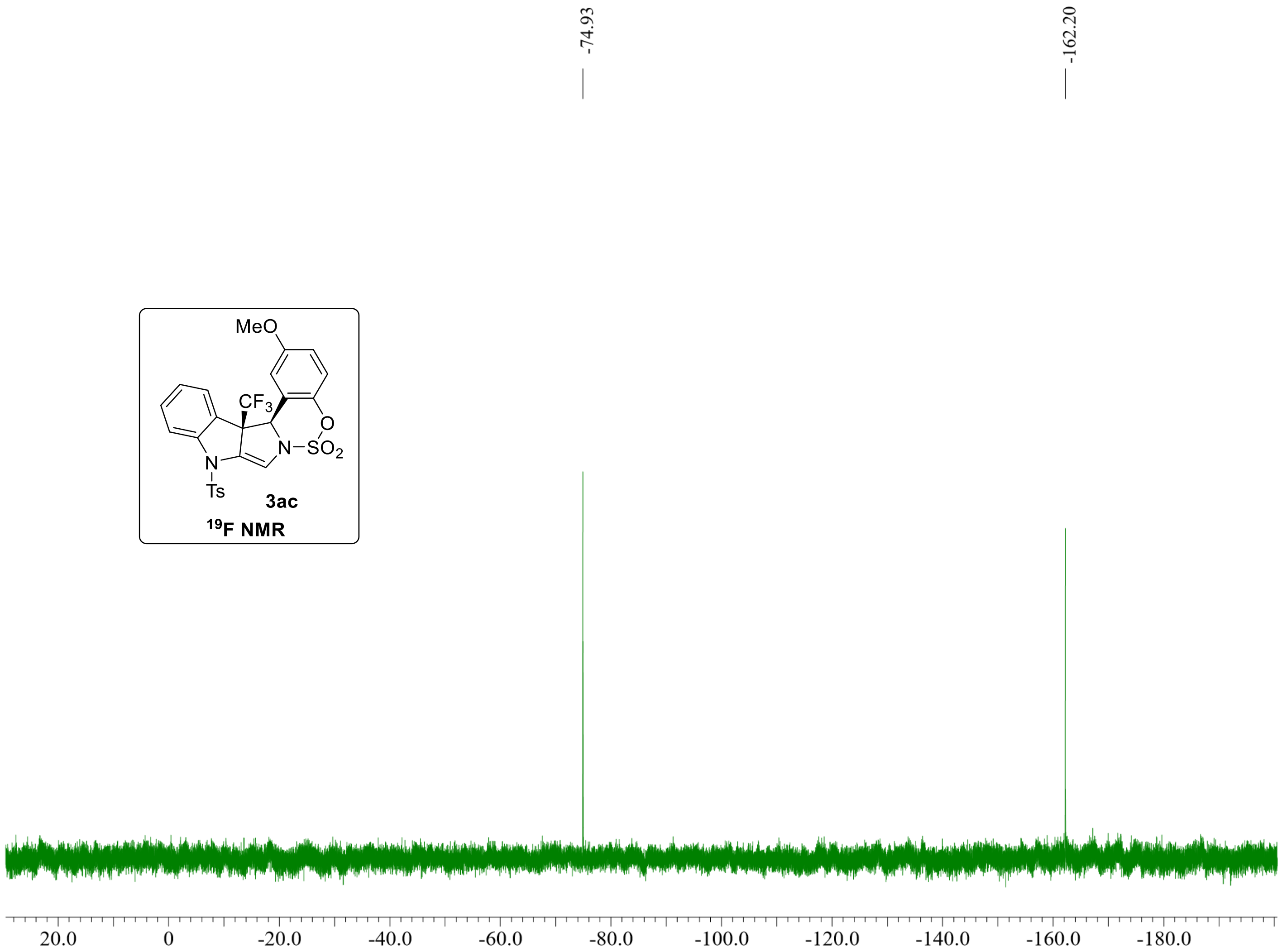

Supplementary Figure 45. ^{19}F NMR of **3ac**.

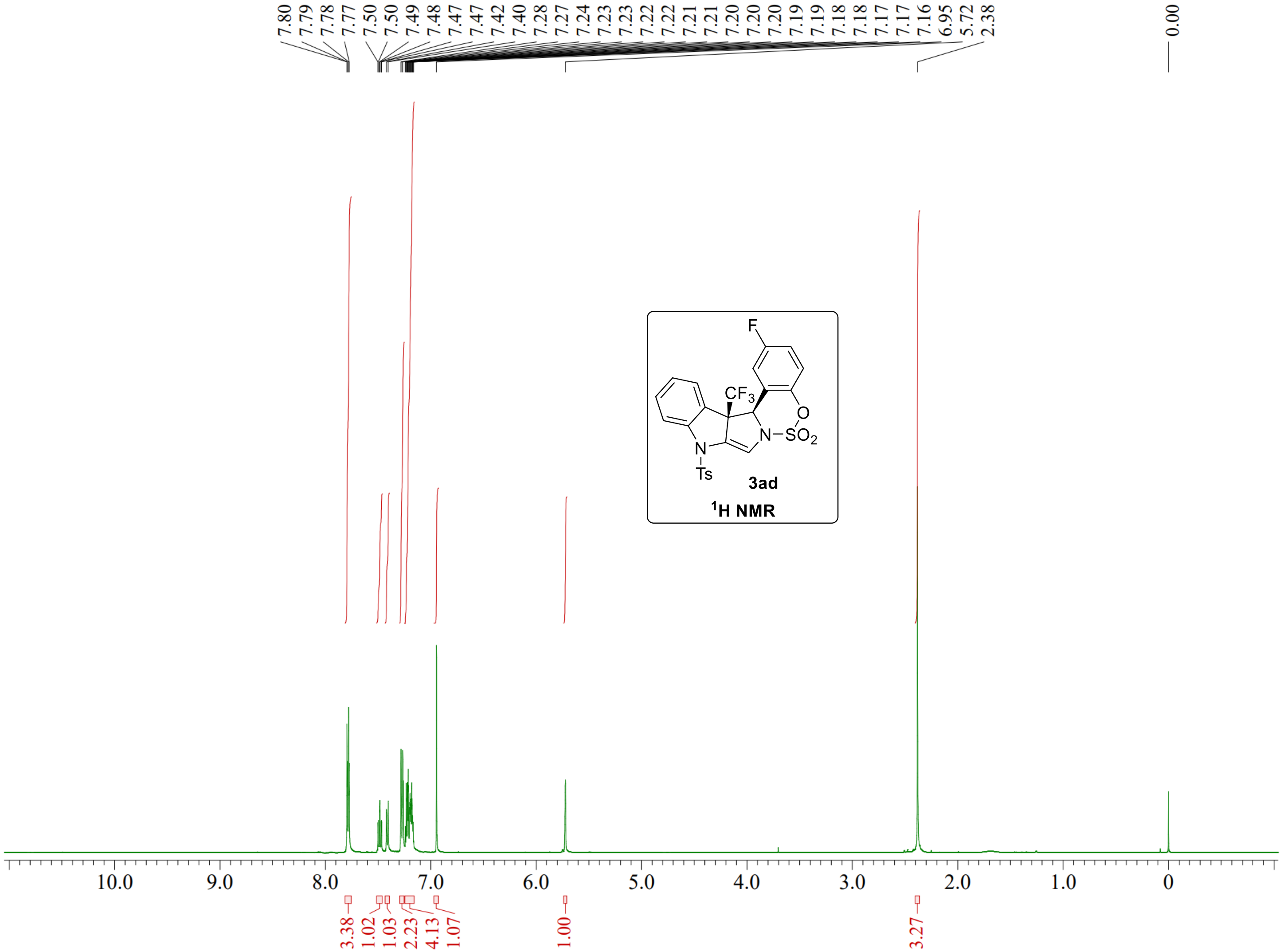

Supplementary Figure 46. ^{1}H NMR of **3ad**.

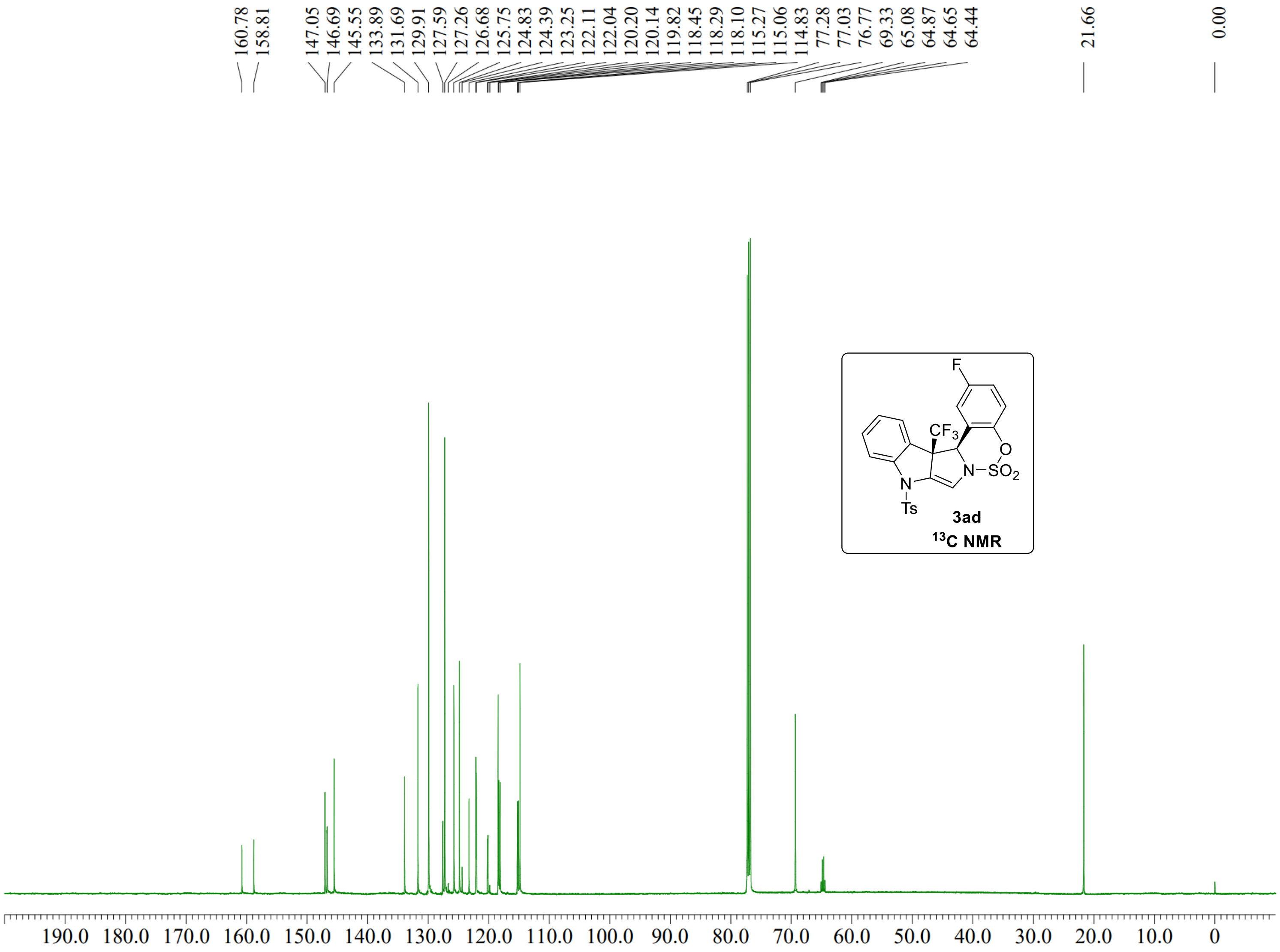

Supplementary Figure 47. ^{13}C NMR of **3ad**.

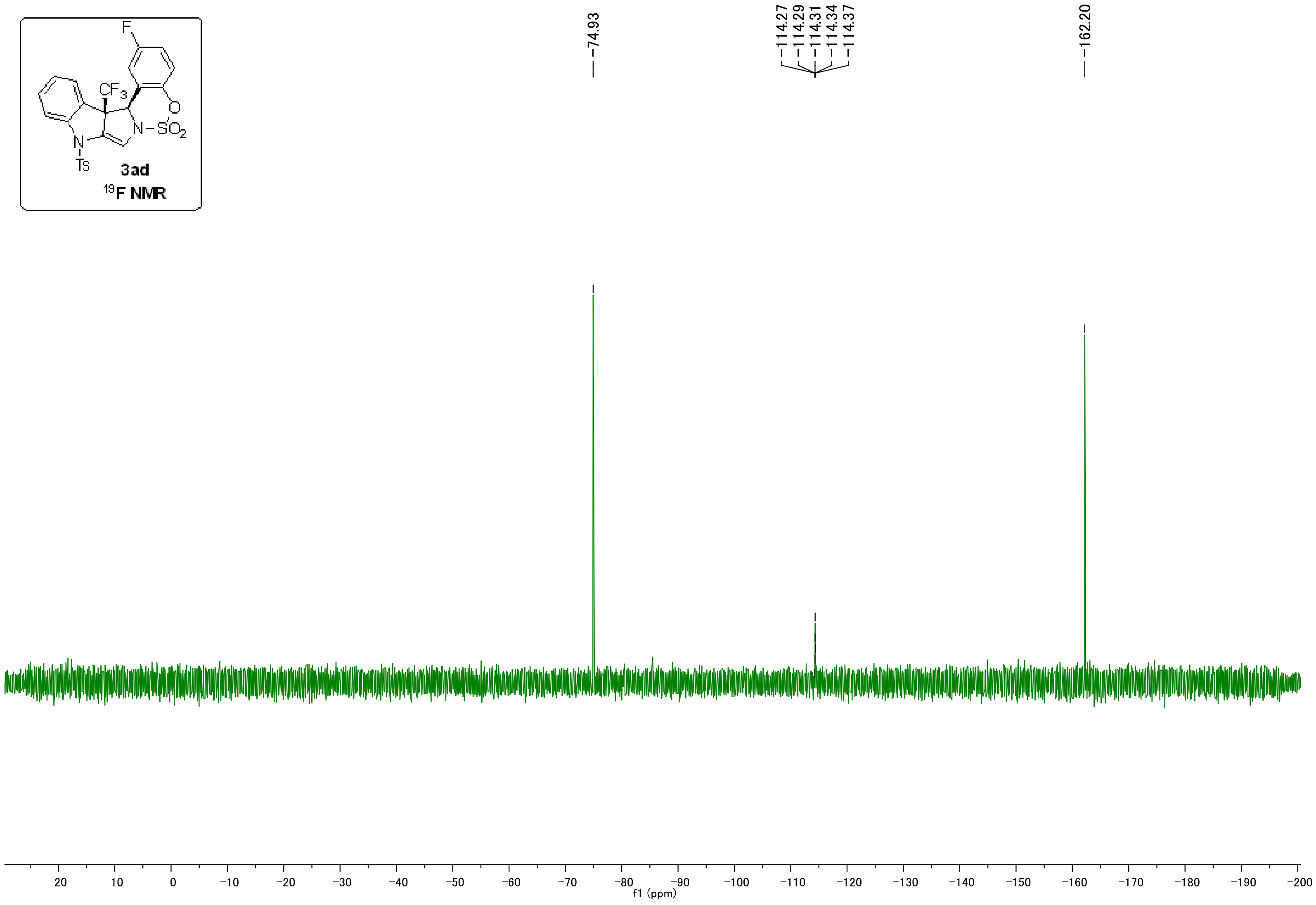

Supplementary Figure 48. ^{19}F NMR of **3ad**.

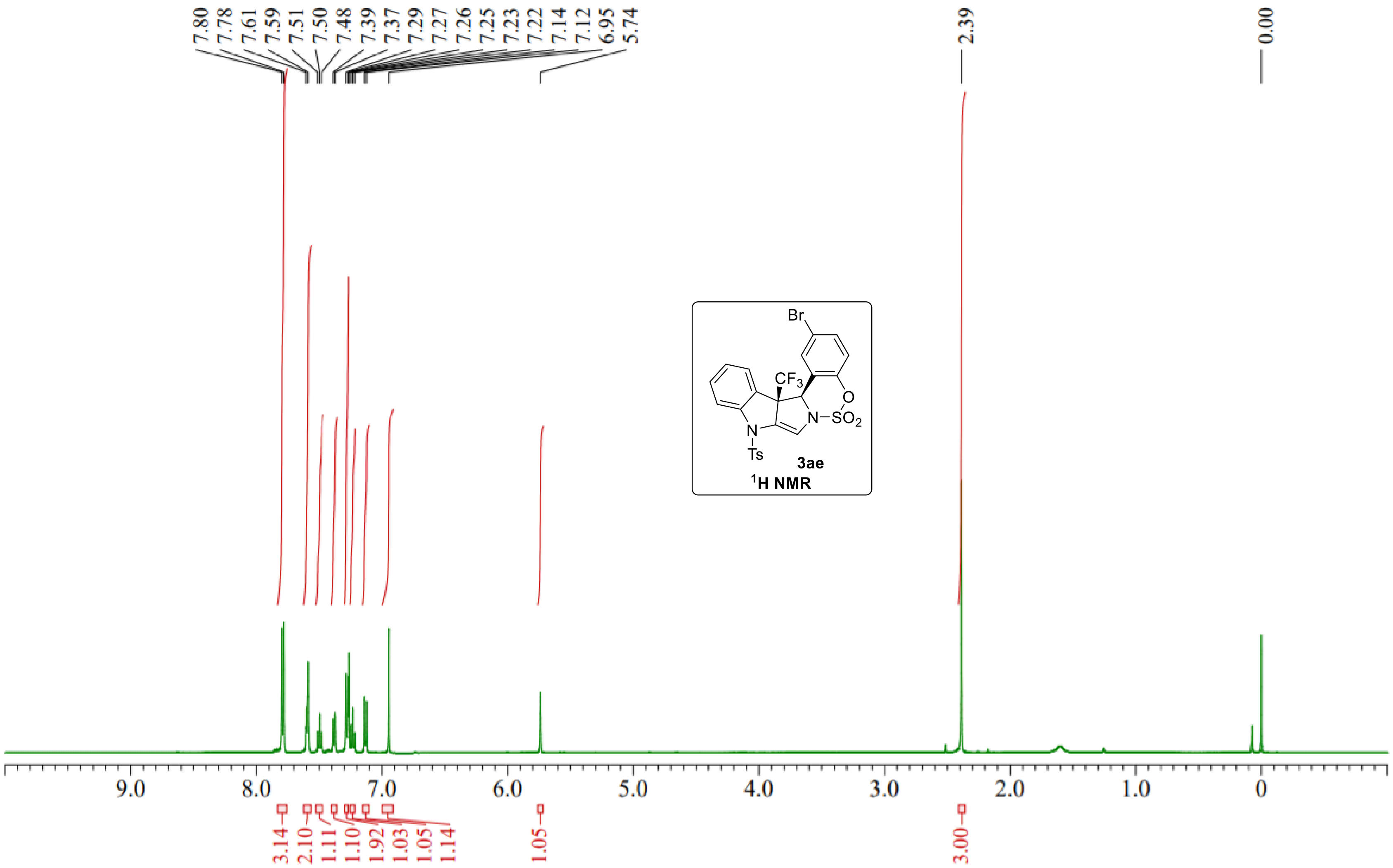

Supplementary Figure 49. ^{1}H NMR of **3ae**.

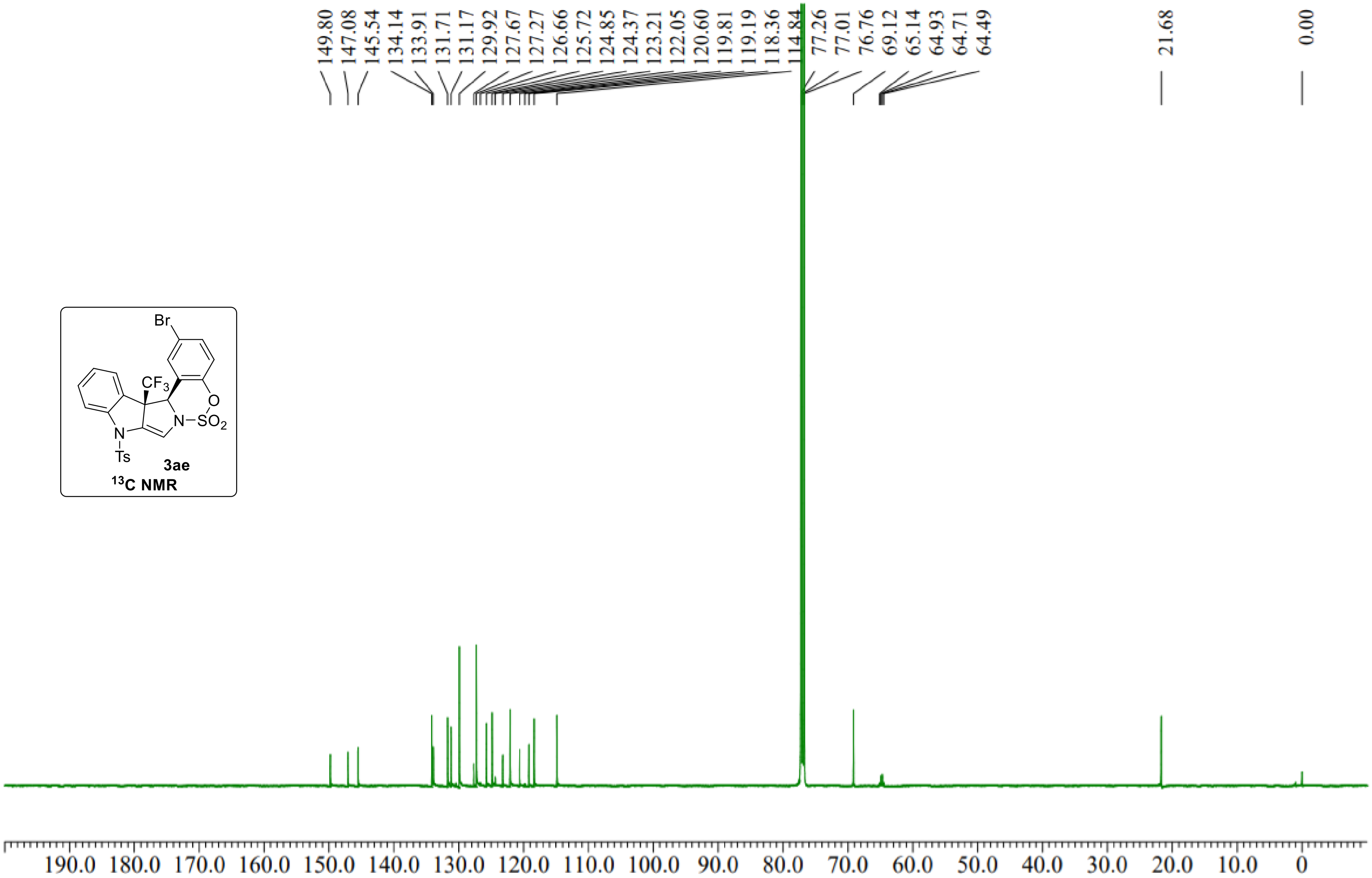

Supplementary Figure 50. ^{13}C NMR of **3ae**.

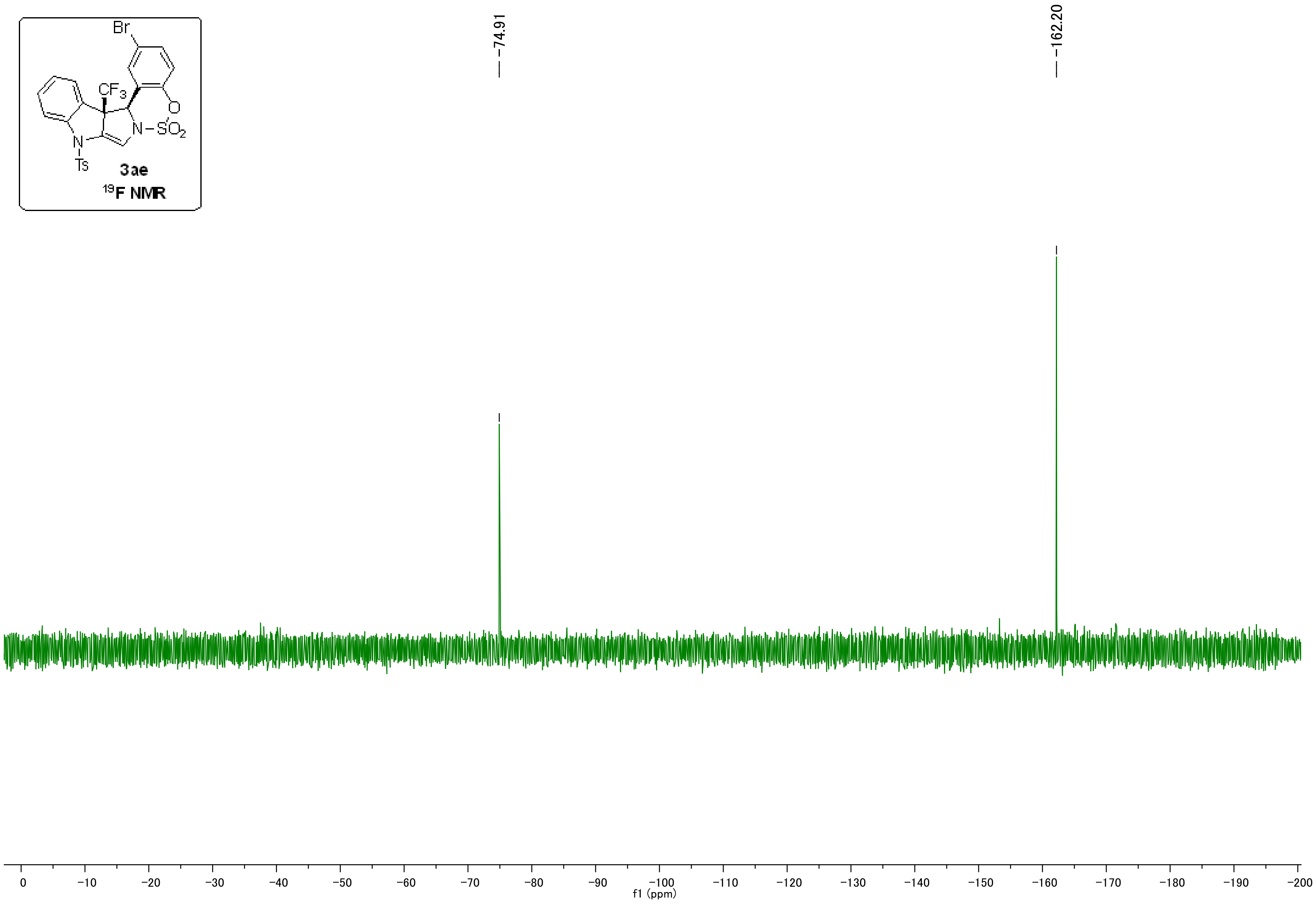

Supplementary Figure 51. ^{19}F NMR of **3ae**.

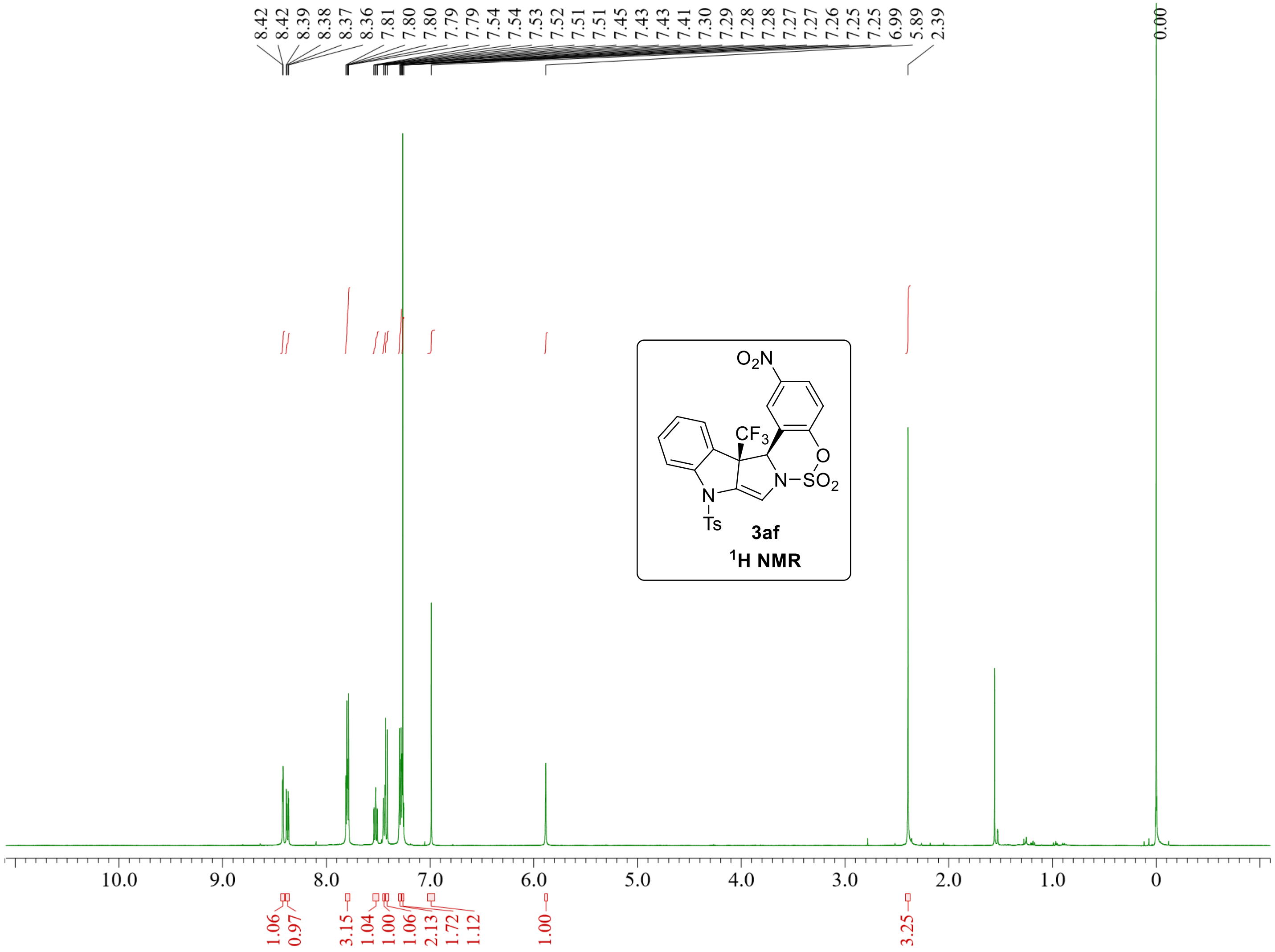

Supplementary Figure 52. ^{1}H NMR of **3af**.

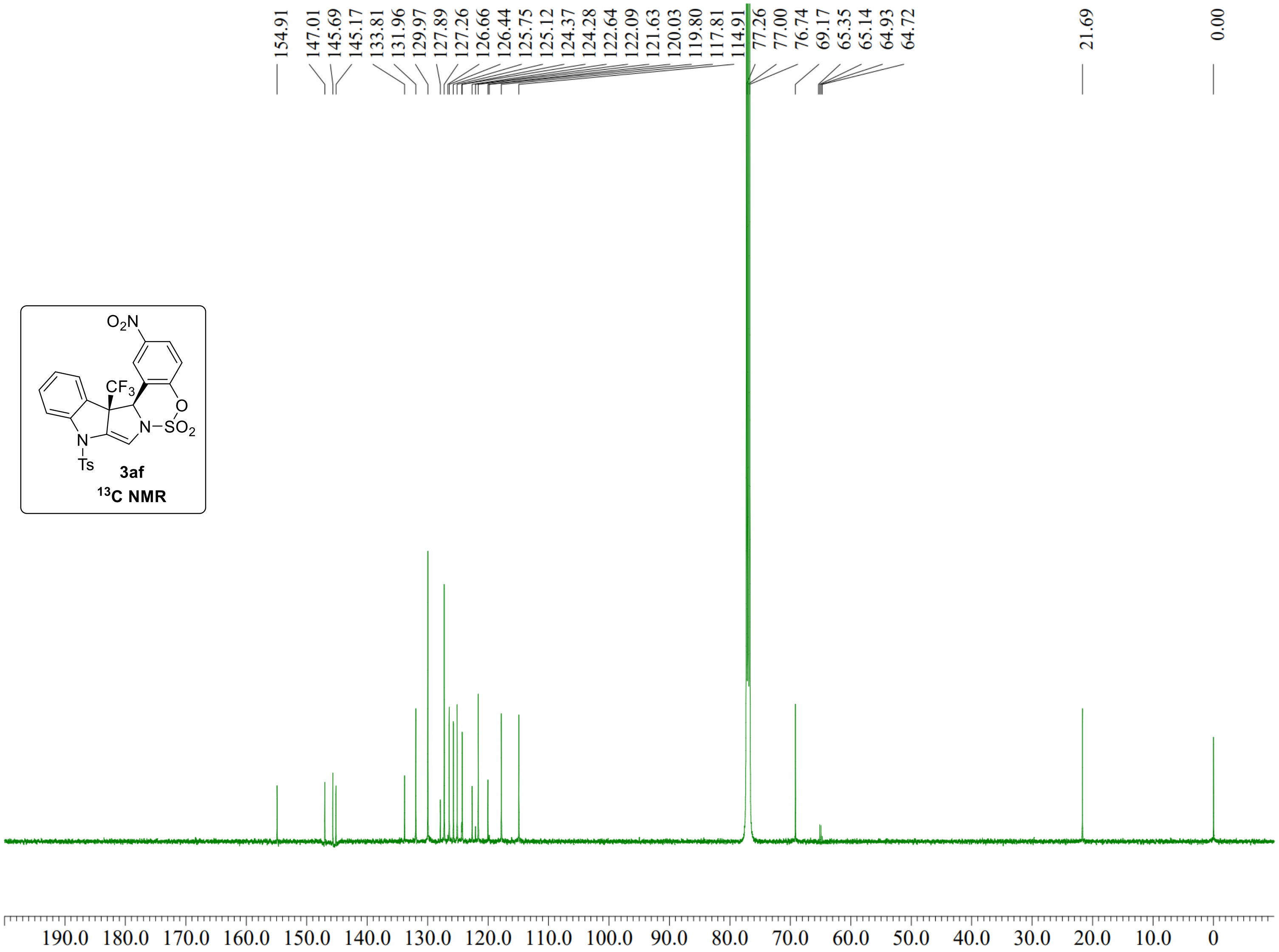

Supplementary Figure 53. ^{13}C NMR of **3af**.

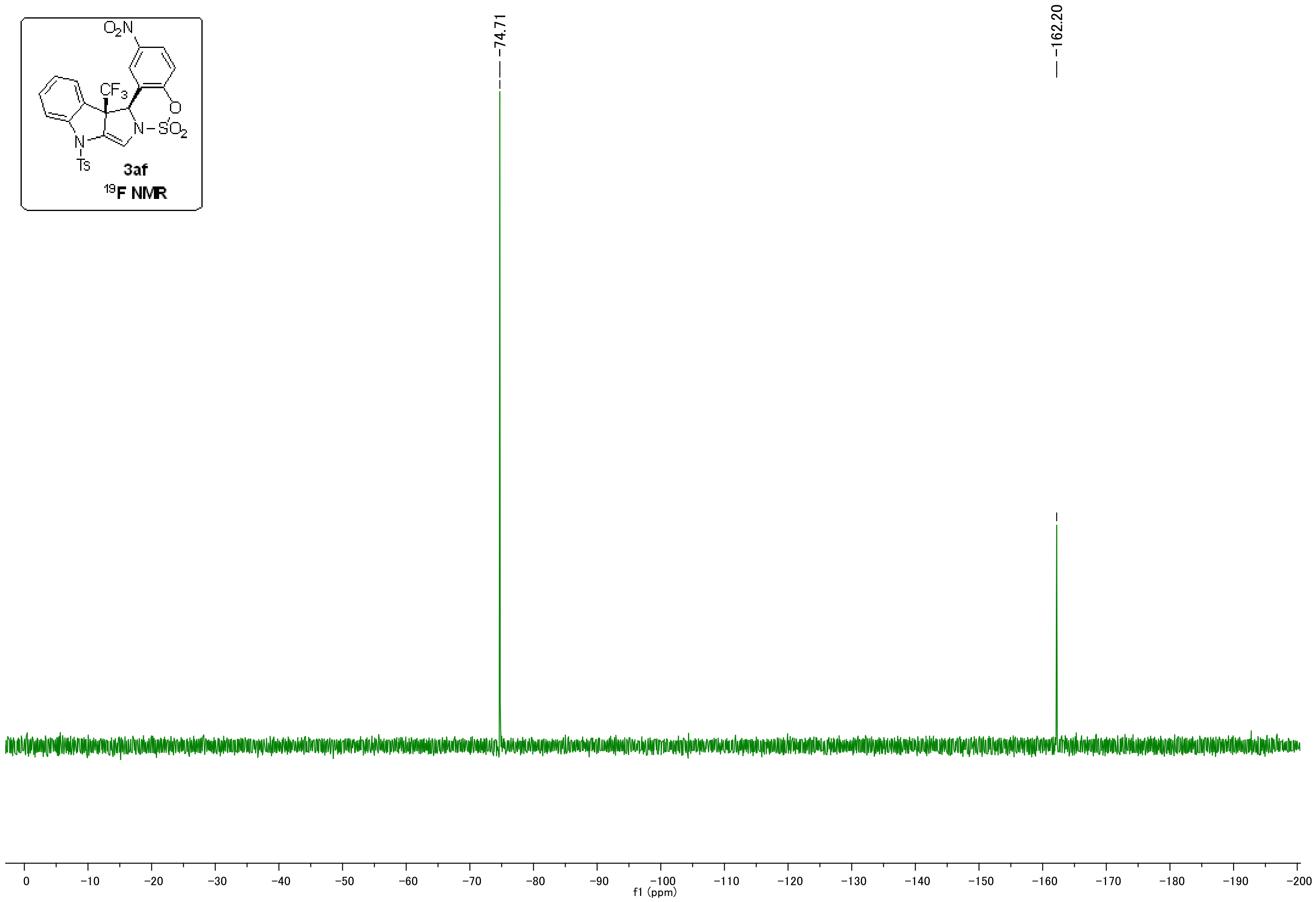

Supplementary Figure 54. ^{19}F NMR of **3af**.

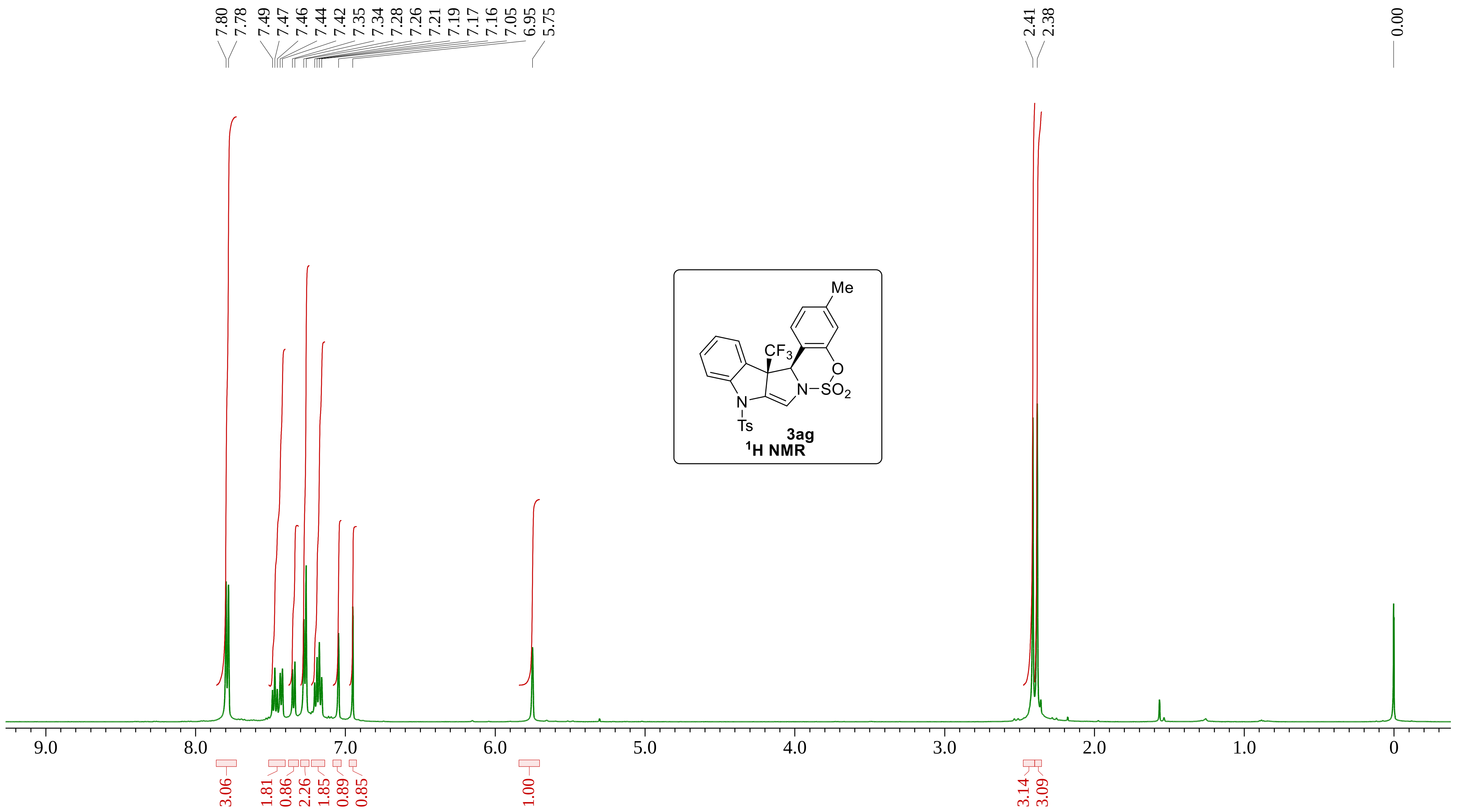

Supplementary Figure 55. ^{1}H NMR of **3ag**.

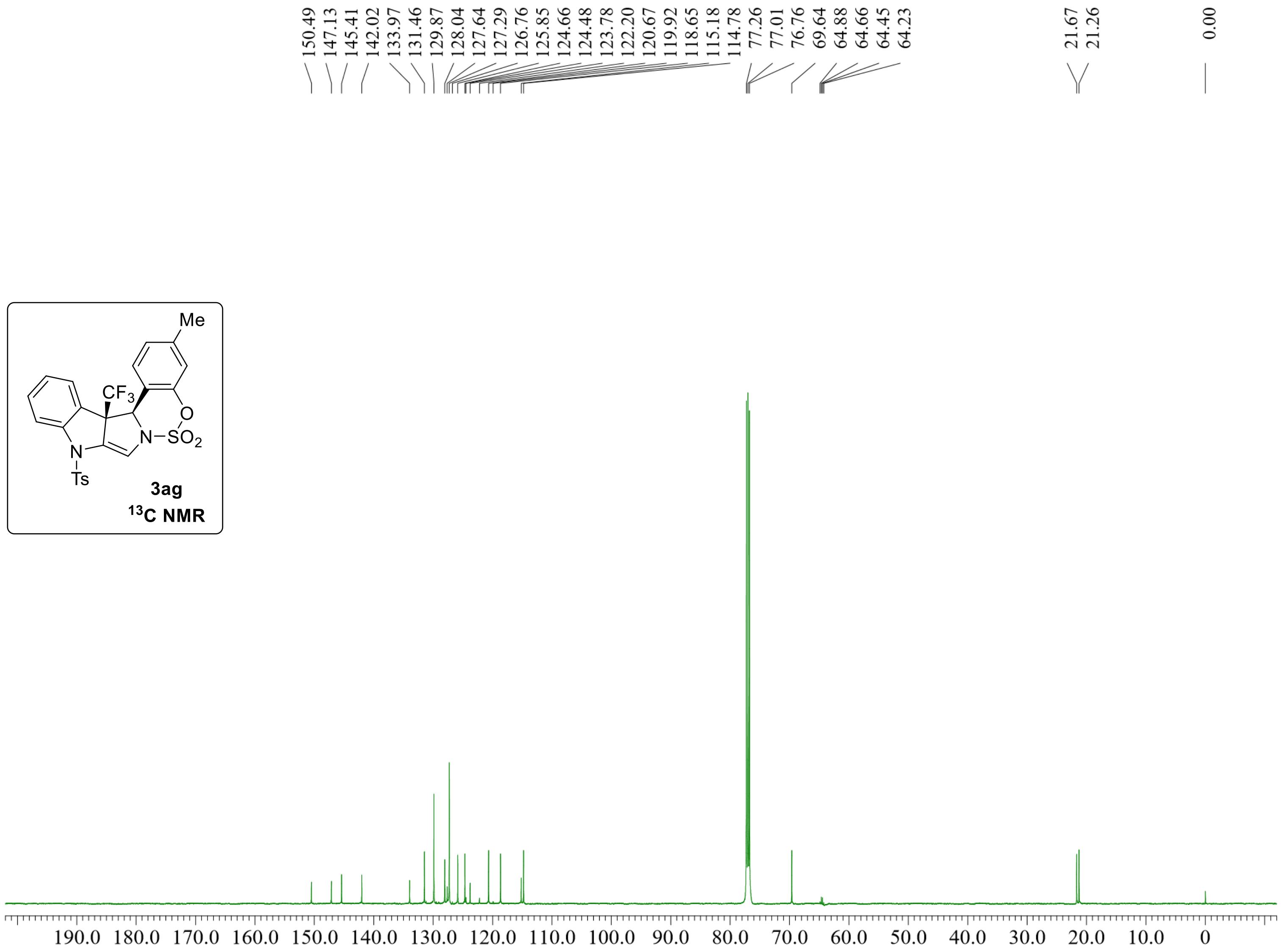

Supplementary Figure 56. ^{13}C NMR of **3ag**.

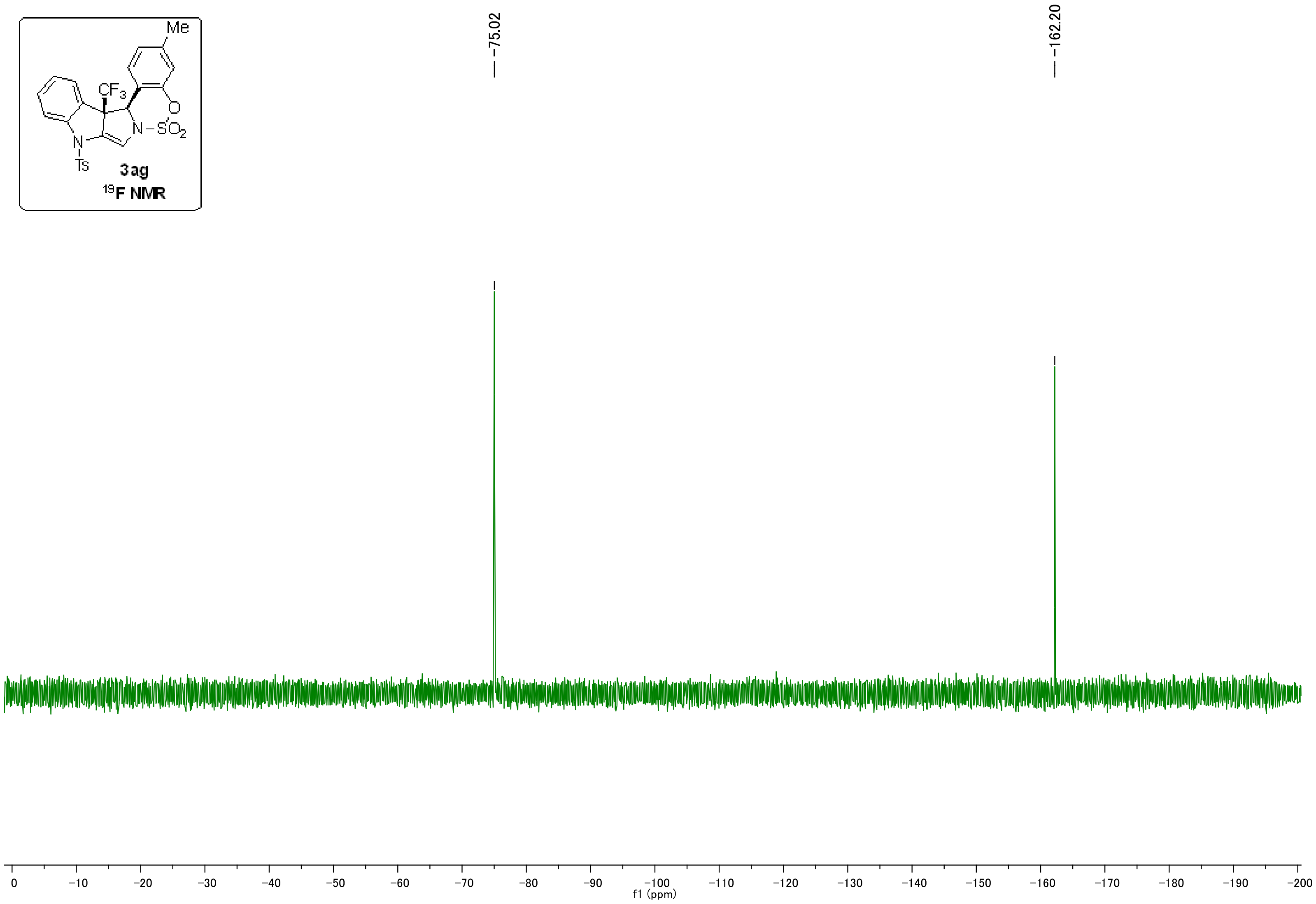

Supplementary Figure 57. ^{19}F NMR of **3ag**.

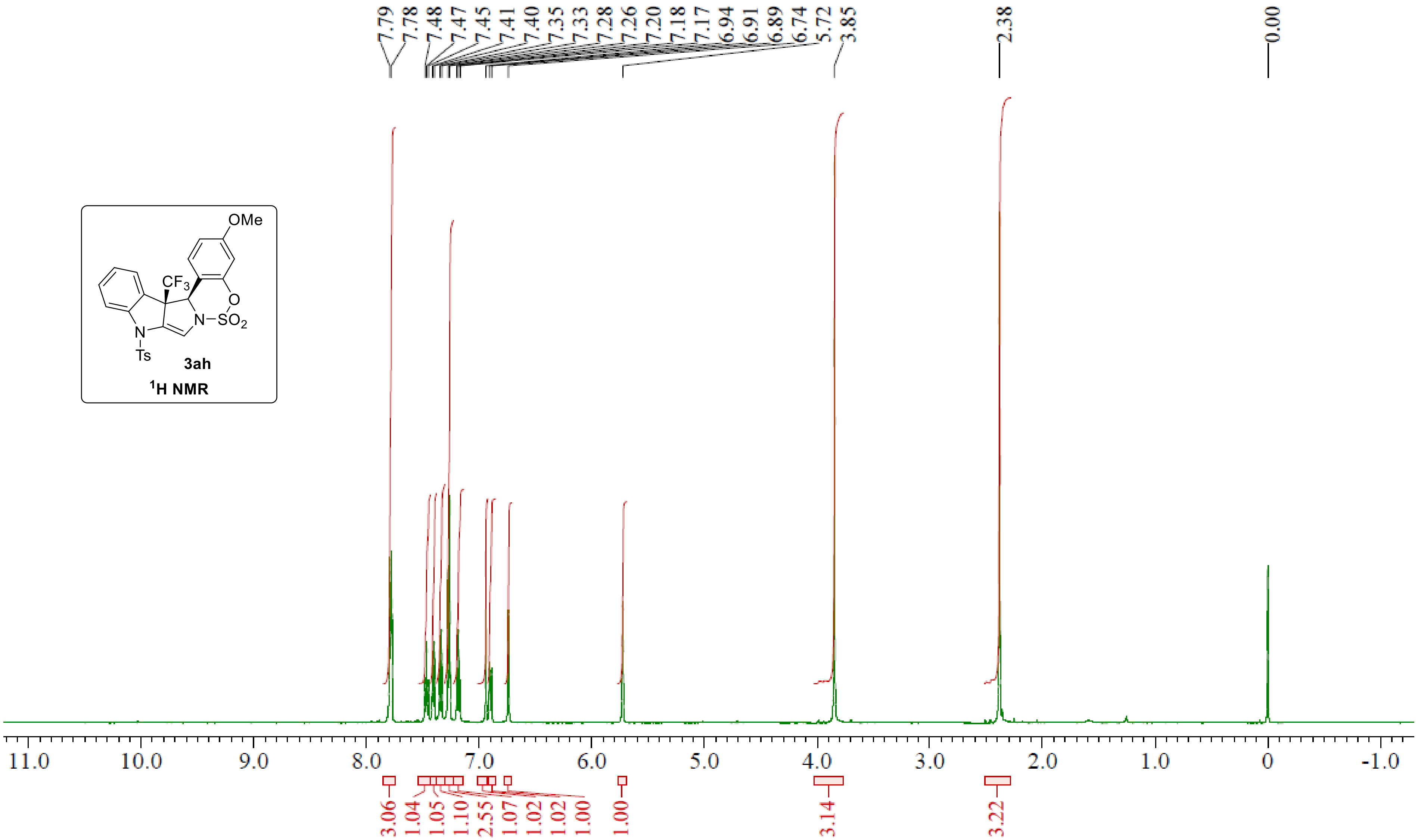

Supplementary Figure 58. ^{1}H NMR of **3ah**.

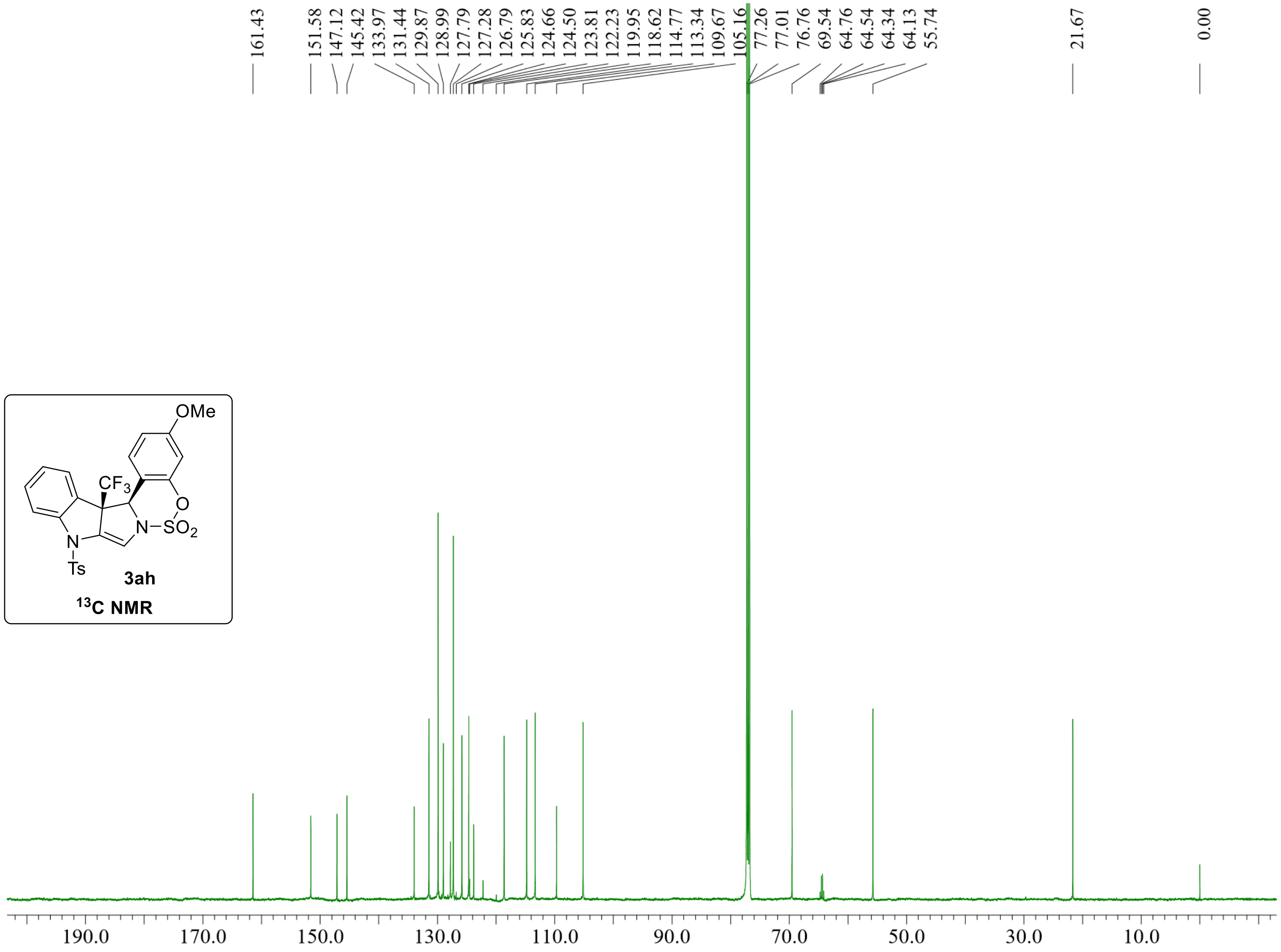

Supplementary Figure 59. ^{13}C NMR of **3ah**.

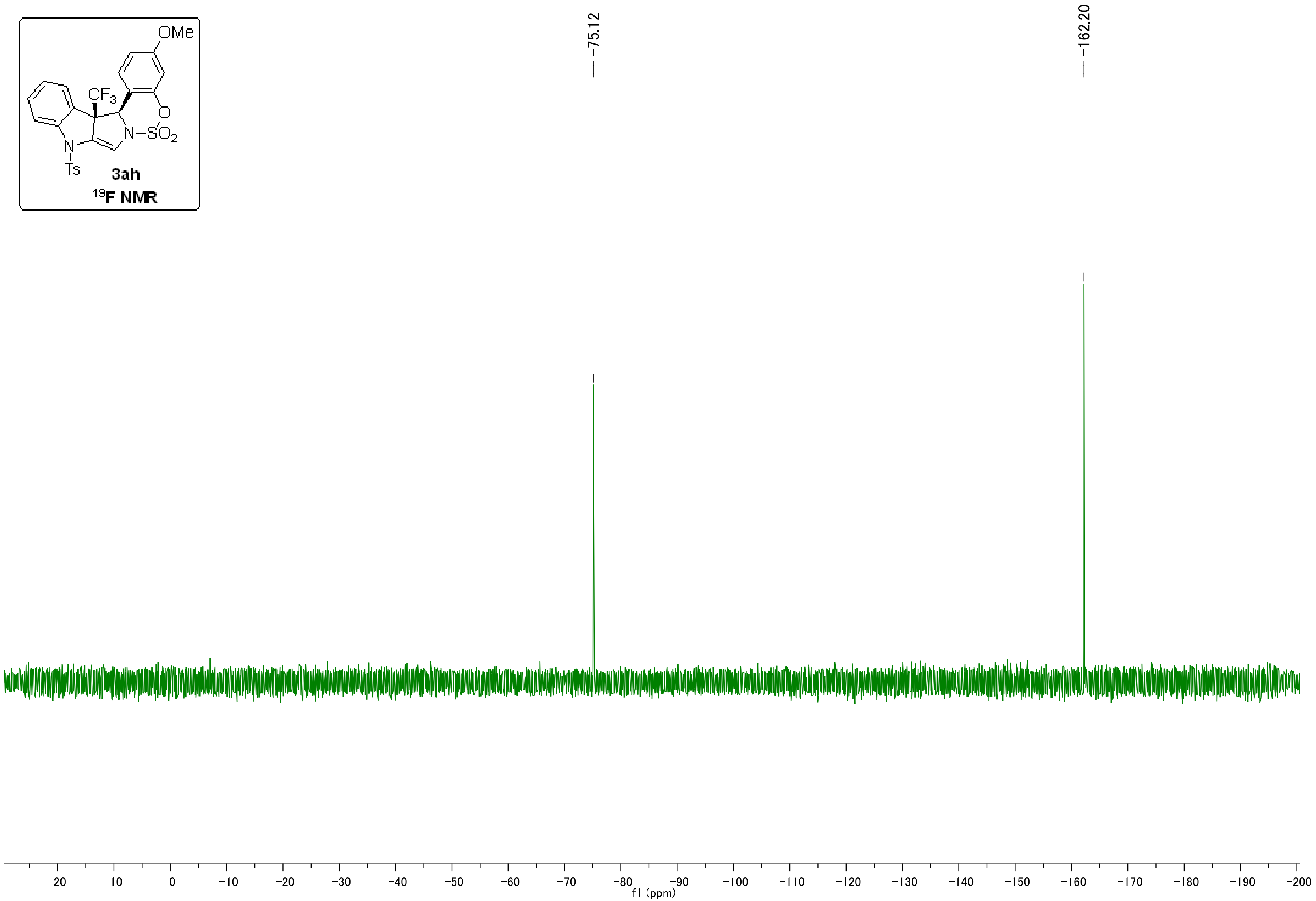

Supplementary Figure 60. ^{19}F NMR of **3ah**.

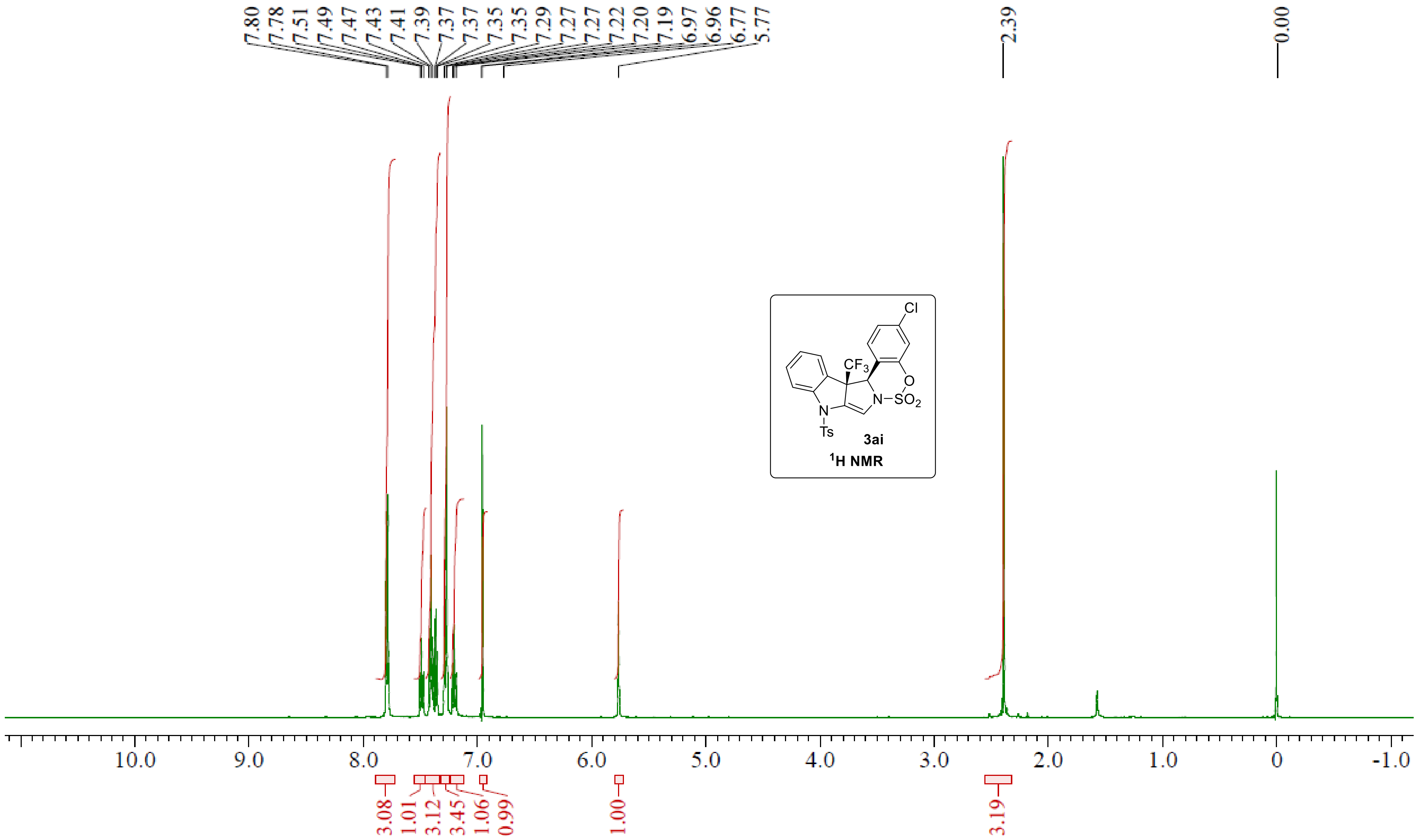

Supplementary Figure 61. ^{1}H NMR of **3ai**.

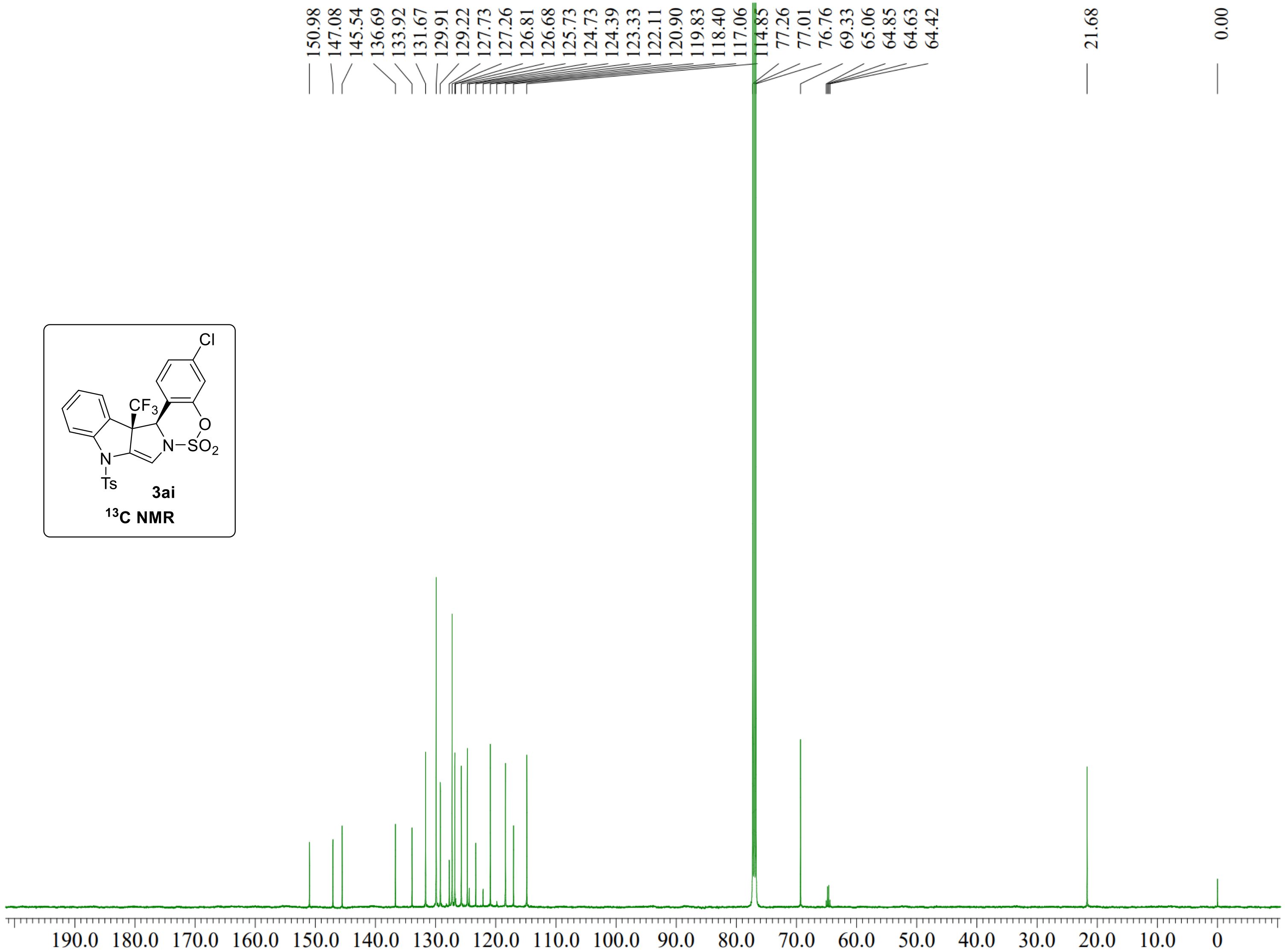

Supplementary Figure 62. ^{13}C NMR of **3ai**.

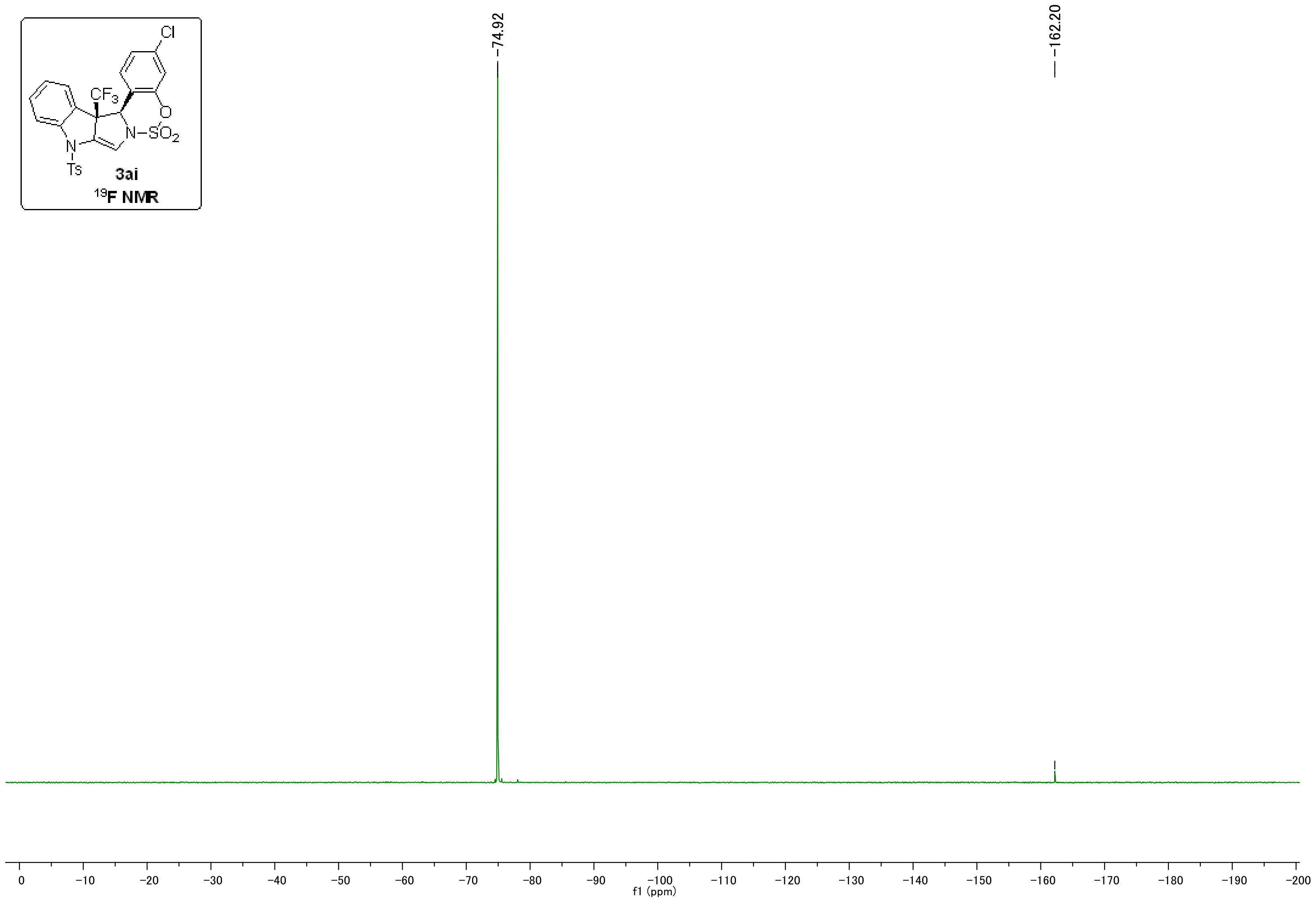

Supplementary Figure 63. ^{19}F NMR of **3ai**.

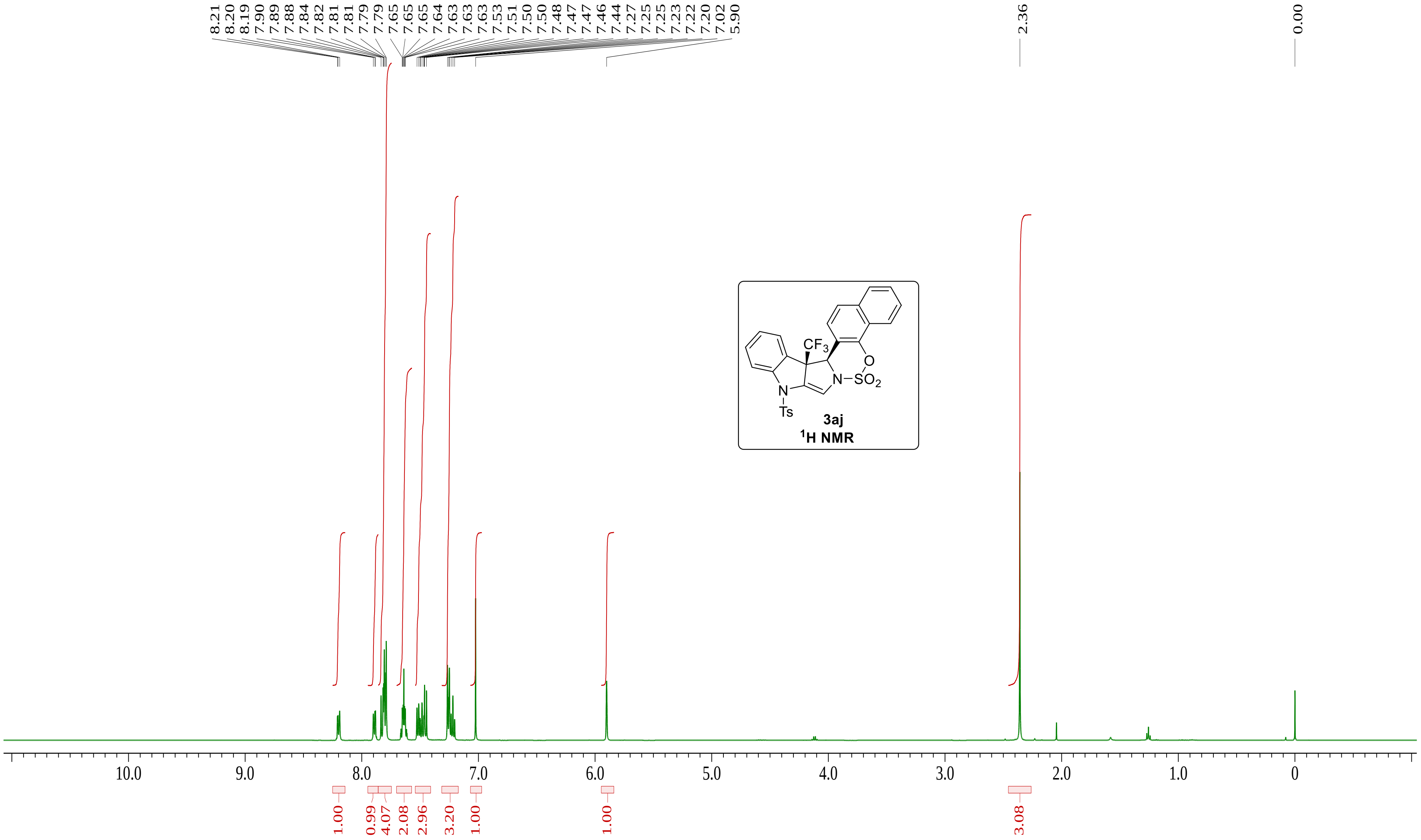

Supplementary Figure 64. ^{1}H NMR of **3aj**.

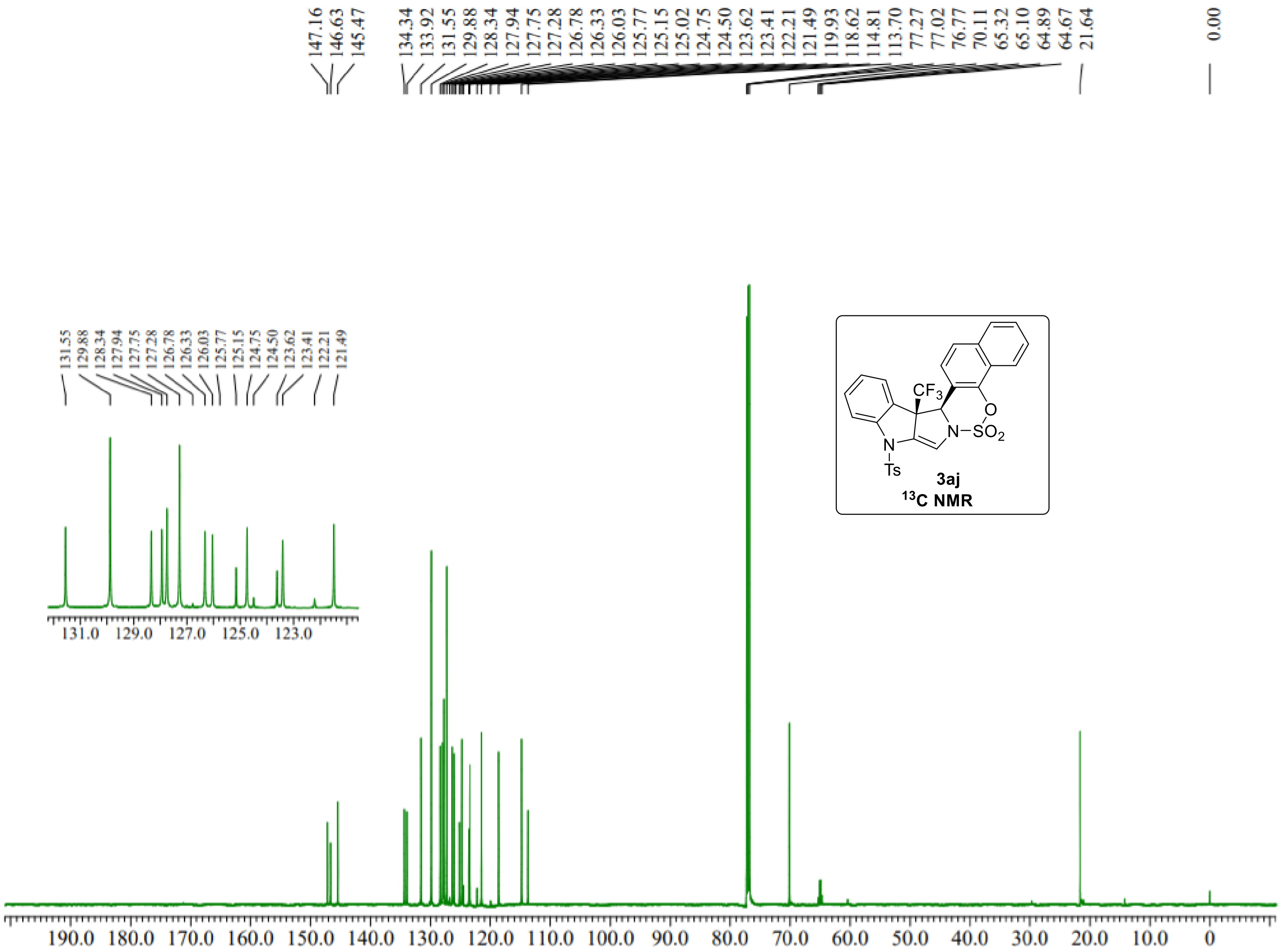

Supplementary Figure 65. ^{13}C NMR of **3aj**.

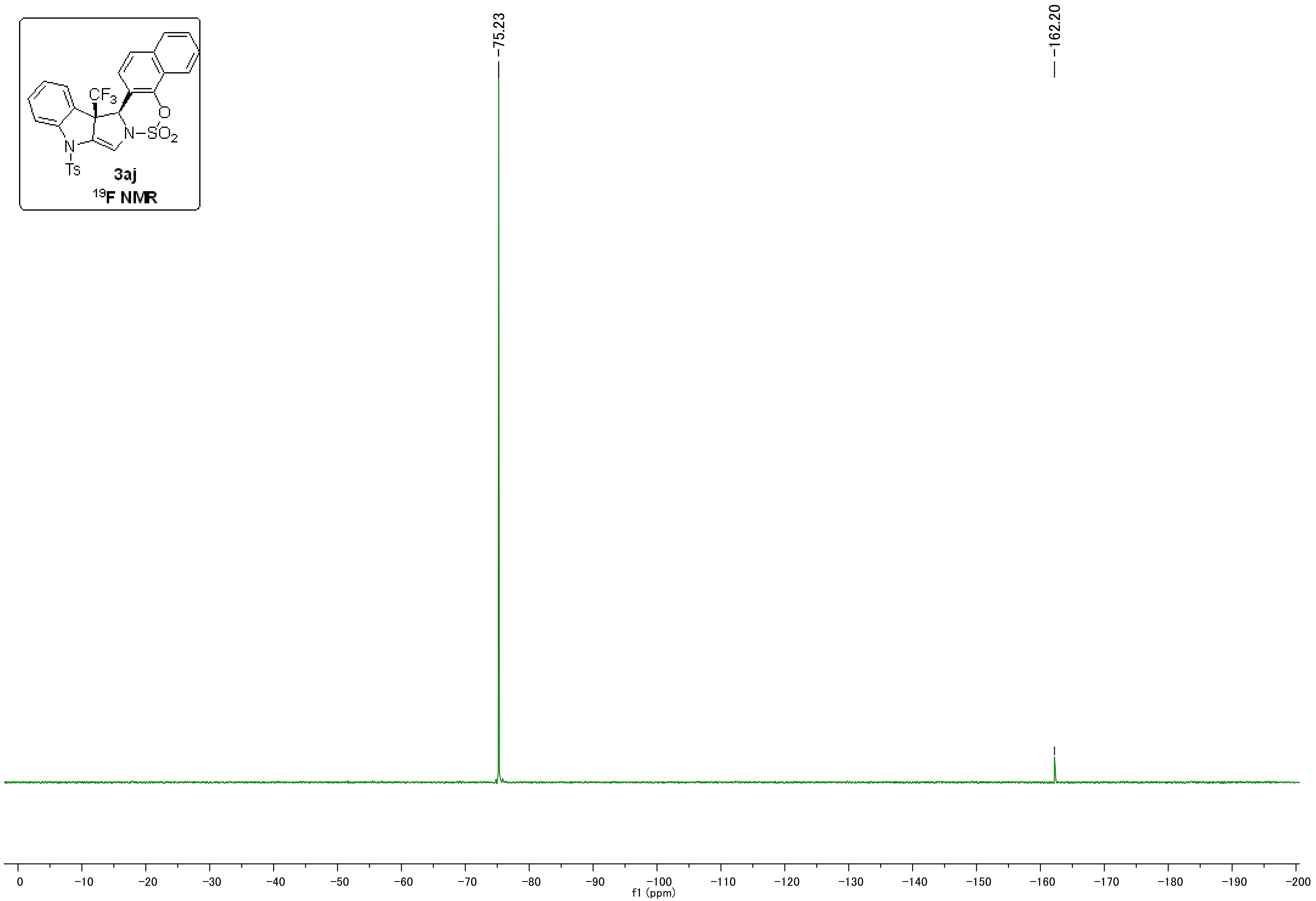

Supplementary Figure 66. ^{19}F NMR of **3aj**.

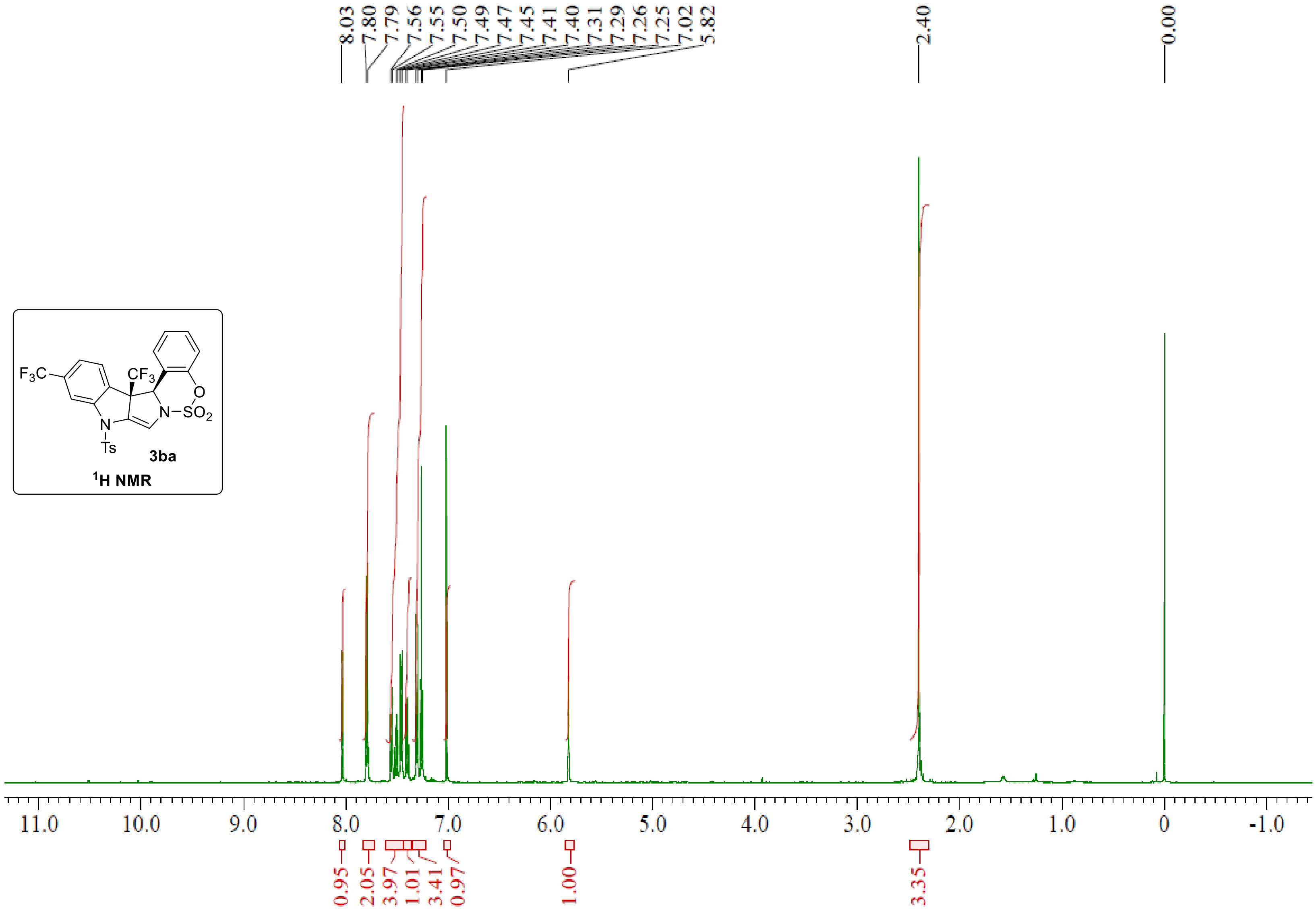

Supplementary Figure 67. ^{1}H NMR of **3ba**.

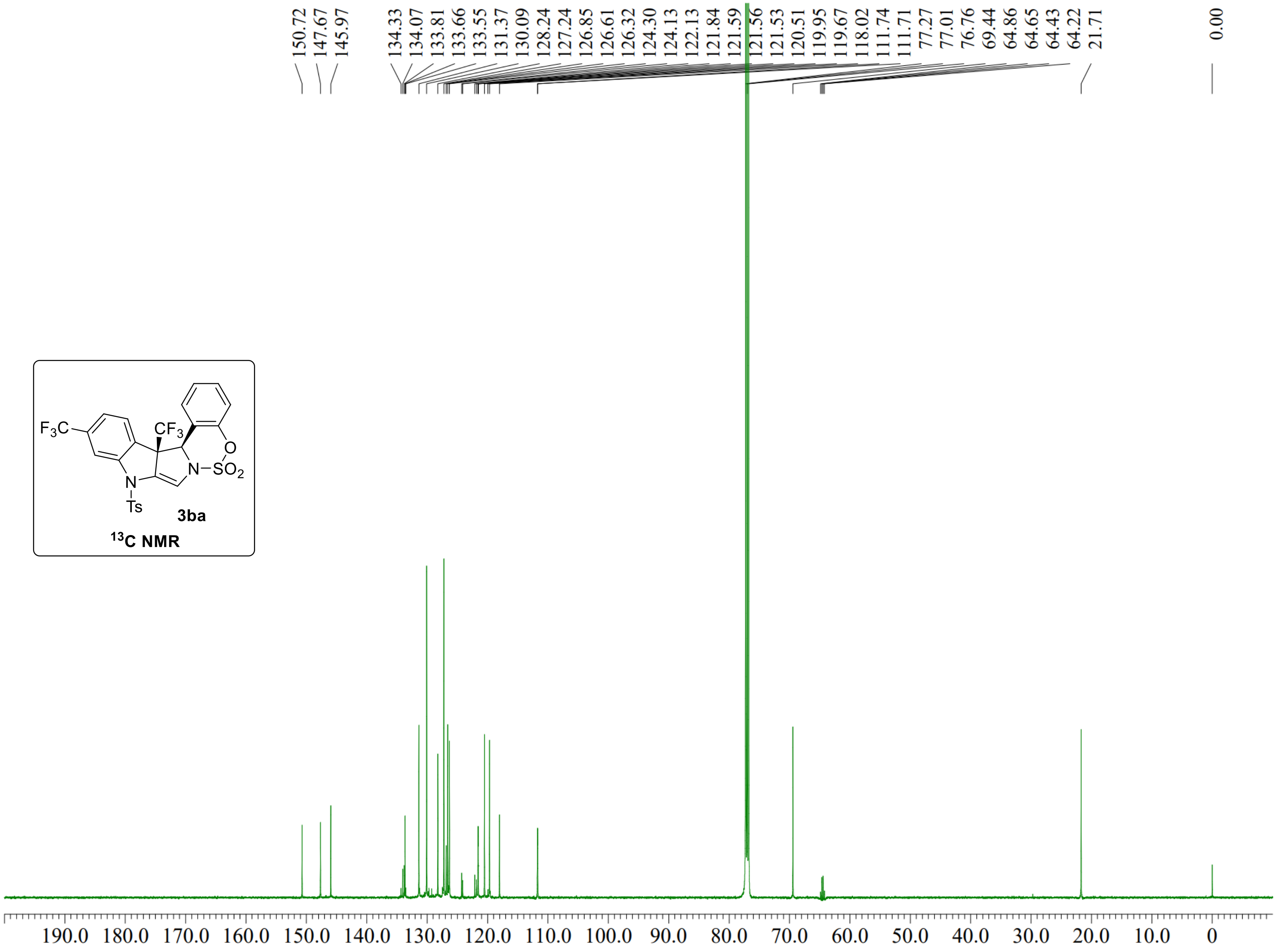

Supplementary Figure 68. ^{13}C NMR of **3ba**.

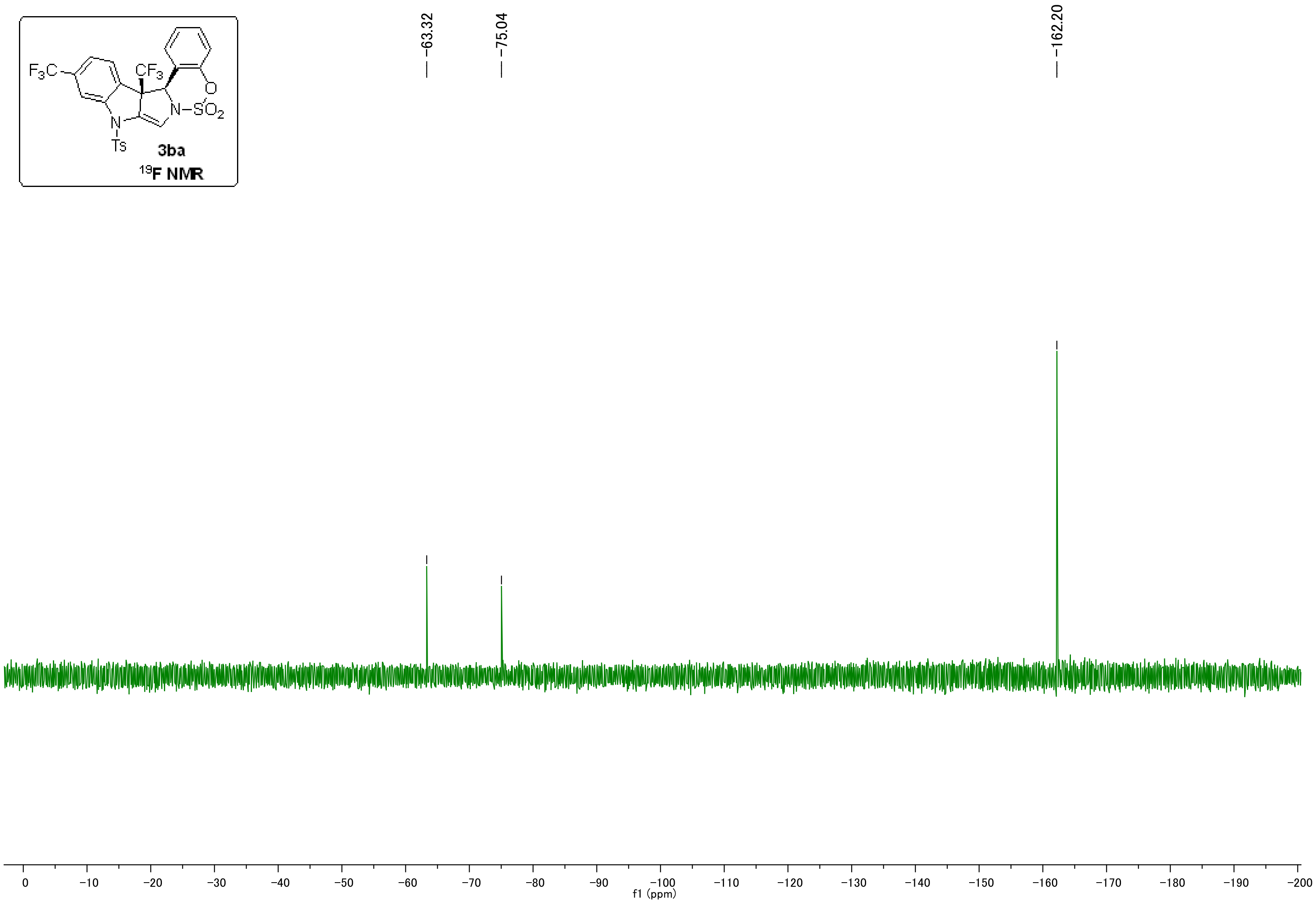

Supplementary Figure 69. ^{19}F NMR of **3ba**.

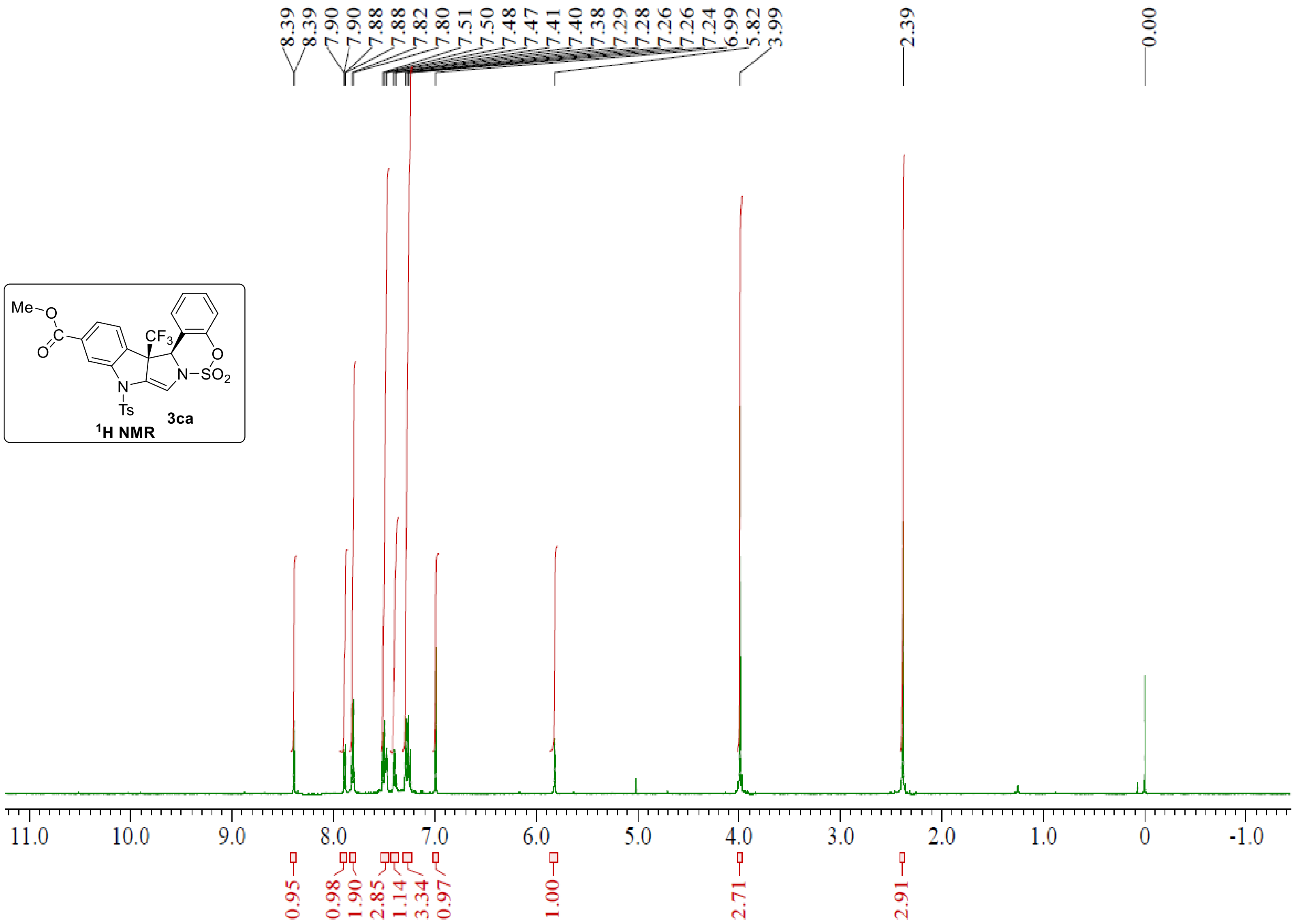

Supplementary Figure 70. ^{1}H NMR of **3ca**.

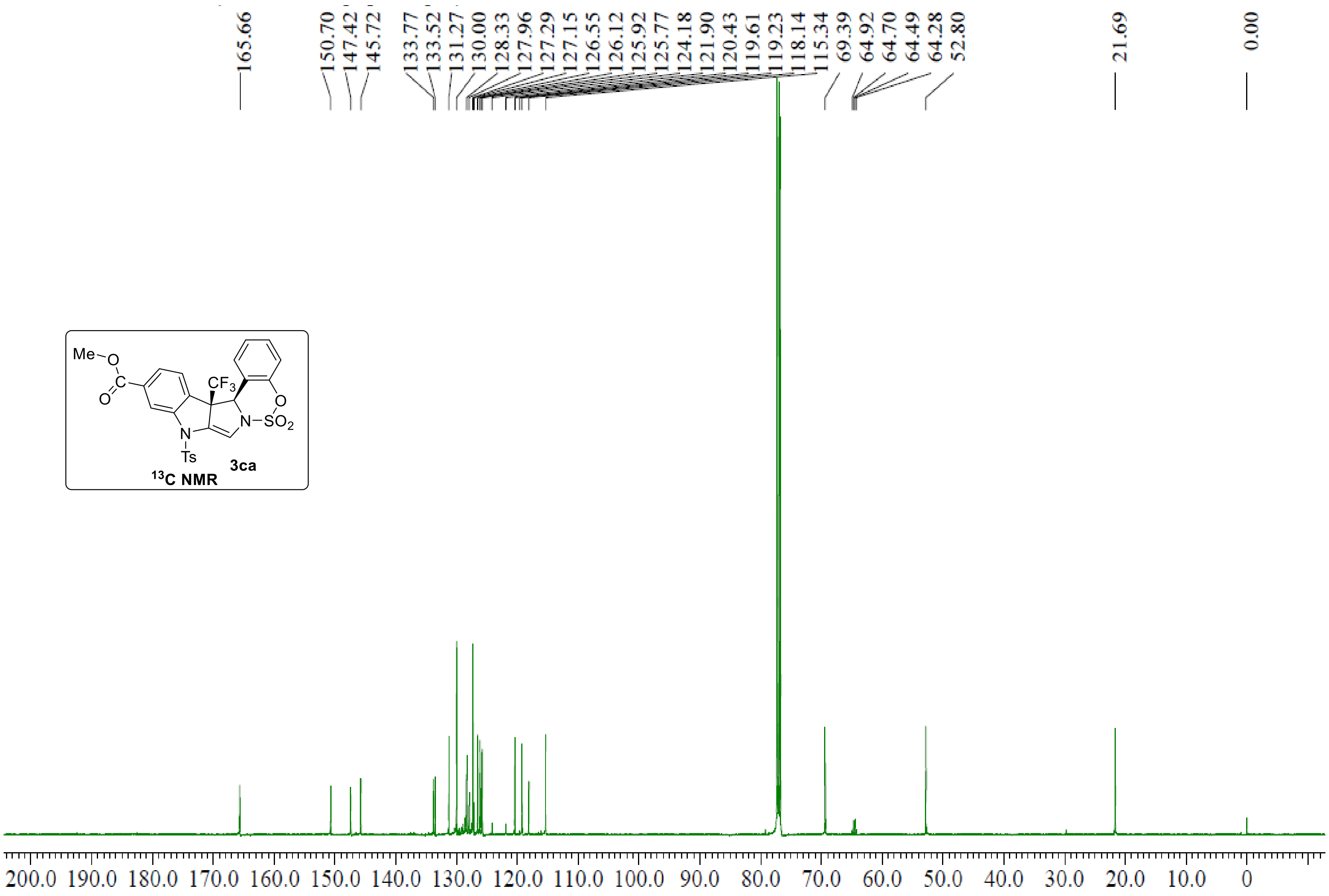

Supplementary Figure 71. ^{13}C NMR of **3ca**.

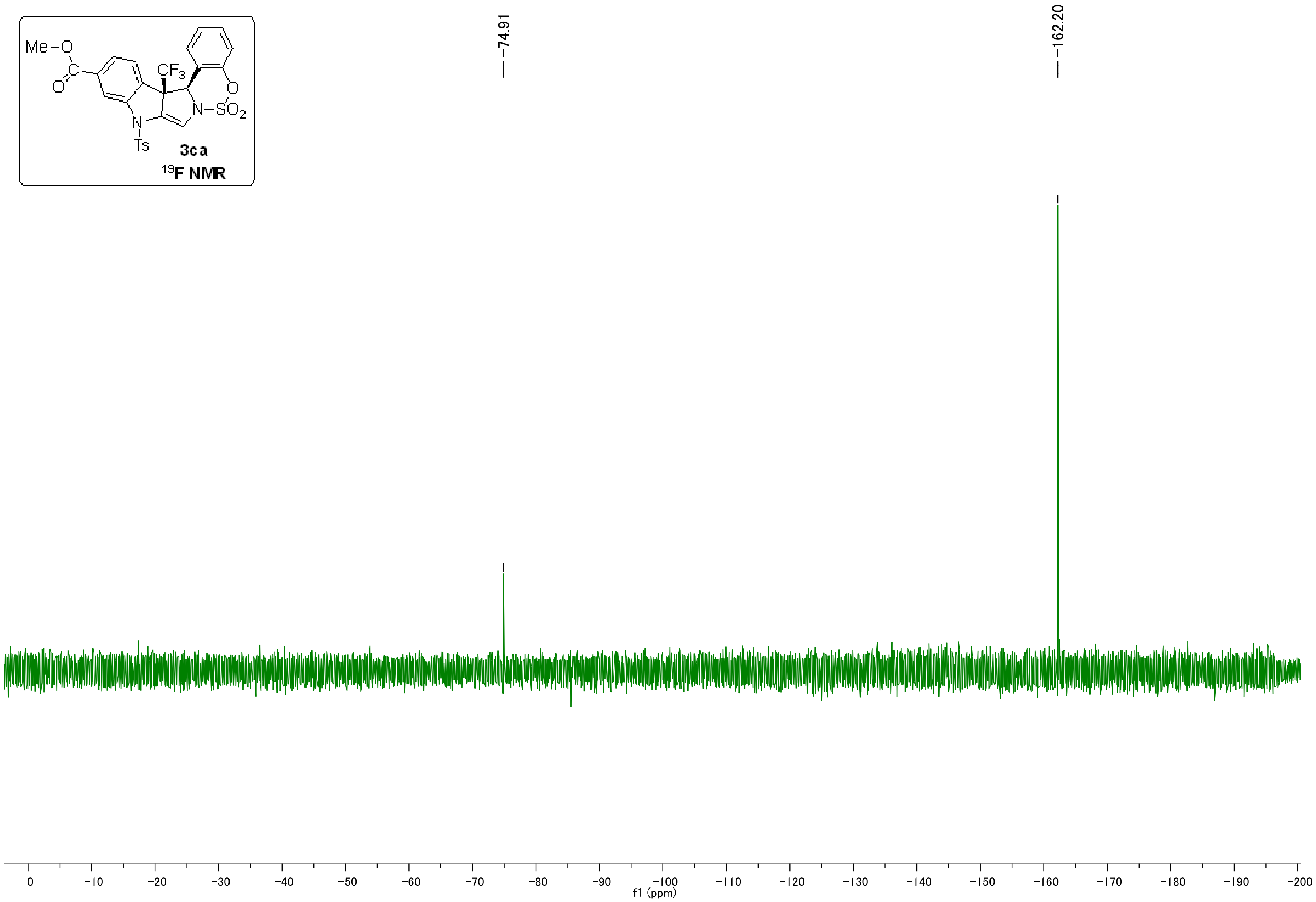

Supplementary Figure 72. ^{19}F NMR of **3ca**.

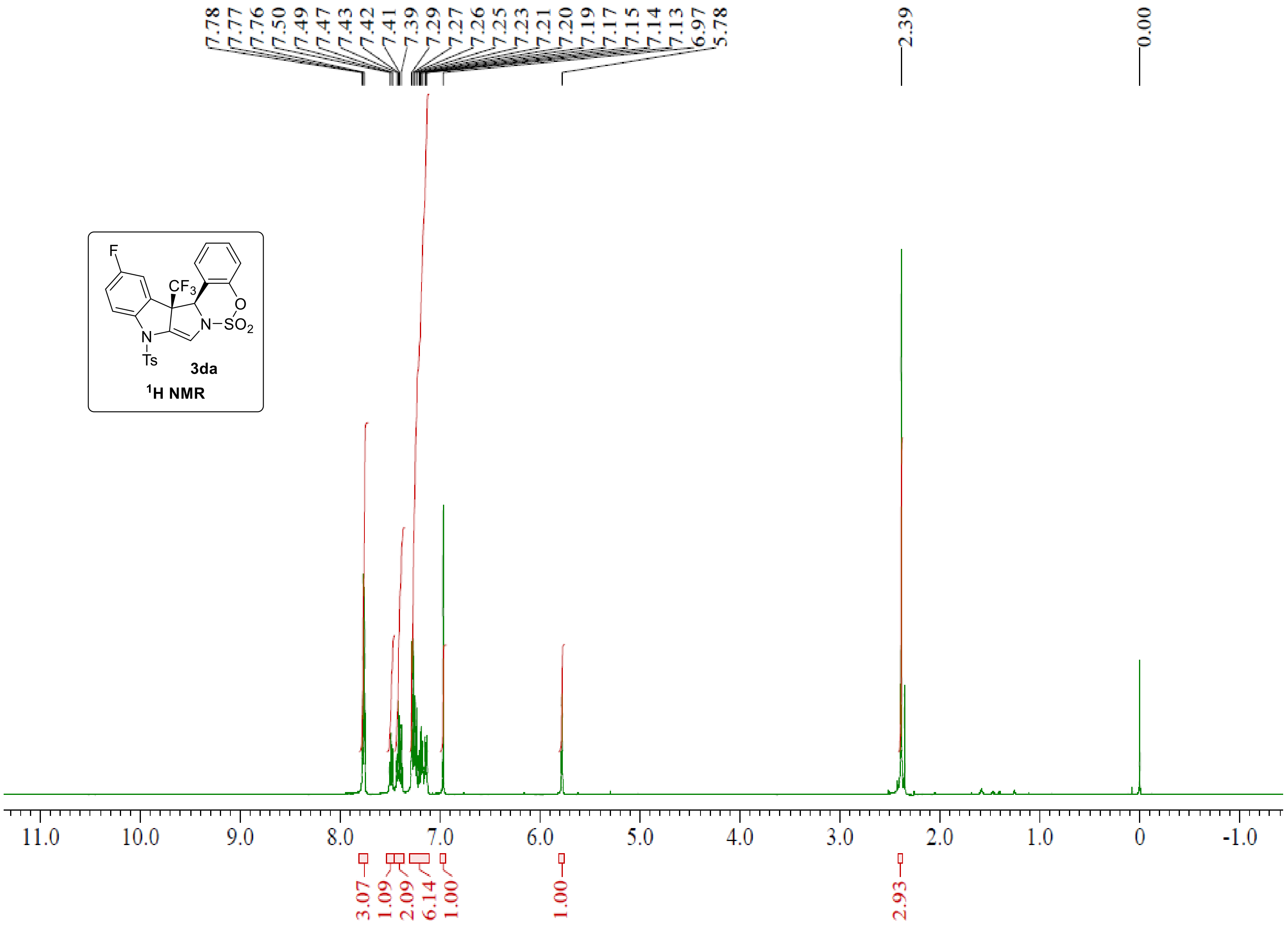

Supplementary Figure 73. ^{1}H NMR of **3da**.

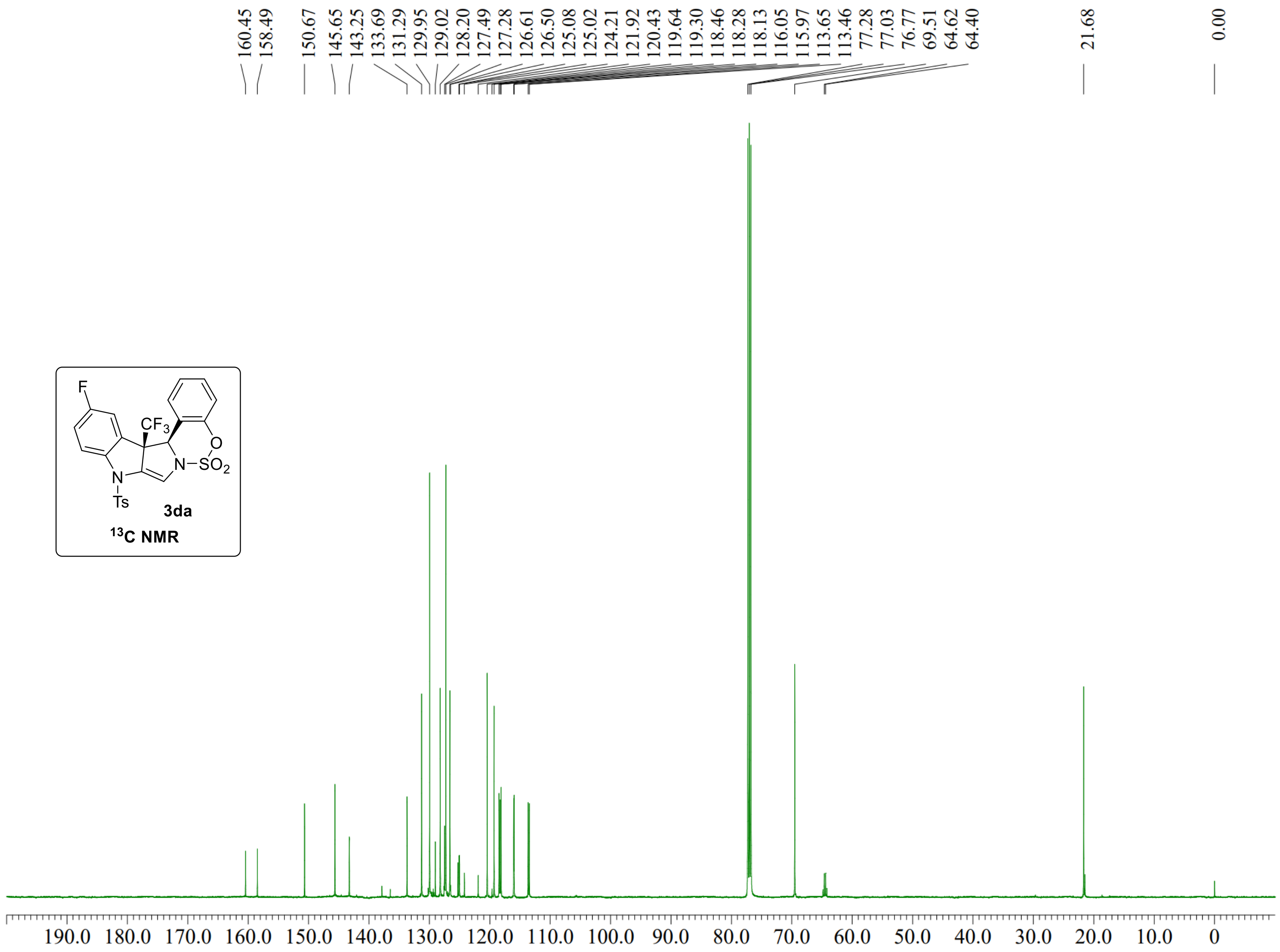

Supplementary Figure 74. ^{13}C NMR of **3da**.

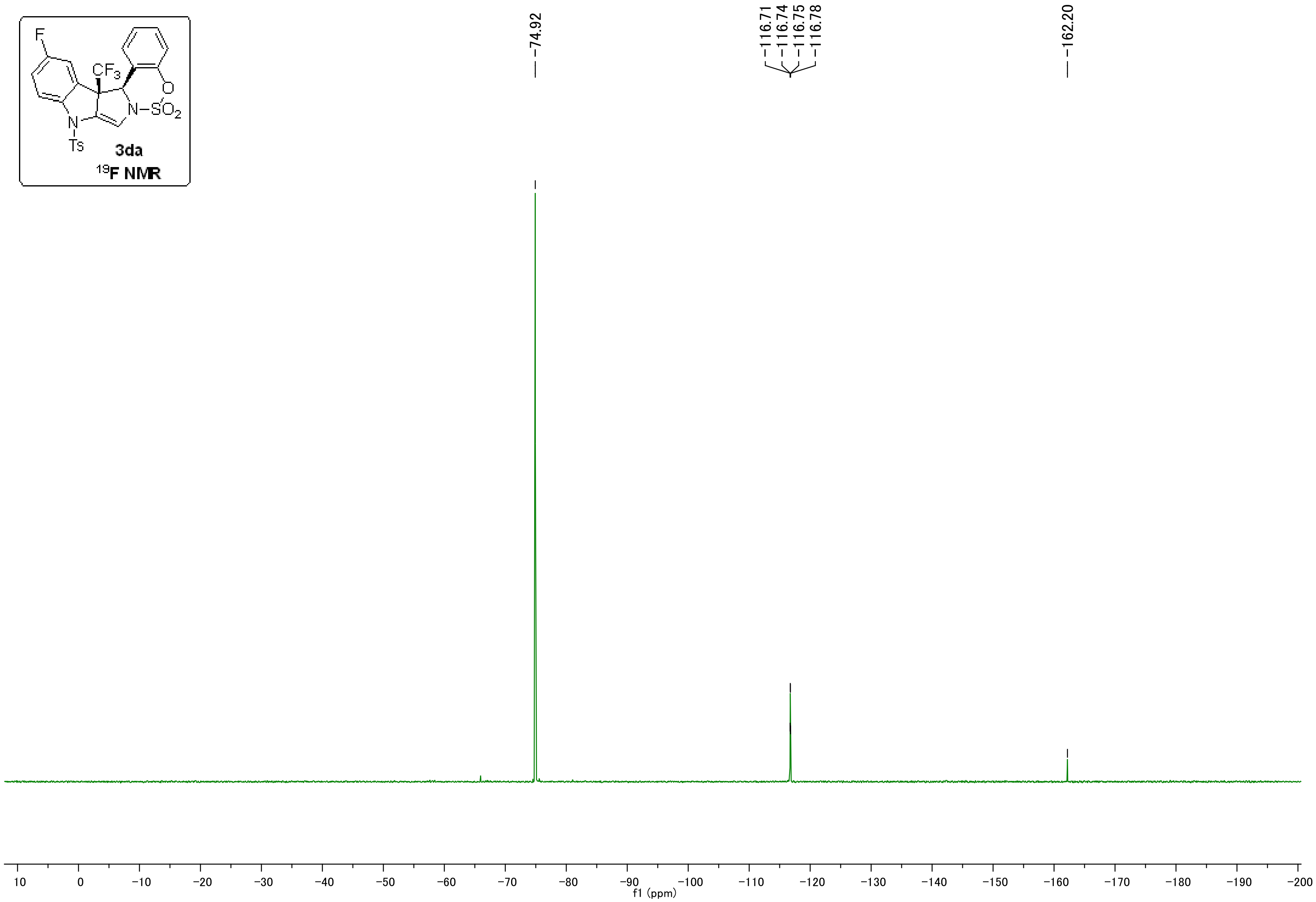

Supplementary Figure 75. ^{19}F NMR of **3da**.

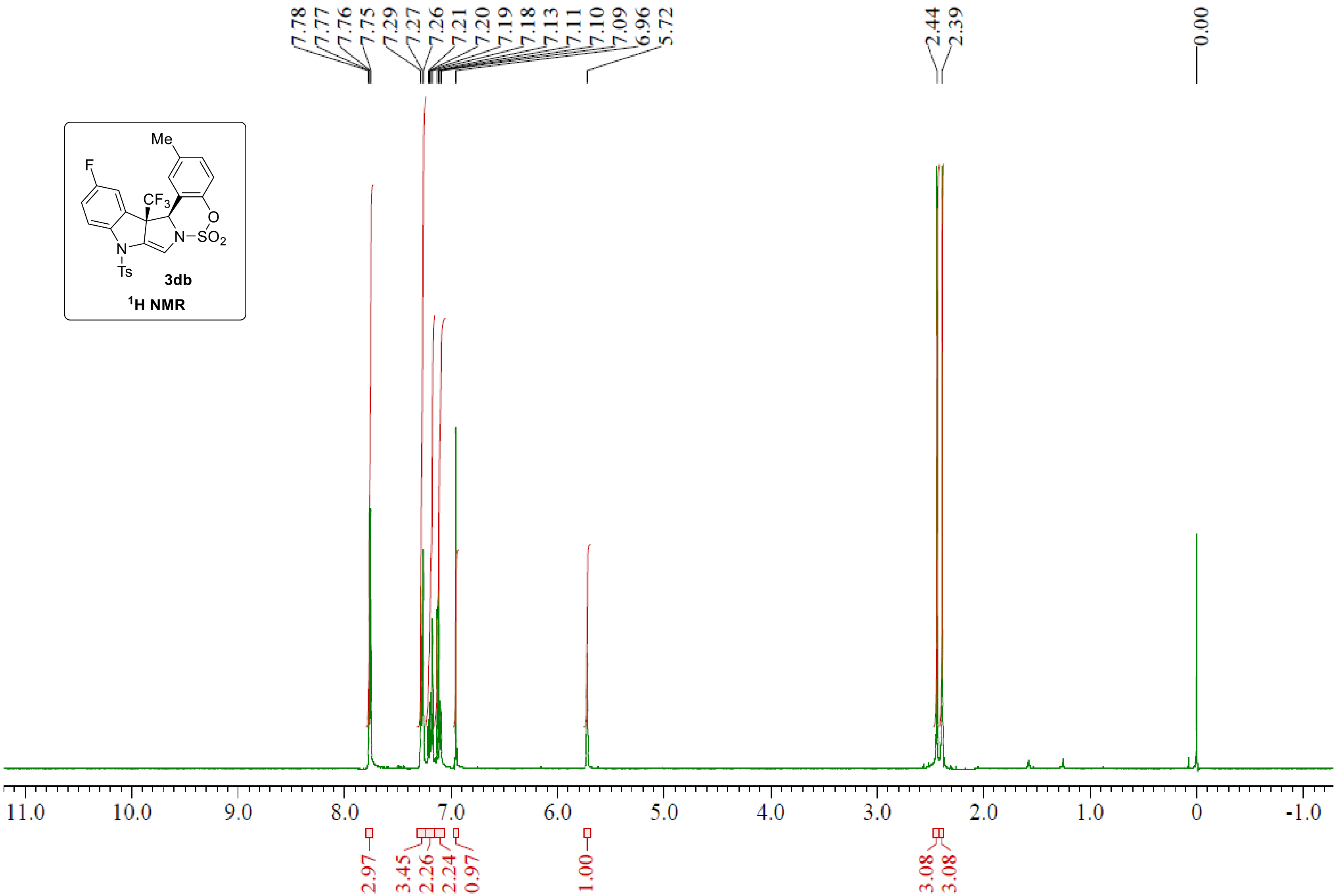

Supplementary Figure 76. ^{1}H NMR of **3db**.

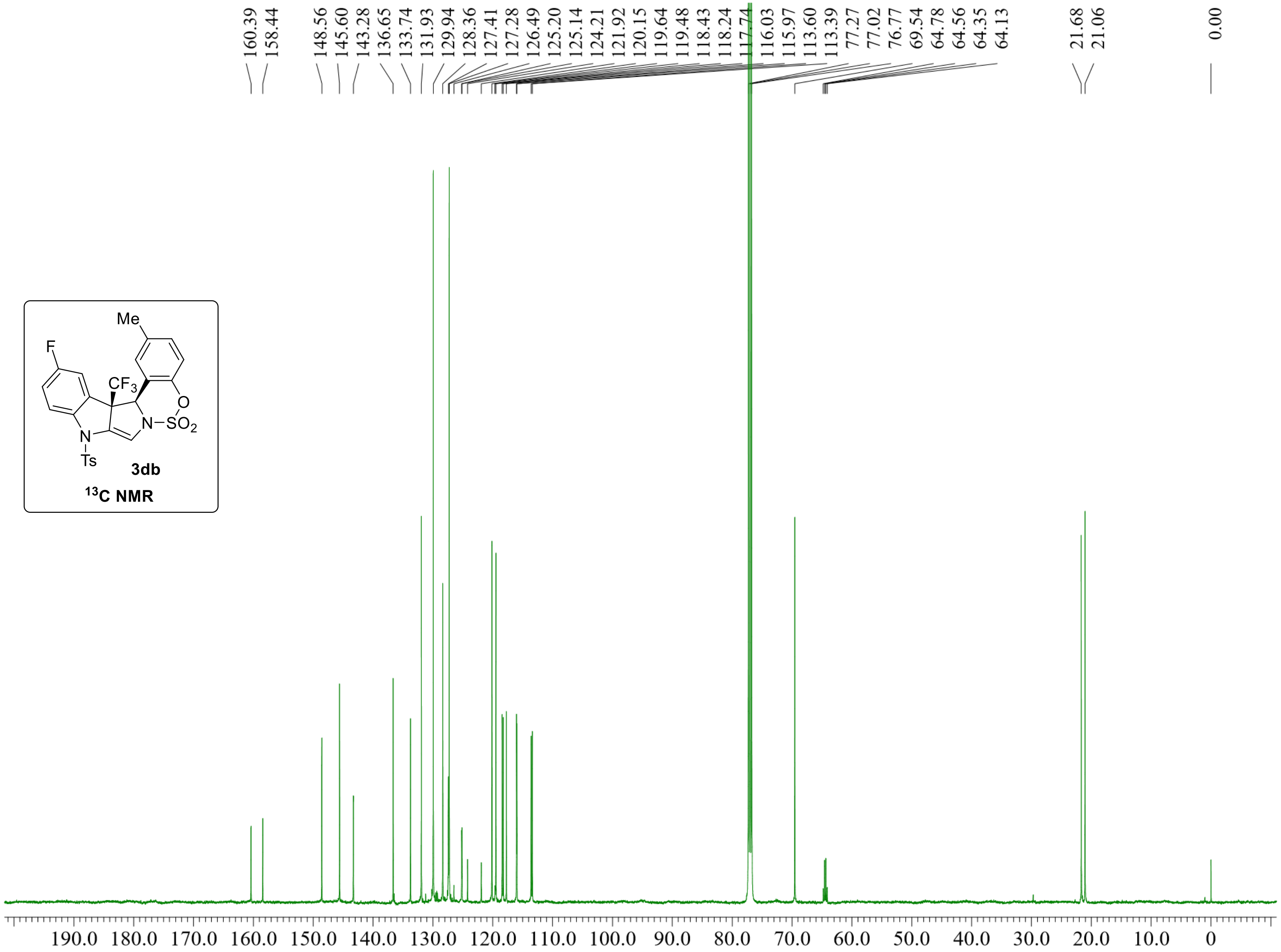

Supplementary Figure 77. ^{13}C NMR of **3db**.

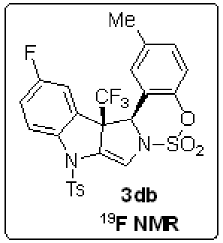

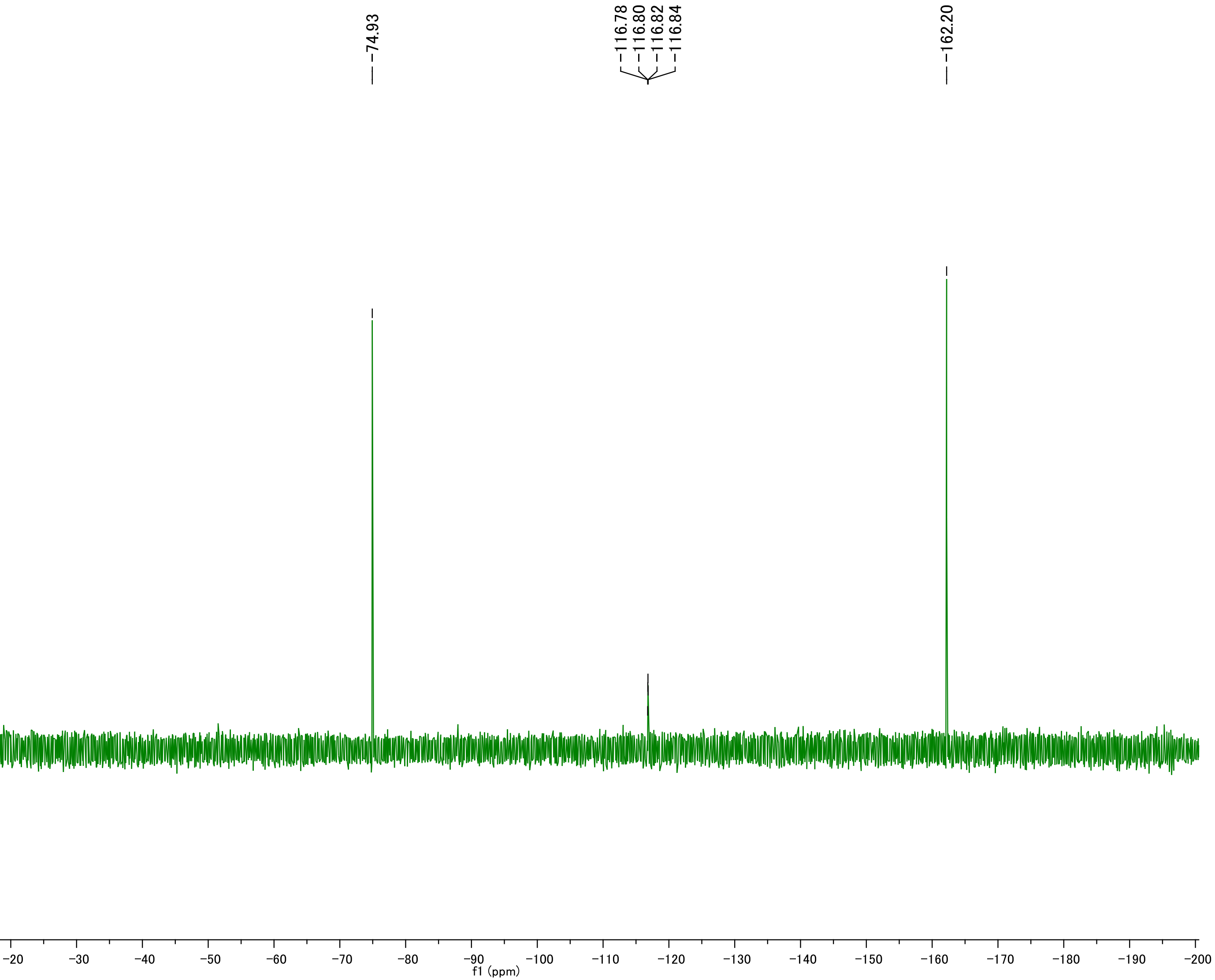

Supplementary Figure 78. ^{19}F NMR of **3db**.

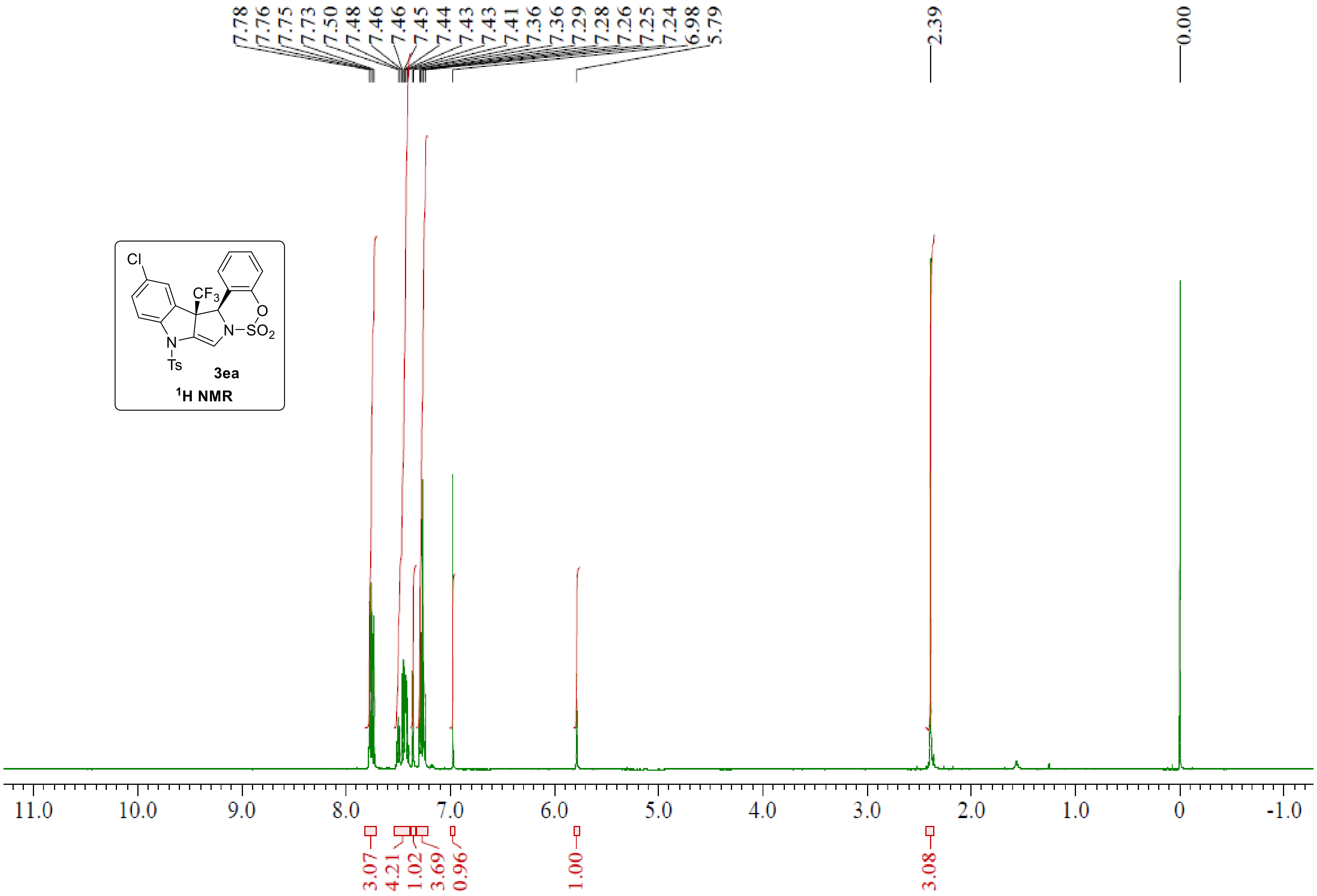

Supplementary Figure 79. ^{1}H NMR of **3ea**.

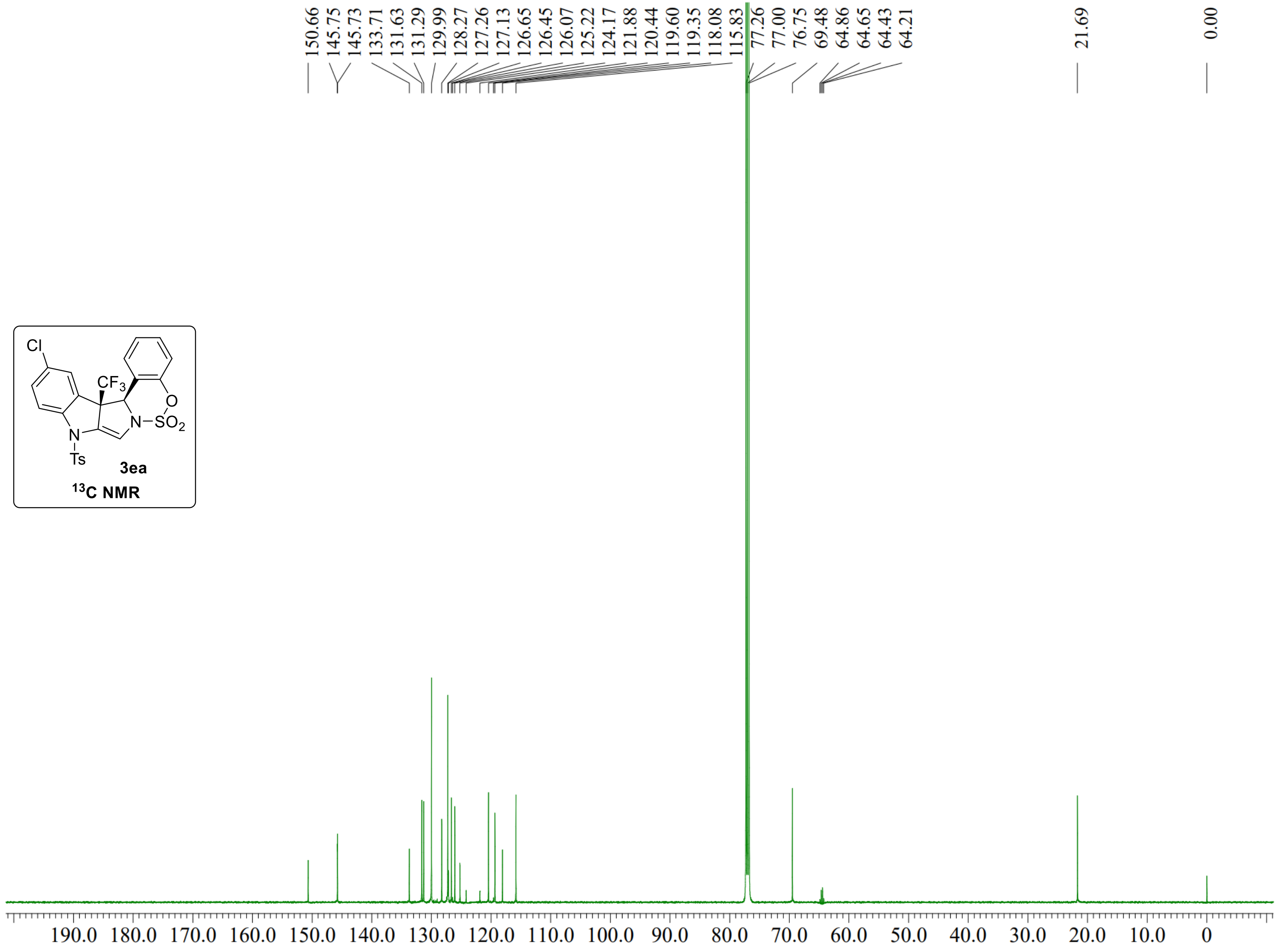

Supplementary Figure 80. ^{13}C NMR of **3ea**.

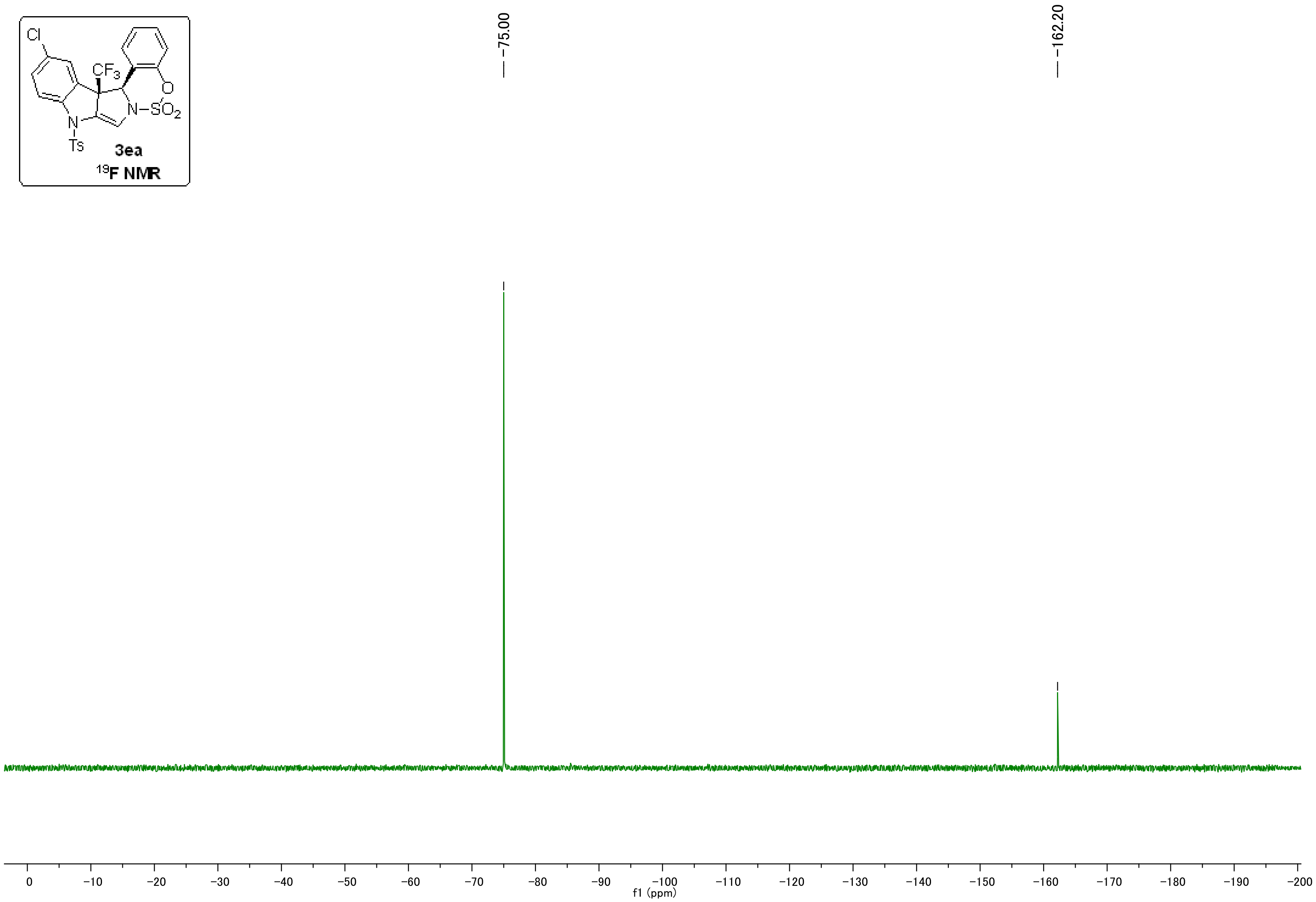

Supplementary Figure 81. ^{19}F NMR of **3ea**.

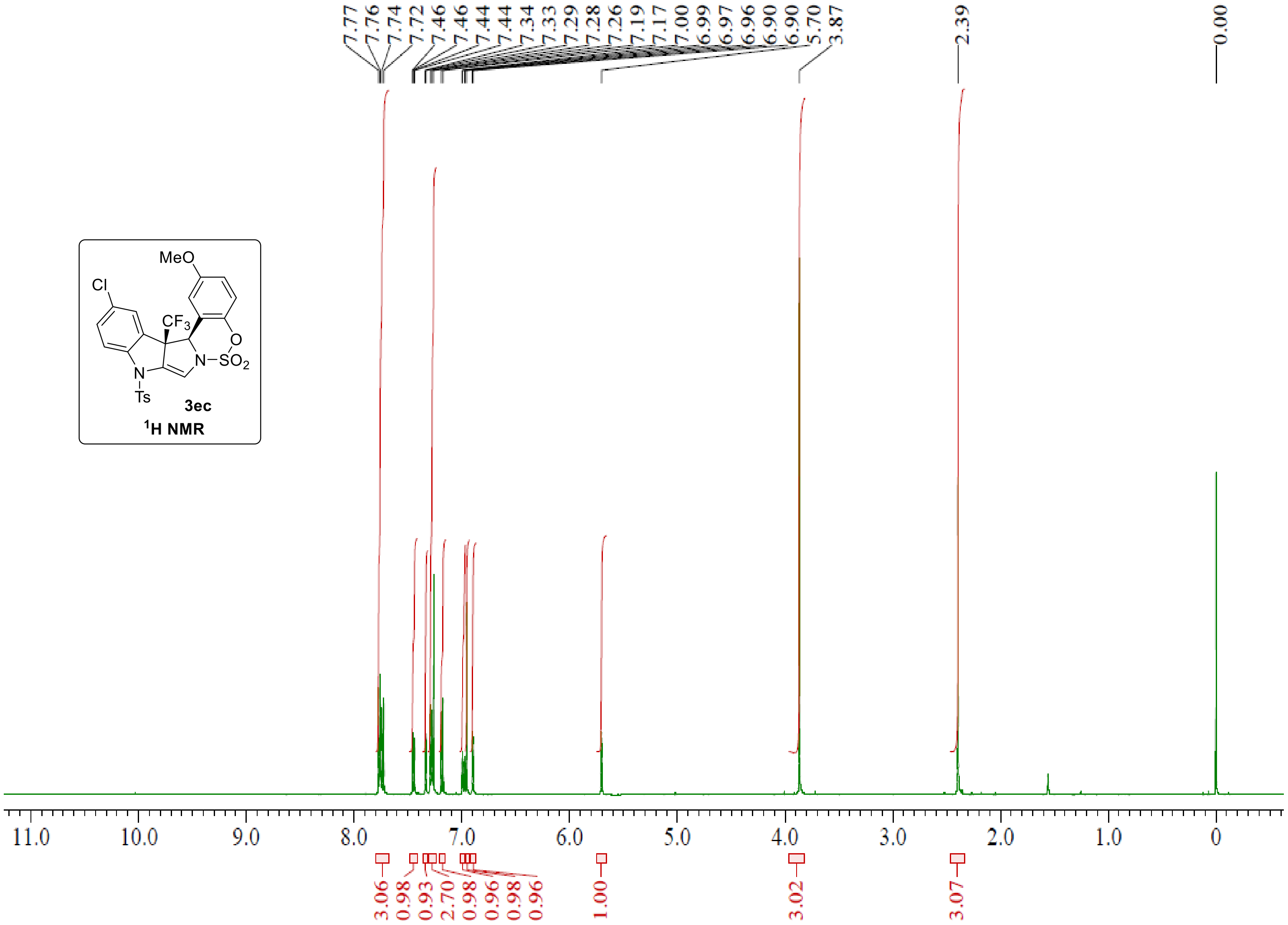

Supplementary Figure 82. ^{1}H NMR of **3ec**.

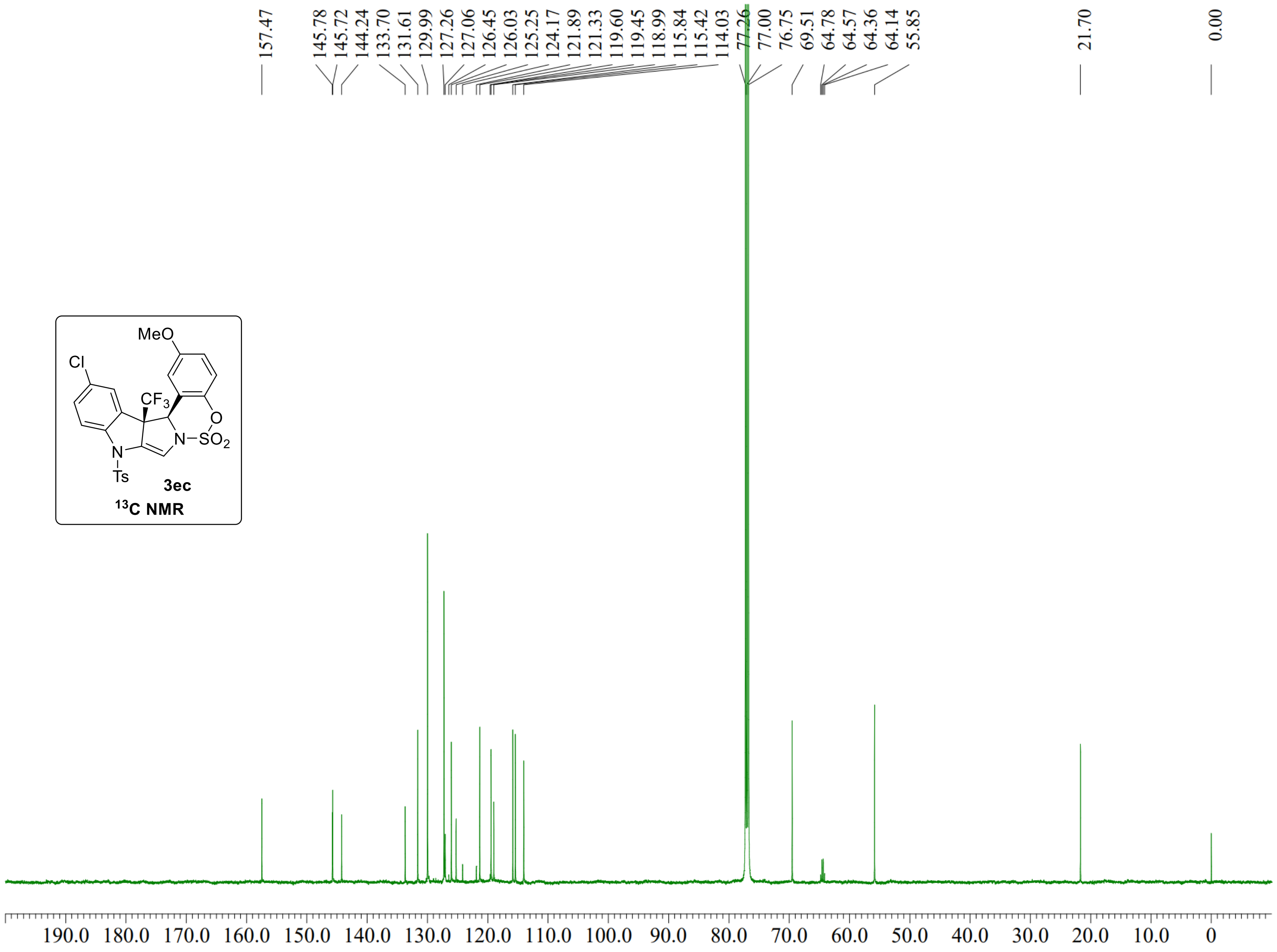

Supplementary Figure 83. ^{13}C NMR of **3ec**.

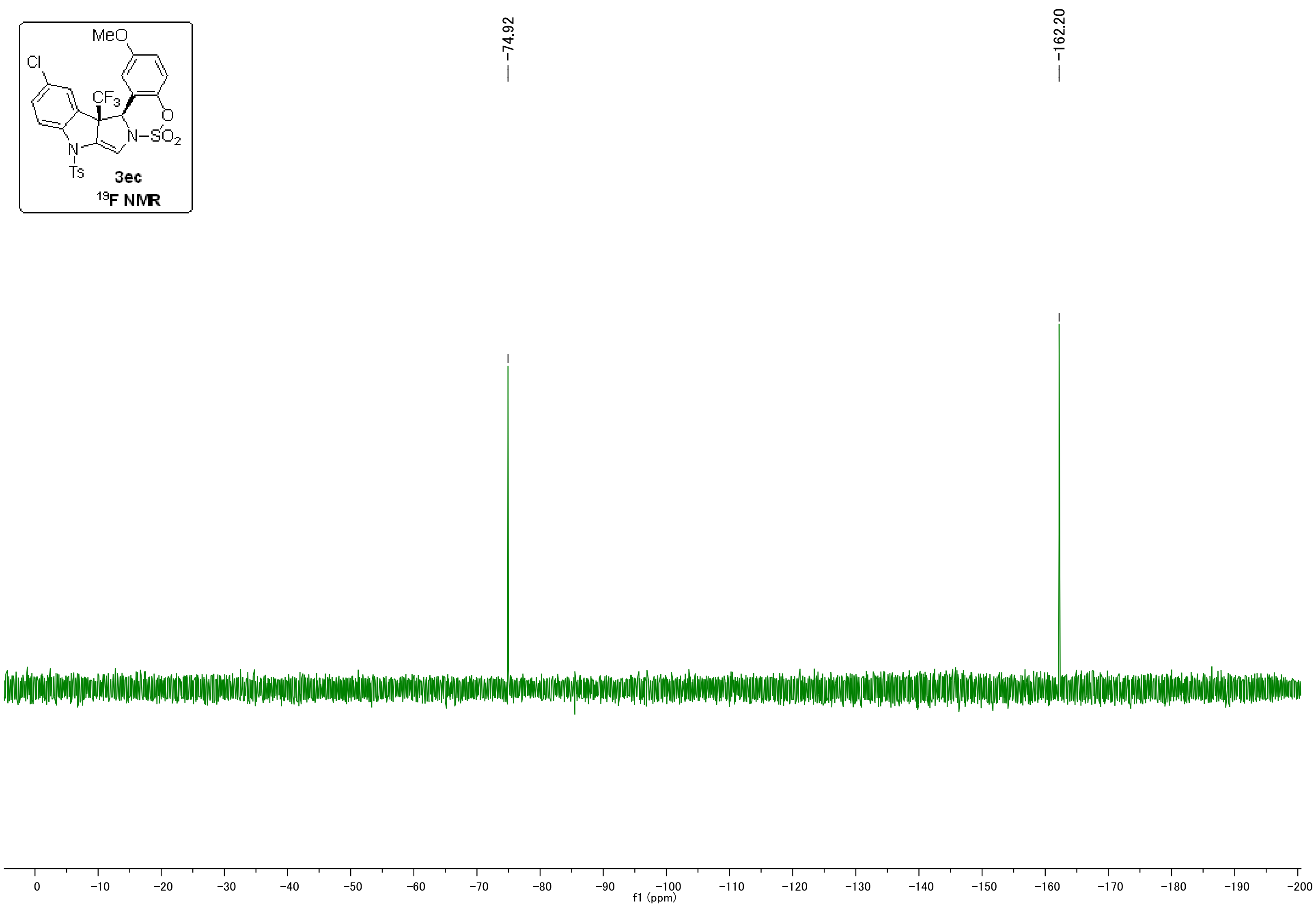

Supplementary Figure 84. ^{19}F NMR of **3ec**.

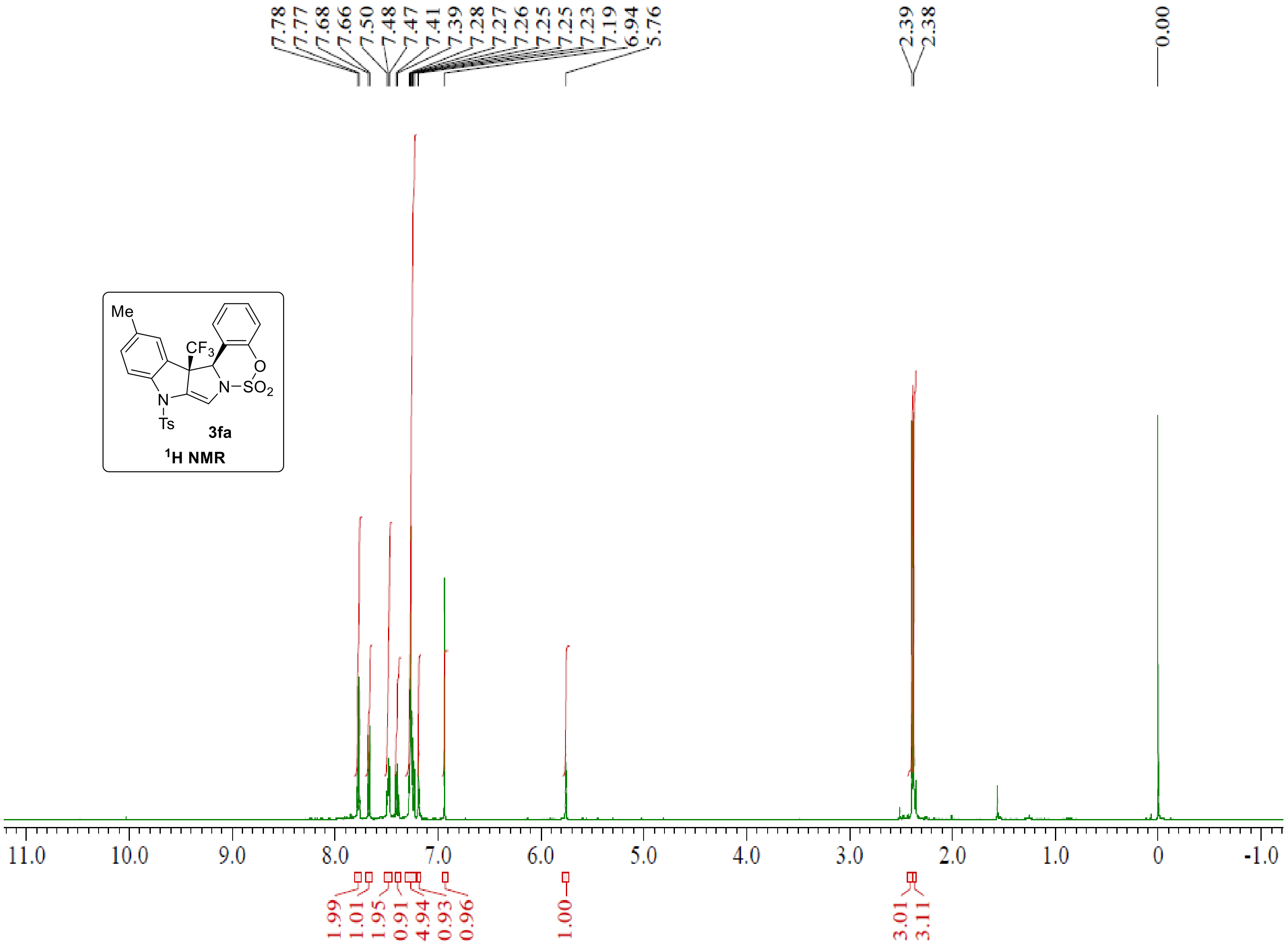

Supplementary Figure 85. ^{1}H NMR of **3fa**.

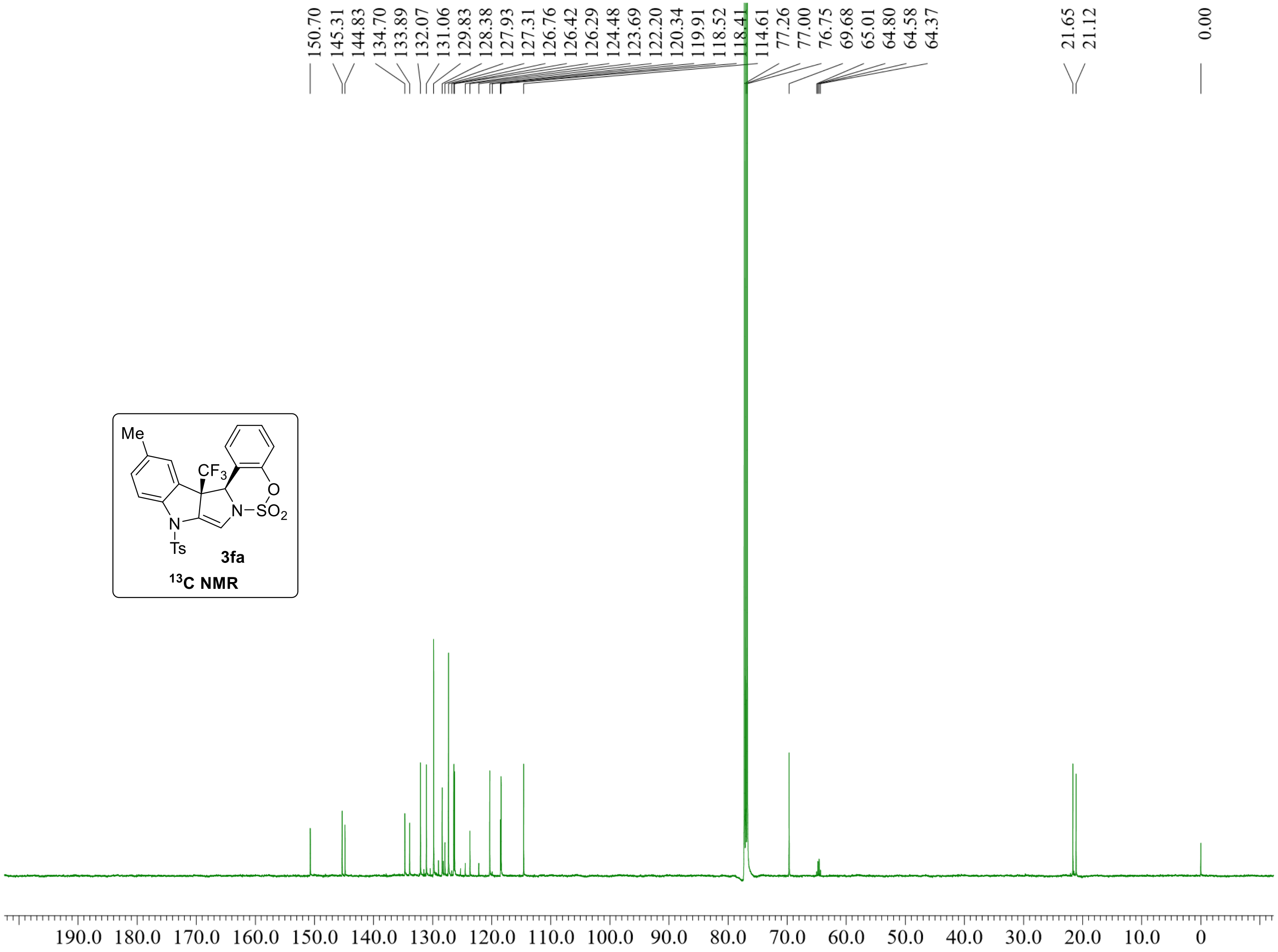

Supplementary Figure 86. ^{13}C NMR of **3fa**.

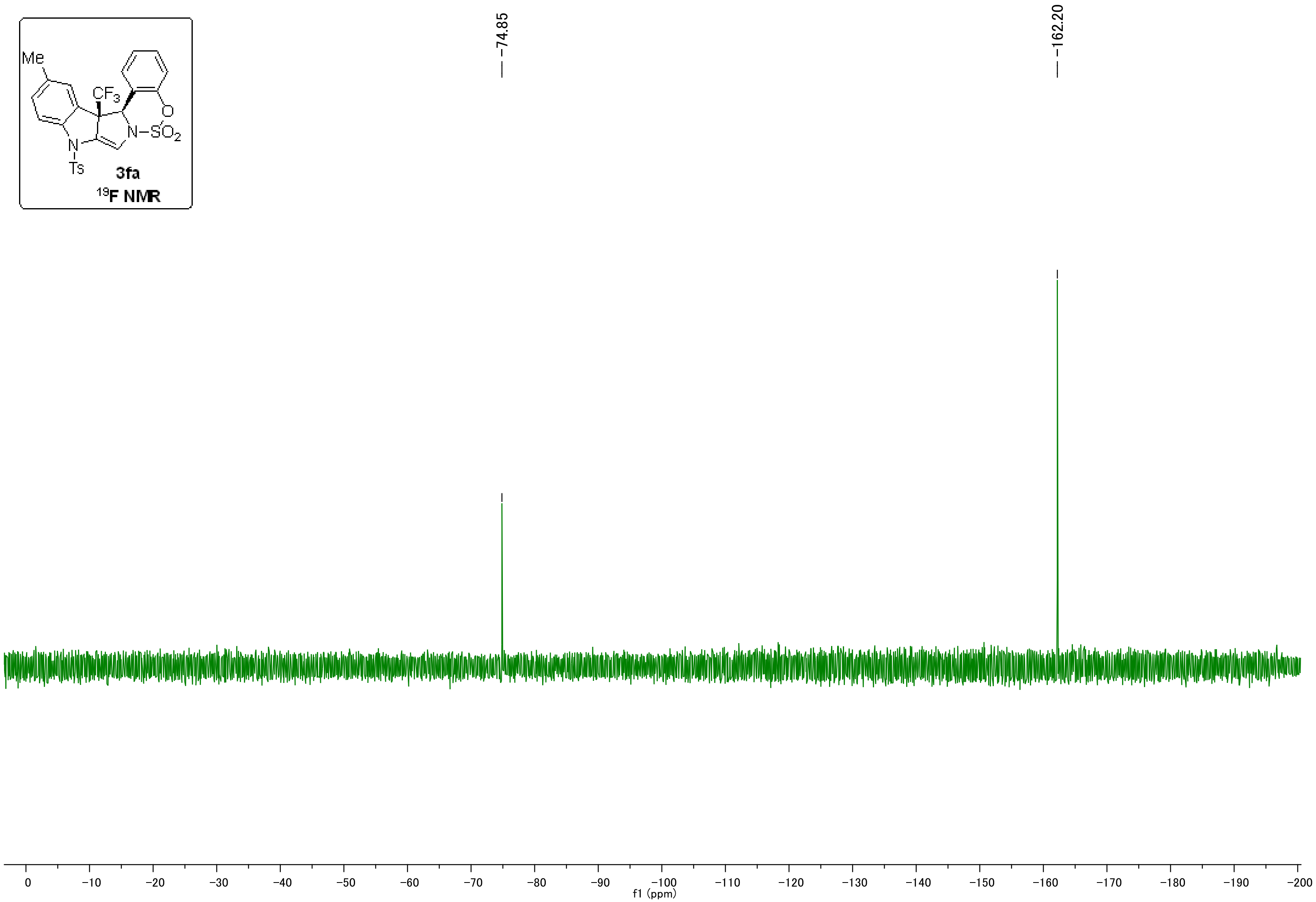

Supplementary Figure 87. ^{19}F NMR of **3fa**.

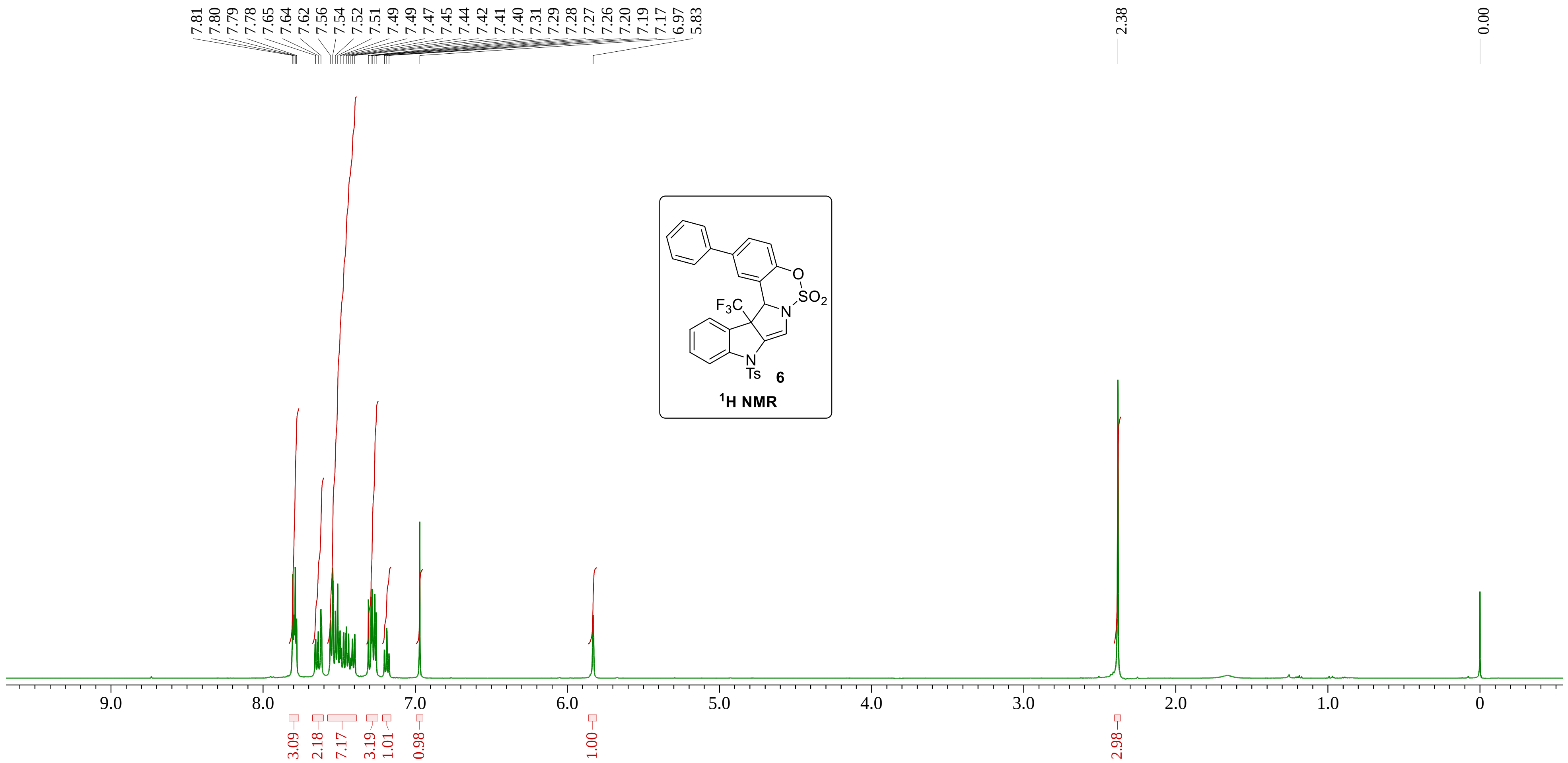

Supplementary Figure 88. ^{1}H NMR of **6**.

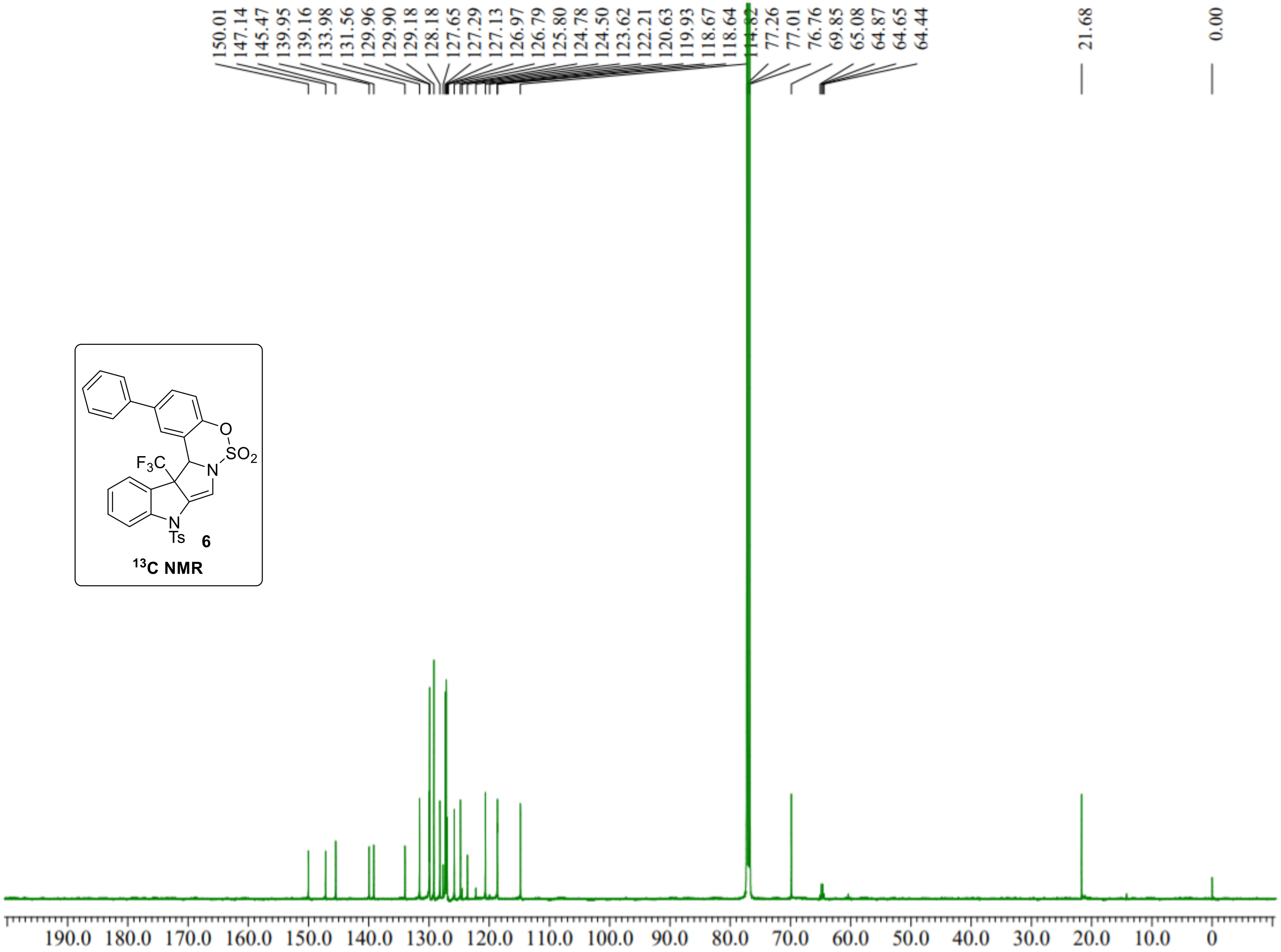

Supplementary Figure 89. ^{13}C NMR of **6**.

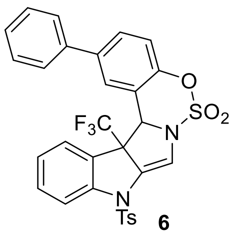

6

19F NMR

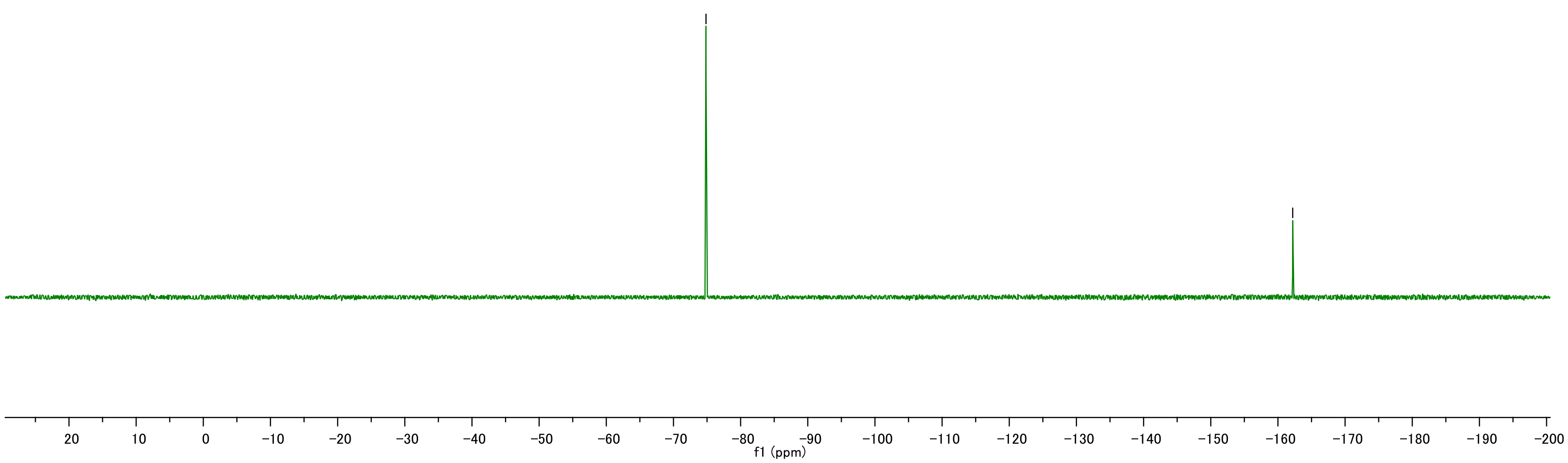

Supplementary Figure 90. ^{19}F NMR of **6**.

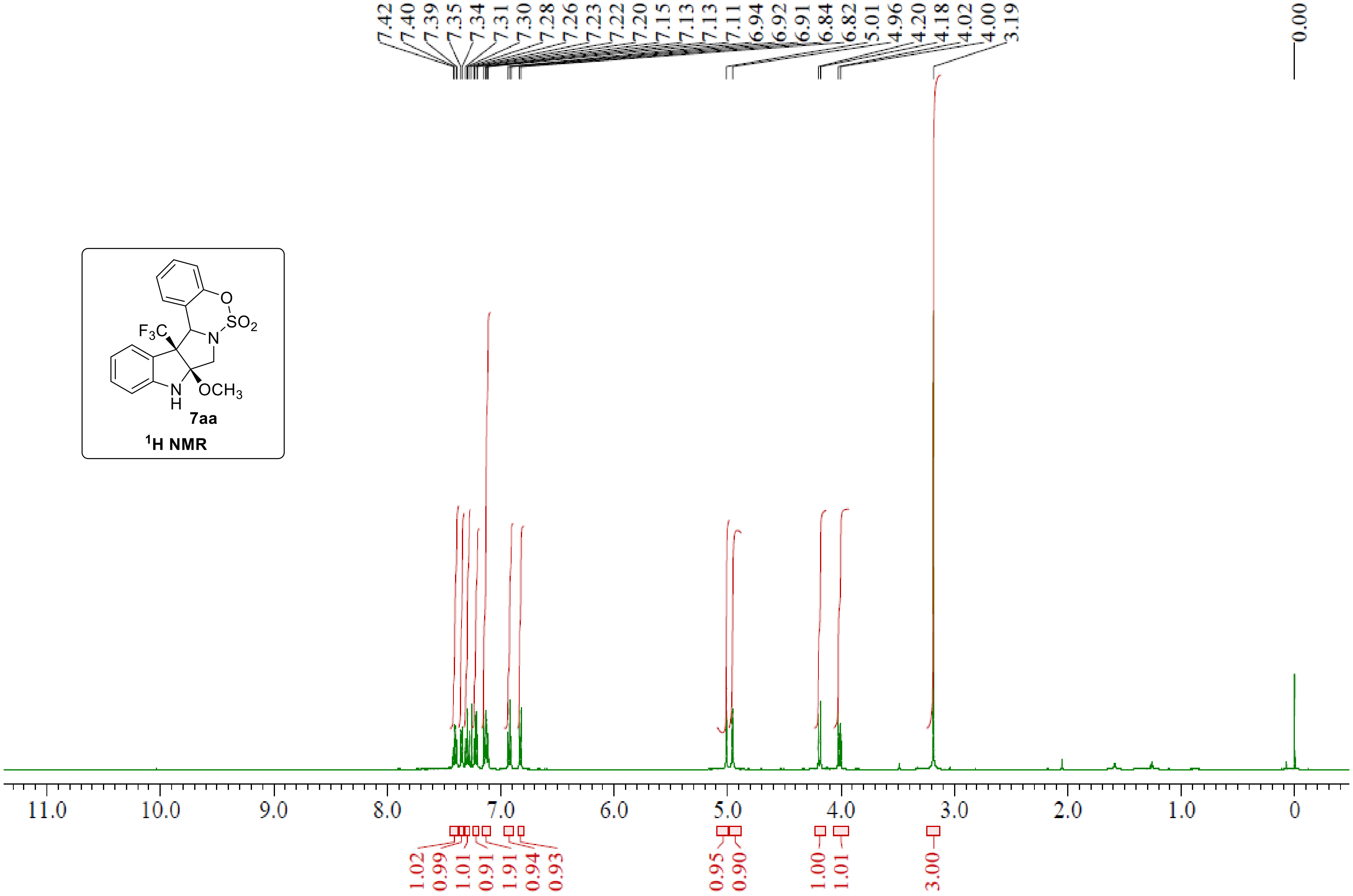

Supplementary Figure 91. ^{1}H NMR of **7aa**.

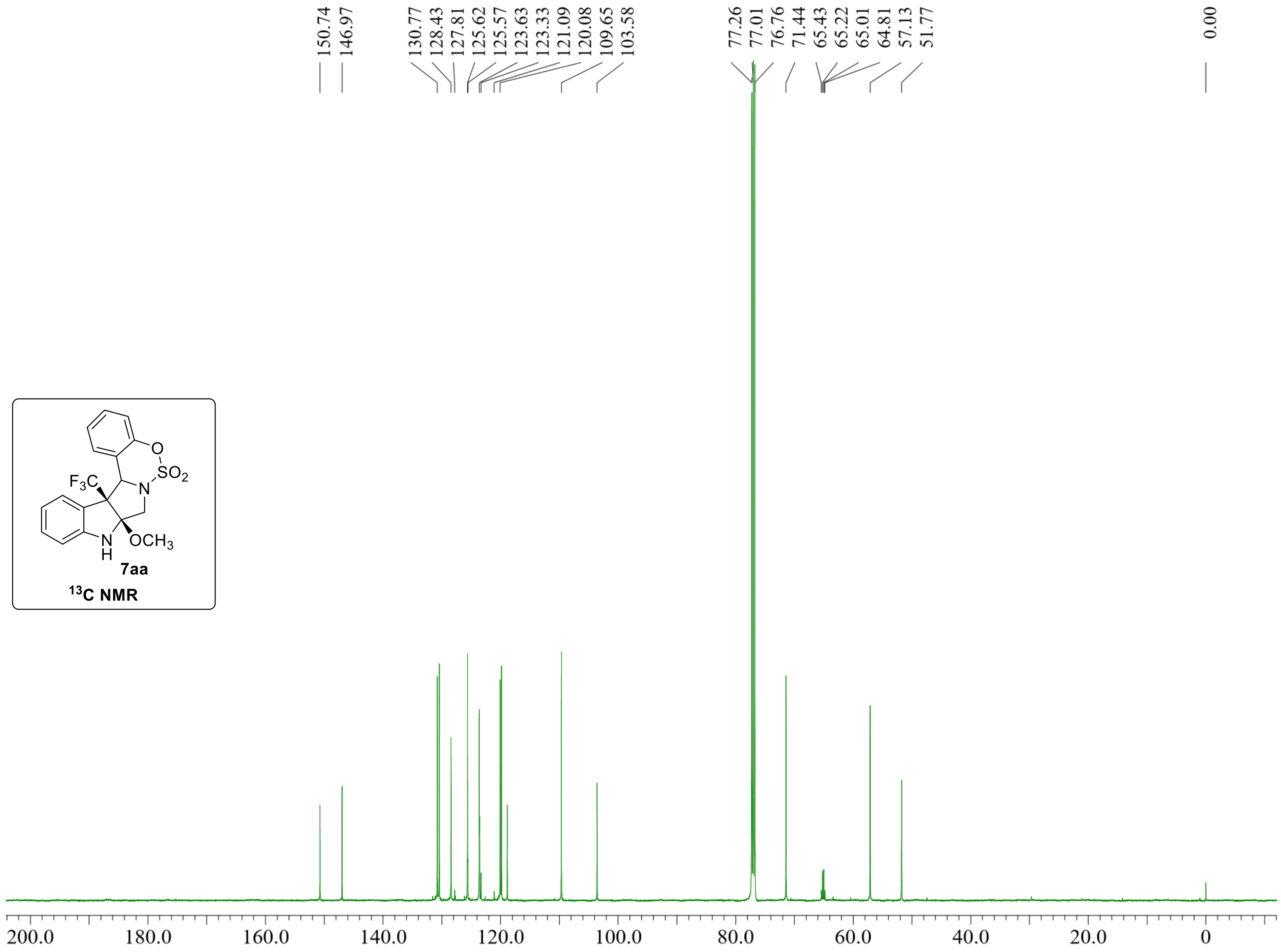

Supplementary Figure 92. ^{13}C NMR of **7aa**.

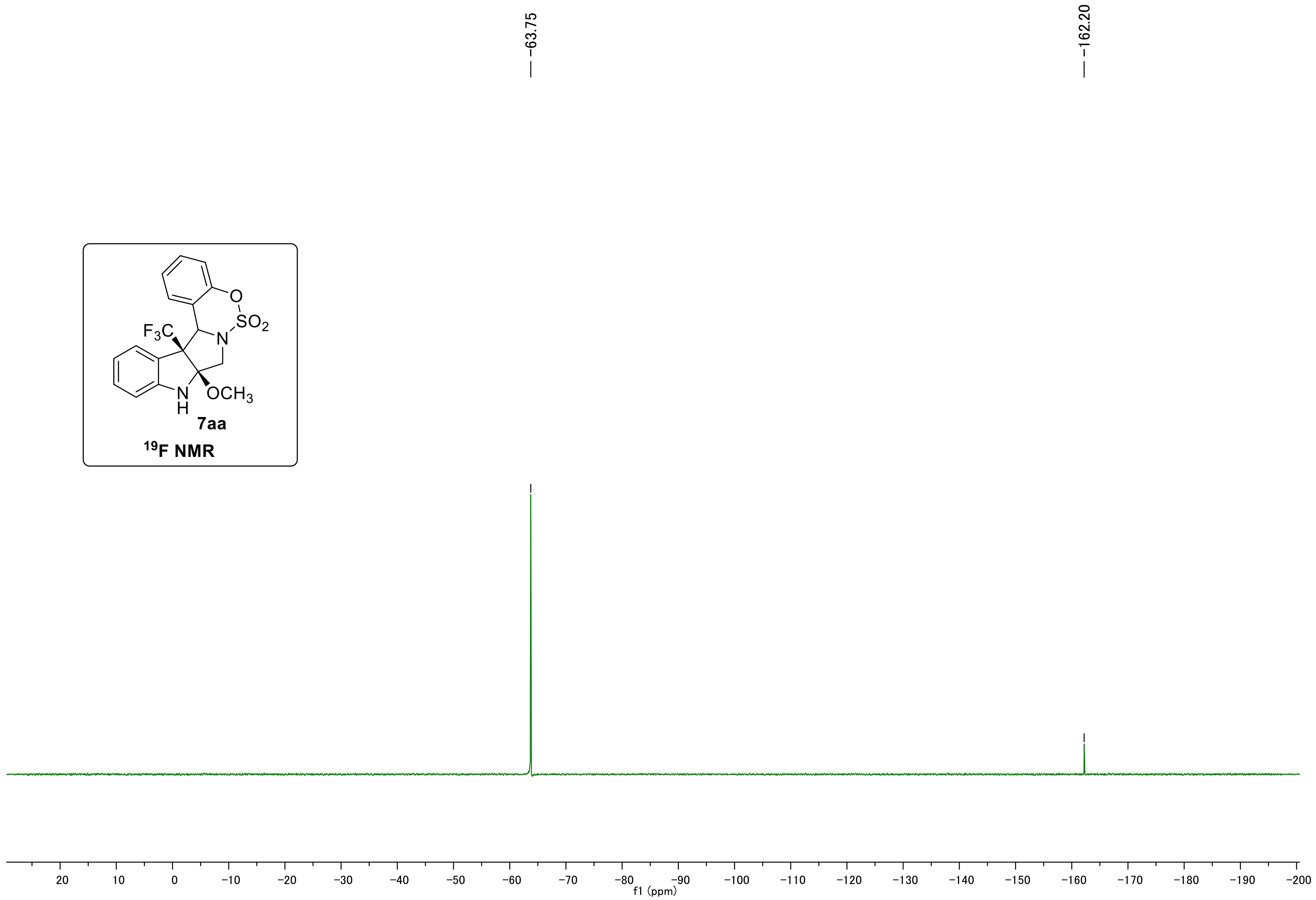

Supplementary Figure 93. ^{19}F NMR of **7aa**.

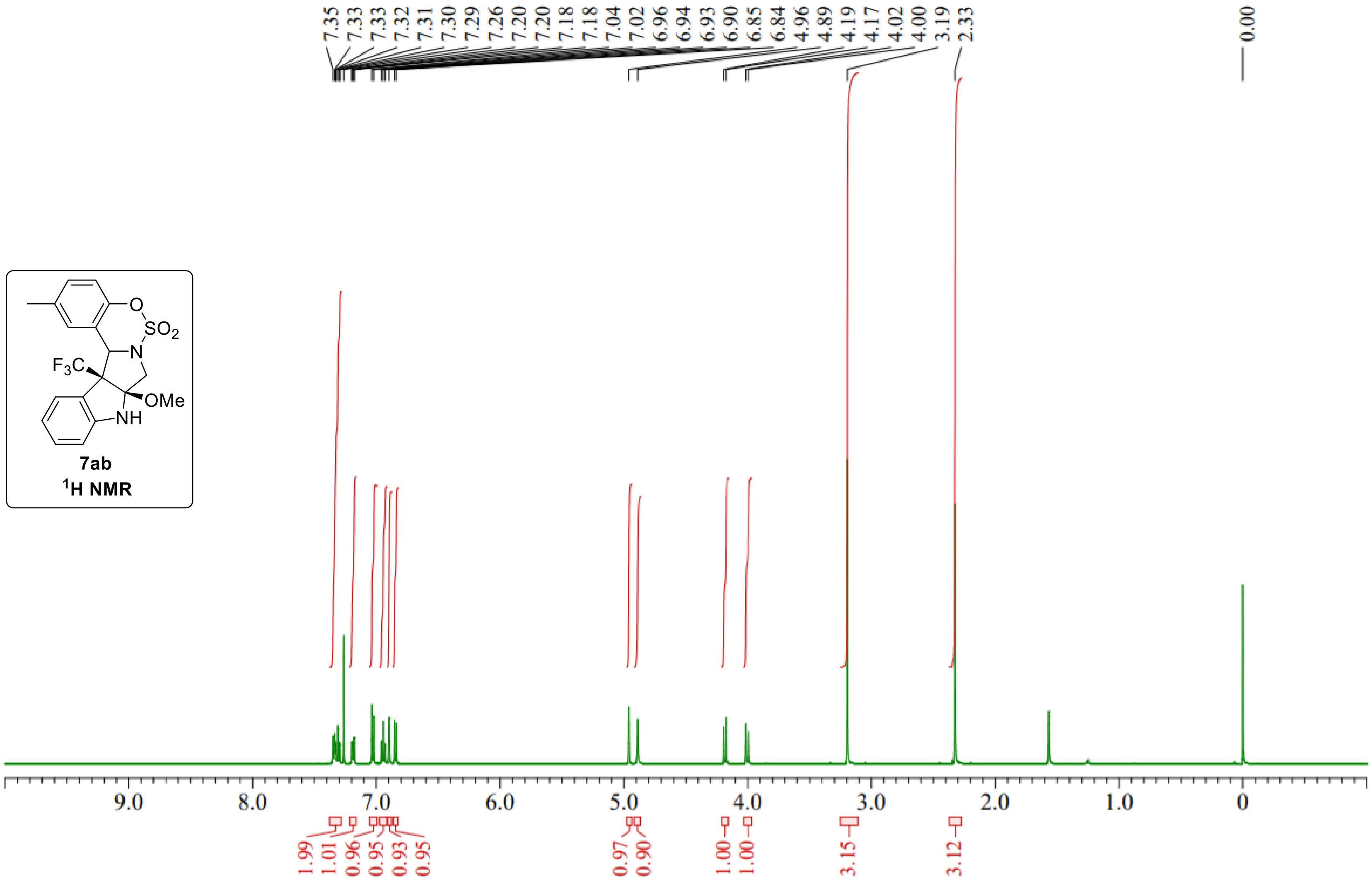

Supplementary Figure 94. ^{1}H NMR of **7ab**.

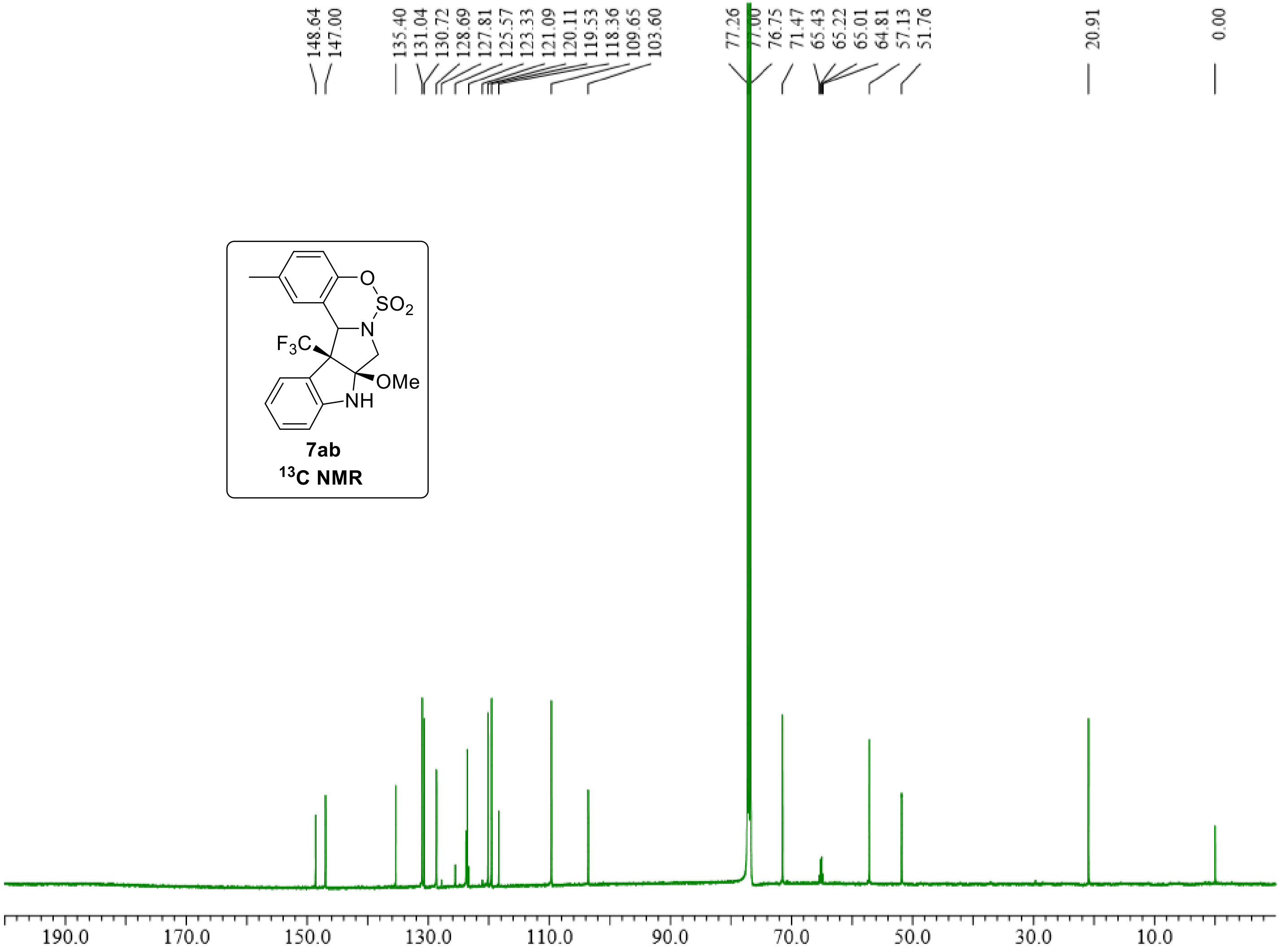

Supplementary Figure 95. ^{13}C NMR of **7ab**.

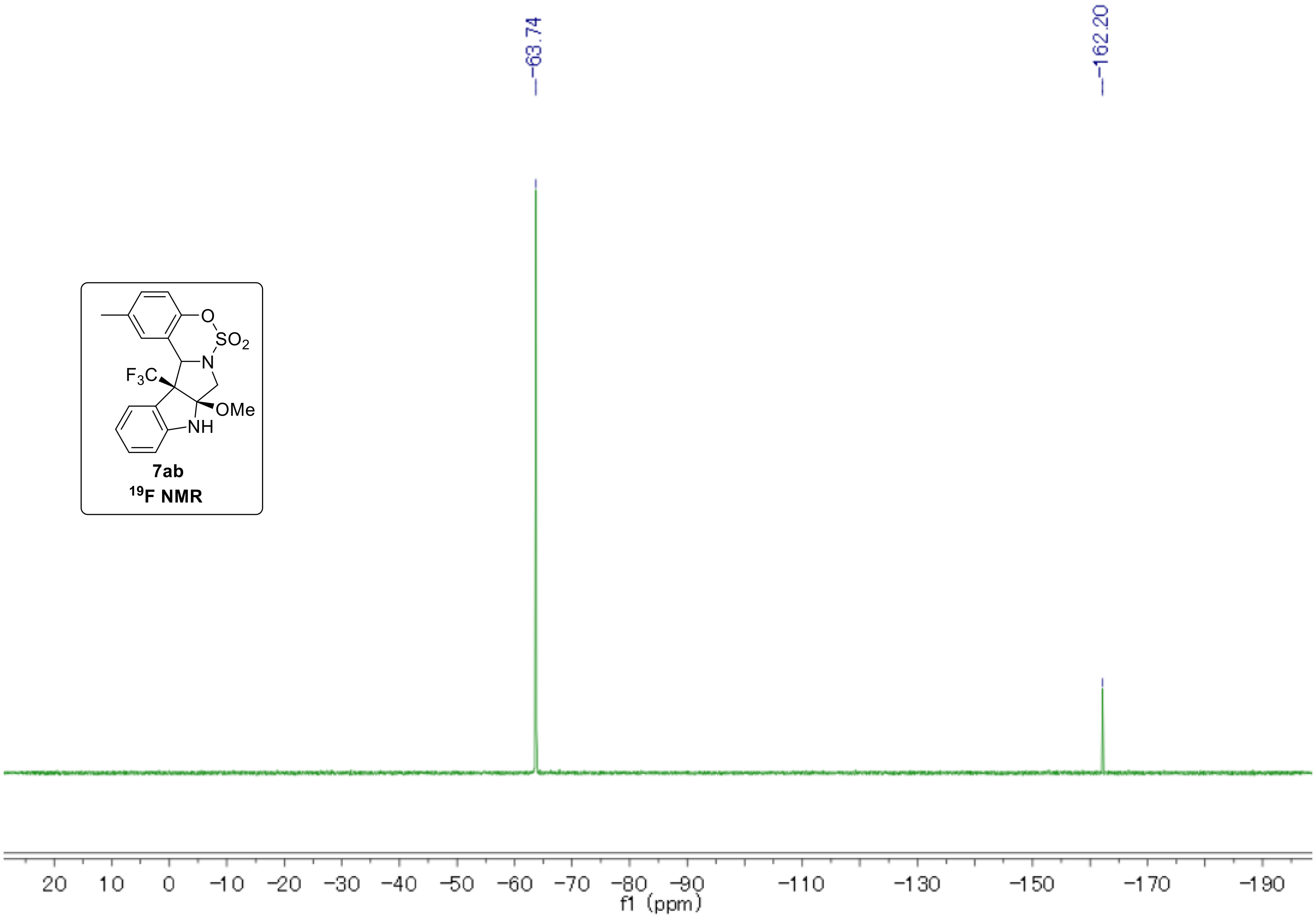

Supplementary Figure 96. ^{19}F NMR of **7ab**.

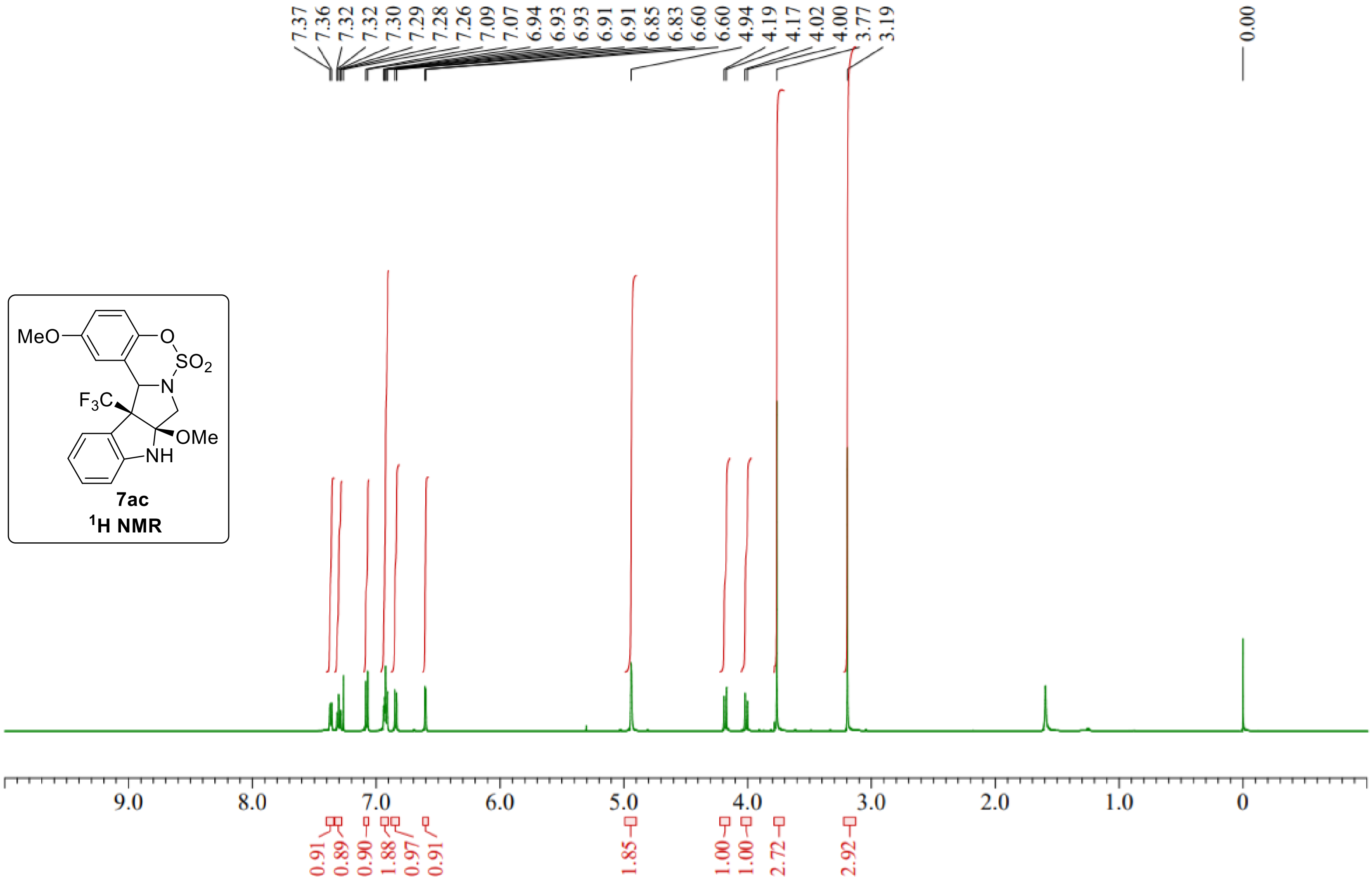

Supplementary Figure 97. ^{1}H NMR of **7ac**.

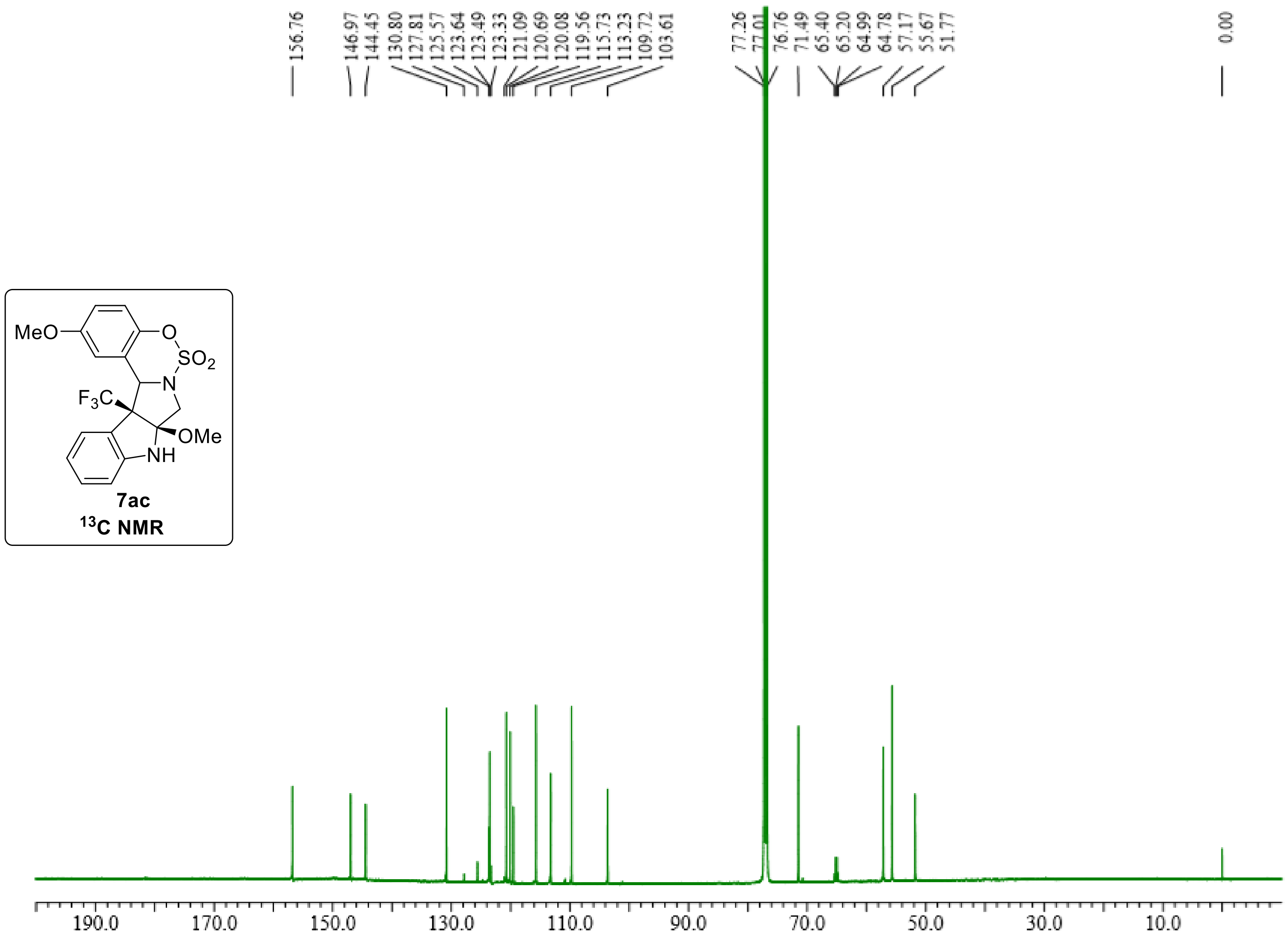

Supplementary Figure 98. ^{13}C NMR of **7ac**.

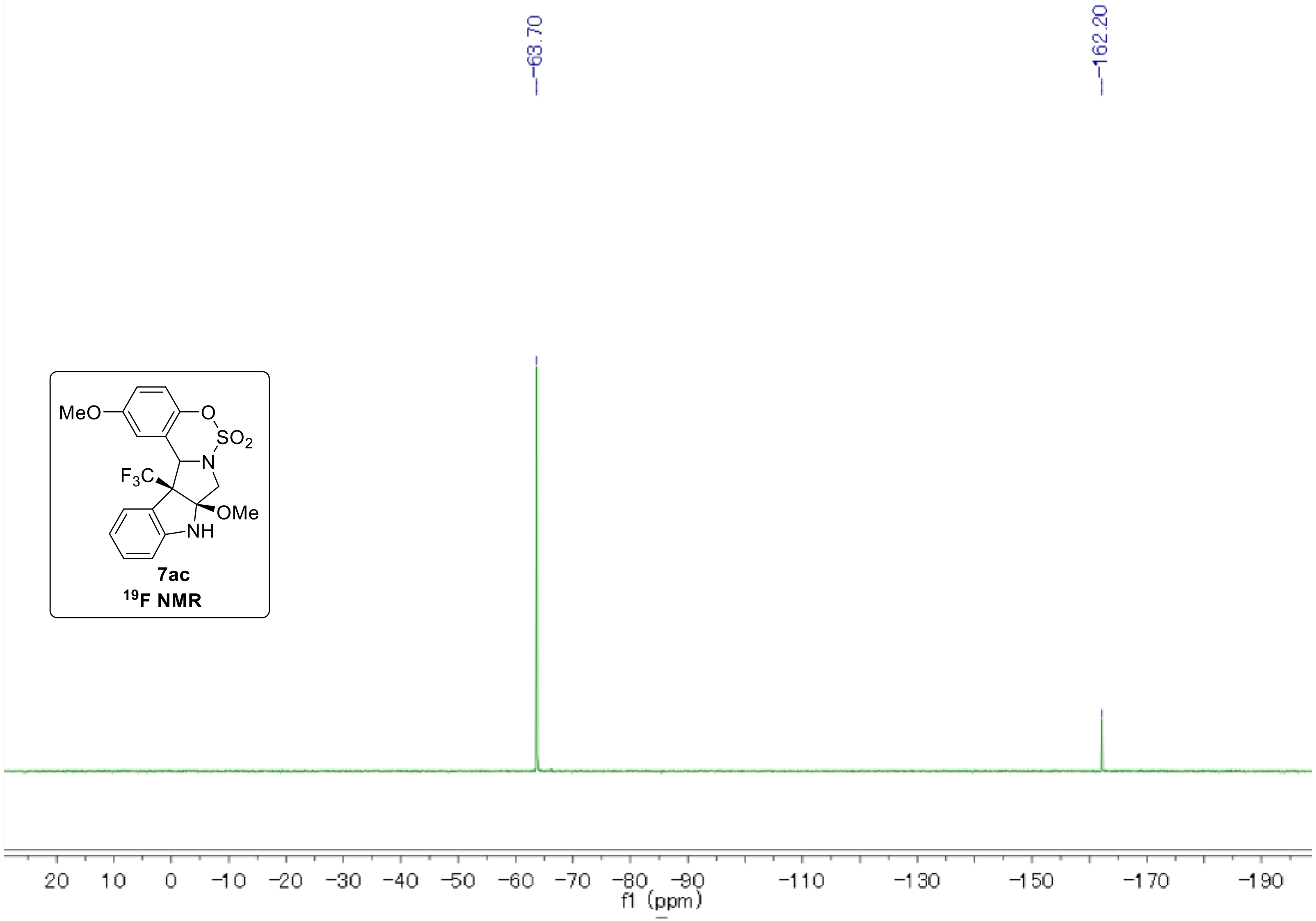

Supplementary Figure 99. ^{19}F NMR of **7ac**.

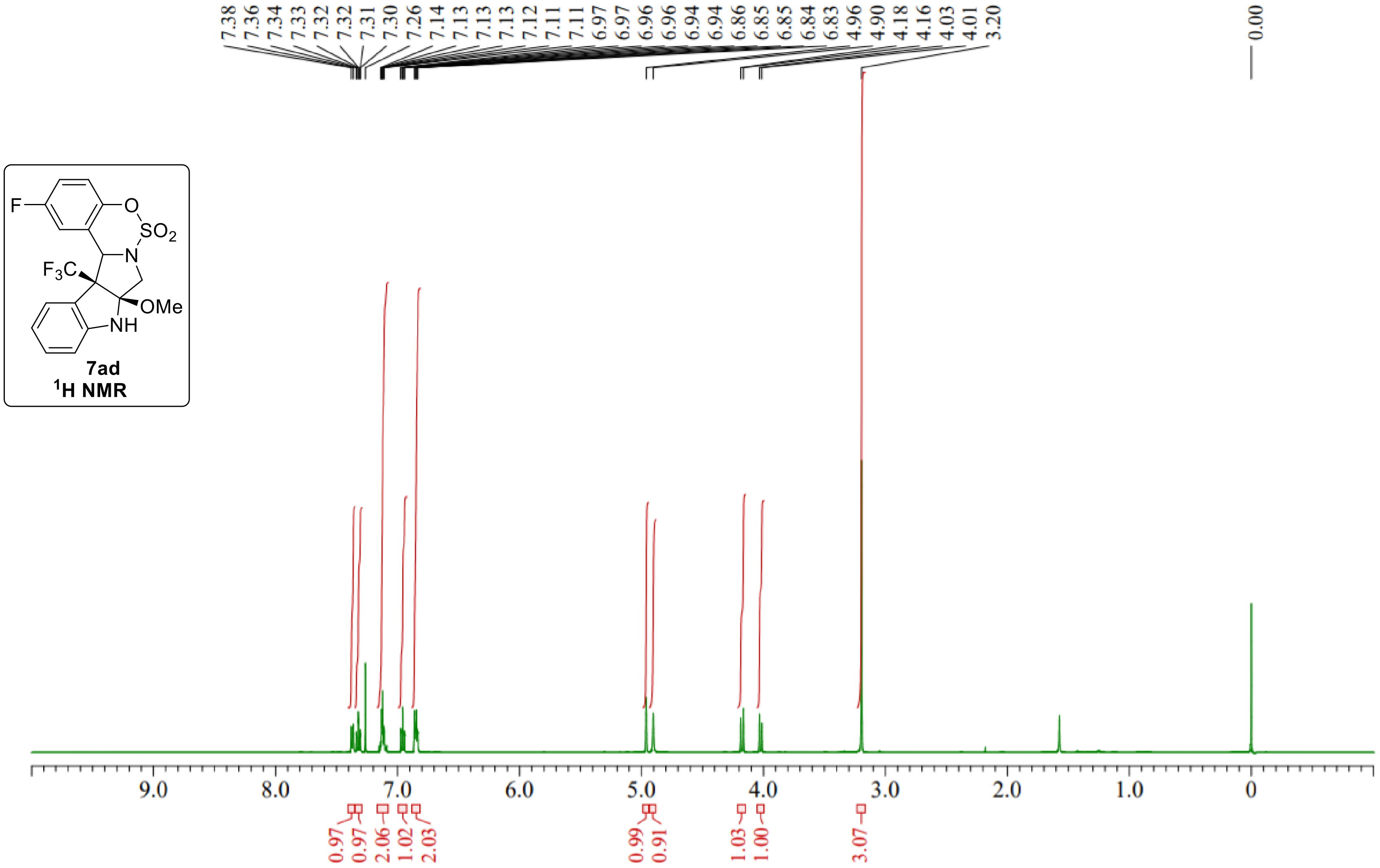

Supplementary Figure 100. ^{1}H NMR of **7ad**.

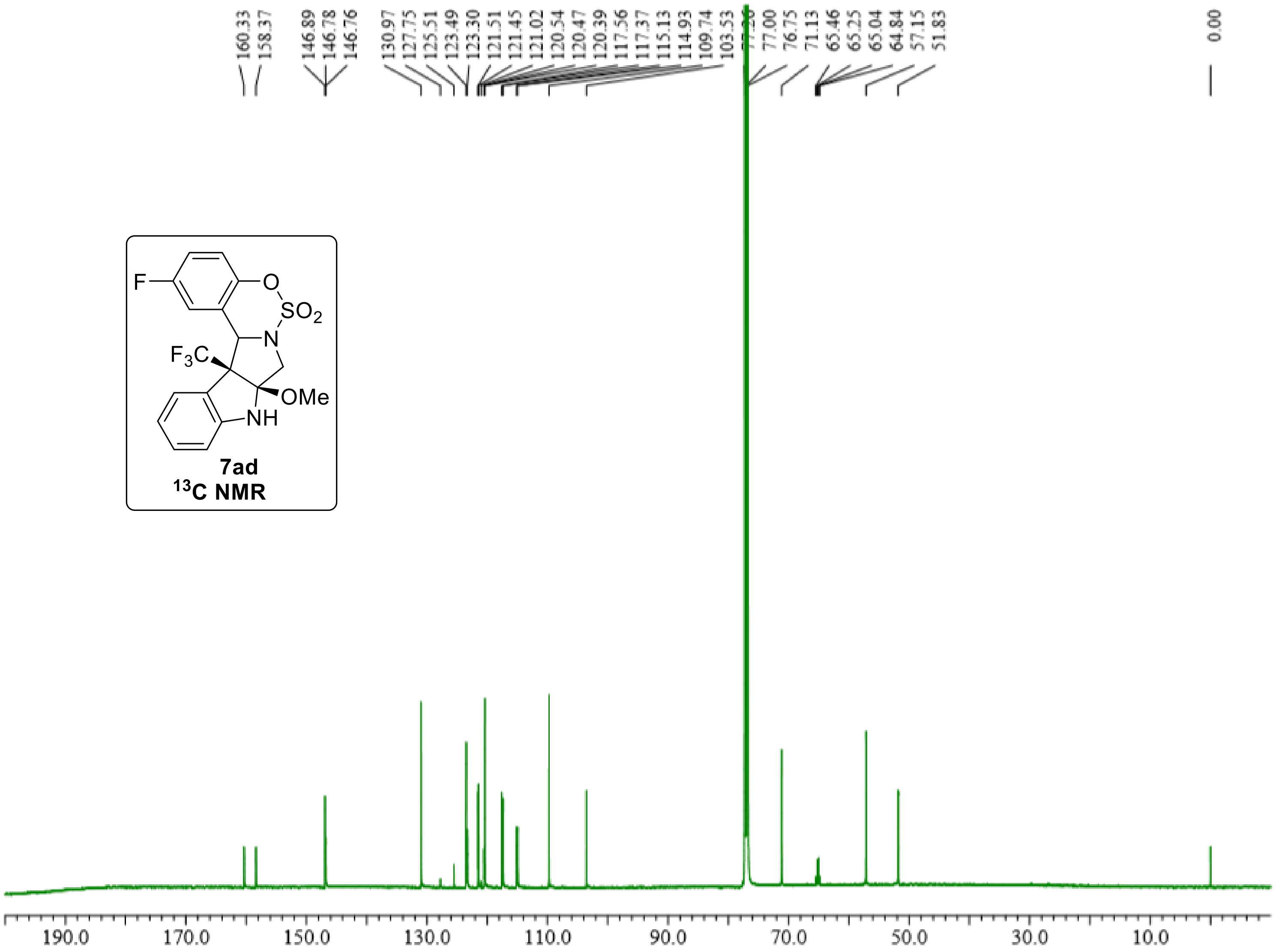

Supplementary Figure 101. ^{13}C NMR of **7ad**.

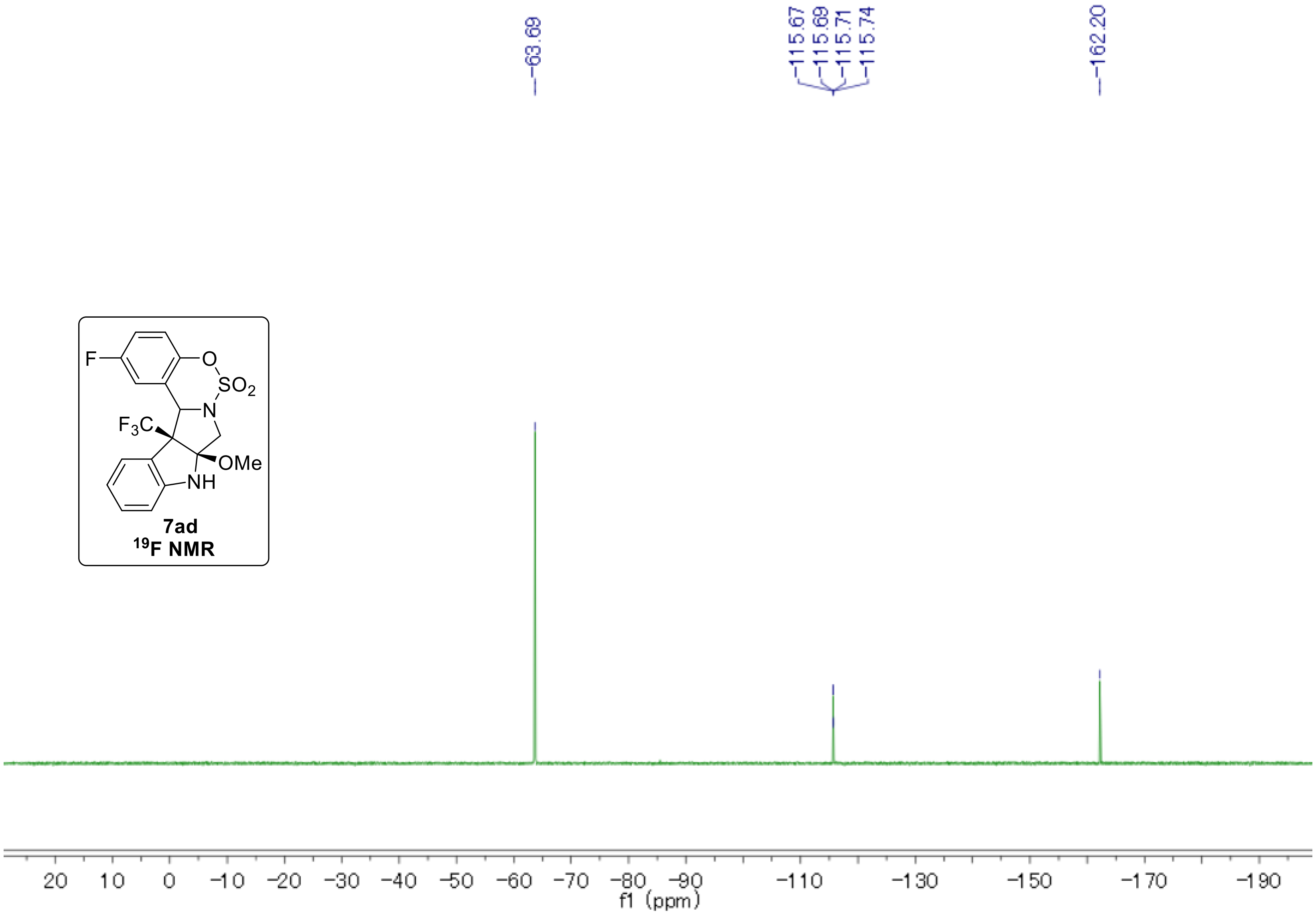

Supplementary Figure 102. ^{19}F NMR of **7ad**.

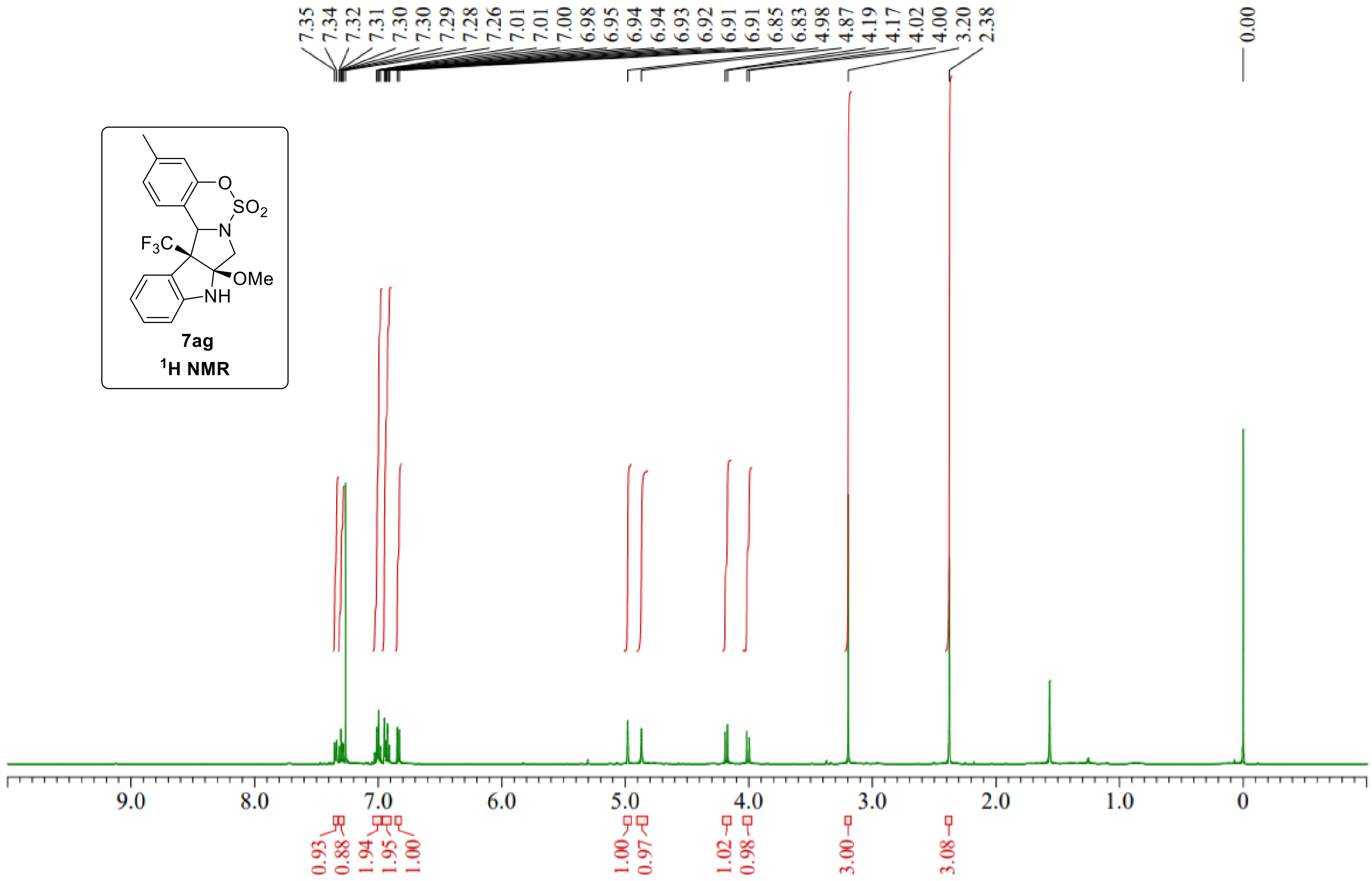

Supplementary Figure 103. ^{1}H NMR of **7ag**.

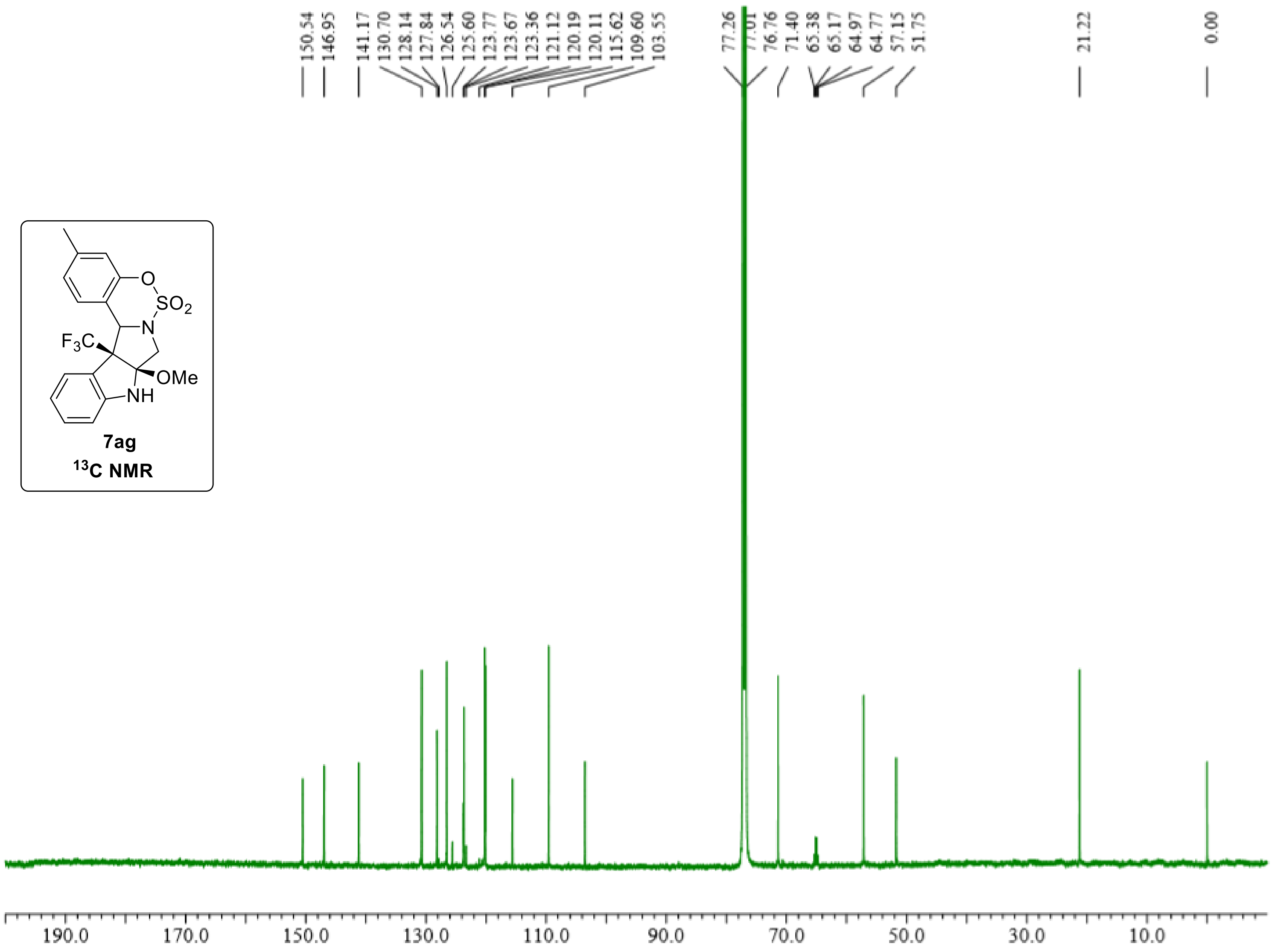

Supplementary Figure 104. ^{13}C NMR of **7ag**.

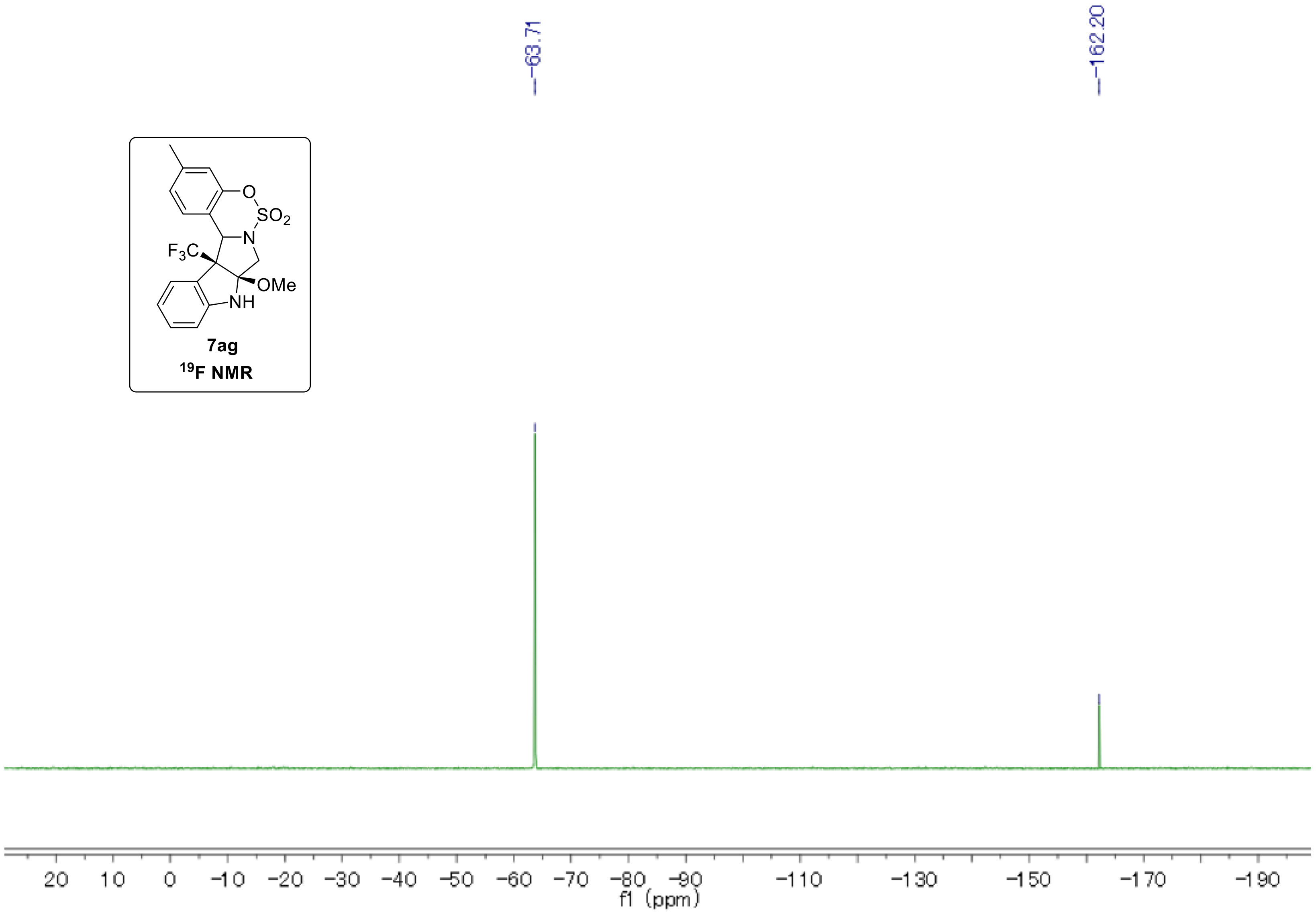

Supplementary Figure 105. ^{19}F NMR of **7ag**.

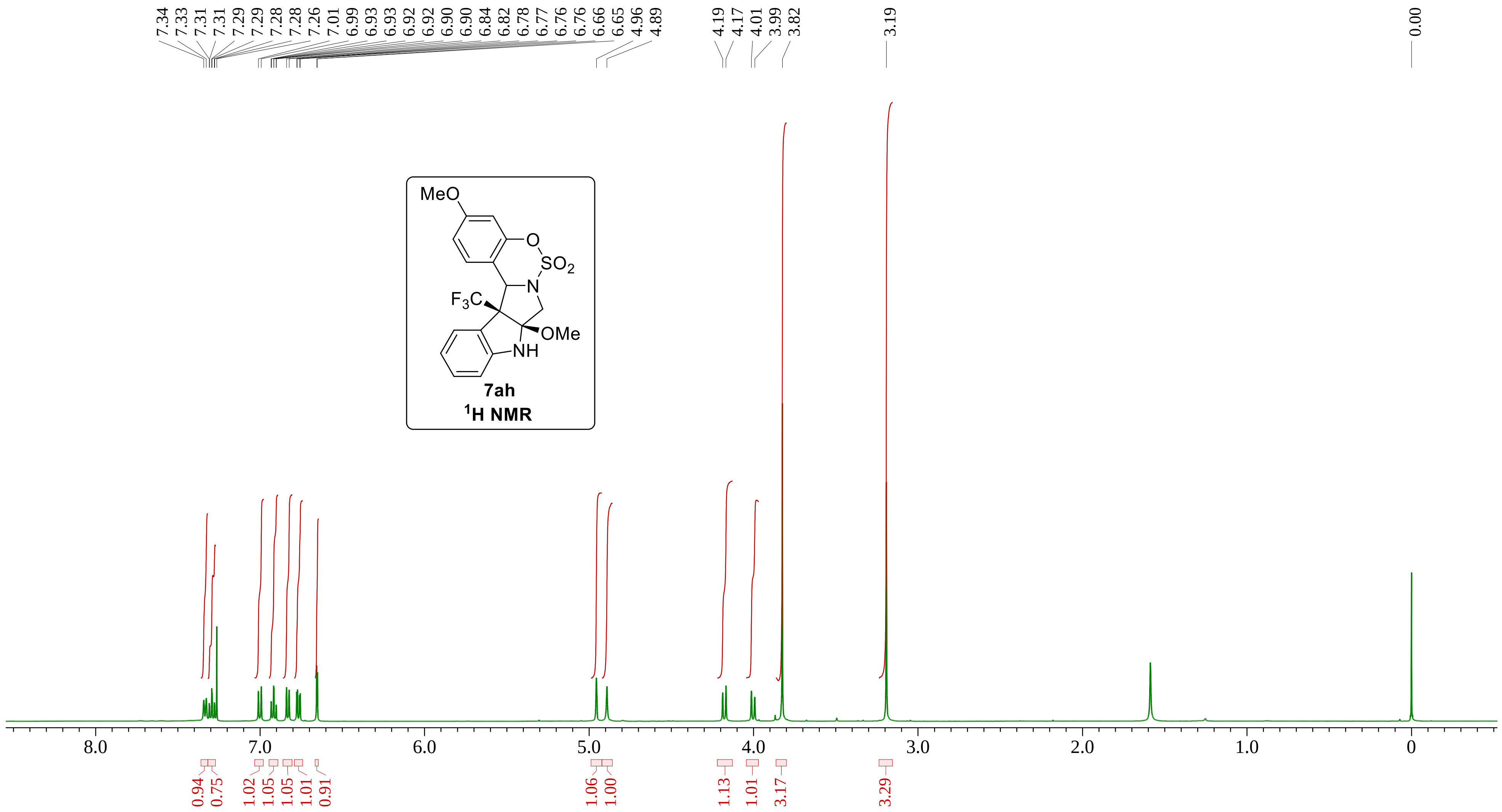

Supplementary Figure 106. ^{1}H NMR of **7ah**.

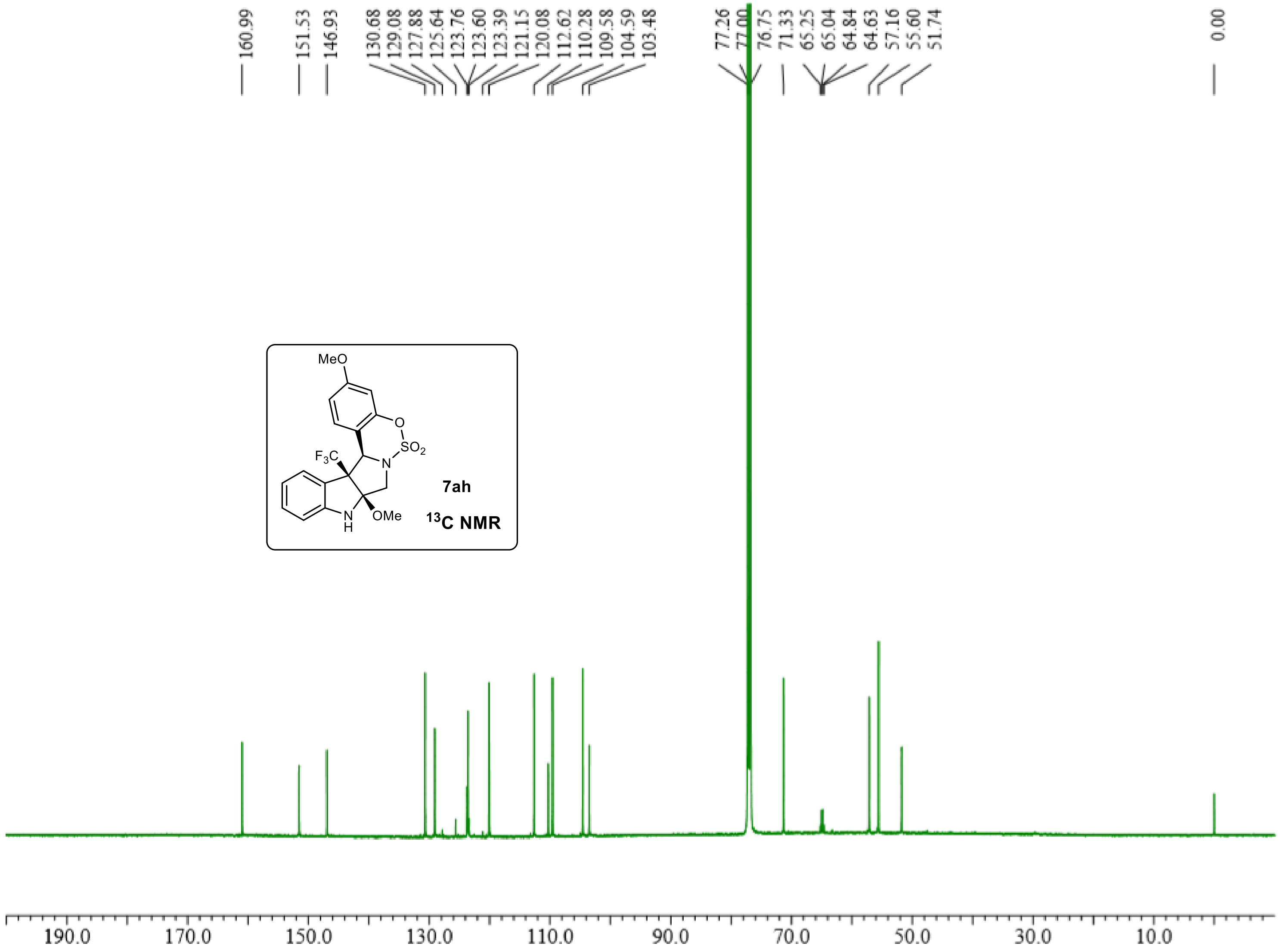

Supplementary Figure 107. ^{13}C NMR of **7ah**.

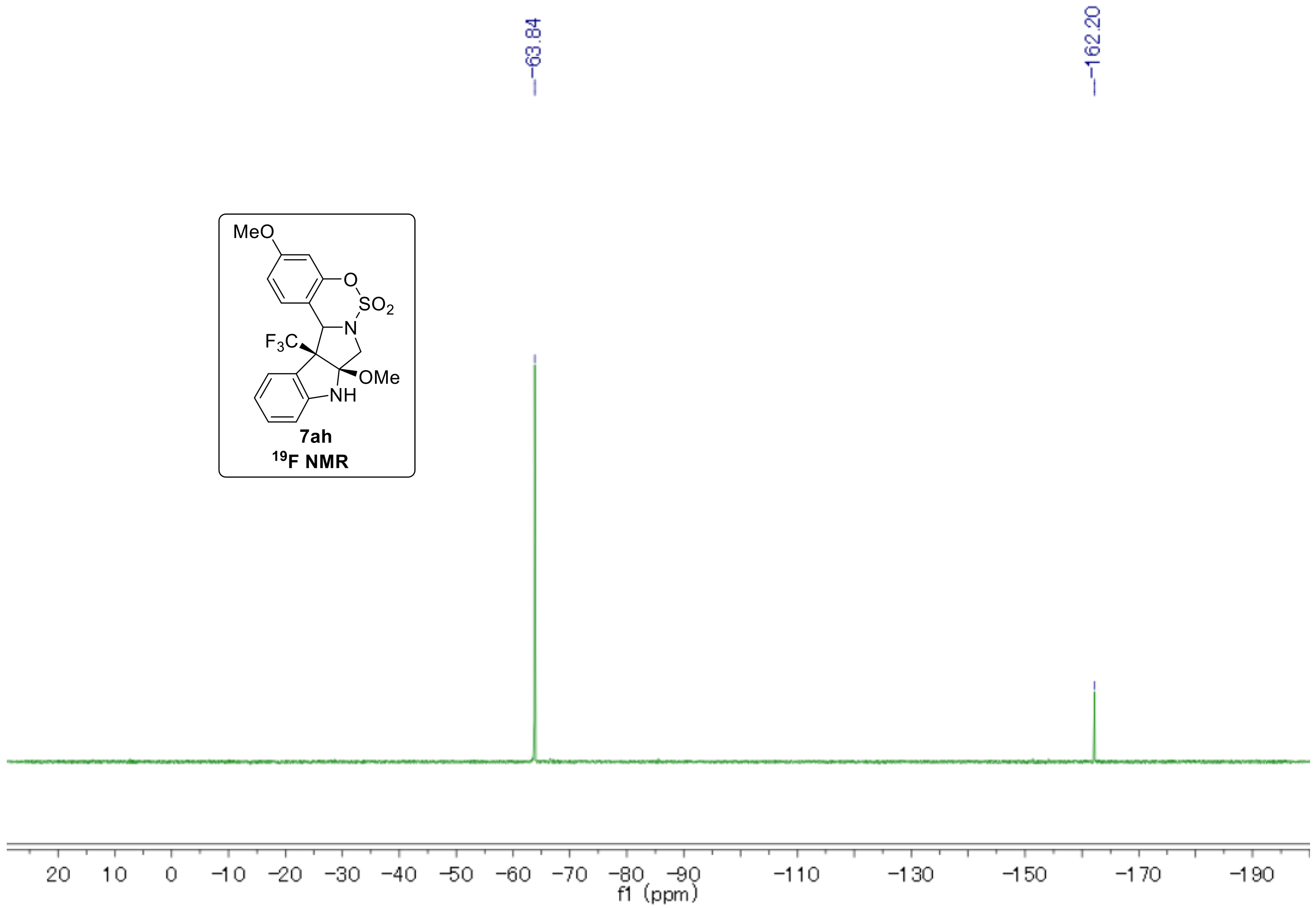

Supplementary Figure 108. ^{19}F NMR of **7ah**.

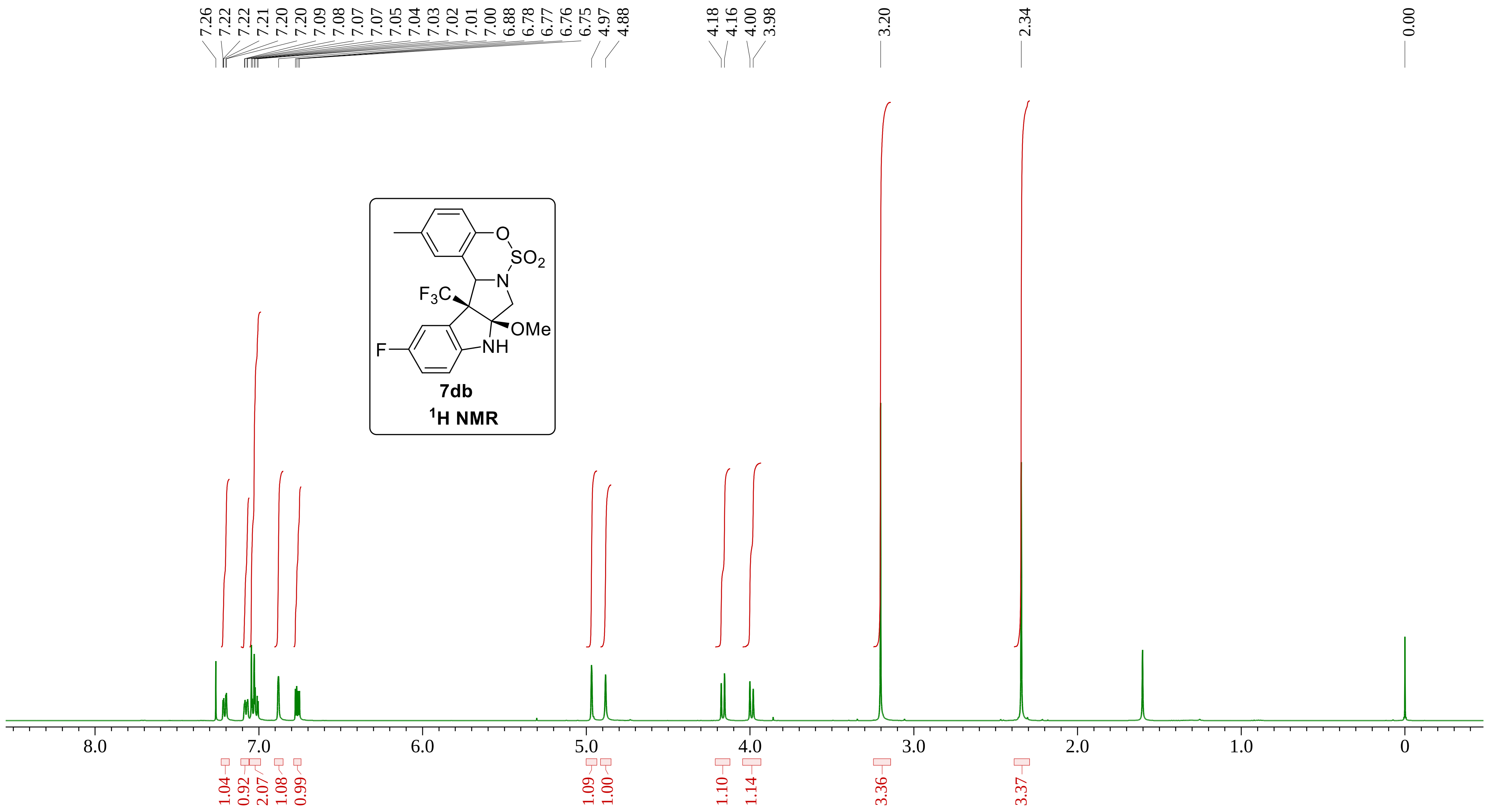

Supplementary Figure 109. ^{1}H NMR of **7db**.

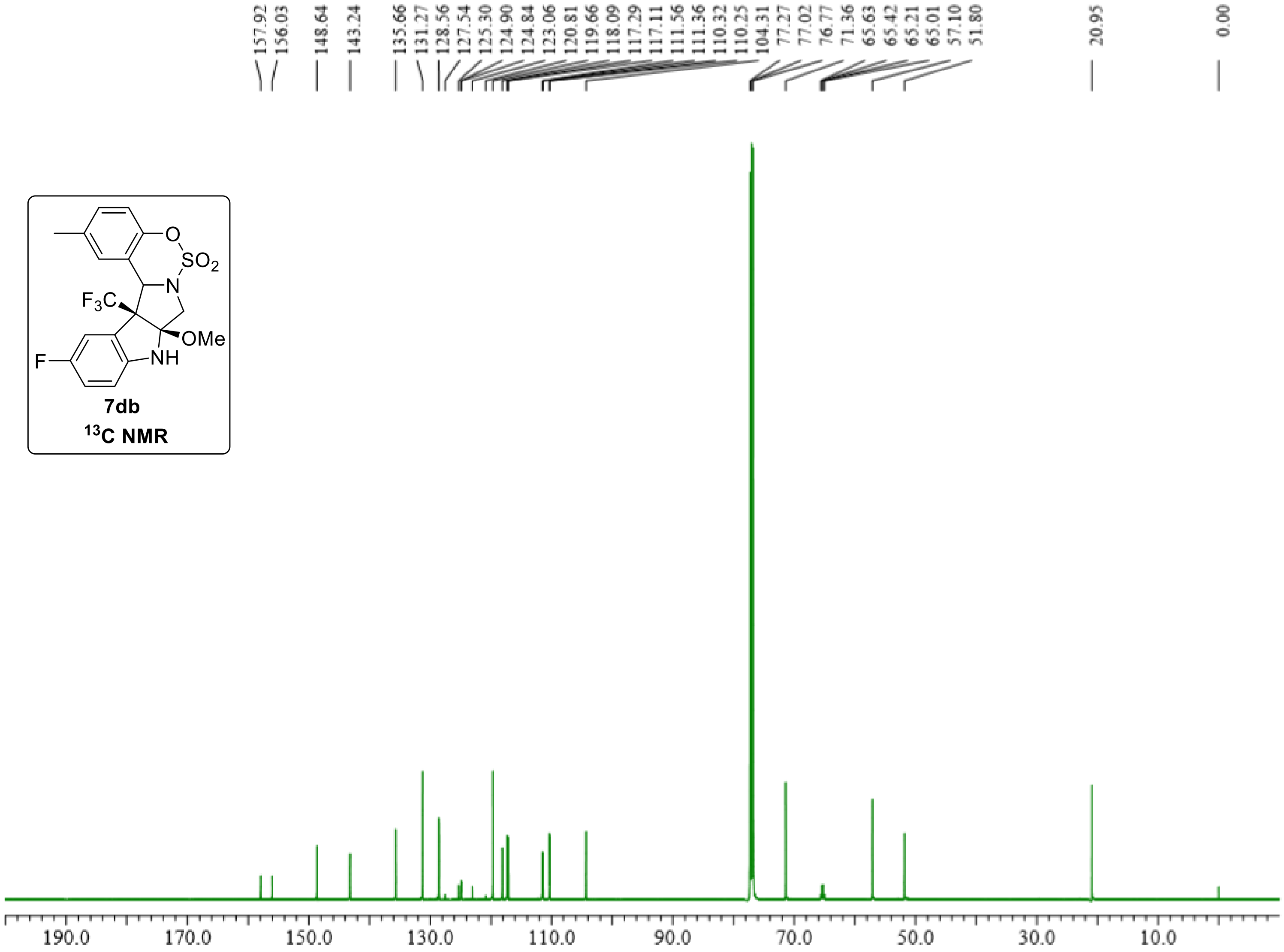

Supplementary Figure 110. ^{13}C NMR of **7db**.

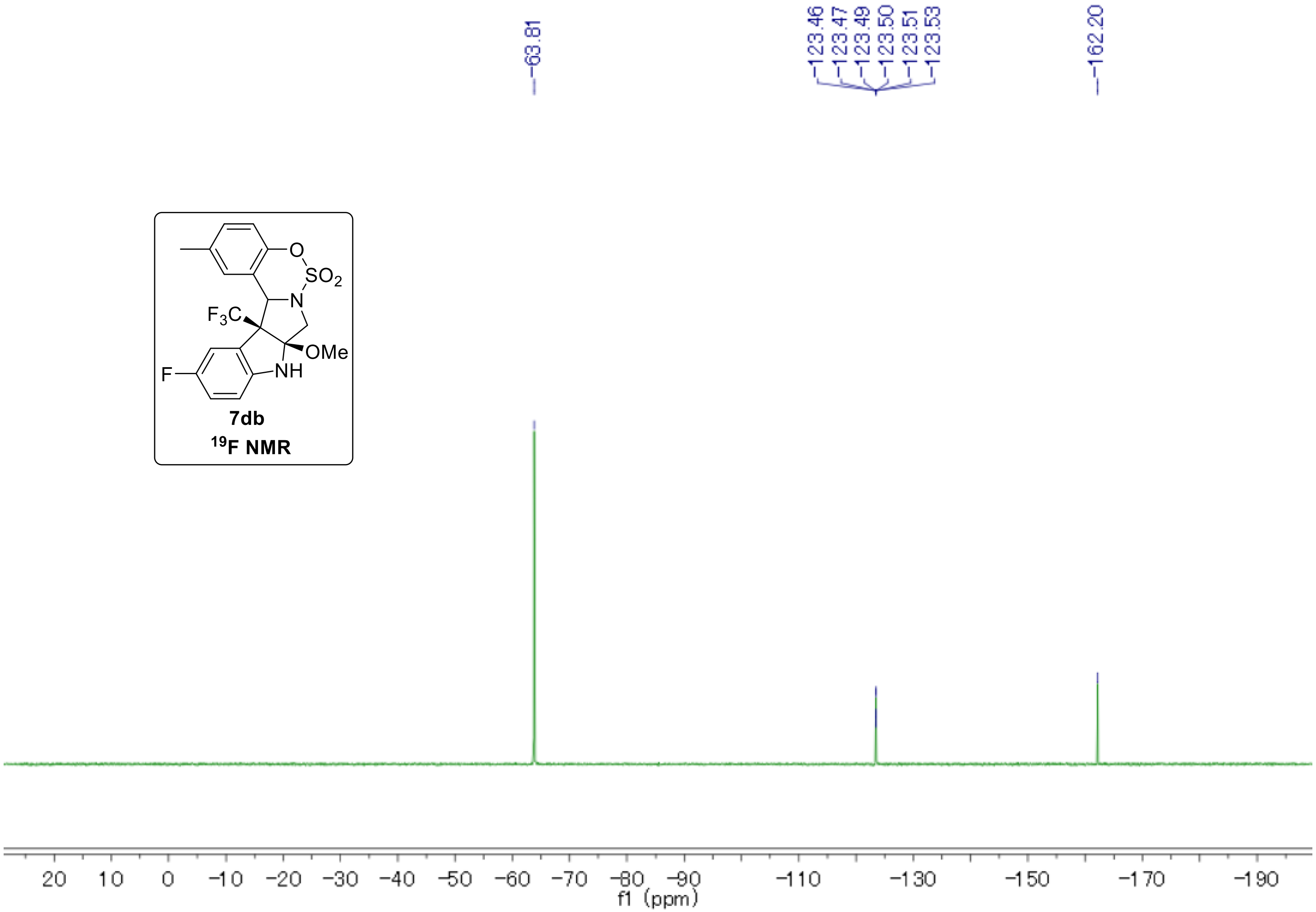

Supplementary Figure 111. ^{19}F NMR of **7db**.

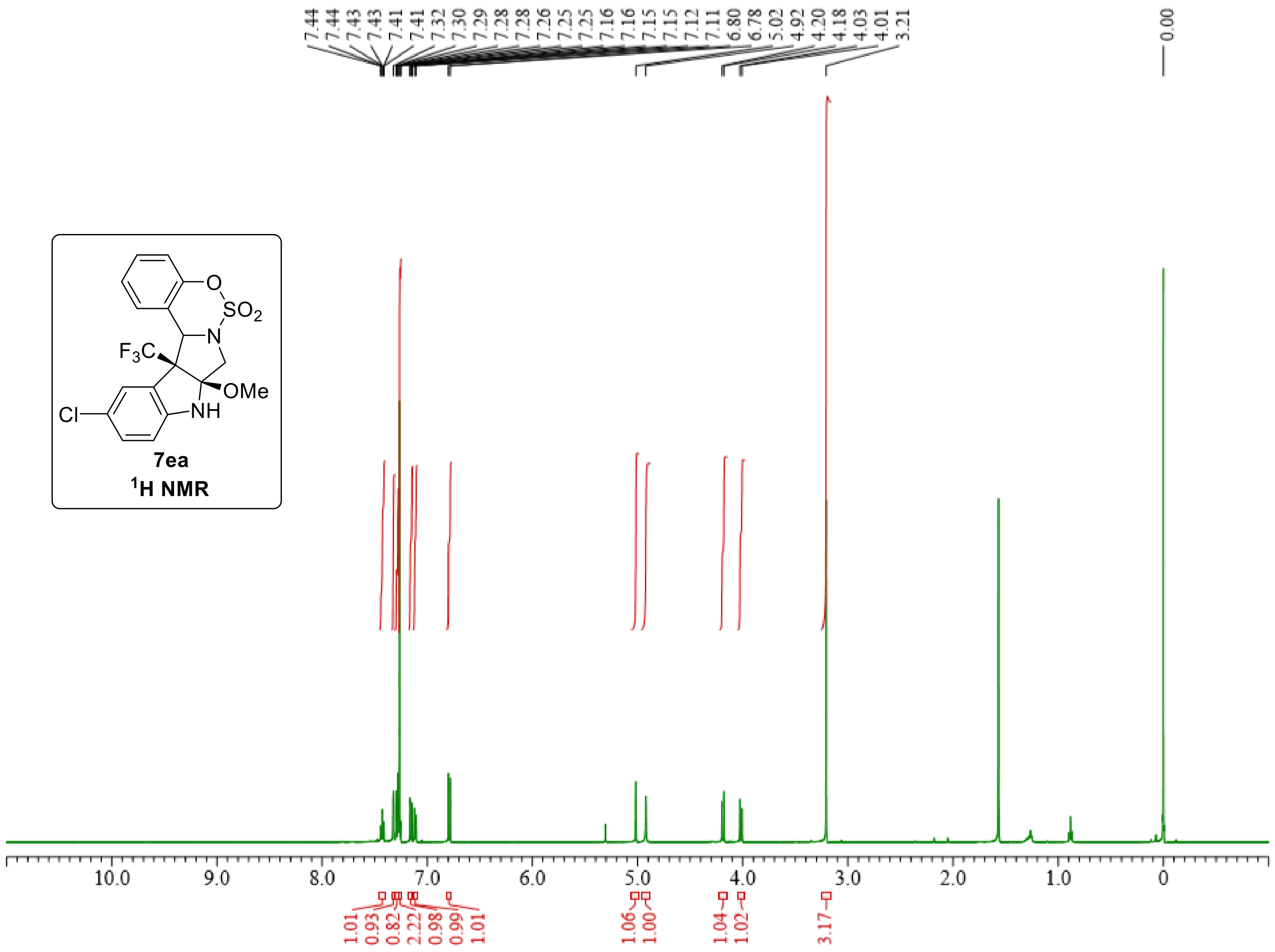

Supplementary Figure 112. ^{1}H NMR of **7ea**.

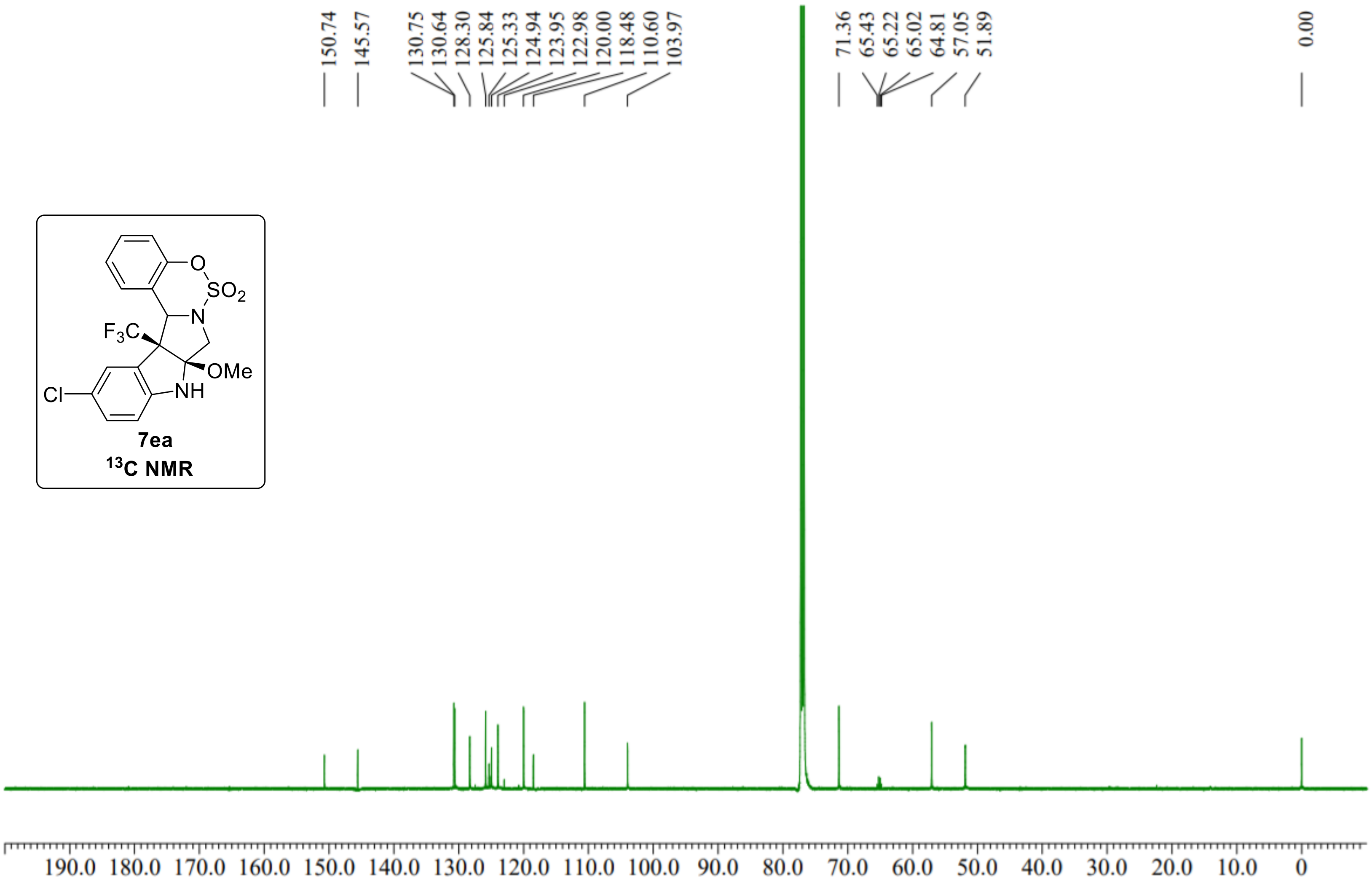

Supplementary Figure 113. ^{13}C NMR of **7ea**.

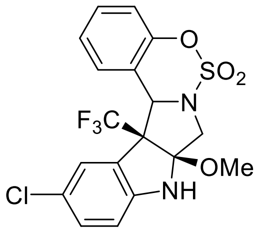

7ea

^{19}F NMR

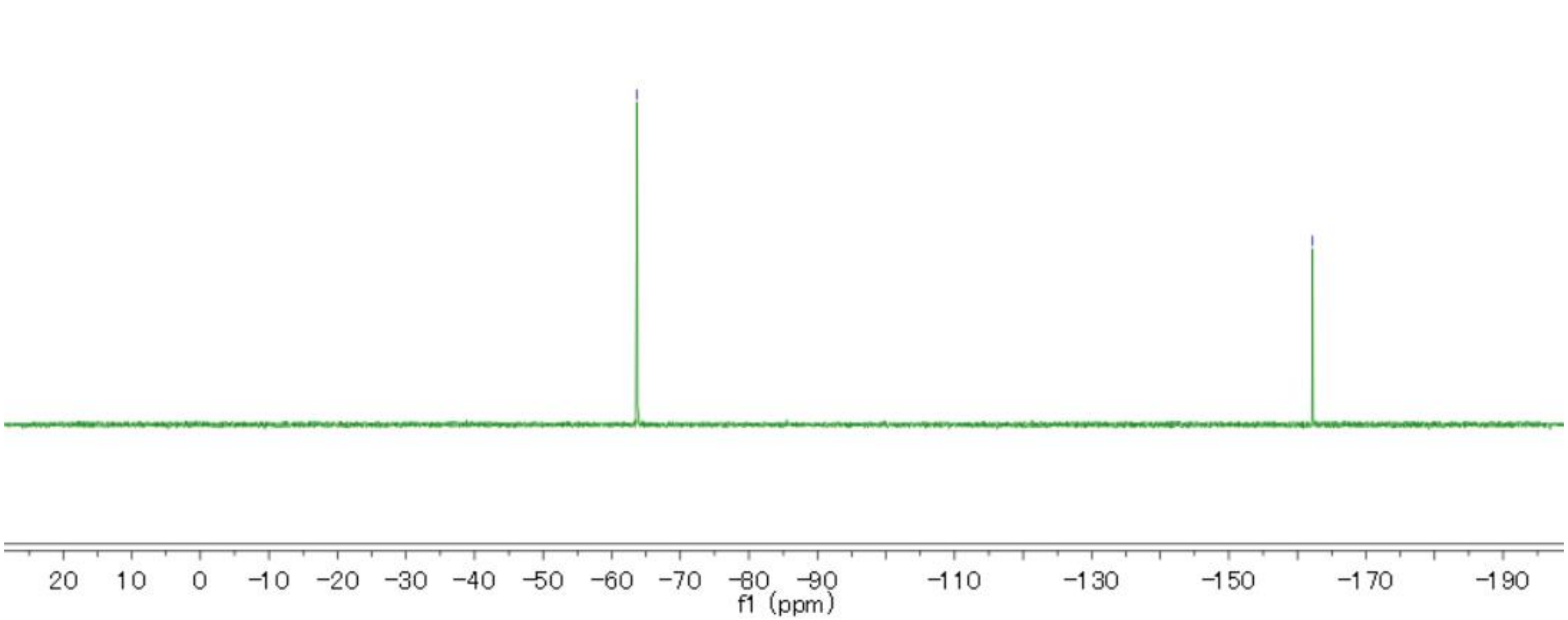

Supplementary Figure 114. ^{19}F NMR of **7ea**.

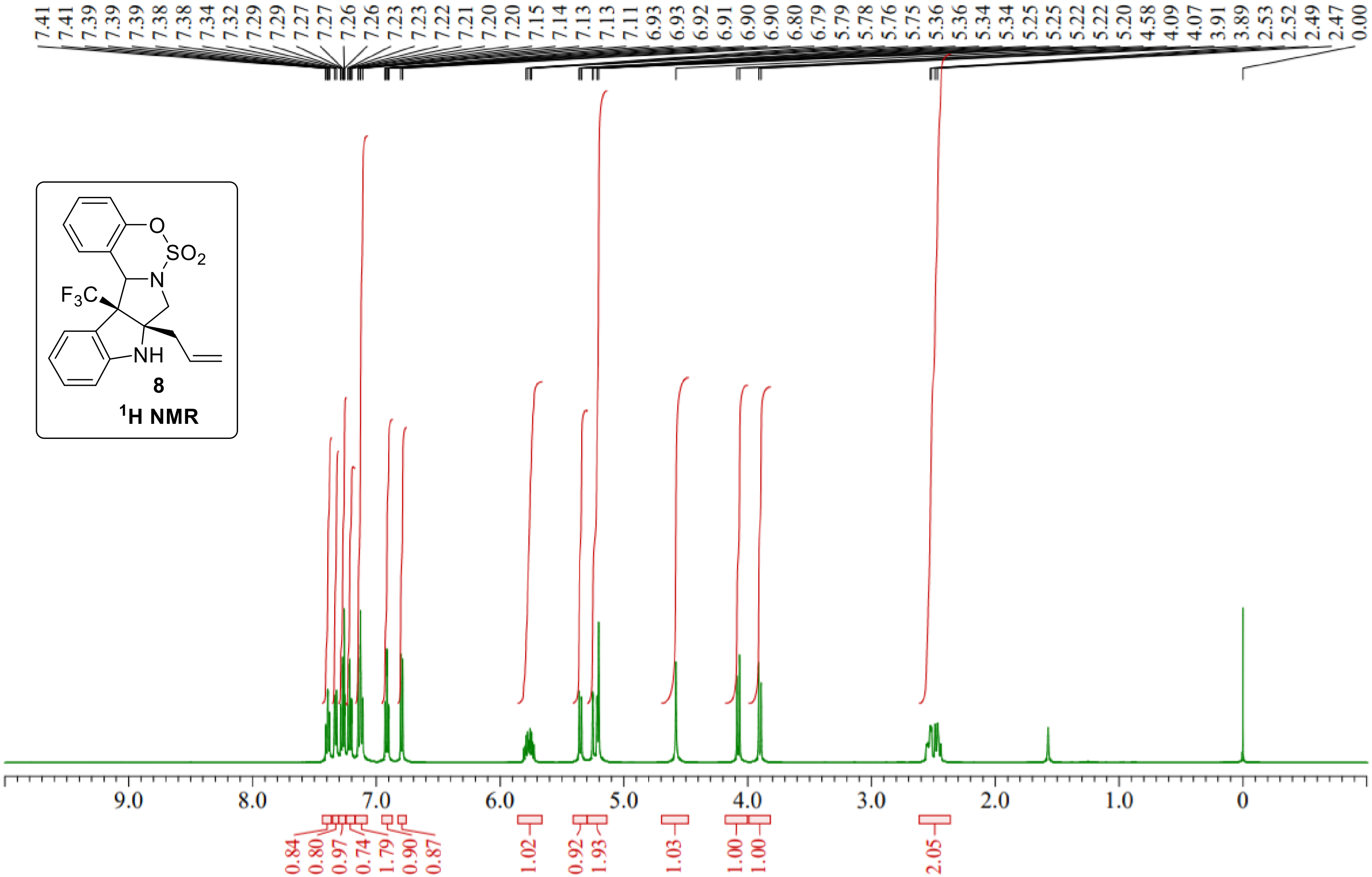

Supplementary Figure 115. ^{1}H NMR of **8**.

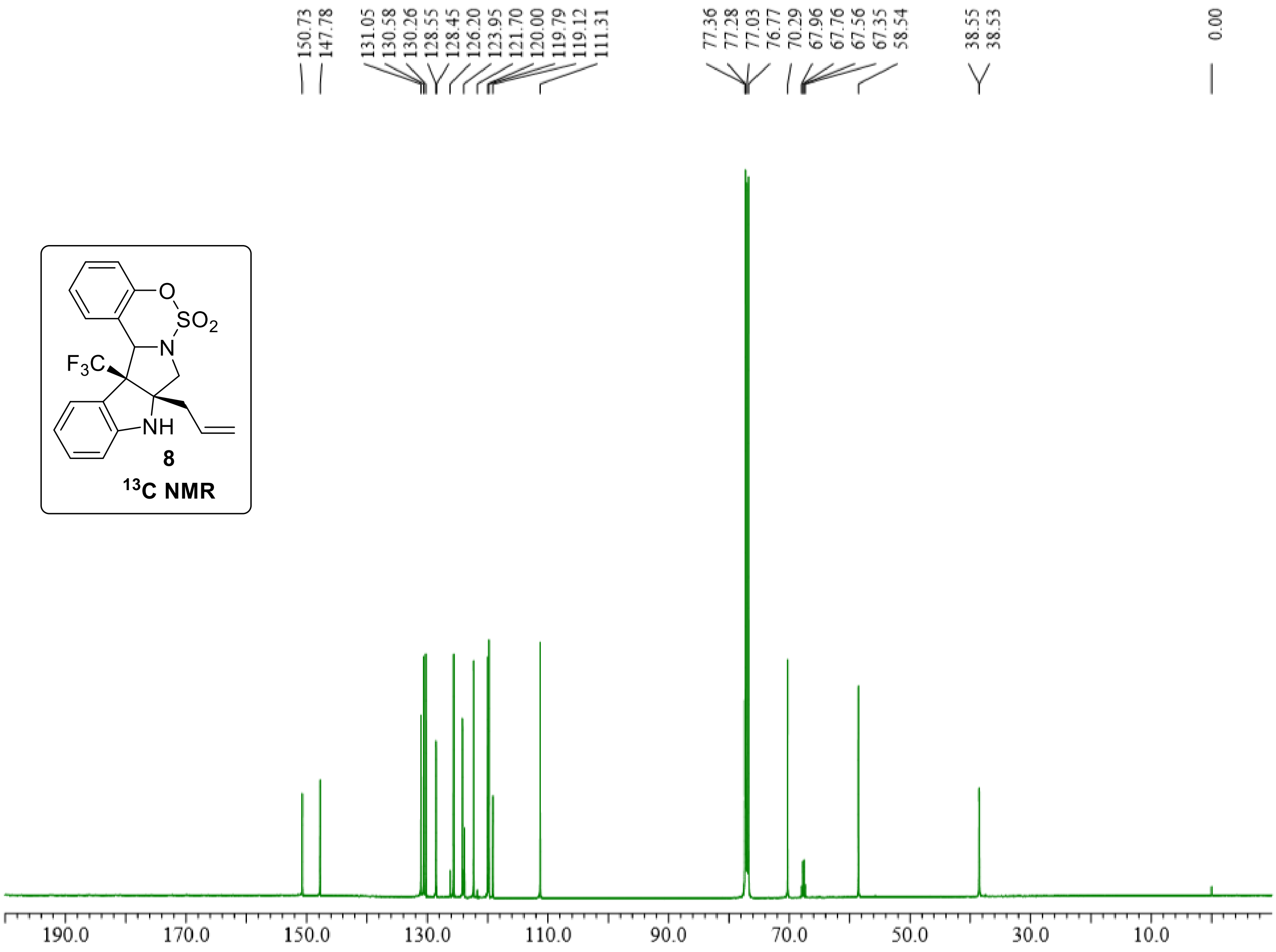

Supplementary Figure 116. ^{13}C NMR of **8**.

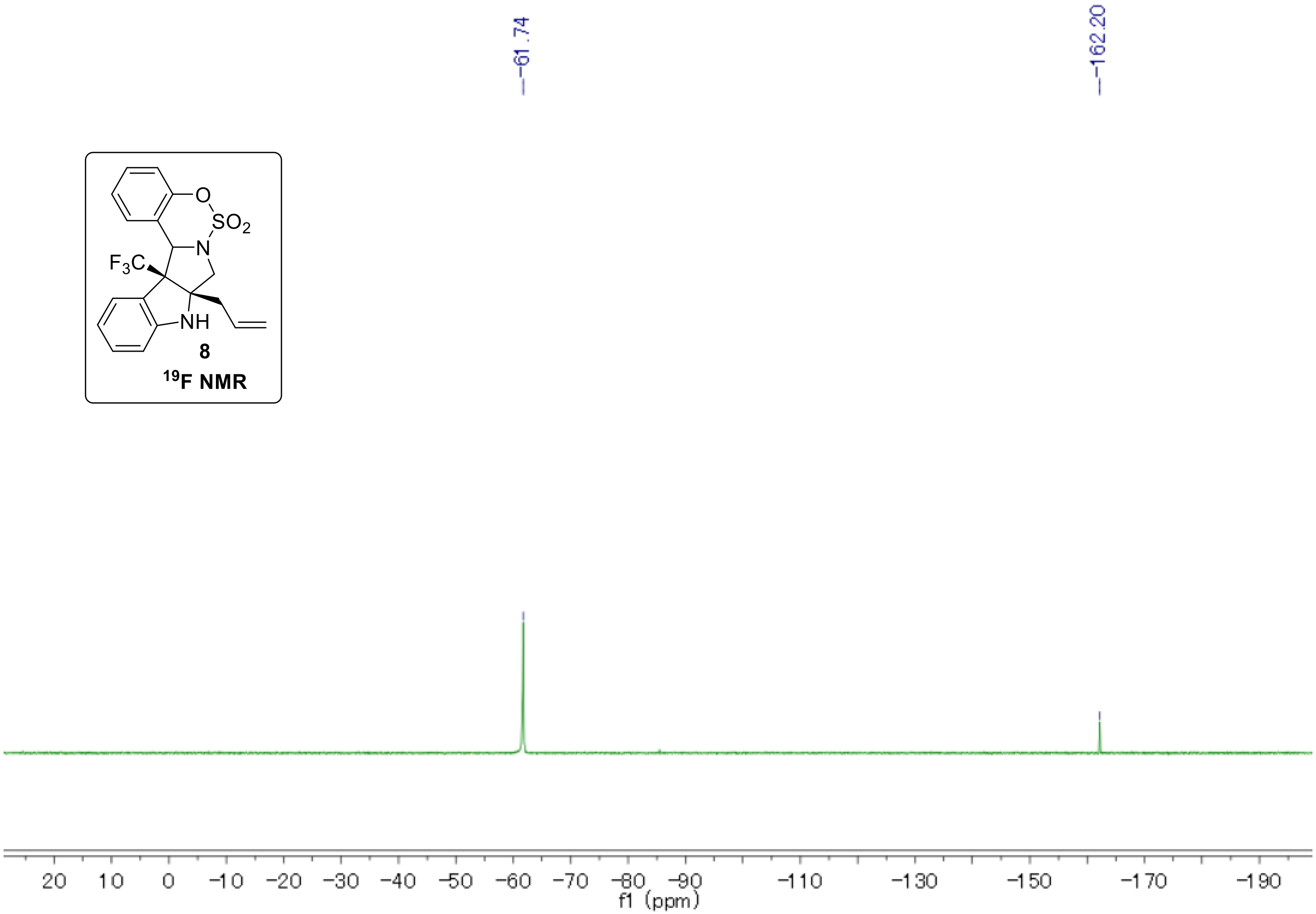

Supplementary Figure 117. ^{19}F NMR of **8**.

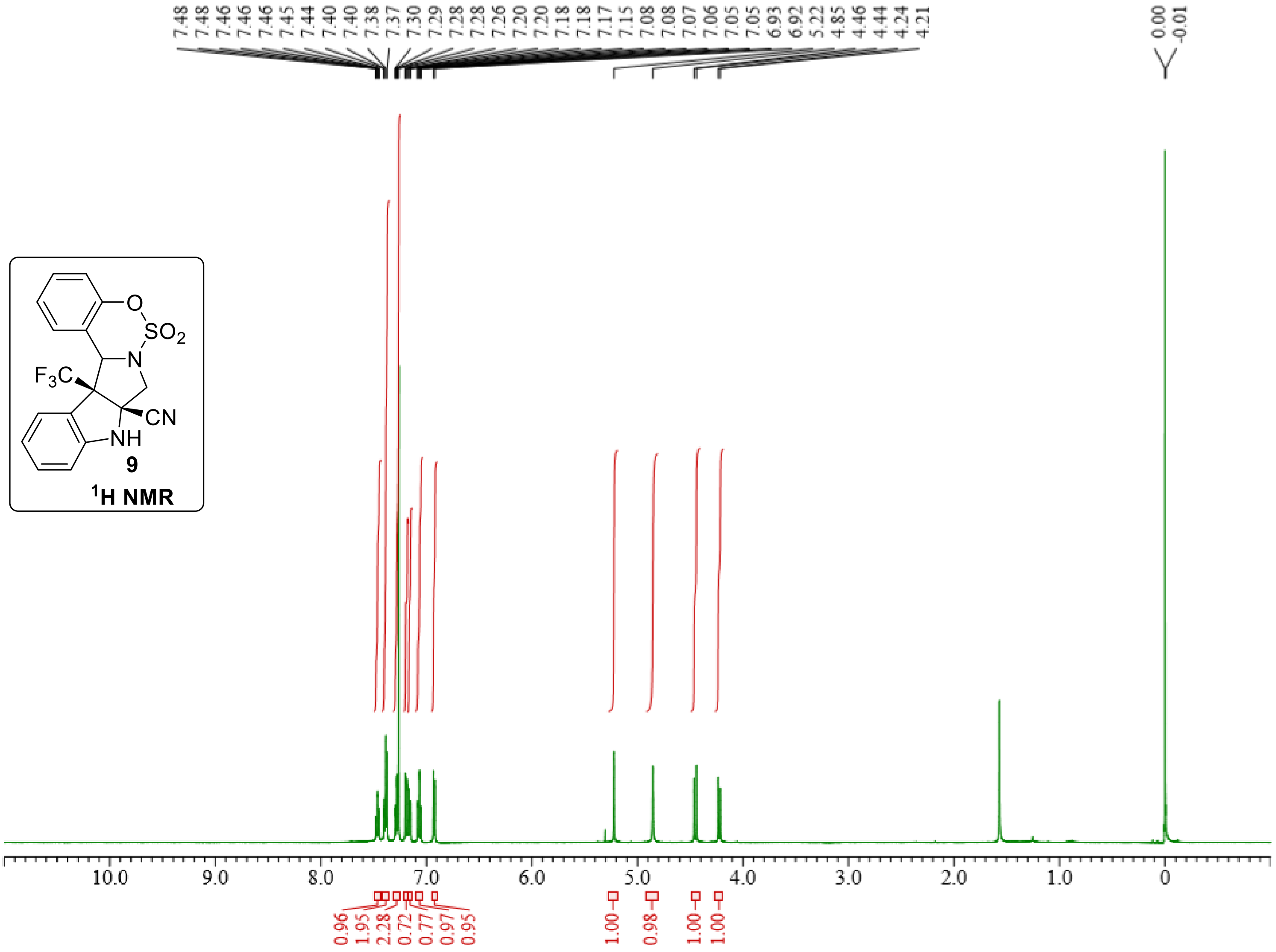

Supplementary Figure 118. ^{1}H NMR of **9**.

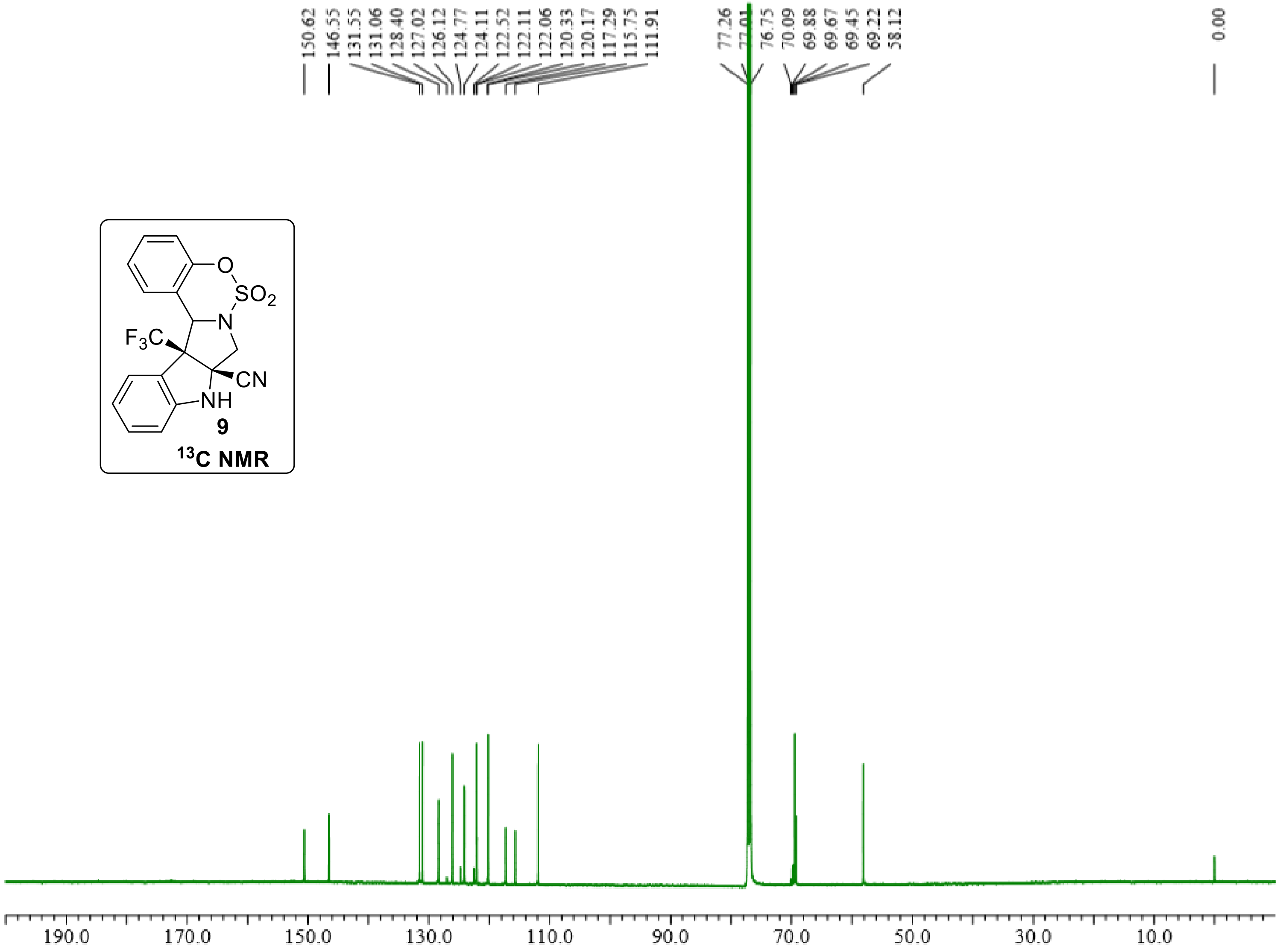

Supplementary Figure 119. ^{13}C NMR of **9**.

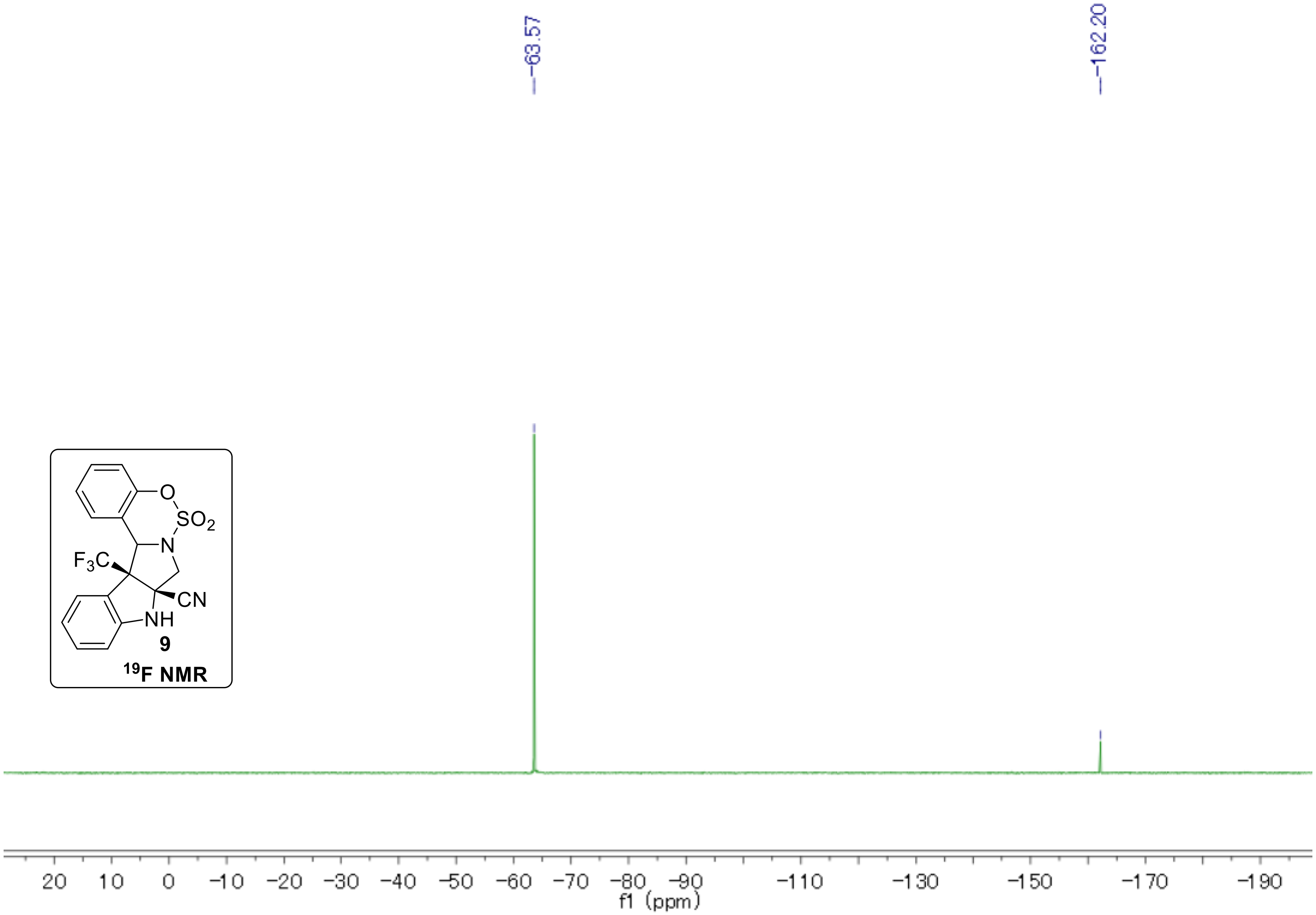

Supplementary Figure 120. ^{19}F NMR of **9**.

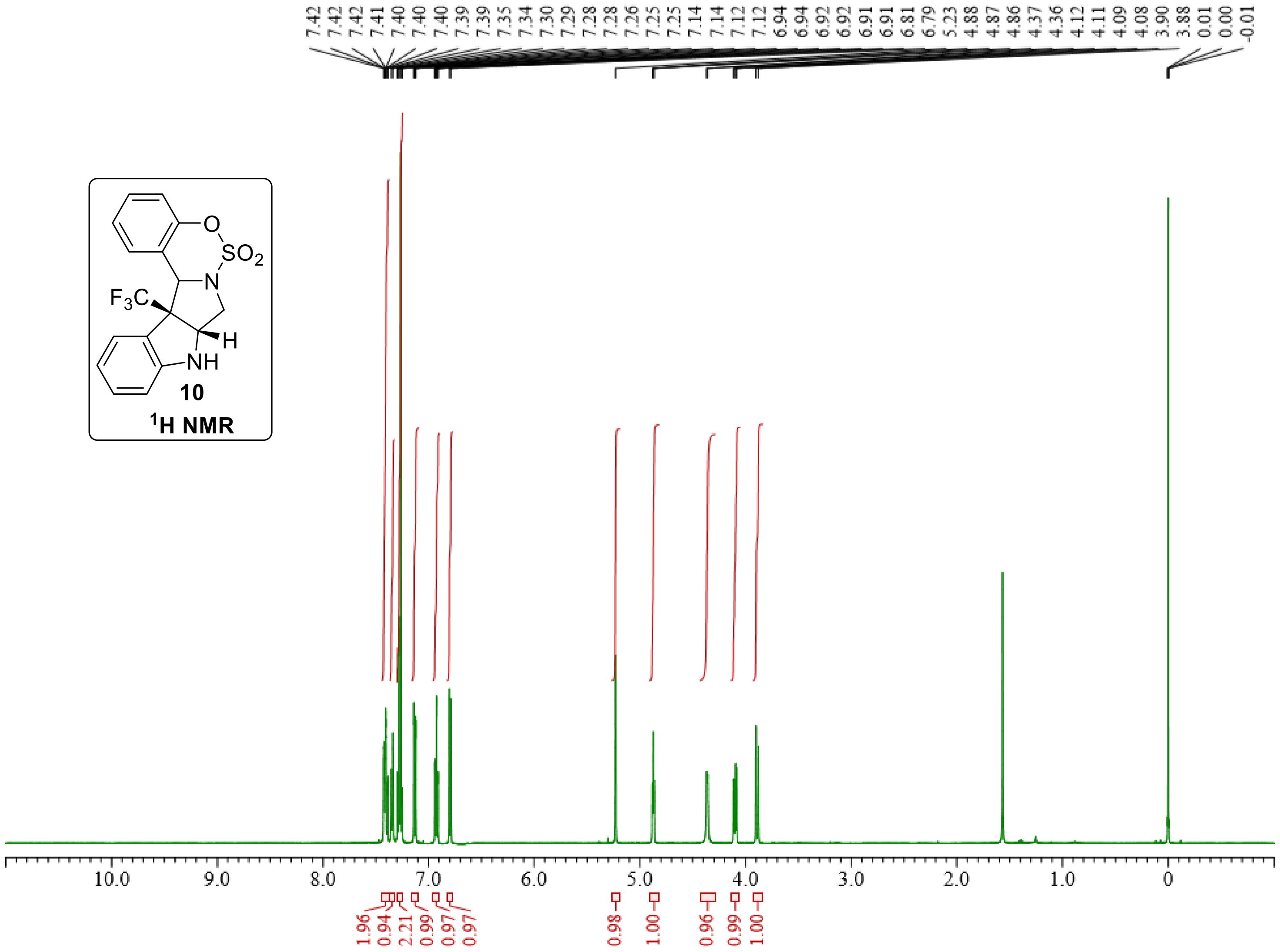

Supplementary Figure 121. ^{1}H NMR of **10**.

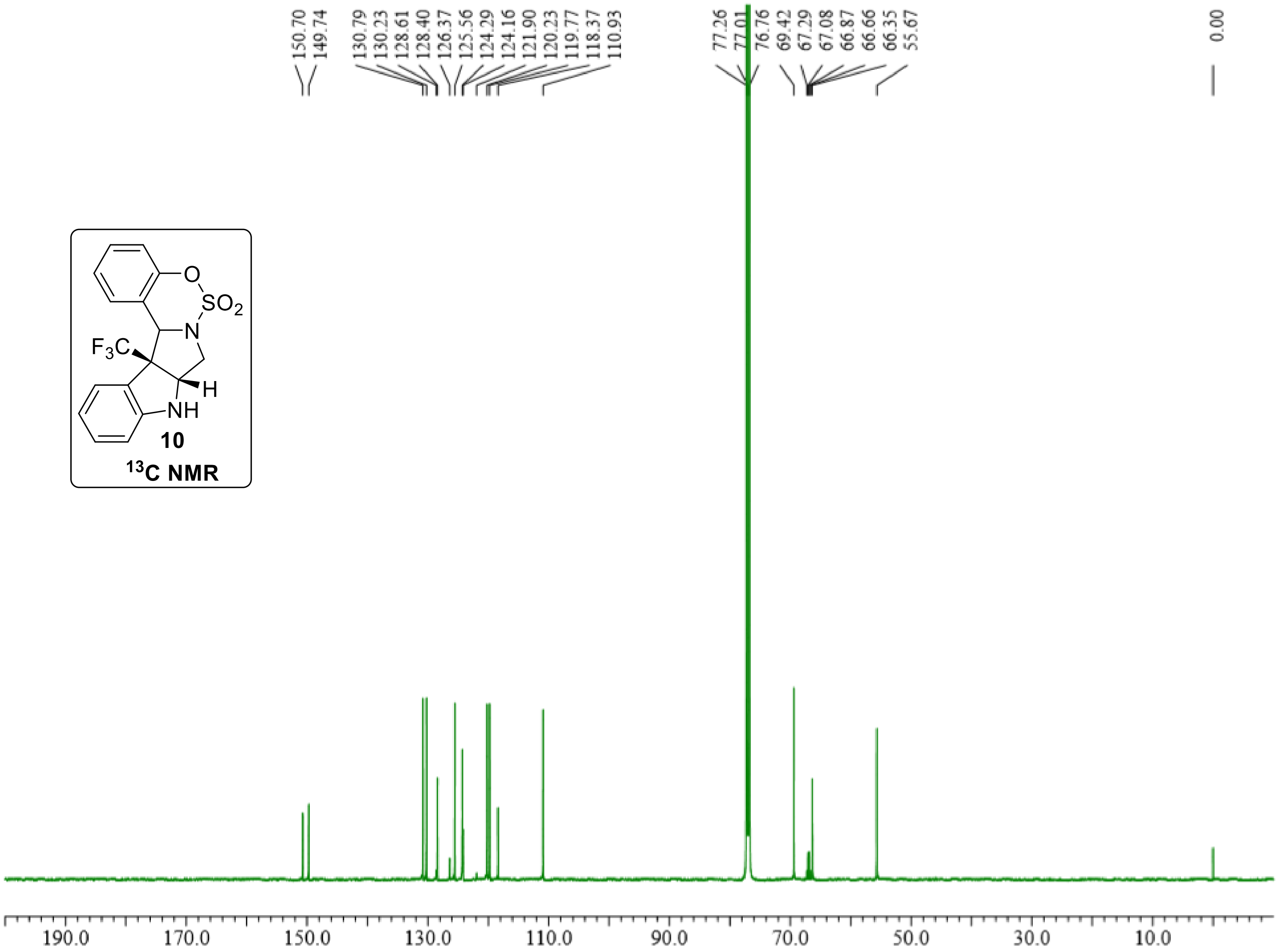

Supplementary Figure 122. ^{13}C NMR of **10**.

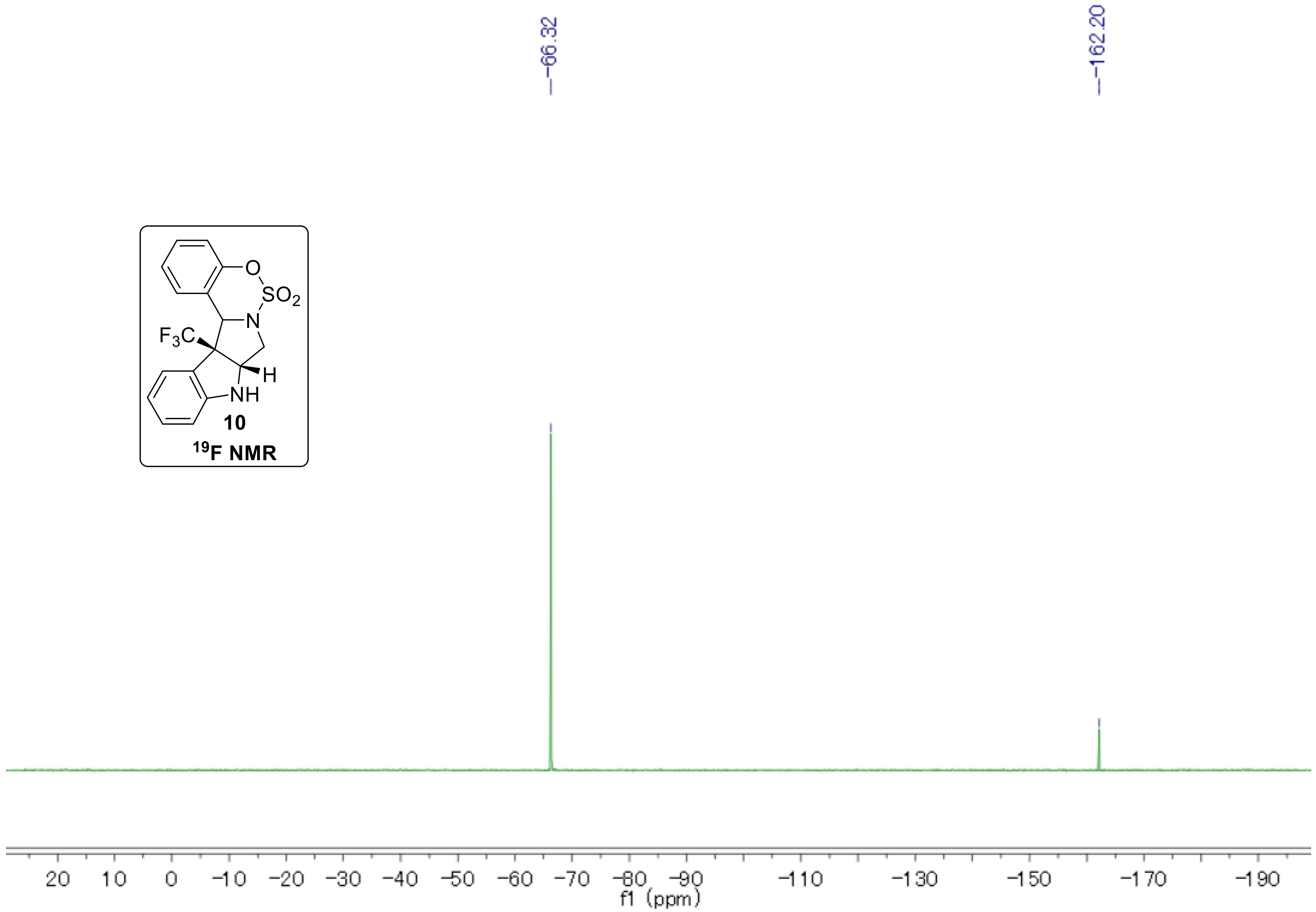

Supplementary Figure 123. ^{19}F NMR of **10**.

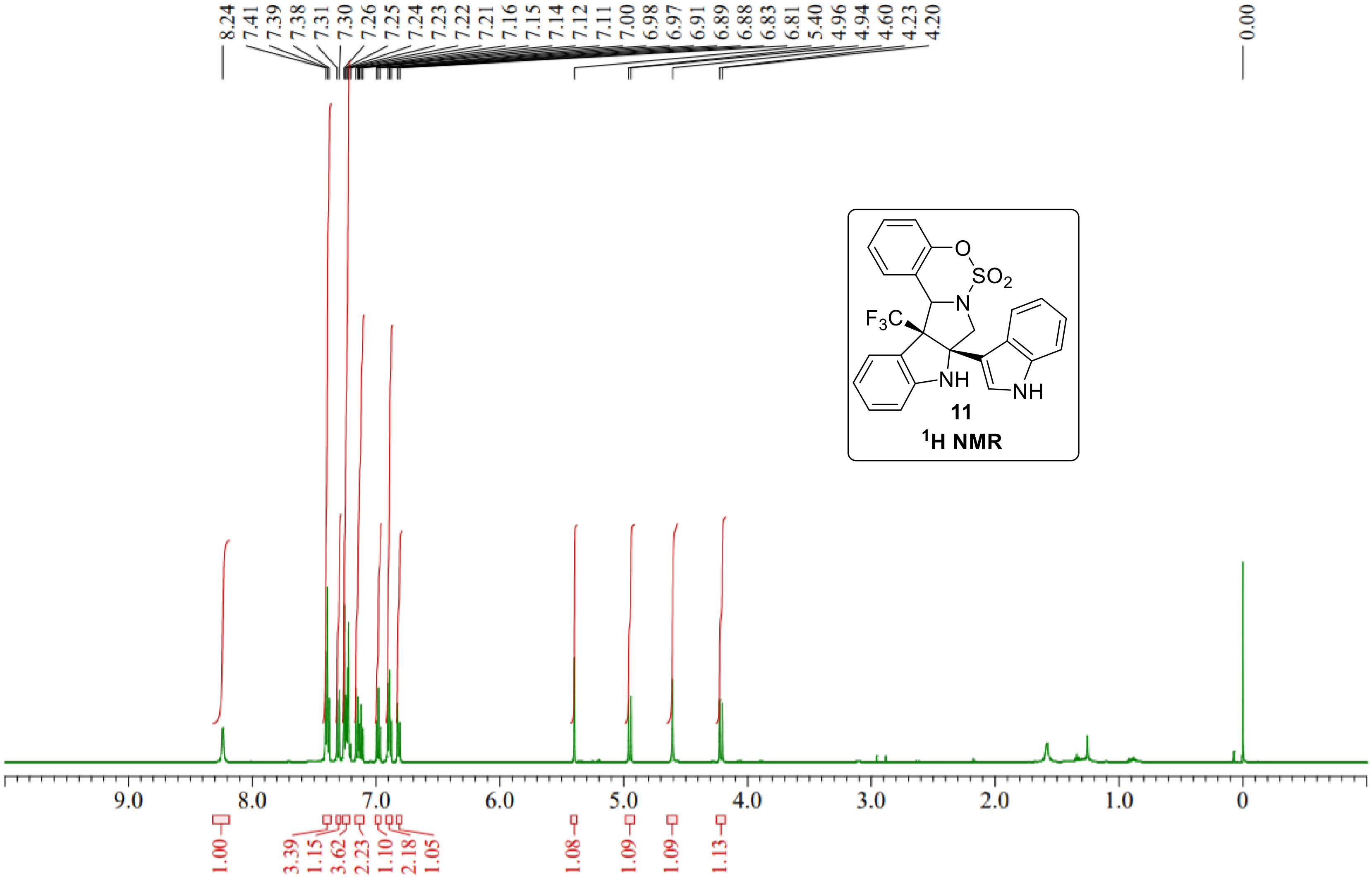

Supplementary Figure 124. ^{1}H NMR of **11**.

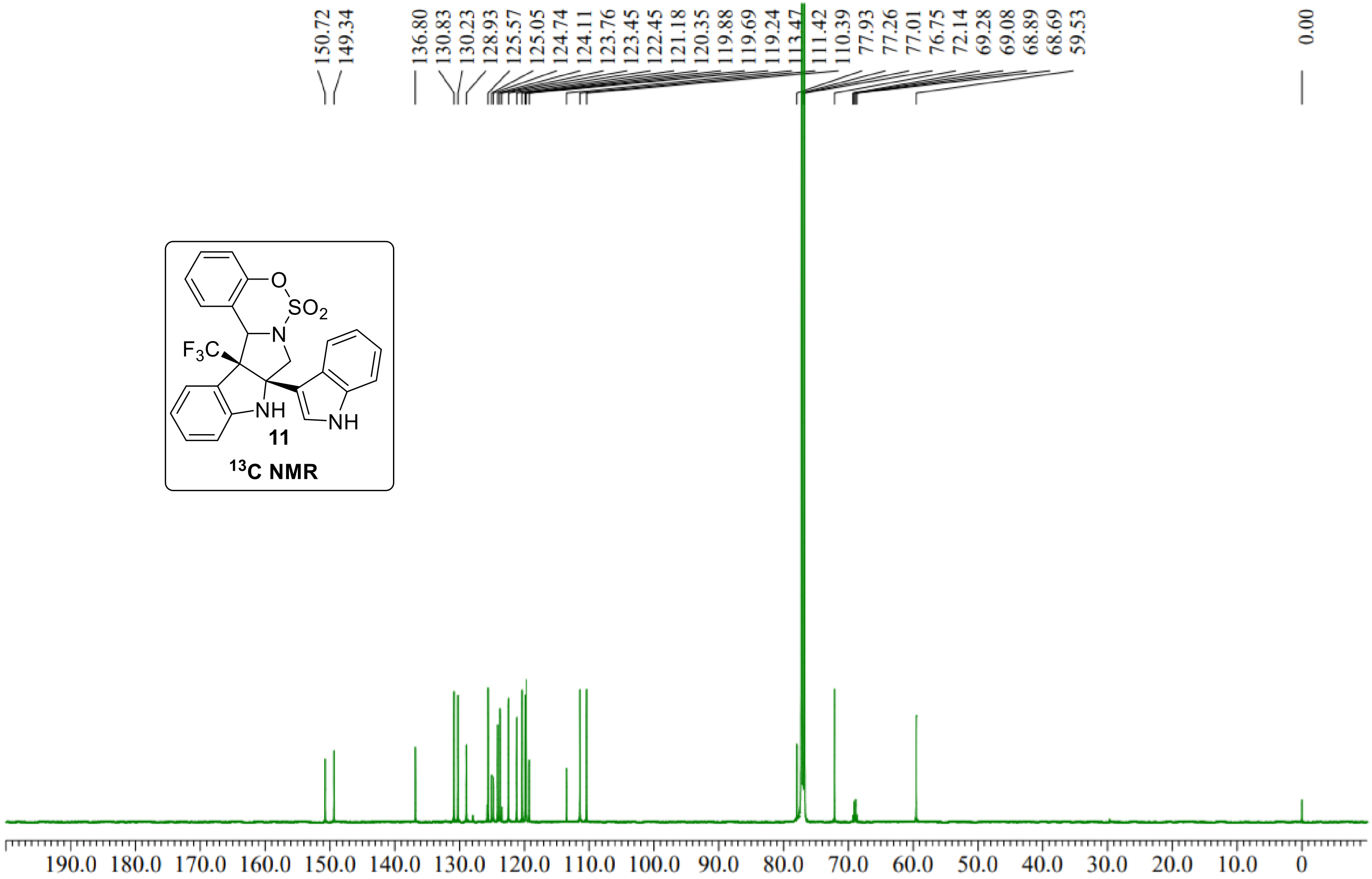

Supplementary Figure 125. ^{13}C NMR of **11**.

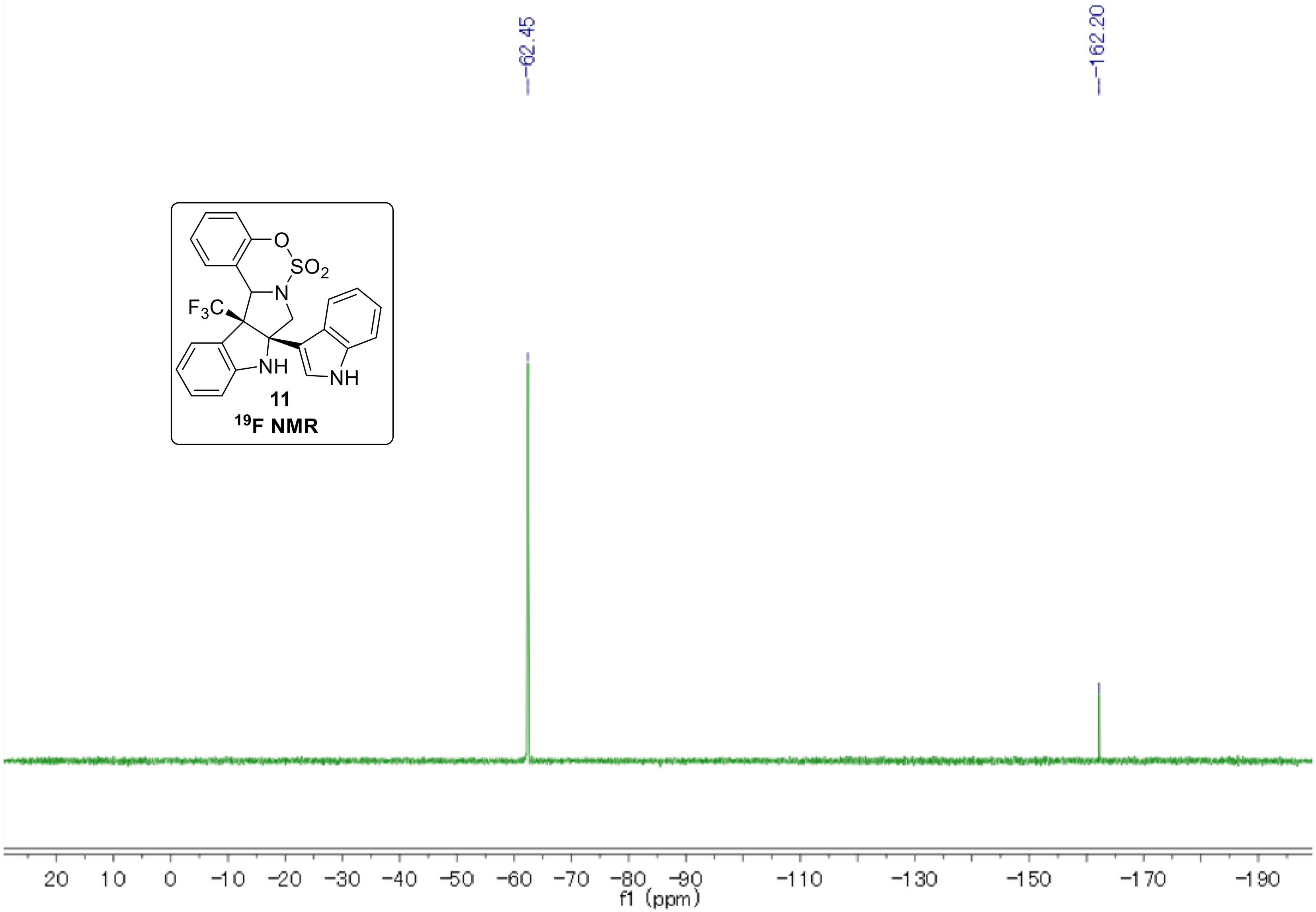

Supplementary Figure 126. ^{19}F NMR of **11**.

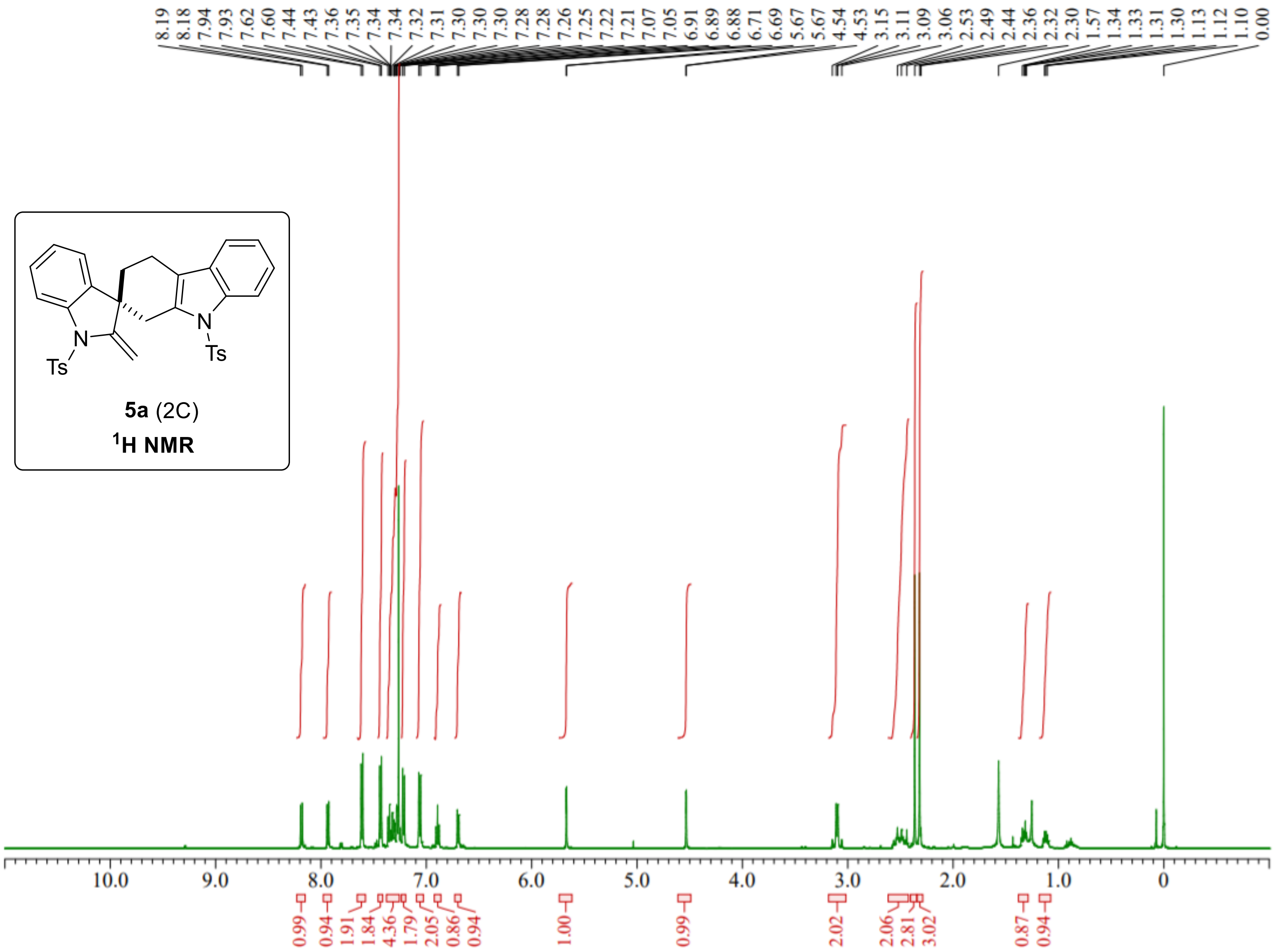

Supplementary Figure 127. ^{1}H NMR of **5a** (2C).

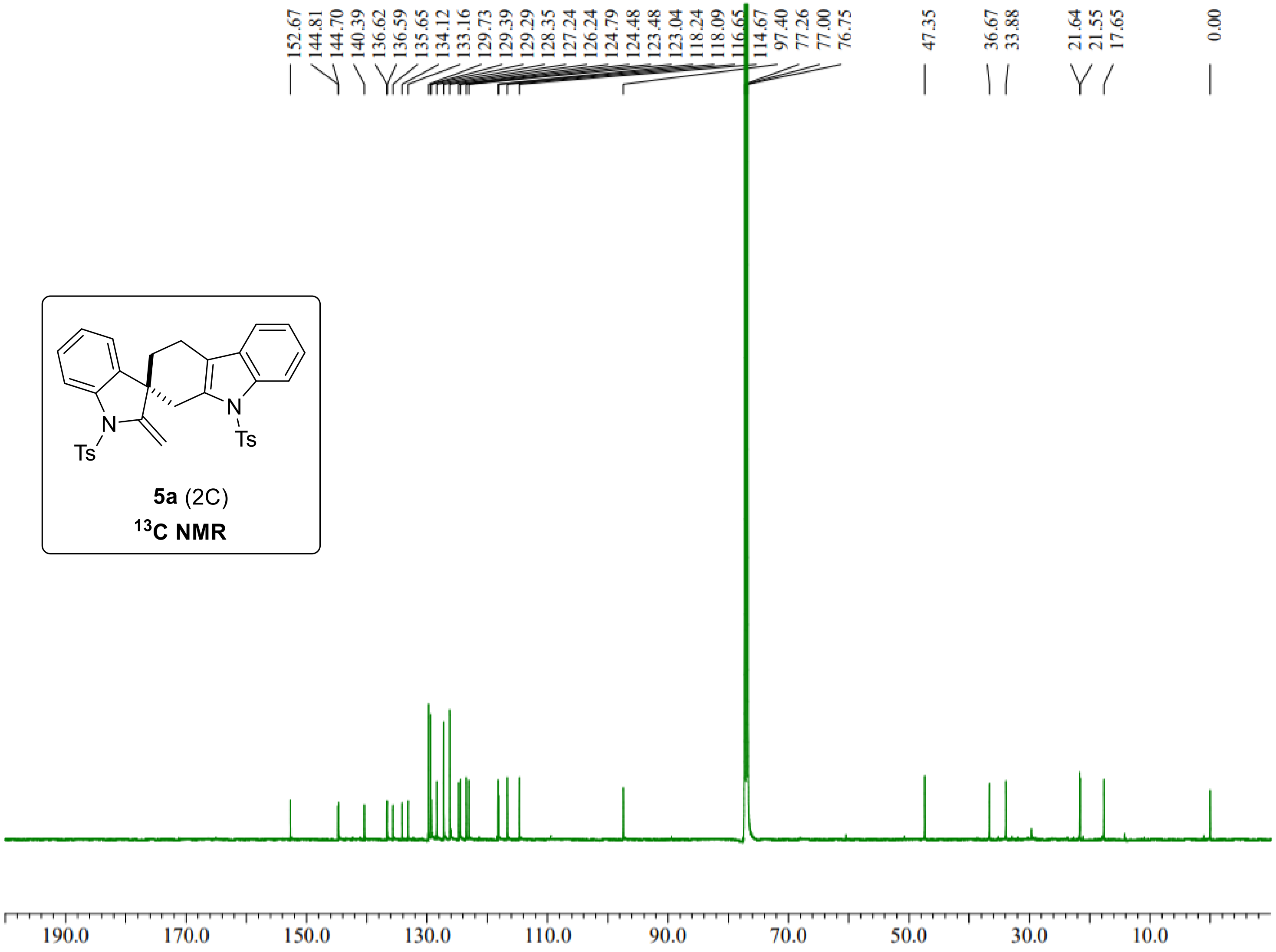

Supplementary Figure 128. ^{13}C NMR of **5a** (2C).

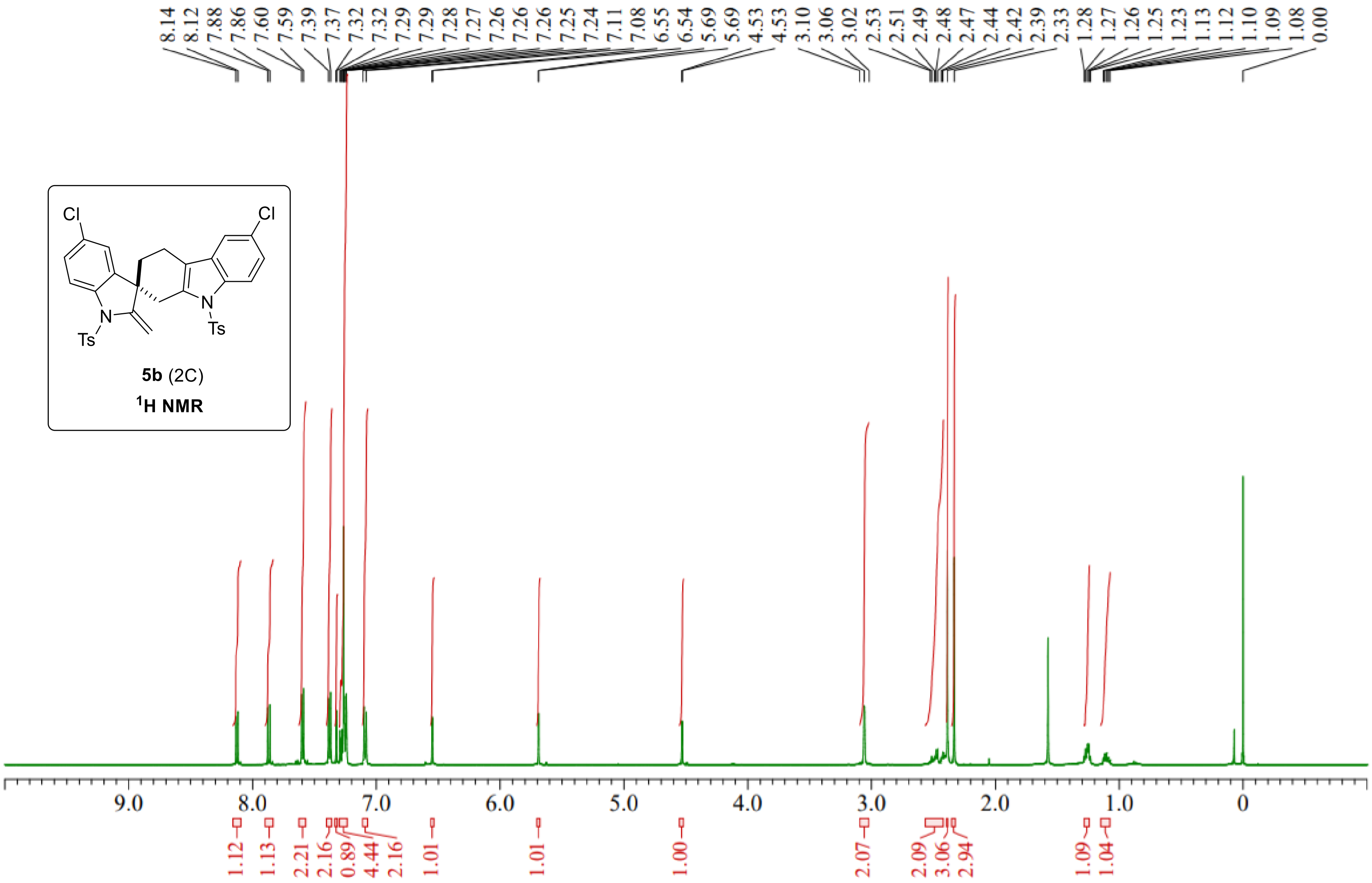

Supplementary Figure 129. ^{1}H NMR of **5b** (2C).

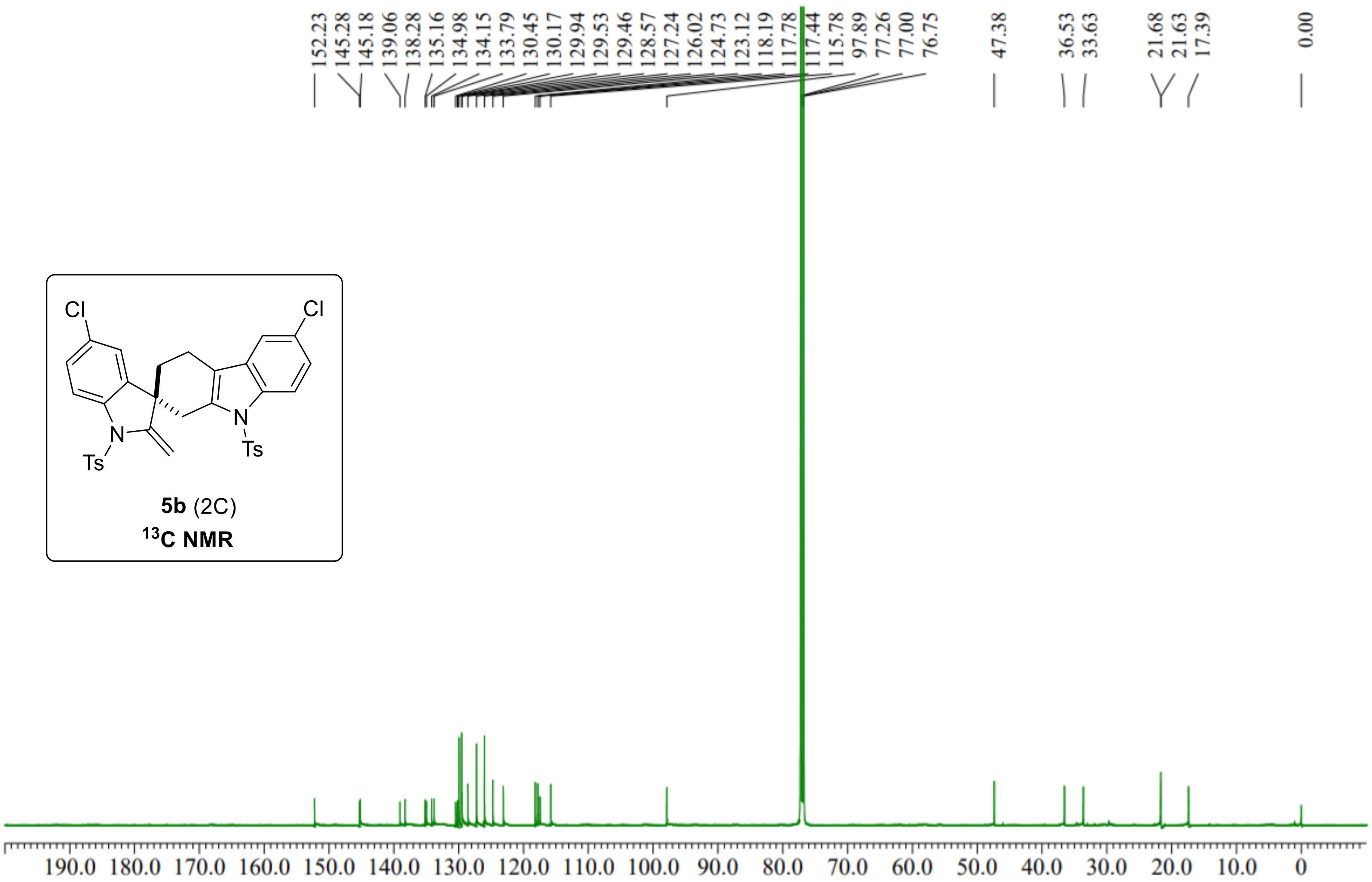

Supplementary Figure 130. ^{13}C NMR of **5b** (2C).

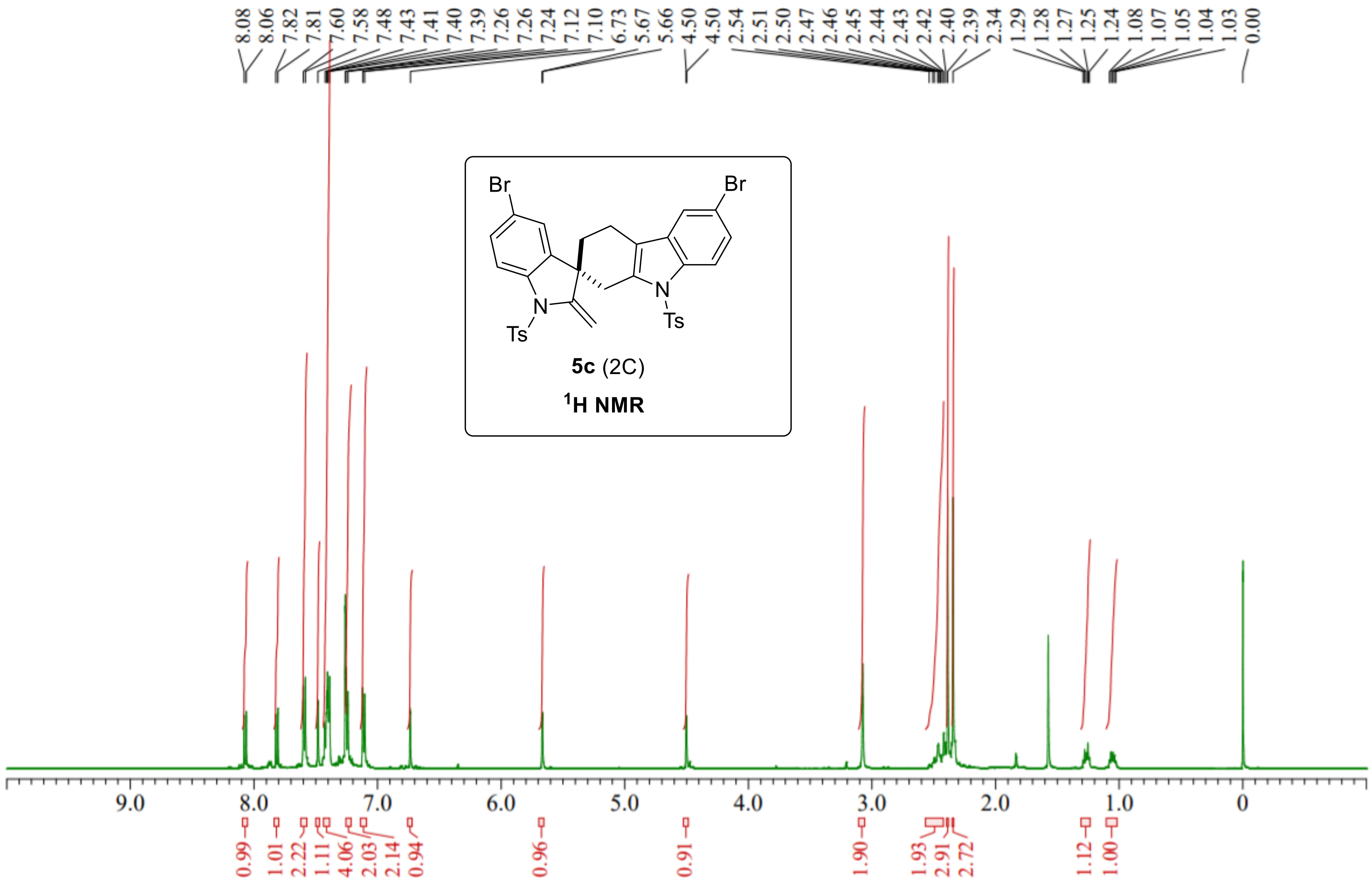

Supplementary Figure 131. ^{1}H NMR of **5c** (2C).

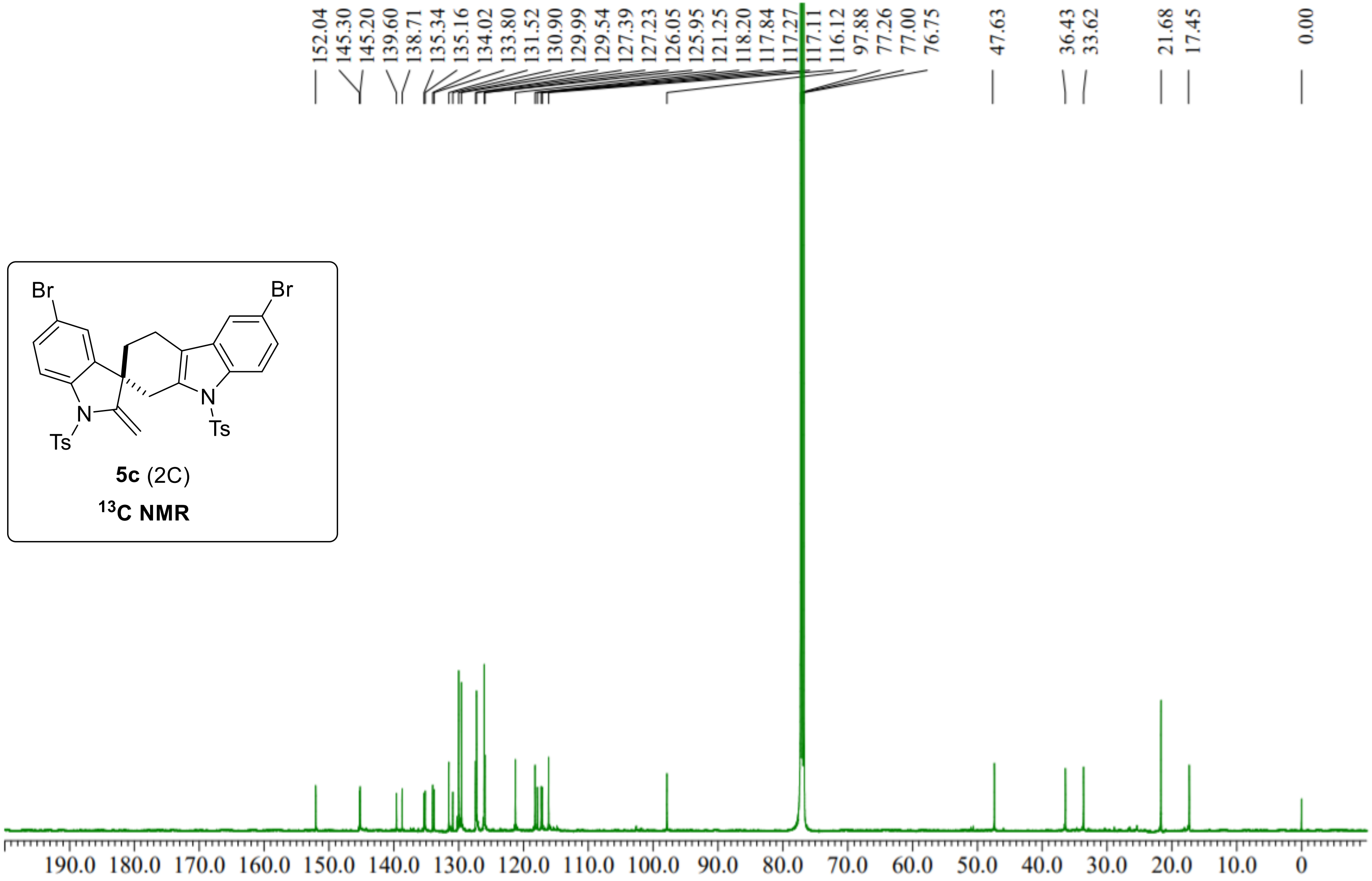

Supplementary Figure 132. ^{13}C NMR of **5c** (2C).

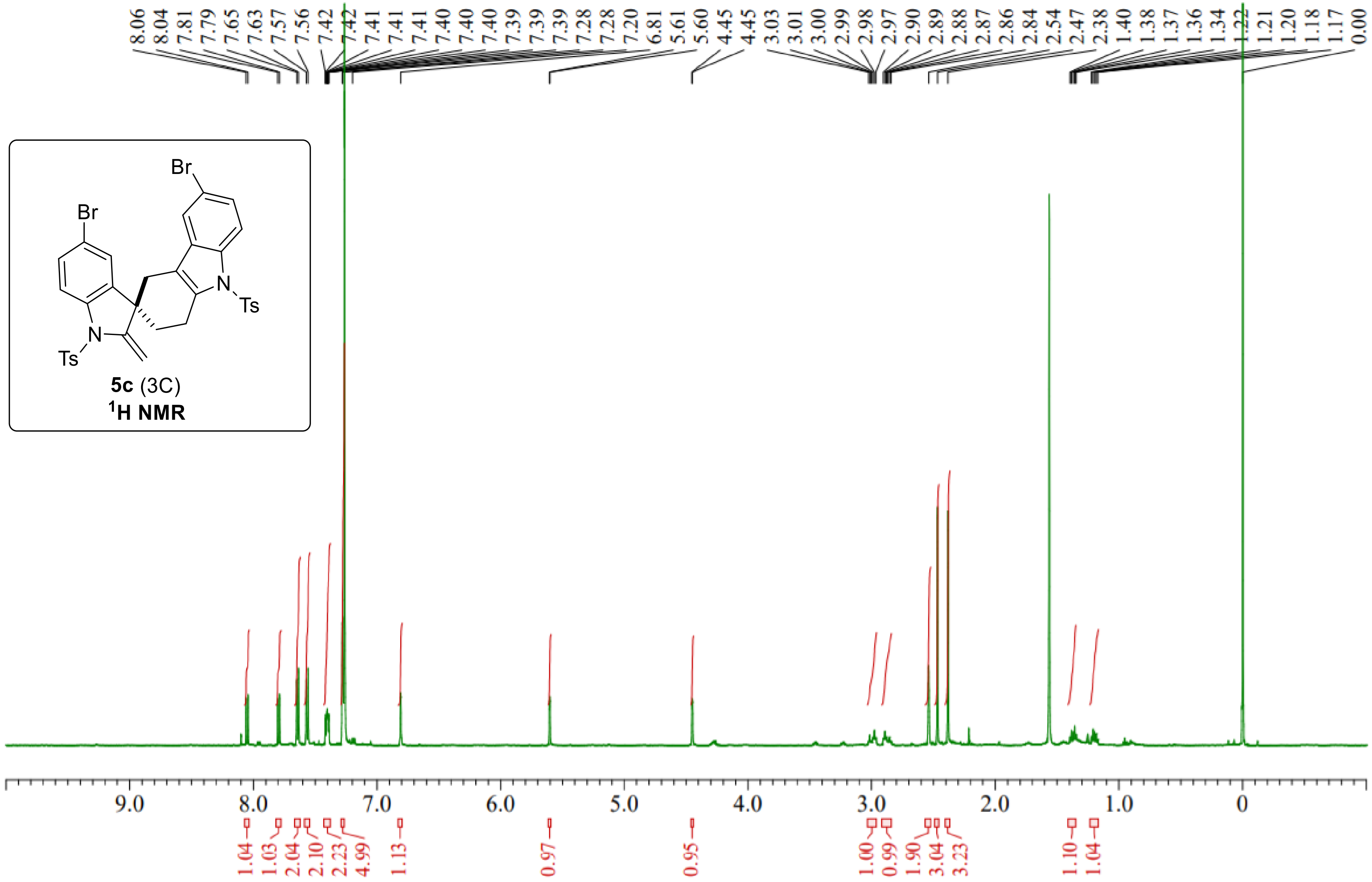

Supplementary Figure 133. ^{1}H NMR of **5c** (3C).

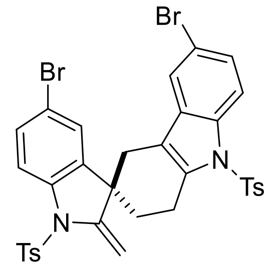

5c (3C)
^{13}C NMR

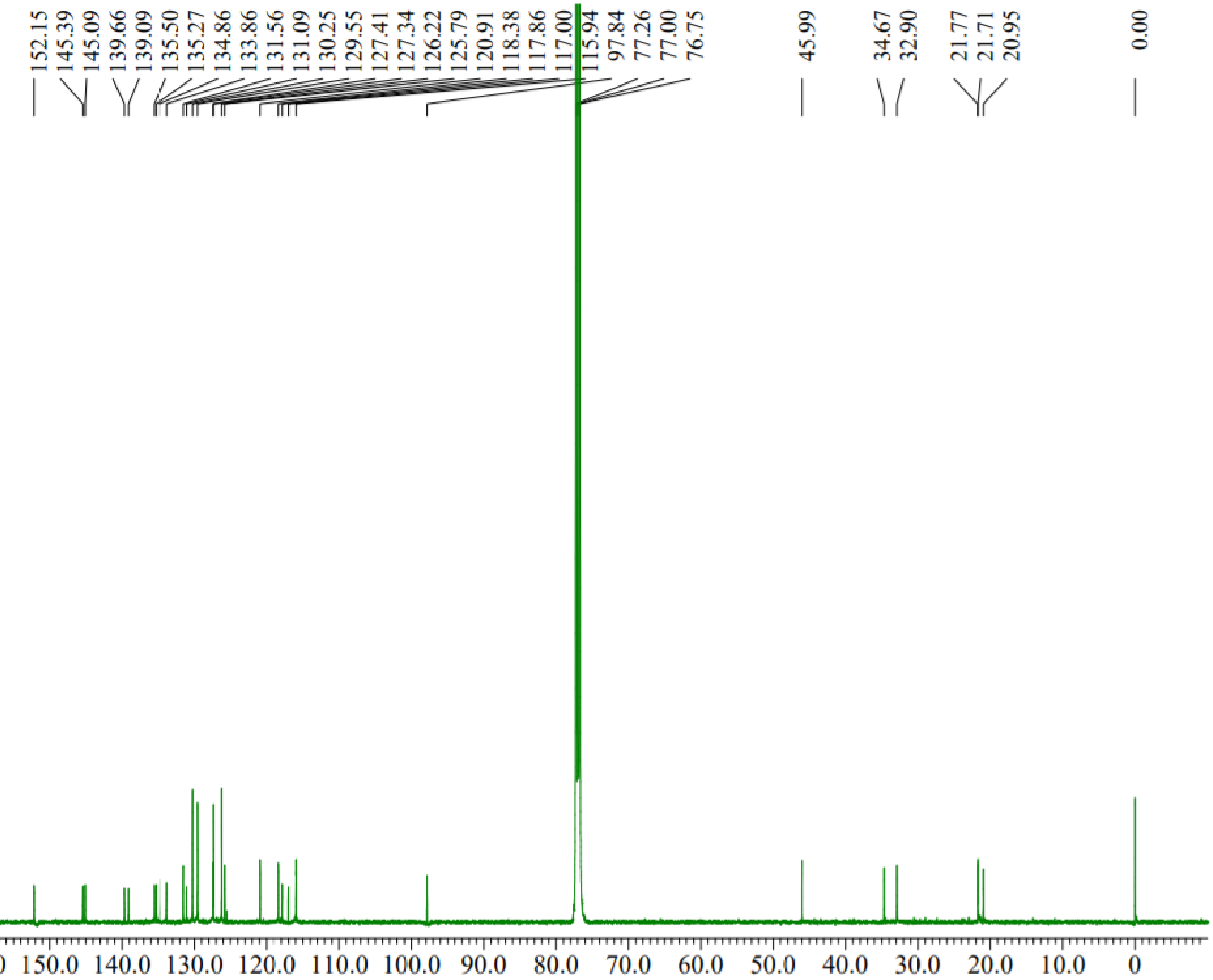

Supplementary Figure 134. ^{13}C NMR of **5c** (3C).

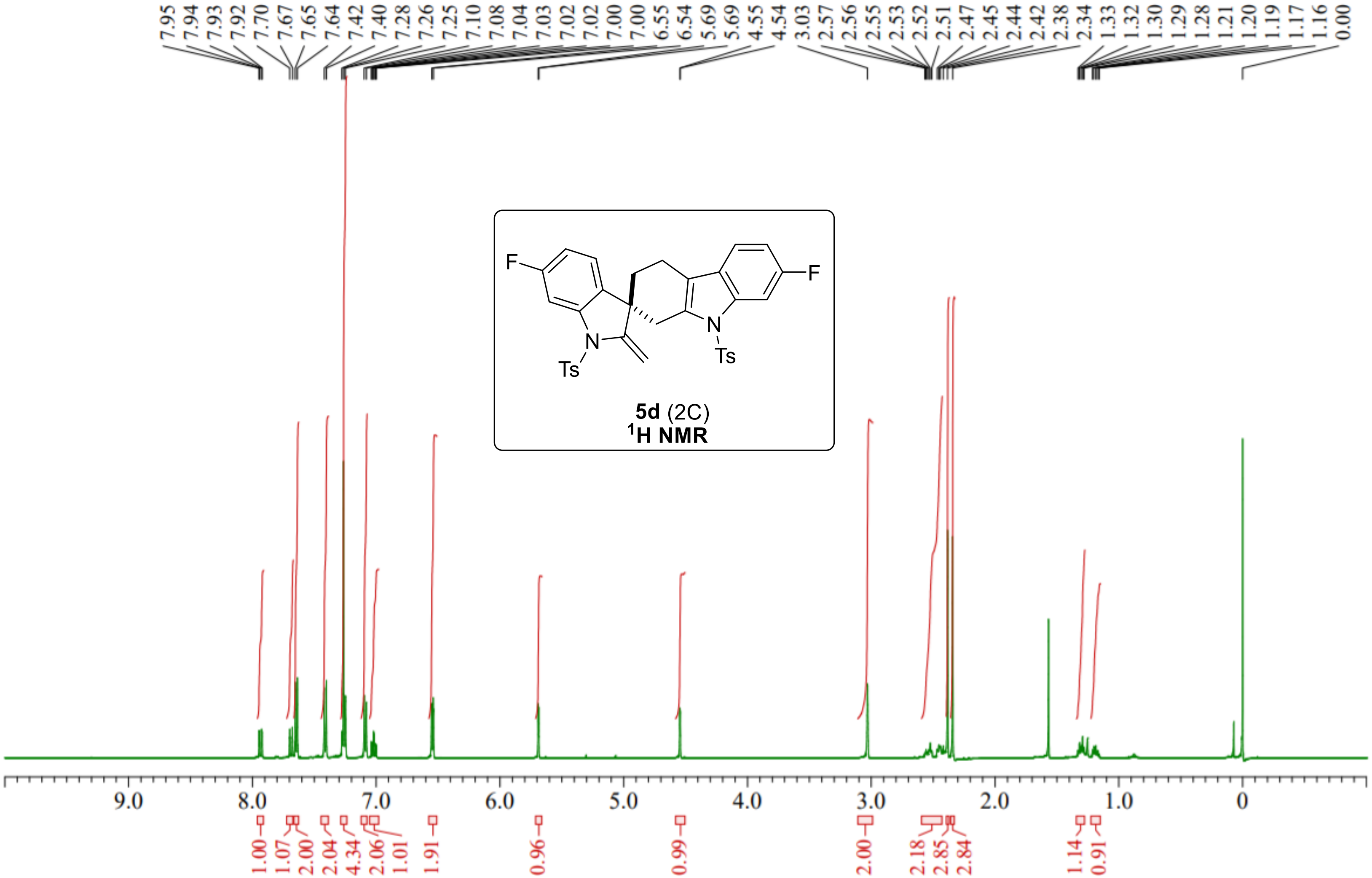

Supplementary Figure 135. ^{1}H NMR of **5d** (2C).

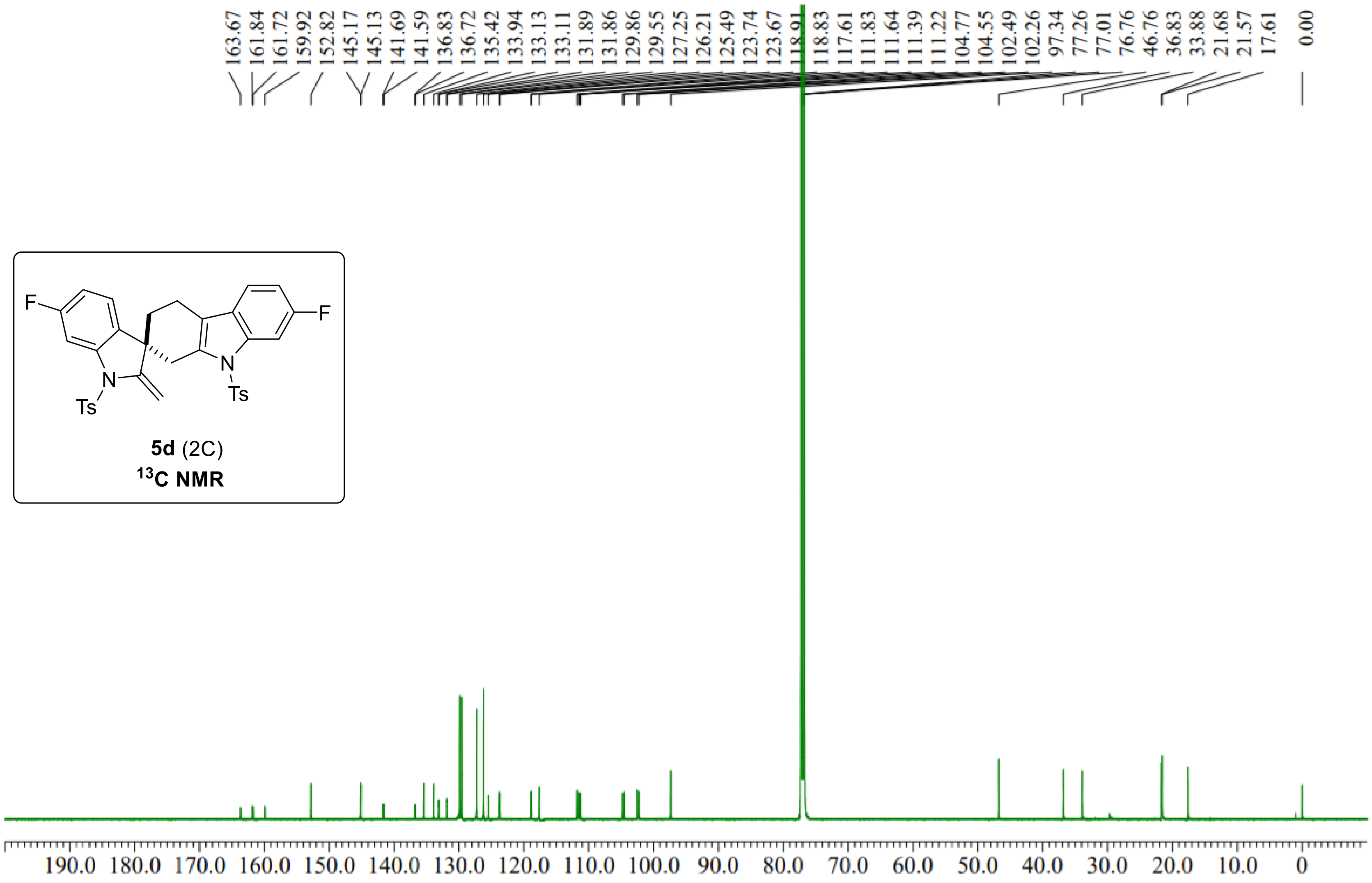

Supplementary Figure 136. ^{13}C NMR of **5d** (2C).

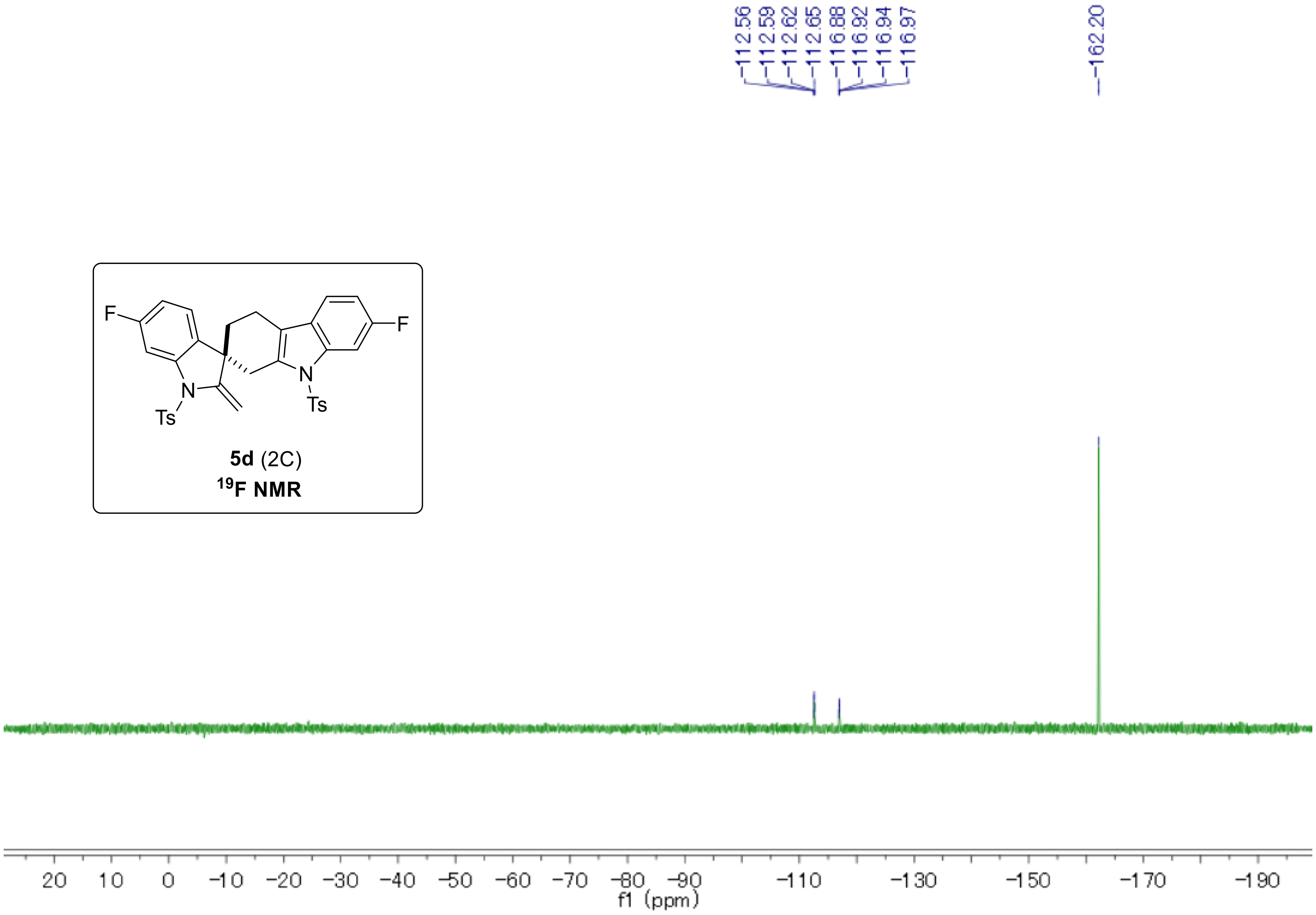

Supplementary Figure 137. ^{19}F NMR of **5d** (2C).

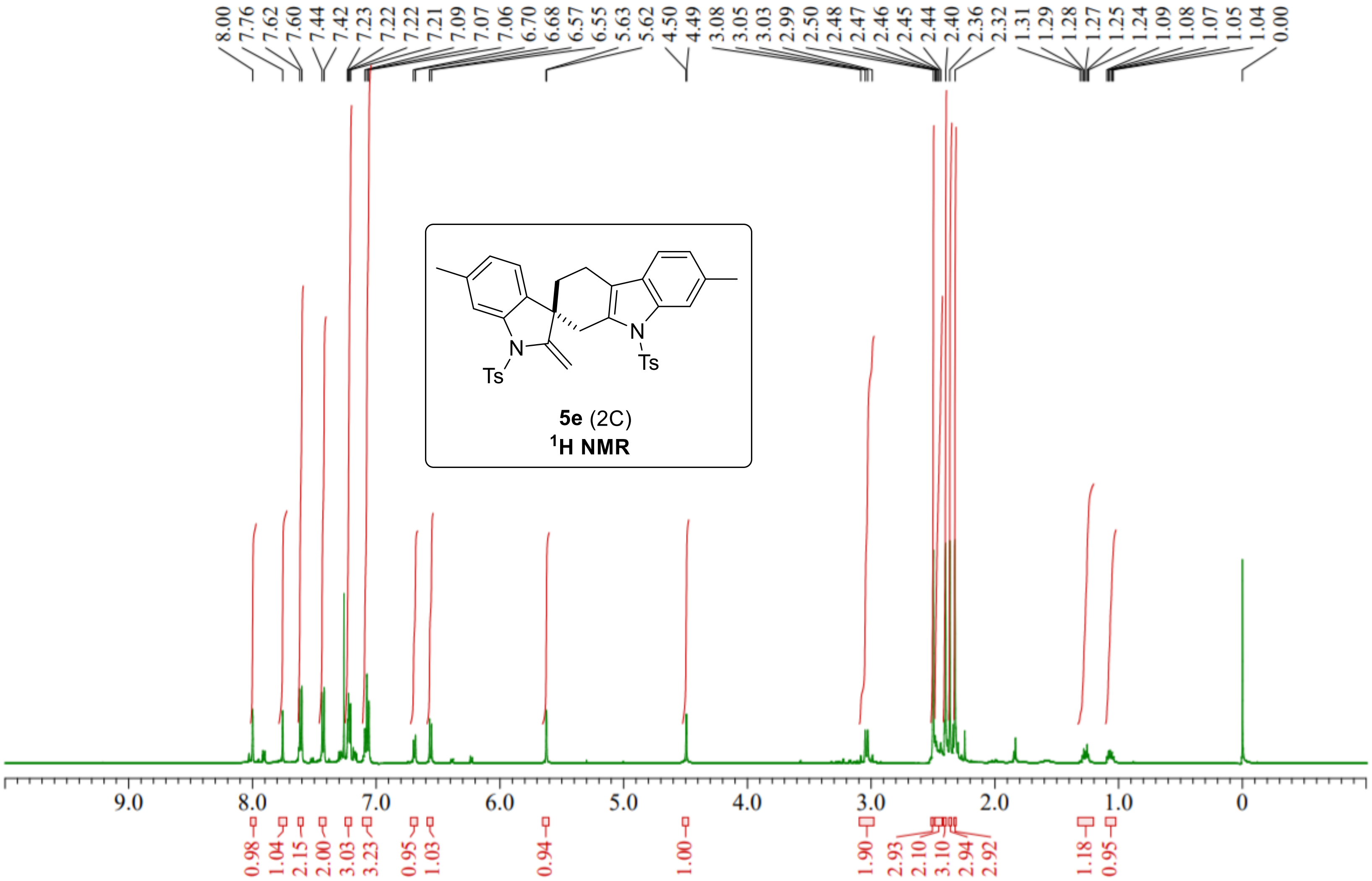

Supplementary Figure 138. ^{1}H NMR of **5e** (2C).

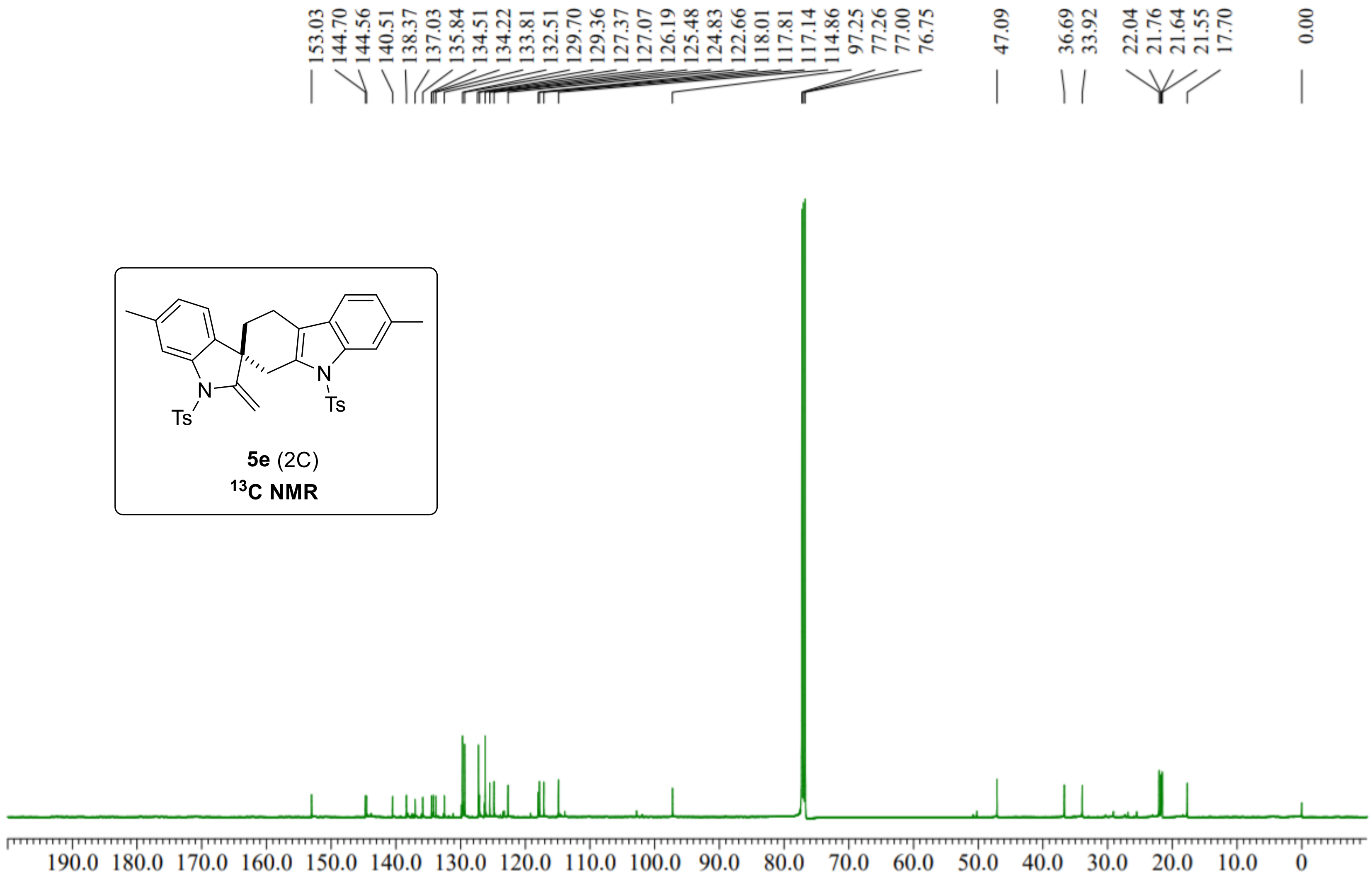

Supplementary Figure 139. ^{13}C NMR of **5e** (2C).

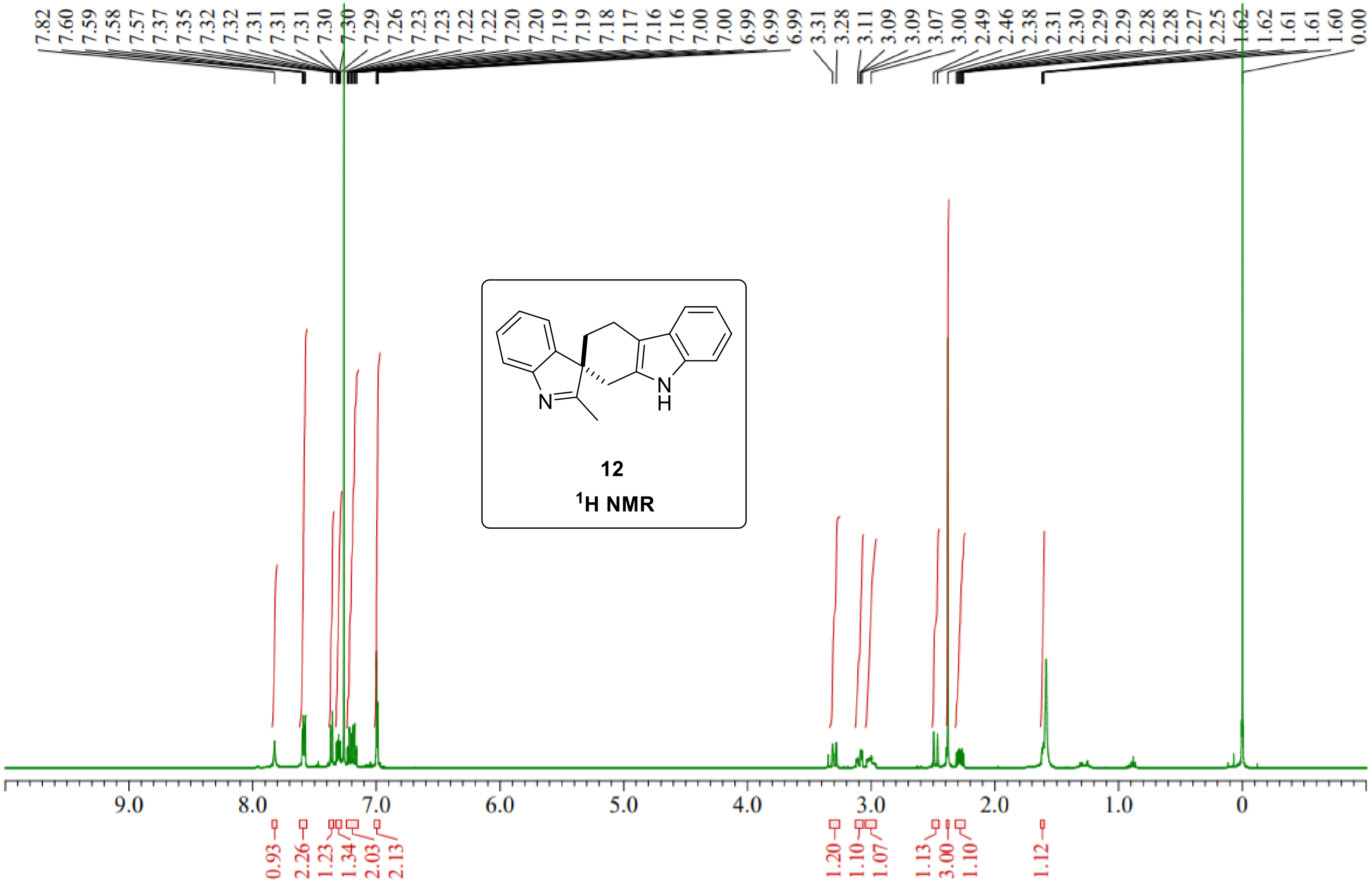

Supplementary Figure 140. ^{1}H NMR of **12**.

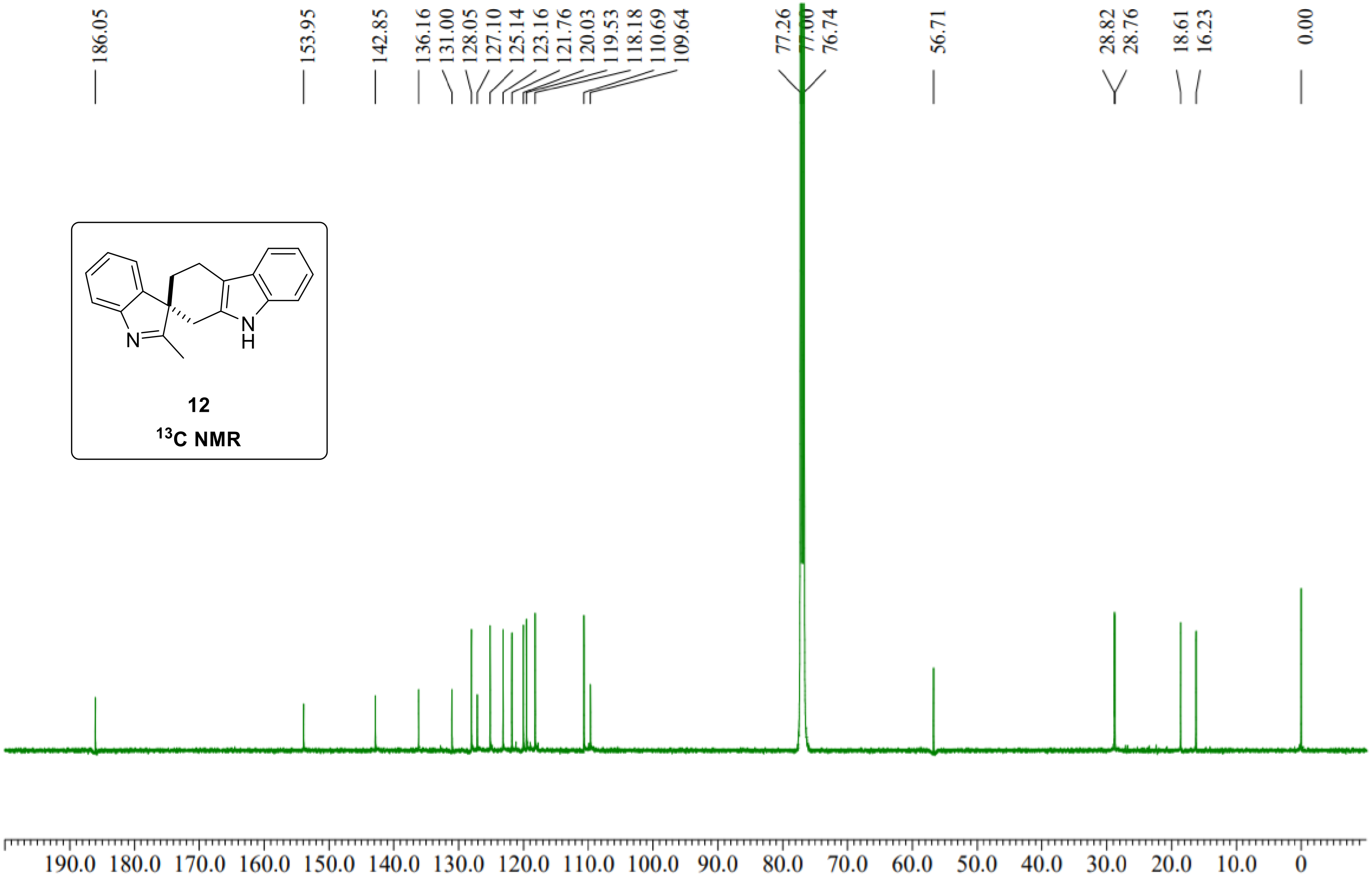

Supplementary Figure 141. ^{13}C NMR of **12**.

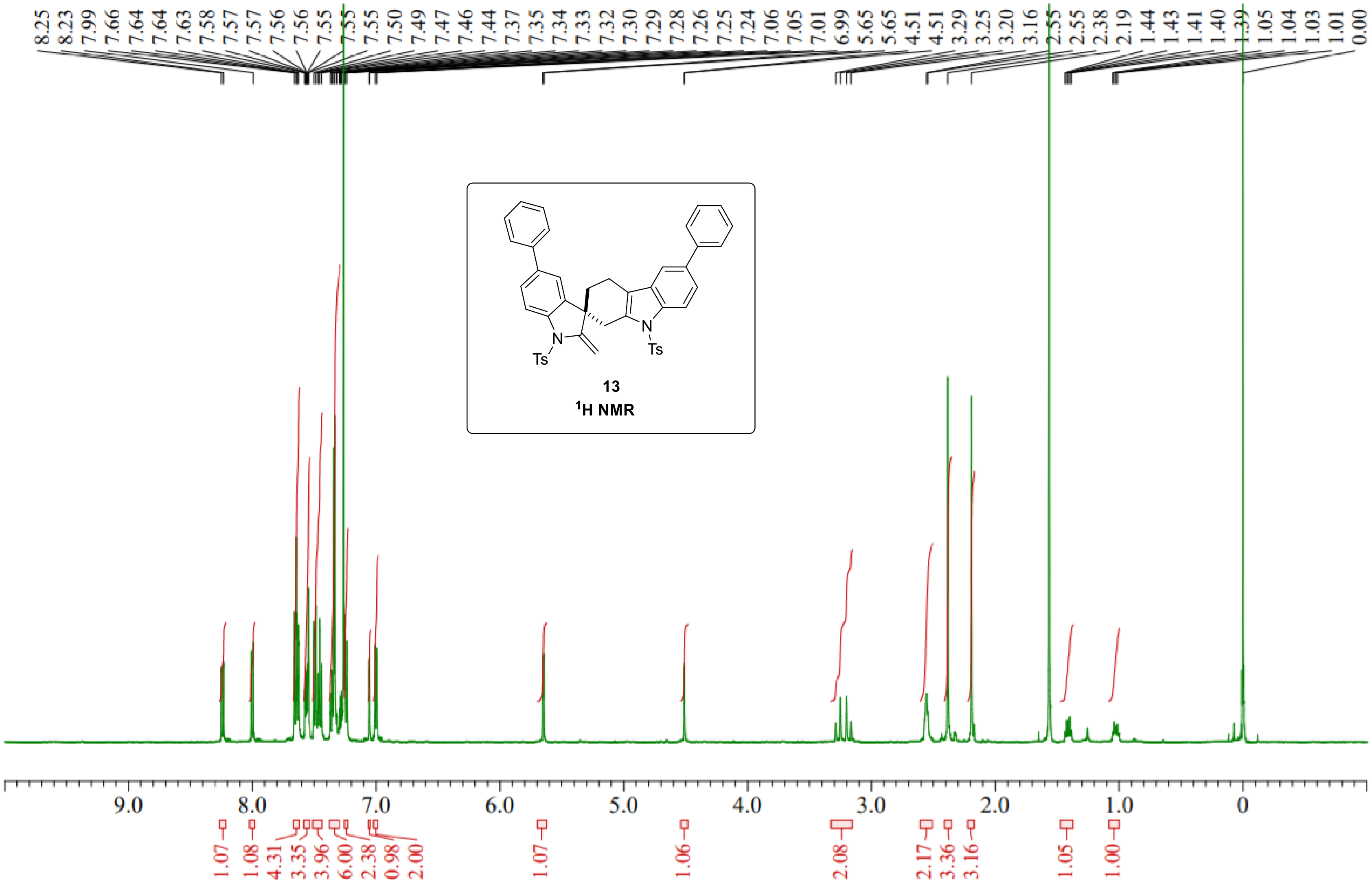

Supplementary Figure 142. ^{1}H NMR of **13**.

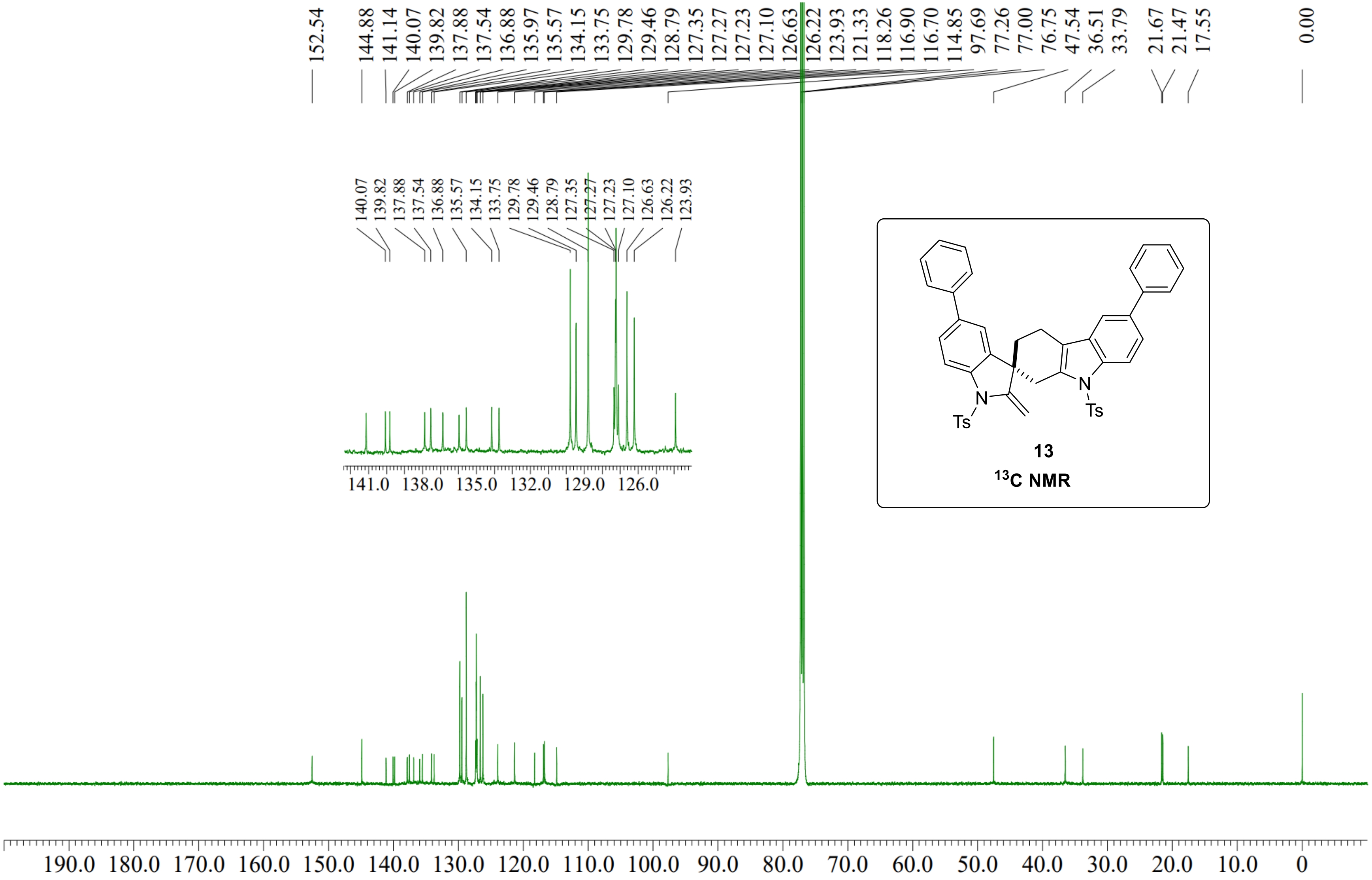

Supplementary Figure 143. ^{13}C NMR of **13**.

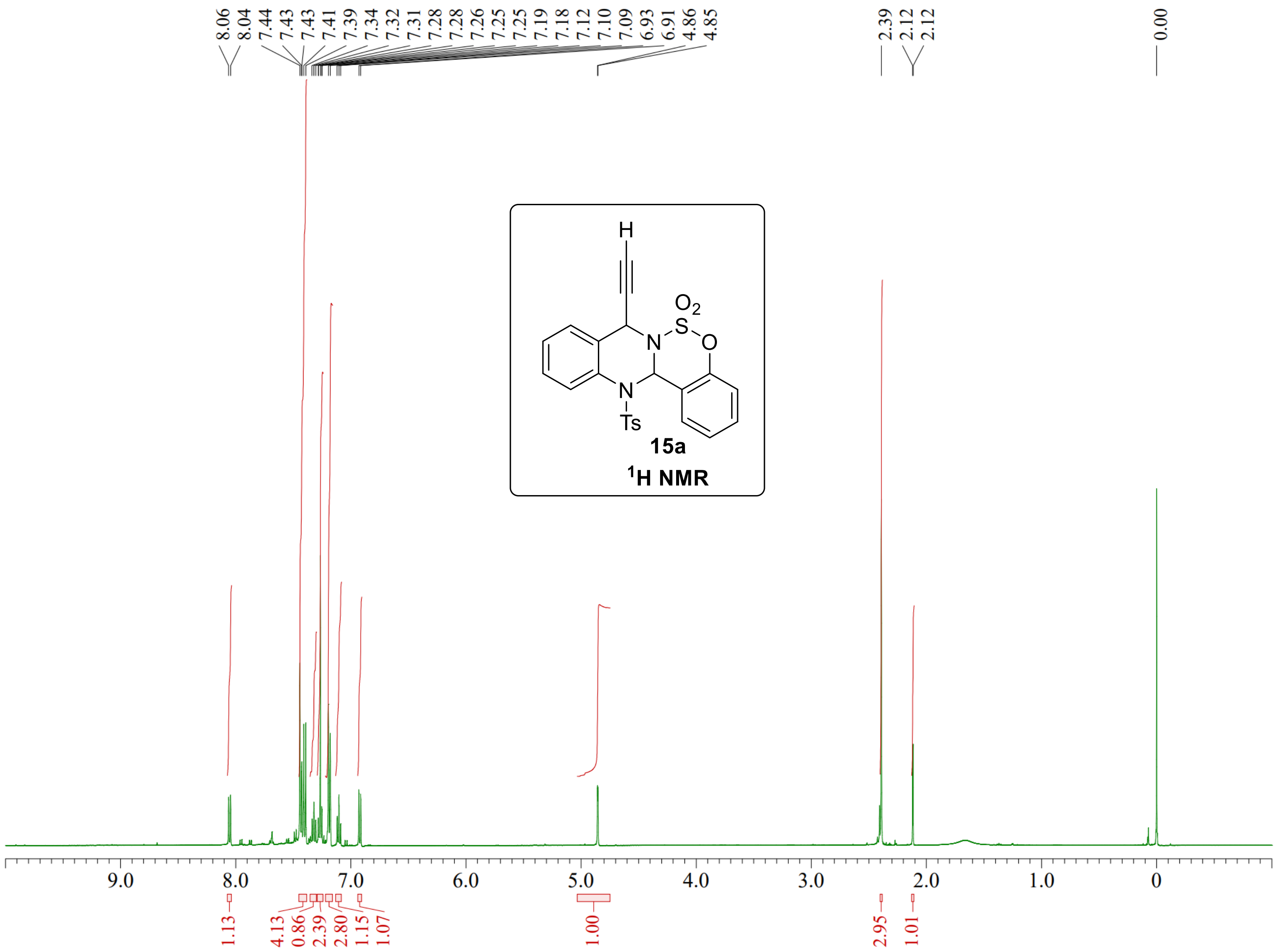

Supplementary Figure 144. ^{1}H NMR of **15a**.

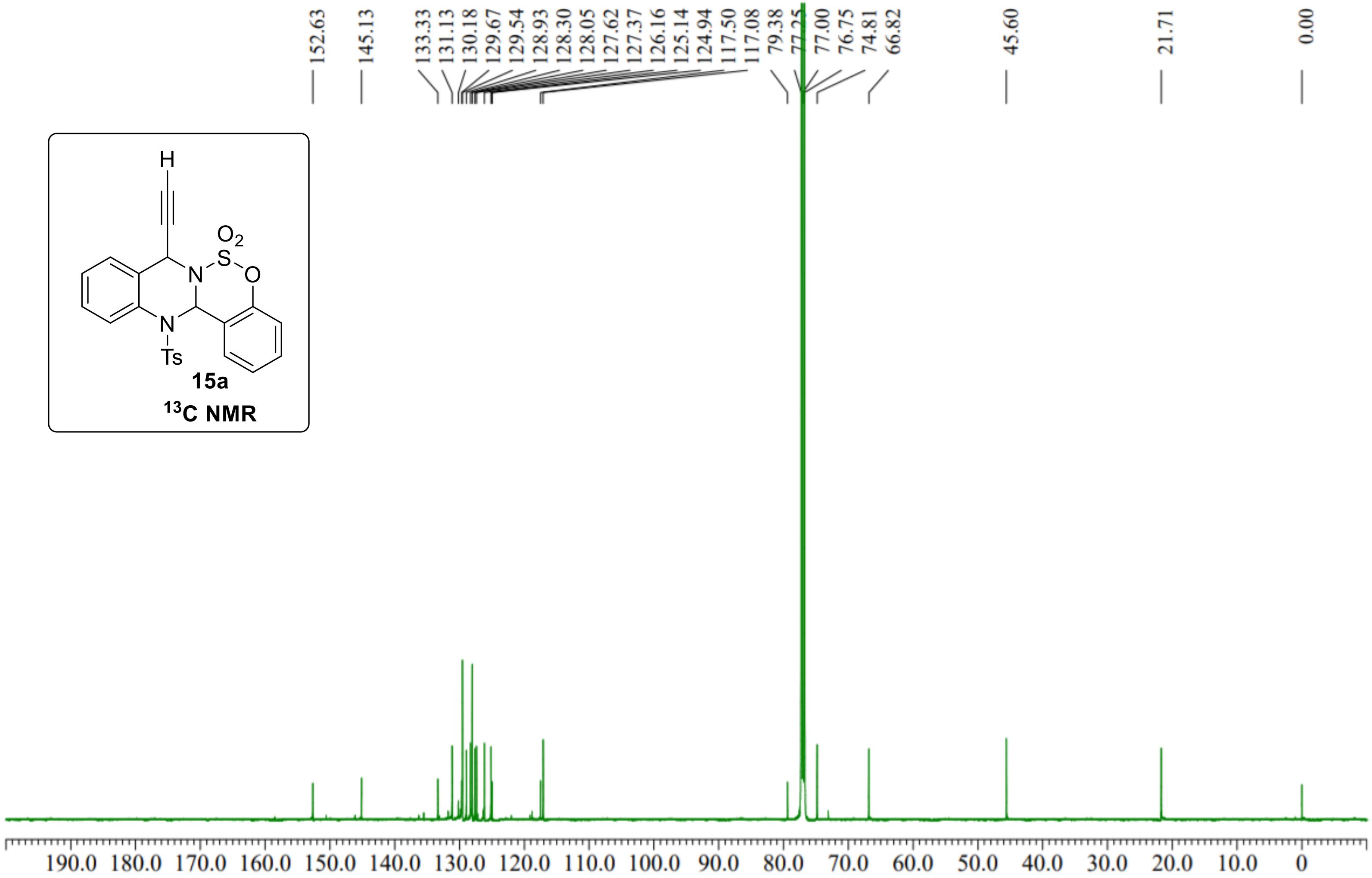

Supplementary Figure 145. ^{13}C NMR of **15a**.

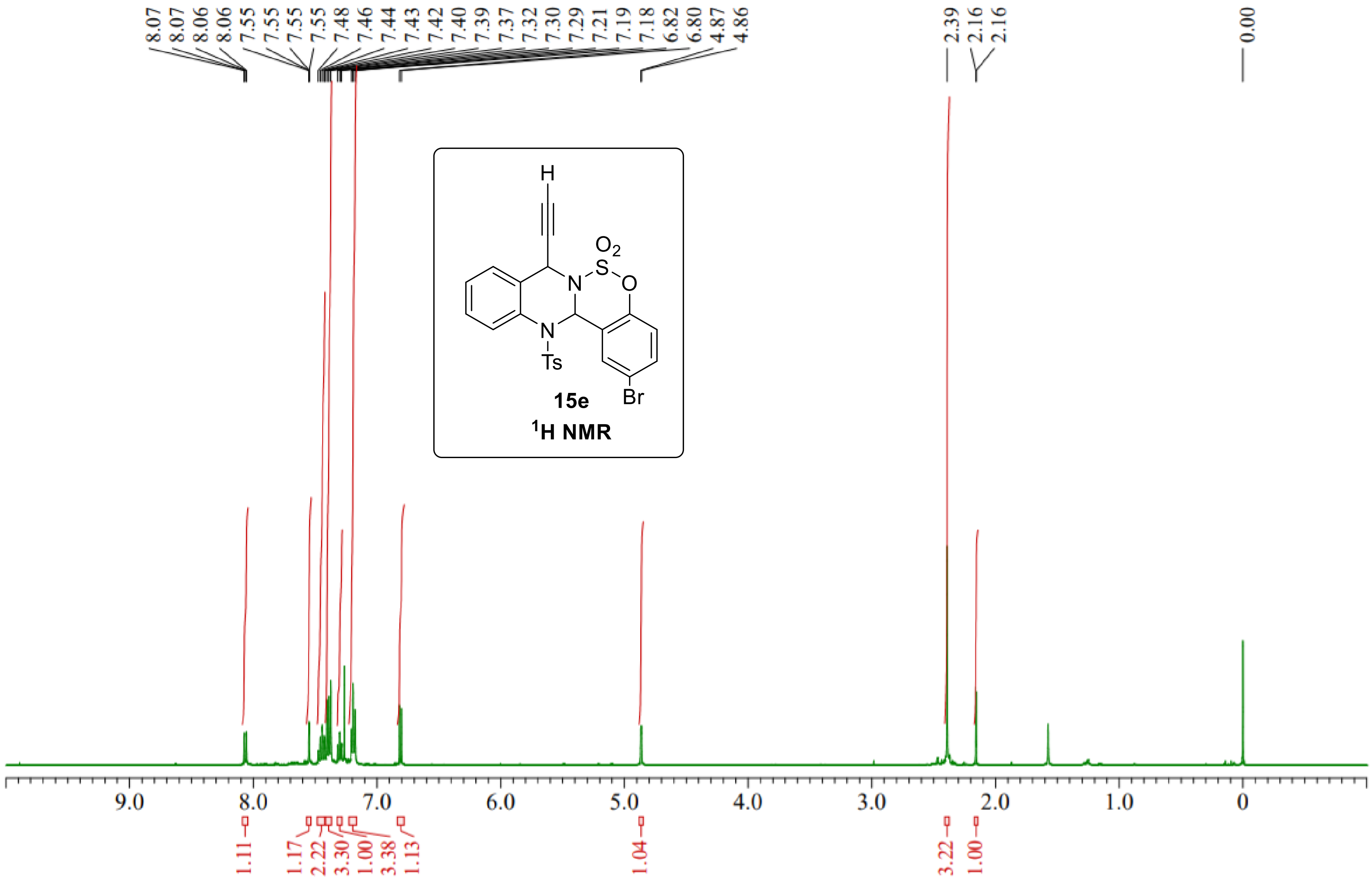

Supplementary Figure 146. ^{1}H NMR of **15e**.

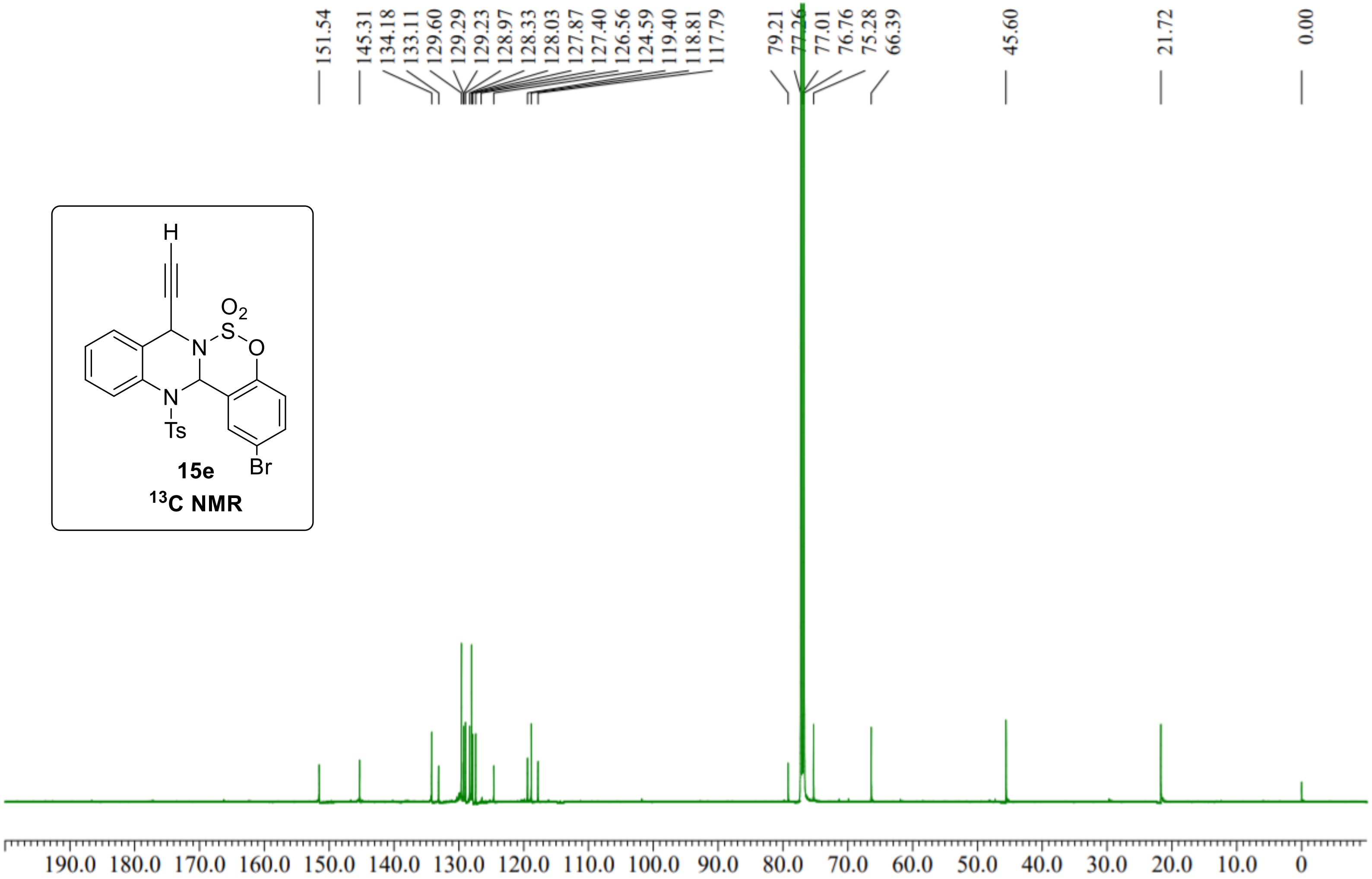

Supplementary Figure 147. ^{1}H NMR of **15e**.

13. Supplementary References

1. Gannarapu, M. R., Zhou, J., Jiang, B. & Shibata, N. Two catalytic annulation modes via Cu-allenylidenes with sulfur ylides that are dominated by the presence or absence of trifluoromethyl substituents. *iScience* **23**, 100994 (2020)
2. Wang, Q., Li, T. R., Lu, L. Q., Li, M. M., Zhang, K. & Xiao, W. J. Catalytic Asymmetric [4+1] Annulation of Sulfur Ylides with Copper–Allenylidene Intermediates. *J. Am. Chem. Soc.* **138**, 8360–8363 (2016).
3. Nouch, R., Cini, M., Magre, M., Abid, M., Dieguez, M., Pamies, M., Woodward, S. & Lewis, W. Enantioselective Synthesis of 6,6‐Disubstituted Pentafulvenes Containing a Chiral Pendant Hydroxy Group. *Chem. Eur. J.* **23**, 17195−17198 (2017).
4. Jia, C. M., Zhang, H. X., Nie, J. & Ma, J.A. Catalytic Asymmetric Decarboxylative Mannich Reaction of Malonic Acid Half Esters with Cyclic Aldimines: Access to Chiral β-Amino Esters and Chroman-4-amines. *J. Org. Chem.* **81**, 8561−8569 (2016).
5. Shi, A., Wang, D., Wang, H., Wu, Y., Tian, H., Guan, Q., Bao, K. & Zhang, W. Synthesis and bioevaluation of 2-phenyl-5-methyl-2H-1,2,3-triazole-4-carboxylic acid/ carbohydrazide derivatives as potent xanthine oxidase inhibitors. *RSC Adv.* **6**, 114879 (2016).
6. Yin, Q. & You, S.-L. Chiral phosphoric acid-catalysed Friedel–Crafts alkylation reaction of indoles with racemic spiro indolin-3-ones. *Chem. Sci.* **2**, 1344−1348 (2011).
7. Frisch, M. J., Trucks, G. W., Schlegel, H. B., Scuseria, G. E., Robb, M. A., Cheeseman, J. R., Scalmani, G., Barone, V., Petersson, G. A., Nakatsuji, H., Li, X., Caricato, M., Marenich, A. V., Bloino, J., Janesko, B. G., Gomperts, R., Mennucci, B., Hratchian, H. P., Ortiz, J. V., Izmaylov, A. F., Sonnenberg, J. L., Williams-Young, D., Ding, F., Lipparini, F., Egidi, F., Goings, J., Peng, B., Petrone, A., Henderson, T., Ranasinghe, D., Zakrzewski, V. G., Gao, J., Rega, N., Zheng, G., Liang, W., Hada, M., Ehara, M., Toyota, K., Fukuda, R., Hasegawa, J., Ishida, M., Nakajima, T., Honda, Y., Kitao, O., Nakai, H., Vreven, T., Throssell, K., Montgomery, J. A., Jr., Peralta, J. E., Ogliaro, F., Bearpark, M. J., Heyd, J. J., Brothers, E. N., Kudin, K. N., Staroverov, V. N., Keith, T. A., Kobayashi, R., Normand, J., Raghavachari, K., Rendell, A. P., Burant, J. C., Iyengar, S. S., Tomasi, J., Cossi, M., Millam, J. M., Klene, M., Adamo, C., Cammi, R., Ochterski, J. W., Martin, R. L., Morokuma, K., Farkas, O., Foresman, J. B. & Fox, D. J. *Gaussian 16, Revision C.01*, Gaussian, Inc., Wallingford CT, (2016).
8. Becke, A. D. Density functional thermochemistry. III. The role of exact exchange. *J. Chem. Phys.* **98***,* 5648−5652 (1993).

9. Grimme, S., Antony, J., Ehrlich, S. & Krieg, H. A consistent and accurate *ab initio* parametrization of density functional dispersion correction (DFT-D) for the 94 elements H-Pu. *J. Chem. Phys*. **132**, 154104 (2010).
10. Singh, U. C. & Kollman, P. A. An Approach to Computing Electrostatic Charges for Molecules. *J. Comput. Chem.* **5**, 129−45 (1984).
11. Besler, B. H., Merz, K. M. Jr & Kollman, P. A. Atomic charges derived from semiempirical methods. *J. Comput. Chem.* **11**, 431–439 (1990).
