# Supplementary material for: Construction of poly-N-heterocyclic scaffolds via the controlled reactivity of Cu-allenylidene intermediates
Source: Commun Chem. 2021 Nov 18;4:158. doi: 10.1038/s42004-021-00596-x (PMC9814594; doi:10.1038/s42004-021-00596-x)
Supplement: Supplementary file 7 — Supplemental Data 5 [file 42004_2021_596_MOESM7_ESM.pdf]

Supplementary Data 5

I (with L1)

TITLE CF3_cis-2cu opt b3lyp/6-311G** + D3

REMARK 1 File created by GaussView 6.0.16

HETATM	1	C	0	-0.672	-1.875	3.666	C
HETATM	2	C	0	-1.677	-3.038	3.825	C
HETATM	3	H	0	-2.652	-2.743	4.208	H
HETATM	4	H	0	-1.277	-3.863	4.420	H
HETATM	5	C	0	-0.908	-2.949	1.722	C
HETATM	6	C	0	-0.821	-3.277	0.291	C
HETATM	7	C	0	-1.719	-4.145	-0.330	C
HETATM	8	H	0	-2.489	-4.633	0.249	H
HETATM	9	C	0	-1.606	-4.325	-1.705	C
HETATM	10	H	0	-2.296	-4.973	-2.230	H
HETATM	11	C	0	-0.603	-3.656	-2.396	C
HETATM	12	H	0	-0.478	-3.767	-3.464	H
HETATM	13	C	0	0.248	-2.810	-1.681	C
HETATM	14	C	0	1.342	-2.091	-2.372	C
HETATM	15	C	0	2.847	-1.836	-4.017	C
HETATM	16	H	0	3.779	-2.382	-4.154	H
HETATM	17	H	0	2.449	-1.532	-4.988	H
HETATM	18	C	0	2.932	-0.661	-3.017	C
HETATM	19	C	0	3.210	-3.110	1.040	C
HETATM	20	C	0	4.723	-3.200	0.728	C
HETATM	21	H	0	4.952	-3.673	-0.227	H
HETATM	22	H	0	5.302	-3.674	1.521	H
HETATM	23	C	0	4.056	-1.054	0.802	C
HETATM	24	C	0	4.267	0.408	0.708	C
HETATM	25	C	0	5.422	1.014	1.210	C
HETATM	26	H	0	6.209	0.413	1.647	H
HETATM	27	C	0	5.515	2.402	1.138	C
HETATM	28	H	0	6.386	2.912	1.532	H
HETATM	29	C	0	4.477	3.127	0.561	C
HETATM	30	H	0	4.500	4.207	0.494	H
HETATM	31	C	0	3.386	2.419	0.051	C
HETATM	32	C	0	2.242	3.076	-0.606	C

HETATM	33	C	0	0.912	4.775	-1.222	C
HETATM	34	H	0	0.304	5.284	-0.475	H
HETATM	35	H	0	1.152	5.453	-2.041	H
HETATM	36	C	0	0.318	3.421	-1.687	C
HETATM	37	N	0	-0.157	-2.081	2.295	N
HETATM	38	N	0	0.147	-2.624	-0.366	N
HETATM	39	N	0	1.817	-0.941	-2.075	N
HETATM	40	N	0	1.302	2.436	-1.192	N
HETATM	41	N	0	3.287	1.089	0.124	N
HETATM	42	N	0	2.940	-1.648	1.022	N
HETATM	43	O	0	-1.866	-3.532	2.460	O
HETATM	44	O	0	1.882	-2.748	-3.413	O
HETATM	45	O	0	2.178	4.412	-0.570	O
HETATM	46	O	0	5.153	-1.808	0.640	O
HETATM	47	Cu	0	1.070	-0.911	1.198	Cu
HETATM	48	Cu	0	0.978	0.456	-0.871	Cu
HETATM	49	C	0	0.170	0.698	0.865	C
HETATM	50	C	0	-0.636	1.611	1.167	C
HETATM	51	C	0	-1.439	2.673	1.435	C
HETATM	52	C	0	-2.692	2.977	0.813	C
HETATM	53	C	0	-3.598	3.926	1.369	C
HETATM	54	C	0	-3.080	2.284	-0.405	C
HETATM	55	C	0	-4.841	4.123	0.824	C
HETATM	56	H	0	-3.322	4.476	2.256	H
HETATM	57	C	0	-4.385	2.500	-0.925	C
HETATM	58	C	0	-5.237	3.392	-0.321	C
HETATM	59	H	0	-5.524	4.833	1.274	H
HETATM	60	H	0	-4.671	1.981	-1.830	H
HETATM	61	H	0	-6.224	3.554	-0.741	H
HETATM	62	N	0	-2.119	1.532	-0.964	N
HETATM	63	S	0	-2.290	0.381	-2.062	S
HETATM	64	O	0	-0.908	-0.153	-2.229	O
HETATM	65	O	0	-2.995	0.727	-3.305	O
HETATM	66	C	0	-3.281	-0.884	-1.236	C
HETATM	67	C	0	-3.226	-1.020	0.150	C
HETATM	68	C	0	-4.125	-1.684	-1.999	C

HETATM	69	H	0	-2.560	-0.389	0.726	H
HETATM	70	C	0	-4.039	-1.961	0.770	C
HETATM	71	H	0	-4.178	-1.536	-3.070	H
HETATM	72	C	0	-4.911	-2.643	-1.364	C
HETATM	73	H	0	-4.010	-2.063	1.848	H
HETATM	74	C	0	-4.886	-2.792	0.027	C
HETATM	75	H	0	-5.569	-3.269	-1.957	H
HETATM	76	C	0	-5.769	-3.799	0.721	C
HETATM	77	H	0	-6.672	-3.317	1.110	H
HETATM	78	H	0	-5.255	-4.258	1.569	H
HETATM	79	H	0	-6.087	-4.592	0.042	H
HETATM	80	C	0	2.829	-3.730	2.379	C
HETATM	81	H	0	3.402	-3.271	3.190	H
HETATM	82	H	0	1.771	-3.574	2.573	H
HETATM	83	H	0	3.035	-4.804	2.372	H
HETATM	84	C	0	0.106	3.275	-3.188	C
HETATM	85	H	0	1.030	3.479	-3.737	H
HETATM	86	H	0	-0.224	2.260	-3.413	H
HETATM	87	H	0	-0.666	3.967	-3.531	H
HETATM	88	C	0	0.437	-1.835	4.706	C
HETATM	89	H	0	0.975	-2.785	4.740	H
HETATM	90	H	0	1.148	-1.042	4.463	H
HETATM	91	H	0	0.028	-1.633	5.698	H
HETATM	92	C	0	2.789	0.704	-3.673	C
HETATM	93	H	0	1.819	0.781	-4.169	H
HETATM	94	H	0	2.844	1.493	-2.926	H
HETATM	95	H	0	3.580	0.858	-4.411	H
HETATM	96	H	0	-1.190	-0.909	3.654	H
HETATM	97	H	0	3.861	-0.699	-2.438	H
HETATM	98	H	0	-0.620	3.197	-1.180	H
HETATM	99	H	0	2.609	-3.554	0.245	H
HETATM	100	C	0	-0.888	3.635	2.485	C
HETATM	101	F	0	-1.625	3.636	3.619	F
HETATM	102	F	0	0.375	3.342	2.837	F
HETATM	103	F	0	-0.876	4.907	2.014	F

END

CONECT	1	2	37	88	96
CONECT	2	1	3	4	43
CONECT	3	2			
CONECT	4	2			
CONECT	5	6	37	43	
CONECT	6	5	7	38	
CONECT	7	6	8	9	
CONECT	8	7			
CONECT	9	7	10	11	
CONECT	10	9			
CONECT	11	9	12	13	
CONECT	12	11			
CONECT	13	11	14	38	
CONECT	14	13	39	44	
CONECT	15	16	17	18	44
CONECT	16	15			
CONECT	17	15			
CONECT	18	15	39	92	97
CONECT	19	20	42	80	99
CONECT	20	19	21	22	46
CONECT	21	20			
CONECT	22	20			
CONECT	23	24	42	46	
CONECT	24	23	25	41	
CONECT	25	24	26	27	
CONECT	26	25			
CONECT	27	25	28	29	
CONECT	28	27			
CONECT	29	27	30	31	
CONECT	30	29			
CONECT	31	29	32	41	
CONECT	32	31	40	45	
CONECT	33	34	35	36	45
CONECT	34	33			
CONECT	35	33			
CONECT	36	33	40	84	98

CONECT	37	5	1		
CONECT	38	6	13		
CONECT	39	14	18		
CONECT	40	32	36		
CONECT	41	24	31		
CONECT	42	19	23		
CONECT	43	2	5		
CONECT	44	14	15		
CONECT	45	32	33		
CONECT	46	20	23		
CONECT	47	49			
CONECT	48	49			
CONECT	49	47	48	50	
CONECT	50	49	51		
CONECT	51	50	52	100	
CONECT	52	51	53	54	
CONECT	53	52	55	56	
CONECT	54	52	57	62	
CONECT	55	53	58	59	
CONECT	56	53			
CONECT	57	54	58	60	
CONECT	58	57	55	61	
CONECT	59	55			
CONECT	60	57			
CONECT	61	58			
CONECT	62	54	63		
CONECT	63	62	64	65	66
CONECT	64	63			
CONECT	65	63			
CONECT	66	63	67	68	
CONECT	67	66	69	70	
CONECT	68	66	71	72	
CONECT	69	67			
CONECT	70	67	73	74	
CONECT	71	68			
CONECT	72	68	74	75	

```
CONECT   73   70
CONECT   74   72   70   76
CONECT   75   72
CONECT   76   74   77   78   79
CONECT   77   76
CONECT   78   76
CONECT   79   76
CONECT   80   19   81   82   83
CONECT   81   80
CONECT   82   80
CONECT   83   80
CONECT   84   36   85   86   87
CONECT   85   84
CONECT   86   84
CONECT   87   84
CONECT   88    1   89   90   91
CONECT   89   88
CONECT   90   88
CONECT   91   88
CONECT   92   18   93   94   95
CONECT   93   92
CONECT   94   92
CONECT   95   92
CONECT   96    1
CONECT   97   18
CONECT   98   36
CONECT   99   19
CONECT  100   51  101  102  103
CONECT  101  100
CONECT  102  100
CONECT  103  100
```

I’ (with L1)

TITLE	Me_cis-2cu opt b3lyp/6-311G** + D3						
REMARK	1 File created by GaussView 6.0.16						
HETATM	1	C	0	0.763	-2.417	-3.363	C
HETATM	2	C	0	1.879	-3.481	-3.287	C
HETATM	3	H	0	2.816	-3.188	-3.756	H
HETATM	4	H	0	1.560	-4.454	-3.672	H
HETATM	5	C	0	1.107	-3.007	-1.239	C
HETATM	6	C	0	1.062	-3.011	0.231	C
HETATM	7	C	0	2.036	-3.637	1.008	C
HETATM	8	H	0	2.835	-4.188	0.534	H
HETATM	9	C	0	1.959	-3.494	2.389	C
HETATM	10	H	0	2.709	-3.944	3.027	H
HETATM	11	C	0	0.917	-2.756	2.937	C
HETATM	12	H	0	0.820	-2.615	4.004	H
HETATM	13	C	0	-0.012	-2.172	2.072	C
HETATM	14	C	0	-1.151	-1.394	2.606	C
HETATM	15	C	0	-2.668	-0.886	4.179	C
HETATM	16	H	0	-3.555	-1.459	4.443	H
HETATM	17	H	0	-2.305	-0.342	5.053	H
HETATM	18	C	0	-2.839	0.026	2.942	C
HETATM	19	C	0	-3.004	-3.288	-0.440	C
HETATM	20	C	0	-4.498	-3.414	-0.055	C
HETATM	21	H	0	-4.666	-3.695	0.984	H
HETATM	22	H	0	-5.058	-4.080	-0.711	H
HETATM	23	C	0	-3.997	-1.293	-0.611	C
HETATM	24	C	0	-4.321	0.138	-0.815	C
HETATM	25	C	0	-5.551	0.539	-1.346	C
HETATM	26	H	0	-6.305	-0.196	-1.593	H
HETATM	27	C	0	-5.760	1.901	-1.549	C
HETATM	28	H	0	-6.693	2.253	-1.972	H
HETATM	29	C	0	-4.761	2.806	-1.202	C
HETATM	30	H	0	-4.879	3.872	-1.345	H
HETATM	31	C	0	-3.585	2.302	-0.641	C
HETATM	32	C	0	-2.477	3.164	-0.194	C

HETATM	33	C	0	-1.314	5.064	0.073	C
HETATM	34	H	0	-0.781	5.545	-0.746	H
HETATM	35	H	0	-1.622	5.813	0.805	H
HETATM	36	C	0	-0.555	3.867	0.702	C
HETATM	37	N	0	0.268	-2.371	-1.970	N
HETATM	38	N	0	0.053	-2.299	0.749	N
HETATM	39	N	0	-1.715	-0.385	2.060	N
HETATM	40	N	0	-1.463	2.729	0.452	N
HETATM	41	N	0	-3.372	0.996	-0.456	N
HETATM	42	N	0	-2.848	-1.843	-0.755	N
HETATM	43	O	0	2.125	-3.634	-1.854	O
HETATM	44	O	0	-1.630	-1.829	3.785	O
HETATM	45	O	0	-2.535	4.472	-0.487	O
HETATM	46	O	0	-5.028	-2.067	-0.236	O
HETATM	47	Cu	0	-1.041	-1.058	-1.142	Cu
HETATM	48	Cu	0	-0.990	0.756	0.540	Cu
HETATM	49	C	0	-0.197	0.618	-1.226	C
HETATM	50	C	0	0.606	1.414	-1.767	C
HETATM	51	C	0	1.414	2.325	-2.386	C
HETATM	52	C	0	2.599	2.887	-1.810	C
HETATM	53	C	0	3.487	3.664	-2.607	C
HETATM	54	C	0	2.955	2.645	-0.424	C
HETATM	55	C	0	4.688	4.114	-2.118	C
HETATM	56	H	0	3.228	3.885	-3.634	H
HETATM	57	C	0	4.219	3.105	0.040	C
HETATM	58	C	0	5.056	3.816	-0.786	C
HETATM	59	H	0	5.355	4.690	-2.748	H
HETATM	60	H	0	4.485	2.919	1.073	H
HETATM	61	H	0	6.007	4.169	-0.404	H
HETATM	62	N	0	2.020	2.030	0.323	N
HETATM	63	S	0	2.270	1.221	1.680	S
HETATM	64	O	0	0.936	0.645	2.009	O
HETATM	65	O	0	2.943	1.929	2.781	O
HETATM	66	C	0	3.362	-0.139	1.202	C
HETATM	67	C	0	3.329	-0.636	-0.099	C
HETATM	68	C	0	4.261	-0.645	2.136	C

HETATM	69	H	0	2.618	-0.232	-0.810	H
HETATM	70	C	0	4.220	-1.640	-0.464	C
HETATM	71	H	0	4.292	-0.220	3.131	H
HETATM	72	C	0	5.126	-1.671	1.762	C
HETATM	73	H	0	4.202	-2.026	-1.476	H
HETATM	74	C	0	5.125	-2.178	0.458	C
HETATM	75	H	0	5.827	-2.067	2.490	H
HETATM	76	C	0	6.096	-3.254	0.041	C
HETATM	77	H	0	6.994	-2.812	-0.404	H
HETATM	78	H	0	5.656	-3.919	-0.707	H
HETATM	79	H	0	6.418	-3.858	0.891	H
HETATM	80	C	0	-2.605	-4.163	-1.622	C
HETATM	81	H	0	-3.230	-3.939	-2.491	H
HETATM	82	H	0	-1.566	-3.978	-1.885	H
HETATM	83	H	0	-2.725	-5.219	-1.370	H
HETATM	84	C	0	-0.250	4.009	2.188	C
HETATM	85	H	0	-1.156	4.254	2.750	H
HETATM	86	H	0	0.161	3.074	2.570	H
HETATM	87	H	0	0.489	4.797	2.350	H
HETATM	88	C	0	-0.343	-2.712	-4.365	C
HETATM	89	H	0	-0.784	-3.695	-4.182	H
HETATM	90	H	0	-1.130	-1.960	-4.282	H
HETATM	91	H	0	0.045	-2.691	-5.386	H
HETATM	92	C	0	-2.784	1.509	3.277	C
HETATM	93	H	0	-1.816	1.756	3.716	H
HETATM	94	H	0	-2.904	2.108	2.378	H
HETATM	95	H	0	-3.575	1.768	3.986	H
HETATM	96	H	0	1.181	-1.424	-3.566	H
HETATM	97	H	0	-3.768	-0.200	2.407	H
HETATM	98	H	0	0.370	3.639	0.171	H
HETATM	99	H	0	-2.350	-3.497	0.409	H
HETATM	100	C	0	1.003	2.712	-3.795	C
HETATM	101	H	0	0.930	3.798	-3.899	H
HETATM	102	H	0	1.739	2.362	-4.526	H
HETATM	103	H	0	0.039	2.270	-4.042	H

END

CONECT	1	2	37	88	96
CONECT	2	1	3	4	43
CONECT	3	2			
CONECT	4	2			
CONECT	5	6	37	43	
CONECT	6	5	7	38	
CONECT	7	6	8	9	
CONECT	8	7			
CONECT	9	7	10	11	
CONECT	10	9			
CONECT	11	9	12	13	
CONECT	12	11			
CONECT	13	11	14	38	
CONECT	14	13	39	44	
CONECT	15	16	17	18	44
CONECT	16	15			
CONECT	17	15			
CONECT	18	15	39	92	97
CONECT	19	20	42	80	99
CONECT	20	19	21	22	46
CONECT	21	20			
CONECT	22	20			
CONECT	23	24	42	46	
CONECT	24	23	25	41	
CONECT	25	24	26	27	
CONECT	26	25			
CONECT	27	25	28	29	
CONECT	28	27			
CONECT	29	27	30	31	
CONECT	30	29			
CONECT	31	29	32	41	
CONECT	32	31	40	45	
CONECT	33	34	35	36	45
CONECT	34	33			
CONECT	35	33			
CONECT	36	33	40	84	98

CONECT	37	1	5		
CONECT	38	6	13		
CONECT	39	14	18		
CONECT	40	32	36		
CONECT	41	24	31		
CONECT	42	23	19		
CONECT	43	2	5		
CONECT	44	14	15		
CONECT	45	32	33		
CONECT	46	20	23		
CONECT	47	48	49		
CONECT	48	47	49		
CONECT	49	47	48	50	
CONECT	50	49	51		
CONECT	51	50	52	100	
CONECT	52	51	53	54	
CONECT	53	52	55	56	
CONECT	54	52	57	62	
CONECT	55	53	58	59	
CONECT	56	53			
CONECT	57	54	58	60	
CONECT	58	57	55	61	
CONECT	59	55			
CONECT	60	57			
CONECT	61	58			
CONECT	62	54	63		
CONECT	63	62	64	65	66
CONECT	64	63			
CONECT	65	63			
CONECT	66	63	67	68	
CONECT	67	66	69	70	
CONECT	68	66	71	72	
CONECT	69	67			
CONECT	70	67	73	74	
CONECT	71	68			
CONECT	72	68	74	75	

CONECT	73	70			
CONECT	74	70	72	76	
CONECT	75	72			
CONECT	76	74	77	78	79
CONECT	77	76			
CONECT	78	76			
CONECT	79	76			
CONECT	80	19	81	82	83
CONECT	81	80			
CONECT	82	80			
CONECT	83	80			
CONECT	84	36	85	86	87
CONECT	85	84			
CONECT	86	84			
CONECT	87	84			
CONECT	88	1	89	90	91
CONECT	89	88			
CONECT	90	88			
CONECT	91	88			
CONECT	92	18	93	94	95
CONECT	93	92			
CONECT	94	92			
CONECT	95	92			
CONECT	96	1			
CONECT	97	18			
CONECT	98	36			
CONECT	99	19			
CONECT	100	51	101	102	103
CONECT	101	100			
CONECT	102	100			
CONECT	103	100			

II (with L1)

TITLE	Indoline cis-cf3-ind-A opt b3lyp/6-311G** + D3						
REMARK	1 File created by GaussView 6.0.16						
HETATM	1	C	0	-1.537	-3.129	1.380	C
HETATM	2	C	0	-1.971	-4.197	0.348	C
HETATM	3	H	0	-2.806	-3.878	-0.276	H
HETATM	4	H	0	-2.176	-5.174	0.785	H
HETATM	5	C	0	0.013	-3.323	-0.210	C
HETATM	6	C	0	1.172	-3.126	-1.109	C
HETATM	7	C	0	1.765	-4.203	-1.775	C
HETATM	8	H	0	1.374	-5.204	-1.644	H
HETATM	9	C	0	2.858	-3.943	-2.596	C
HETATM	10	H	0	3.359	-4.750	-3.116	H
HETATM	11	C	0	3.287	-2.629	-2.757	C
HETATM	12	H	0	4.116	-2.379	-3.405	H
HETATM	13	C	0	2.595	-1.617	-2.082	C
HETATM	14	C	0	2.910	-0.197	-2.334	C
HETATM	15	C	0	4.143	1.492	-3.143	C
HETATM	16	H	0	4.945	1.763	-2.454	H
HETATM	17	H	0	4.414	1.764	-4.162	H
HETATM	18	C	0	2.759	1.996	-2.701	C
HETATM	19	C	0	2.734	-2.780	2.105	C
HETATM	20	C	0	4.264	-2.915	2.019	C
HETATM	21	H	0	4.605	-3.380	1.092	H
HETATM	22	H	0	4.718	-3.411	2.875	H
HETATM	23	C	0	3.641	-0.780	1.725	C
HETATM	24	C	0	3.873	0.671	1.630	C
HETATM	25	C	0	5.066	1.231	2.103	C
HETATM	26	H	0	5.867	0.591	2.447	H
HETATM	27	C	0	5.168	2.617	2.148	C
HETATM	28	H	0	6.068	3.088	2.524	H
HETATM	29	C	0	4.089	3.389	1.729	C
HETATM	30	H	0	4.104	4.469	1.783	H
HETATM	31	C	0	2.963	2.729	1.227	C
HETATM	32	C	0	1.785	3.484	0.753	C
HETATM	33	C	0	0.293	5.147	0.848	C

HETATM	34	H	0	-0.381	5.077	1.703	H
HETATM	35	H	0	0.387	6.184	0.529	H
HETATM	36	C	0	-0.059	4.167	-0.295	C
HETATM	37	N	0	-0.269	-2.600	0.806	N
HETATM	38	N	0	1.578	-1.864	-1.253	N
HETATM	39	N	0	2.208	0.818	-1.979	N
HETATM	40	N	0	0.936	3.073	-0.111	N
HETATM	41	N	0	2.856	1.402	1.168	N
HETATM	42	N	0	2.503	-1.377	1.668	N
HETATM	43	O	0	-0.809	-4.338	-0.529	O
HETATM	44	O	0	3.996	0.039	-3.094	O
HETATM	45	O	0	1.611	4.699	1.299	O
HETATM	46	O	0	4.721	-1.531	2.009	O
HETATM	47	Cu	0	0.696	-0.747	1.134	Cu
HETATM	48	Cu	0	0.741	1.062	-0.687	Cu
HETATM	49	C	0	-0.540	0.489	0.534	C
HETATM	50	C	0	-1.835	0.840	0.790	C
HETATM	51	C	0	-2.715	0.393	1.794	C
HETATM	52	C	0	-4.039	0.872	1.545	C
HETATM	53	C	0	-5.242	0.738	2.258	C
HETATM	54	C	0	-3.981	1.729	0.421	C
HETATM	55	C	0	-6.347	1.463	1.841	C
HETATM	56	H	0	-5.291	0.089	3.123	H
HETATM	57	C	0	-5.078	2.493	0.032	C
HETATM	58	C	0	-6.267	2.330	0.741	C
HETATM	59	H	0	-7.283	1.370	2.379	H
HETATM	60	H	0	-5.014	3.170	-0.806	H
HETATM	61	H	0	-7.142	2.892	0.438	H
HETATM	62	N	0	-2.655	1.714	-0.093	N
HETATM	63	S	0	-2.463	1.580	-1.798	S
HETATM	64	O	0	-1.057	1.876	-2.092	O
HETATM	65	O	0	-3.508	2.394	-2.397	O
HETATM	66	C	0	-2.754	-0.135	-2.181	C
HETATM	67	C	0	-1.667	-0.993	-2.342	C
HETATM	68	C	0	-4.067	-0.583	-2.312	C
HETATM	69	H	0	-0.666	-0.628	-2.169	H

HETATM	70	C	0	-1.914	-2.318	-2.675	C
HETATM	71	H	0	-4.893	0.105	-2.190	H
HETATM	72	C	0	-4.289	-1.922	-2.616	C
HETATM	73	H	0	-1.080	-2.992	-2.827	H
HETATM	74	C	0	-3.220	-2.805	-2.813	C
HETATM	75	H	0	-5.307	-2.281	-2.717	H
HETATM	76	C	0	-3.458	-4.245	-3.191	C
HETATM	77	H	0	-4.429	-4.598	-2.839	H
HETATM	78	H	0	-2.681	-4.893	-2.778	H
HETATM	79	H	0	-3.441	-4.364	-4.279	H
HETATM	80	C	0	2.166	-2.956	3.510	C
HETATM	81	H	0	2.714	-2.337	4.225	H
HETATM	82	H	0	1.123	-2.646	3.518	H
HETATM	83	H	0	2.228	-4.000	3.829	H
HETATM	84	C	0	0.059	4.795	-1.680	C
HETATM	85	H	0	1.067	5.189	-1.840	H
HETATM	86	H	0	-0.172	4.065	-2.450	H
HETATM	87	H	0	-0.650	5.622	-1.772	H
HETATM	88	C	0	-1.337	-3.701	2.779	C
HETATM	89	H	0	-0.596	-4.505	2.764	H
HETATM	90	H	0	-1.019	-2.929	3.475	H
HETATM	91	H	0	-2.282	-4.107	3.146	H
HETATM	92	C	0	1.828	2.344	-3.861	C
HETATM	93	H	0	1.843	1.547	-4.610	H
HETATM	94	H	0	0.806	2.448	-3.500	H
HETATM	95	H	0	2.140	3.276	-4.338	H
HETATM	96	H	0	-2.249	-2.305	1.410	H
HETATM	97	H	0	2.826	2.826	-2.000	H
HETATM	98	H	0	-1.051	3.737	-0.159	H
HETATM	99	H	0	2.235	-3.452	1.409	H
HETATM	100	C	0	-2.356	-0.147	3.126	C
HETATM	101	F	0	-1.076	-0.578	3.247	F
HETATM	102	F	0	-2.542	0.762	4.111	F
HETATM	103	F	0	-3.147	-1.217	3.463	F

END

CONECT 1 2 37 88 96

CONECT	2	1	3	4	43
CONECT	3	2			
CONECT	4	2			
CONECT	5	6	37	43	
CONECT	6	5	7	38	
CONECT	7	6	8	9	
CONECT	8	7			
CONECT	9	7	10	11	
CONECT	10	9			
CONECT	11	9	12	13	
CONECT	12	11			
CONECT	13	11	14	38	
CONECT	14	13	39	44	
CONECT	15	16	17	18	44
CONECT	16	15			
CONECT	17	15			
CONECT	18	15	39	92	97
CONECT	19	20	42	80	99
CONECT	20	19	21	22	46
CONECT	21	20			
CONECT	22	20			
CONECT	23	24	42	46	
CONECT	24	23	25	41	
CONECT	25	24	26	27	
CONECT	26	25			
CONECT	27	25	28	29	
CONECT	28	27			
CONECT	29	27	30	31	
CONECT	30	29			
CONECT	31	29	32	41	
CONECT	32	31	40	45	
CONECT	33	34	35	36	45
CONECT	34	33			
CONECT	35	33			
CONECT	36	33	40	84	98
CONECT	37	1	5		

CONECT	38	6	13		
CONECT	39	14	18	48	
CONECT	40	36	32		
CONECT	41	24	31		
CONECT	42	19	23		
CONECT	43	2	5		
CONECT	44	14	15		
CONECT	45	33	32		
CONECT	46	20	23		
CONECT	47	49			
CONECT	48	39	49		
CONECT	49	47	48	50	
CONECT	50	49	51	62	
CONECT	51	50	52	100	
CONECT	52	51	53	54	
CONECT	53	52	55	56	
CONECT	54	52	57	62	
CONECT	55	53	58	59	
CONECT	56	53			
CONECT	57	54	58	60	
CONECT	58	55	57	61	
CONECT	59	55			
CONECT	60	57			
CONECT	61	58			
CONECT	62	50	54	63	
CONECT	63	62	64	65	66
CONECT	64	63			
CONECT	65	63			
CONECT	66	63	67	68	
CONECT	67	66	69	70	
CONECT	68	66	71	72	
CONECT	69	67			
CONECT	70	67	73	74	
CONECT	71	68			
CONECT	72	68	74	75	
CONECT	73	70			

```
CONECT   74   72   70   76
CONECT   75   72
CONECT   76   74   77   78   79
CONECT   77   76
CONECT   78   76
CONECT   79   76
CONECT   80   19   81   82   83
CONECT   81   80
CONECT   82   80
CONECT   83   80
CONECT   84   36   85   86   87
CONECT   85   84
CONECT   86   84
CONECT   87   84
CONECT   88    1   89   90   91
CONECT   89   88
CONECT   90   88
CONECT   91   88
CONECT   92   18   93   94   95
CONECT   93   92
CONECT   94   92
CONECT   95   92
CONECT   96    1
CONECT   97   18
CONECT   98   36
CONECT   99   19
CONECT  100   51  101  102  103
CONECT  101  100
CONECT  102  100
CONECT  103  100
```

II’, II” (with L1)

TITLE	Indoline cis-me-ind-A opt b3lyp/6-311G** + D3						
REMARK	1 File created by GaussView 6.0.16						
HETATM	1	C	0	0.523	-3.351	-1.658	C
HETATM	2	C	0	0.042	-4.781	-1.343	C
HETATM	3	H	0	0.801	-5.425	-0.906	H
HETATM	4	H	0	-0.419	-5.271	-2.205	H
HETATM	5	C	0	-1.292	-3.263	-0.382	C
HETATM	6	C	0	-2.336	-2.758	0.534	C
HETATM	7	C	0	-3.424	-3.540	0.929	C
HETATM	8	H	0	-3.536	-4.548	0.551	H
HETATM	9	C	0	-4.339	-2.979	1.816	C
HETATM	10	H	0	-5.207	-3.541	2.138	H
HETATM	11	C	0	-4.112	-1.698	2.307	C
HETATM	12	H	0	-4.778	-1.242	3.026	H
HETATM	13	C	0	-2.967	-1.011	1.877	C
HETATM	14	C	0	-2.587	0.274	2.493	C
HETATM	15	C	0	-2.876	2.006	3.890	C
HETATM	16	H	0	-3.479	2.793	3.430	H
HETATM	17	H	0	-2.962	2.052	4.974	H
HETATM	18	C	0	-1.431	1.966	3.371	C
HETATM	19	C	0	-3.231	-1.437	-2.704	C
HETATM	20	C	0	-4.717	-1.040	-2.725	C
HETATM	21	H	0	-5.307	-1.540	-1.953	H
HETATM	22	H	0	-5.197	-1.162	-3.696	H
HETATM	23	C	0	-3.458	0.649	-1.944	C
HETATM	24	C	0	-3.222	2.051	-1.561	C
HETATM	25	C	0	-4.132	3.043	-1.954	C
HETATM	26	H	0	-5.058	2.768	-2.440	H
HETATM	27	C	0	-3.792	4.372	-1.737	C
HETATM	28	H	0	-4.462	5.167	-2.041	H
HETATM	29	C	0	-2.567	4.667	-1.148	C
HETATM	30	H	0	-2.237	5.686	-0.995	H
HETATM	31	C	0	-1.753	3.602	-0.748	C
HETATM	32	C	0	-0.435	3.852	-0.129	C
HETATM	33	C	0	1.500	4.962	0.120	C

HETATM	34	H	0	2.239	4.920	-0.680	H
HETATM	35	H	0	1.619	5.887	0.685	H
HETATM	36	C	0	1.475	3.694	1.012	C
HETATM	37	N	0	-0.592	-2.521	-1.147	N
HETATM	38	N	0	-2.117	-1.520	0.983	N
HETATM	39	N	0	-1.508	0.945	2.290	N
HETATM	40	N	0	0.162	3.078	0.693	N
HETATM	41	N	0	-2.073	2.320	-0.934	N
HETATM	42	N	0	-2.591	-0.304	-1.985	N
HETATM	43	O	0	-1.004	-4.581	-0.336	O
HETATM	44	O	0	-3.420	0.724	3.454	O
HETATM	45	O	0	0.180	4.990	-0.504	O
HETATM	46	O	0	-4.694	0.379	-2.413	O
HETATM	47	Cu	0	-0.741	-0.454	-1.318	Cu
HETATM	48	Cu	0	-0.185	1.011	0.856	Cu
HETATM	49	C	0	0.767	0.320	-0.595	C
HETATM	50	C	0	2.095	0.425	-0.984	C
HETATM	51	C	0	2.633	0.605	-2.263	C
HETATM	52	C	0	4.042	0.778	-2.170	C
HETATM	53	C	0	5.013	1.003	-3.162	C
HETATM	54	C	0	4.424	0.659	-0.807	C
HETATM	55	C	0	6.341	1.081	-2.791	C
HETATM	56	H	0	4.715	1.108	-4.199	H
HETATM	57	C	0	5.771	0.740	-0.434	C
HETATM	58	C	0	6.707	0.948	-1.437	C
HETATM	59	H	0	7.108	1.249	-3.537	H
HETATM	60	H	0	6.072	0.657	0.598	H
HETATM	61	H	0	7.753	1.023	-1.164	H
HETATM	62	N	0	3.250	0.507	-0.063	N
HETATM	63	S	0	3.175	0.019	1.575	S
HETATM	64	O	0	2.163	0.841	2.233	O
HETATM	65	O	0	4.544	0.027	2.070	O
HETATM	66	C	0	2.606	-1.679	1.552	C
HETATM	67	C	0	1.272	-1.969	1.820	C
HETATM	68	C	0	3.548	-2.688	1.360	C
HETATM	69	H	0	0.550	-1.168	1.886	H

HETATM	70	C	0	0.890	-3.300	1.927	C
HETATM	71	H	0	4.586	-2.440	1.179	H
HETATM	72	C	0	3.135	-4.015	1.437	C
HETATM	73	H	0	-0.141	-3.535	2.162	H
HETATM	74	C	0	1.807	-4.341	1.739	C
HETATM	75	H	0	3.861	-4.806	1.288	H
HETATM	76	C	0	1.371	-5.775	1.897	C
HETATM	77	H	0	2.025	-6.459	1.350	H
HETATM	78	H	0	0.346	-5.914	1.547	H
HETATM	79	H	0	1.403	-6.069	2.951	H
HETATM	80	C	0	-2.595	-1.579	-4.084	C
HETATM	81	H	0	-2.787	-0.689	-4.688	H
HETATM	82	H	0	-1.516	-1.691	-3.976	H
HETATM	83	H	0	-2.992	-2.452	-4.609	H
HETATM	84	C	0	1.608	3.993	2.500	C
HETATM	85	H	0	0.810	4.665	2.831	H
HETATM	86	H	0	1.572	3.071	3.075	H
HETATM	87	H	0	2.567	4.476	2.700	H
HETATM	88	C	0	0.848	-3.063	-3.113	C
HETATM	89	H	0	0.027	-3.365	-3.768	H
HETATM	90	H	0	1.012	-1.990	-3.227	H
HETATM	91	H	0	1.753	-3.588	-3.424	H
HETATM	92	C	0	-0.410	1.503	4.411	C
HETATM	93	H	0	-0.766	0.598	4.913	H
HETATM	94	H	0	0.539	1.273	3.928	H
HETATM	95	H	0	-0.252	2.279	5.164	H
HETATM	96	H	0	1.387	-3.093	-1.036	H
HETATM	97	H	0	-1.125	2.910	2.925	H
HETATM	98	H	0	2.241	2.976	0.715	H
HETATM	99	H	0	-3.075	-2.347	-2.129	H
HETATM	100	C	0	1.850	0.674	-3.529	C
HETATM	101	H	0	2.261	-0.008	-4.280	H
HETATM	102	H	0	0.806	0.428	-3.331	H
HETATM	103	H	0	1.884	1.682	-3.956	H

END

CONECT 1 2 37 88 96

```
CONECT    2    1    3    4   43
CONECT    3    2
CONECT    4    2
CONECT    5    6   37   43
CONECT    6    5    7   38
CONECT    7    6    8    9
CONECT    8    7
CONECT    9    7   10   11
CONECT   10    9
CONECT   11    9   12   13
CONECT   12   11
CONECT   13   11   14   38
CONECT   14   13   39   44
CONECT   15   16   17   18   44
CONECT   16   15
CONECT   17   15
CONECT   18   15   39   92   97
CONECT   19   20   42   80   99
CONECT   20   19   21   22   46
CONECT   21   20
CONECT   22   20
CONECT   23   24   42   46
CONECT   24   23   25   41
CONECT   25   24   26   27
CONECT   26   25
CONECT   27   25   28   29
CONECT   28   27
CONECT   29   27   30   31
CONECT   30   29
CONECT   31   29   32   41
CONECT   32   31   40   45
CONECT   33   34   35   36   45
CONECT   34   33
CONECT   35   33
CONECT   36   33   40   84   98
CONECT   37    1    5
```

CONECT	38	6	13		
CONECT	39	14	18	48	
CONECT	40	32	36		
CONECT	41	24	31		
CONECT	42	19	23	47	
CONECT	43	2	5		
CONECT	44	14	15		
CONECT	45	32	33		
CONECT	46	20	23		
CONECT	47	42	49		
CONECT	48	39	49		
CONECT	49	47	48	50	
CONECT	50	49	51	62	
CONECT	51	50	52	100	
CONECT	52	51	53	54	
CONECT	53	52	55	56	
CONECT	54	52	57	62	
CONECT	55	53	58	59	
CONECT	56	53			
CONECT	57	54	58	60	
CONECT	58	55	57	61	
CONECT	59	55			
CONECT	60	57			
CONECT	61	58			
CONECT	62	50	54	63	
CONECT	63	62	64	65	66
CONECT	64	63			
CONECT	65	63			
CONECT	66	63	67	68	
CONECT	67	66	69	70	
CONECT	68	66	71	72	
CONECT	69	67			
CONECT	70	67	73	74	
CONECT	71	68			
CONECT	72	68	74	75	
CONECT	73	70			

```
CONECT   74   72   70   76
CONECT   75   72
CONECT   76   74   77   78   79
CONECT   77   76
CONECT   78   76
CONECT   79   76
CONECT   80   19   81   82   83
CONECT   81   80
CONECT   82   80
CONECT   83   80
CONECT   84   36   85   86   87
CONECT   85   84
CONECT   86   84
CONECT   87   84
CONECT   88    1   89   90   91
CONECT   89   88
CONECT   90   88
CONECT   91   88
CONECT   92   18   93   94   95
CONECT   93   92
CONECT   94   92
CONECT   95   92
CONECT   96    1
CONECT   97   18
CONECT   98   36
CONECT   99   19
CONECT  100   51  101  102  103
CONECT  101  100
CONECT  102  100
CONECT  103  100
```

I" (with L1)

TITLE H_cis-2cu opt b3lyp/6-311G** + D3

REMARK 1 File created by GaussView 6.0.16

HETATM	1	C	0	0.742	2.594	3.295	C
HETATM	2	C	0	1.901	3.608	3.172	C
HETATM	3	H	0	2.816	3.312	3.681	H
HETATM	4	H	0	1.613	4.616	3.480	H
HETATM	5	C	0	1.159	3.014	1.143	C
HETATM	6	C	0	1.143	2.919	-0.324	C
HETATM	7	C	0	2.139	3.479	-1.122	C
HETATM	8	H	0	2.939	4.047	-0.671	H
HETATM	9	C	0	2.082	3.250	-2.493	C
HETATM	10	H	0	2.849	3.646	-3.146	H
HETATM	11	C	0	1.037	2.494	-3.010	C
HETATM	12	H	0	0.954	2.287	-4.068	H
HETATM	13	C	0	0.085	1.981	-2.126	C
HETATM	14	C	0	-1.057	1.187	-2.627	C
HETATM	15	C	0	-2.566	0.608	-4.186	C
HETATM	16	H	0	-3.441	1.183	-4.485	H
HETATM	17	H	0	-2.210	0.011	-5.028	H
HETATM	18	C	0	-2.759	-0.231	-2.901	C
HETATM	19	C	0	-2.946	3.318	0.210	C
HETATM	20	C	0	-4.437	3.446	-0.186	C
HETATM	21	H	0	-4.599	3.680	-1.238	H
HETATM	22	H	0	-4.989	4.151	0.437	H
HETATM	23	C	0	-3.977	1.357	0.509	C
HETATM	24	C	0	-4.329	-0.051	0.803	C
HETATM	25	C	0	-5.578	-0.395	1.329	C
HETATM	26	H	0	-6.324	0.368	1.508	H
HETATM	27	C	0	-5.818	-1.736	1.618	C
HETATM	28	H	0	-6.767	-2.042	2.041	H
HETATM	29	C	0	-4.828	-2.679	1.357	C
HETATM	30	H	0	-4.970	-3.731	1.568	H
HETATM	31	C	0	-3.630	-2.235	0.793	C
HETATM	32	C	0	-2.528	-3.145	0.434	C
HETATM	33	C	0	-1.396	-5.079	0.325	C

HETATM	34	H	0	-0.897	-5.510	1.191	H
HETATM	35	H	0	-1.696	-5.870	-0.364	H
HETATM	36	C	0	-0.596	-3.939	-0.359	C
HETATM	37	N	0	0.283	2.458	1.897	N
HETATM	38	N	0	0.131	2.190	-0.812	N
HETATM	39	N	0	-1.645	0.223	-2.028	N
HETATM	40	N	0	-1.488	-2.770	-0.208	N
HETATM	41	N	0	-3.388	-0.948	0.526	N
HETATM	42	N	0	-2.823	1.900	0.639	N
HETATM	43	O	0	2.183	3.651	1.737	O
HETATM	44	O	0	-1.512	1.553	-3.839	O
HETATM	45	O	0	-2.622	-4.430	0.805	O
HETATM	46	O	0	-4.986	2.117	0.057	O
HETATM	47	Cu	0	-1.038	1.121	1.128	Cu
HETATM	48	Cu	0	-0.982	-0.816	-0.412	Cu
HETATM	49	C	0	-0.235	-0.561	1.361	C
HETATM	50	C	0	0.533	-1.328	1.990	C
HETATM	51	C	0	1.362	-2.208	2.614	C
HETATM	52	H	0	1.128	-2.437	3.654	H
HETATM	53	C	0	2.515	-2.848	2.096	C
HETATM	54	C	0	3.338	-3.607	2.977	C
HETATM	55	C	0	2.909	-2.712	0.705	C
HETATM	56	C	0	4.526	-4.144	2.554	C
HETATM	57	H	0	3.017	-3.731	4.006	H
HETATM	58	C	0	4.162	-3.258	0.311	C
HETATM	59	C	0	4.937	-3.950	1.213	C
HETATM	60	H	0	5.150	-4.708	3.235	H
HETATM	61	H	0	4.469	-3.155	-0.722	H
HETATM	62	H	0	5.880	-4.372	0.883	H
HETATM	63	N	0	2.012	-2.100	-0.085	N
HETATM	64	S	0	2.308	-1.403	-1.498	S
HETATM	65	O	0	0.994	-0.824	-1.894	O
HETATM	66	O	0	2.987	-2.211	-2.523	O
HETATM	67	C	0	3.422	-0.037	-1.093	C
HETATM	68	C	0	3.383	0.548	0.171	C
HETATM	69	C	0	4.341	0.387	-2.048	C

HETATM	70	H	0	2.658	0.205	0.899	H
HETATM	71	C	0	4.287	1.558	0.477	C
HETATM	72	H	0	4.377	-0.105	-3.012	H
HETATM	73	C	0	5.221	1.420	-1.733	C
HETATM	74	H	0	4.263	2.014	1.460	H
HETATM	75	C	0	5.213	2.016	-0.467	C
HETATM	76	H	0	5.937	1.754	-2.477	H
HETATM	77	C	0	6.199	3.100	-0.111	C
HETATM	78	H	0	7.087	2.673	0.366	H
HETATM	79	H	0	5.767	3.818	0.590	H
HETATM	80	H	0	6.536	3.644	-0.996	H
HETATM	81	C	0	-2.528	4.278	1.317	C
HETATM	82	H	0	-3.154	4.136	2.202	H
HETATM	83	H	0	-1.492	4.097	1.593	H
HETATM	84	H	0	-2.631	5.313	0.981	H
HETATM	85	C	0	-0.256	-4.182	-1.824	C
HETATM	86	H	0	-1.152	-4.451	-2.392	H
HETATM	87	H	0	0.178	-3.281	-2.256	H
HETATM	88	H	0	0.473	-4.990	-1.916	H
HETATM	89	C	0	-0.379	3.011	4.236	C
HETATM	90	H	0	-0.776	3.990	3.959	H
HETATM	91	H	0	-1.192	2.283	4.191	H
HETATM	92	H	0	-0.020	3.061	5.266	H
HETATM	93	C	0	-2.705	-1.731	-3.152	C
HETATM	94	H	0	-1.731	-2.005	-3.564	H
HETATM	95	H	0	-2.839	-2.278	-2.223	H
HETATM	96	H	0	-3.487	-2.027	-3.856	H
HETATM	97	H	0	1.114	1.606	3.589	H
HETATM	98	H	0	-3.694	0.027	-2.394	H
HETATM	99	H	0	0.318	-3.696	0.182	H
HETATM	100	H	0	-2.288	3.444	-0.652	H

END

CONECT	1	2	37	89	97
CONECT	2	1	3	4	43
CONECT	3	2			
CONECT	4	2			

```
CONECT    5    6   37   43
CONECT    6    5    7   38
CONECT    7    6    8    9
CONECT    8    7
CONECT    9    7   10   11
CONECT   10    9
CONECT   11    9   12   13
CONECT   12   11
CONECT   13   11   14   38
CONECT   14   13   39   44
CONECT   15   16   17   18   44
CONECT   16   15
CONECT   17   15
CONECT   18   15   39   93   98
CONECT   19   20   42   81  100
CONECT   20   19   21   22   46
CONECT   21   20
CONECT   22   20
CONECT   23   24   42   46
CONECT   24   23   25   41
CONECT   25   24   26   27
CONECT   26   25
CONECT   27   25   28   29
CONECT   28   27
CONECT   29   27   30   31
CONECT   30   29
CONECT   31   29   32   41
CONECT   32   31   40   45
CONECT   33   34   35   36   45
CONECT   34   33
CONECT   35   33
CONECT   36   33   40   85   99
CONECT   37    1    5
CONECT   38   13    6
CONECT   39   14   18
CONECT   40   32   36
```

CONECT	41	24	31		
CONECT	42	23	19		
CONECT	43	5	2		
CONECT	44	14	15		
CONECT	45	32	33		
CONECT	46	23	20		
CONECT	47	48	49		
CONECT	48	47	49		
CONECT	49	47	48	50	
CONECT	50	49	51		
CONECT	51	50	52	53	
CONECT	52	51			
CONECT	53	51	54	55	
CONECT	54	53	56	57	
CONECT	55	53	58	63	
CONECT	56	54	59	60	
CONECT	57	54			
CONECT	58	55	59	61	
CONECT	59	56	58	62	
CONECT	60	56			
CONECT	61	58			
CONECT	62	59			
CONECT	63	55	64		
CONECT	64	63	65	66	67
CONECT	65	64			
CONECT	66	64			
CONECT	67	64	68	69	
CONECT	68	67	70	71	
CONECT	69	67	72	73	
CONECT	70	68			
CONECT	71	68	74	75	
CONECT	72	69			
CONECT	73	69	75	76	
CONECT	74	71			
CONECT	75	71	73	77	
CONECT	76	73			

```
CONECT   77   75   78   79   80
CONECT   78   77
CONECT   79   77
CONECT   80   77
CONECT   81   19   82   83   84
CONECT   82   81
CONECT   83   81
CONECT   84   81
CONECT   85   36   86   87   88
CONECT   86   85
CONECT   87   85
CONECT   88   85
CONECT   89    1   90   91   92
CONECT   90   89
CONECT   91   89
CONECT   92   89
CONECT   93   18   94   95   96
CONECT   94   93
CONECT   95   93
CONECT   96   93
CONECT   97    1
CONECT   98   18
CONECT   99   36
CONECT  100   19
```
